# Visible light-induced palladium–carbon bond weakening in catalytically relevant T-shaped complexes†

**DOI:** 10.1039/d3sc02588h

**Published:** 2023-11-28

**Authors:** Peter M. Waddell, Lei Tian, Anthony R. Scavuzzo, Lalu Venigalla, Gregory D. Scholes, Brad P. Carrow

**Affiliations:** a Department of Chemistry, Princeton University Princeton NJ 08544 USA; b Department of Chemistry, University of Houston Houston TX 77204 USA bcarrow@uh.edu

## Abstract

Triggering one-electron redox processes during palladium catalysis holds the potential to unlock new reaction mechanisms and synthetic methods not previously accessible in the typical two-electron reaction manifolds that dominate palladium catalysis. We report that T-shaped organopalladium(ii) complexes coordinated by a bulky monophosphine, a class of organometallic intermediate featured in a range of contemporary catalytic reactions, undergo blue light-promoted bond weakening leading to mild and efficient homolytic cleavage of strong Pd(ii)–C(sp^3^) bonds under ambient conditions. The origin of light-triggered radical formation in these systems, which lack an obvious ligand-based chromophore (*i.e.*, π-systems), was investigated using a combination of DFT calculations, photoactinometry, and transient absorption spectroscopy. The available data suggest T-shaped organopalladium(ii) complexes manifest unusual blue light-accessible Pd-to-C(sp^3^) transition. The quantum efficiency and excited state lifetime of this process were unexpectedly superior compared to a prototypical (α-diimine)Pd(ii) complex featuring a low-lying, ligand-centered LUMO (π*). These results suggest coordinatively-unsaturated organopalladium(ii) compounds, catalysts in myriad catalytic processes, have untapped potential for one-electron reactivity under visible light excitation.

## Abstract 2

Triggering one-electron redox processes during palladium catalysis holds the potential to unlock new reaction mechanisms and synthetic methods not previously accessible in the typical two-electron reaction manifolds that dominate palladium catalysis.

## Graphical abstract

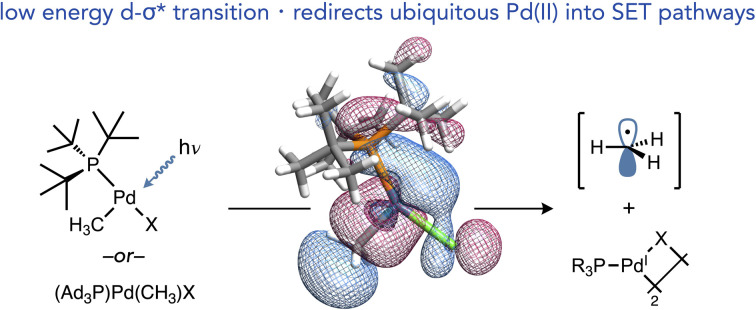

## Introduction

The photophysical properties of transition metal complexes have for many years been the subject of intense interest and have been exploited for a myriad of applications, from synthetic and preparative chemistry to artificial photosynthesis.^1–9^ A variety of electronic transitions are possible when a transition metal complex absorbs light. For metal complexes with 1 to 9 d electrons, d–d transitions can occur.^10,11^ Another important class of electronic transition is metal-to-ligand charge transfer (MLCT), which involves transitions from a d-orbital to low-lying antibonding orbitals on the ligands (*e.g.*, π*) and generally feature intense absorption bands. The resulting charge separation can engender reactivity not accessible in the ground state. For example, [Ru(2,2′-bipyridine)_3_]^2+^ is both a better oxidant and better reductant in the excited state, and this characteristic is an important feature of the photocatalysts used in photoredox processes.^4,6,12–14^

Depending on the nature of the orbitals involved in the electronic transition (*i.e.* bonding, antibonding or nonbonding), excitation may strengthen or weaken bonds in the complex and lead to chemical reactivity. A classic example of this phenomenon is photoinduced CO dissociation from metal carbonyl complexes (*e.g.*, M(CO)_6_, M = Cr, Mo, W).^15–20^ In this case, absorption of a photon promotes a transition from an orbital that is π-bonding to an orbital that is σ-antibonding with respect to the M–L bond. Excited state reactivity in metal complexes has emerged as a broadly useful concept in synthetic chemistry.^2,21^ As a corollary, the discovery of previously unknown excited state reactivity, particularly with complexes with established utility in catalysis, holds the potential to enable novel and interesting transformations. In this regard, palladium is one of the most used transition metals in catalysis yet the large majority of processes developed using Pd catalysts operate under ground state conditions. While several photoredox systems have been developed which use Ru or Ir photosensitizers to absorb visible light and subsequently perform electron or energy transfer with Pd or Ni catalysts, transformations that take advantage of direct photoexcitation of Pd catalysis are less explored.^22–38^

**Scheme 1:**
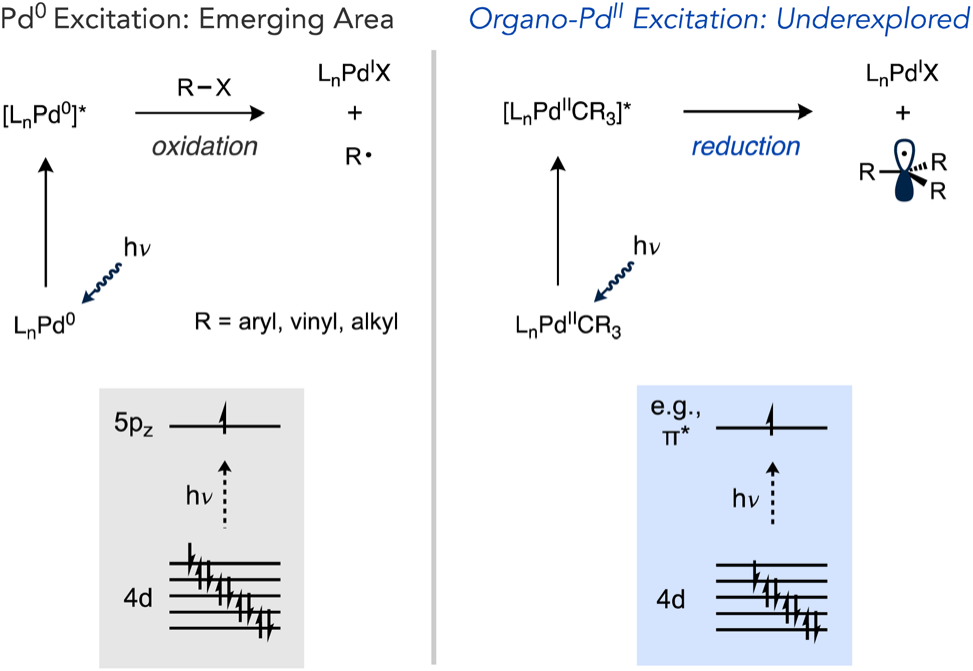
Photochemical excitation of Pd(0) *versus* Pd(ii).

The interaction of visible light with Pd(0) complexes has recently been advanced by the groups of Gevorgyan, Shang and Fu, Ryu and others as a method for one-electron reduction of organic halides that departs from the typical strong preference of Pd(0) to react by two-electron oxidative addition mechanisms (Scheme 1).^39^ This manifold has been used in a wide range of reactions including Heck and Negishi-type couplings, the coupling of alkyl halides with C(sp^2^)–H and C(sp^3^)–H sites, desaturations, tandem reactions involving the cyclization of radical intermediates with carbonylation, C–C coupling, and others.^40–56^ However, these examples only alter catalysis at the oxidative addition step.

**Scheme 2:**
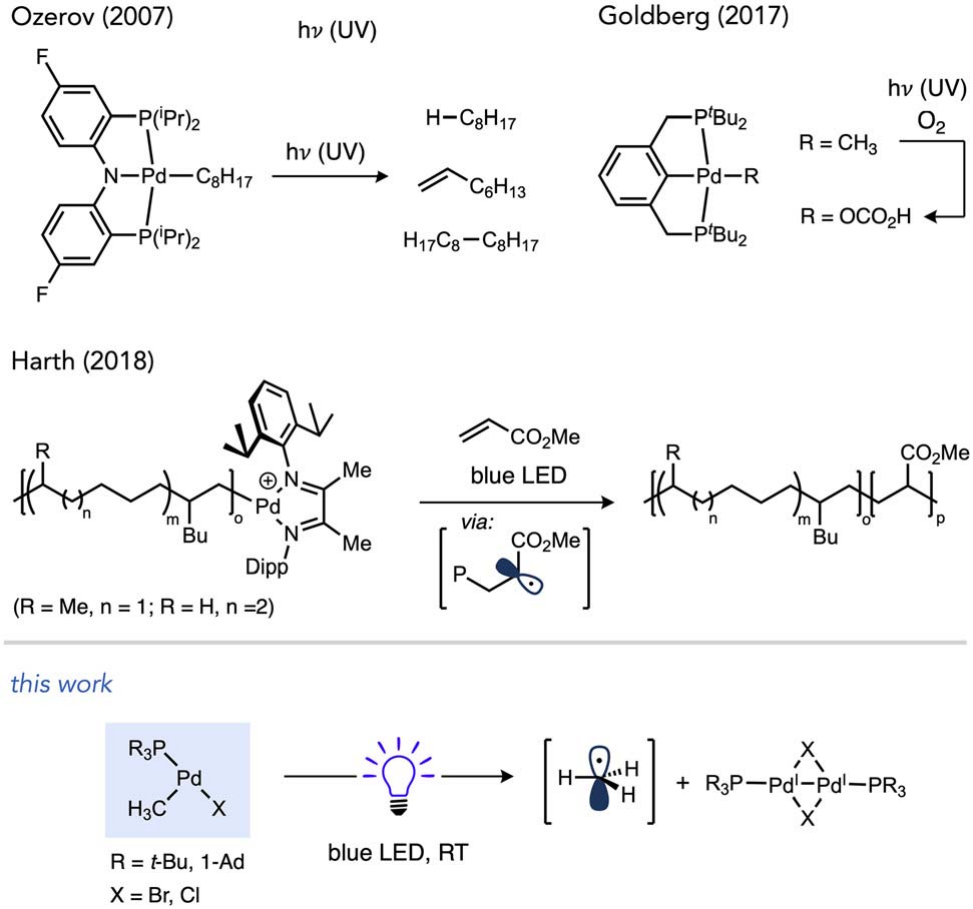
Representative examples of Pd(ii)–C photocleavage from organo–Pd(ii) complexes.

Light-promoted reactivity from organo–Pd(ii) species, on the other hand, represents a less explored aspect that could expand the utility of one-electron reactivity in Pd catalysis,^57–59^ especially considering these intermediates can be accessed not just by oxidative addition but other elementary reactions as well (*e.g.*, transmetalation, migratory insertion, C–H activation, *etc.*). Prior studies have established the potential for organopalladium complexes to undergo photochemical reactions (Scheme 2). Complexes featuring tri- or tetradentate ancillary ligands, such as PNP or PCP pincer ligands in independent studies by Ozerov and Goldberg or N_4_ ligands by Mirica undergo Pd–C cleavage under UV irradiation.^60–63^ Photochemical Pd(ii)–C scission in (bisphosphine)Pd complexes has also been observed, which occurred using visible light in cases of weaker metal–acyl bonds in work by Arndtsen while stronger Pd(ii)–alkyl bonds required UV light.^64–66^

Recently, Harth and co-workers reported that Pd catalysts featuring a redox active α-diimine ligand were able to liberate free radicals during olefin polymerization upon irradiation with blue LEDs. This phenomenon was exploited to prepare block copolymers by light-induced switching from isomerizing coordination-insertion polymerization of 1-hexene in the dark to radical polymerization of an acrylate under visible light irradiation.^67–69^ Accessing mild, visible light-inducted photochemistry with organo–Pd complexes coordinated by privileged ligand classes would be desirable to more broadly exploit the utility of light-induced one-electron chemistry in Pd catalysis.

Photochemical reactions of coordinatively unsaturated organopalladium complexes lacks precedent even though sterically hindered alkylphosphines that generate such species are some of the most frequently used ancillary ligands in modern Pd chemistry. The high donicity and the steric pressure of these ligands facilitates catalyst stability by resisting deactivation *via* deligation and accelerates reductive elimination, respectively.^70–76^ Tri-*tert*-butylphosphine in particular has been applied extensively across many contemporary synthetic organic methods, including C–C bond-forming reactions such as Migita–Kosugi–Stille, Suzuki–Miyaura, Sonogashira, Murahashi and Mizoroki–Heck reactions, as well as Buchwald–Hartwig amination and related C–O cross-coupling reactions.^77–85^ A related bulky monophosphine Ad_3_P (Ad = 1-adamantyl) has also emerged recently as an effective ligand for challenging Pd- and Ni-catalysed reactions.^86–92^ Furthermore, complexes such as (^*t*^Bu_3_P)Pd(CH_3_)Cl (1a) have been demonstrated as catalysts for alkene insertion (co)polymerizations in a living fashion.^93–95^ In this study we report on a serendipitous discovery that T-shaped organo–Pd complexes ligated by ^*t*^Bu_3_P or Ad_3_P undergo efficient visible light-induced bond weakening, which induces homolysis of otherwise strong Pd(ii)–C(sp^3^) bonds under ambient conditions. Experiments and computations in this study shed light on the nature and fate of the relevant excited state involved in these processes.

## Results and discussion

### Visible light photochemistry of T-shaped complexes

**Scheme 3:**
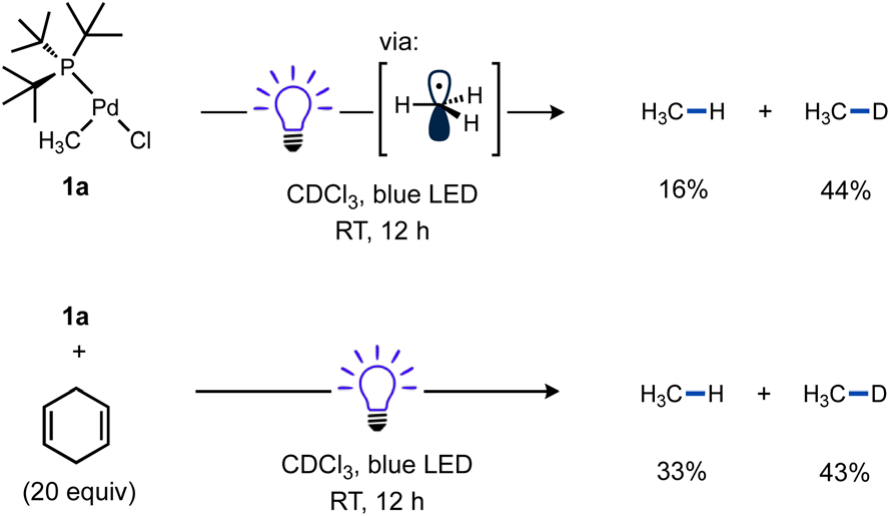
Reactivity of T-shaped complexes under blue LED irradiation. Cyclohexane was included as a ^1^H NMR standard. In the reaction with 1,4-cyclohexadiene (20 equiv.), Bu_4_P^+^BF_4_^−^ was also included as a ^31^P NMR standard. A fan was used to cool the reaction mixtures. Yields determined by NMR. See ESI† (p. S28) for full details.

The known T-shaped complex (^*t*^Bu_3_P)Pd(CH_3_)Cl (1a)^96^ is a bench-stable solid under ambient light. It is also indefinitely stable in CDCl_3_ in the dark. However, we found that irradiation of a CDCl_3_ solution of 1a with blue LED resulted in the formation of CH_4_ (16%) and CH_3_D (44%) over 12 h at RT, as determined by ^1^H NMR *versus* cyclohexane as an internal standard (Scheme 3). This observation is indicative of the formation of methyl radicals and their subsequent trapping by hydrogen atom abstraction, likely from the phosphine ligand and/or solvent. The addition of 1,4-cyclohexadiene as an H-atom donor led to an increase in the yield of CH_4_ (33%) and is also consistent with methyl radical formation.^97^

**Fig. 1:**
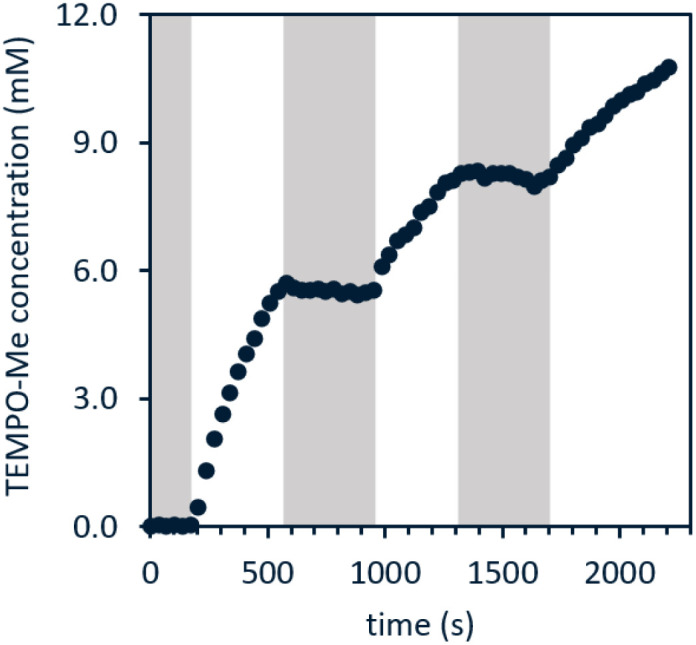
Light on–off study of 1a in the presence of excess TEMPO; white indicates blue LED irradiation while grey indicates dark. Reaction performed using fiber optic insert in NMR probe, see ESI page S47† for full details.

A reaction mixture of 1a and excess TEMPO, a common trap for radicals, was used to monitor evolution of methyl radical over time. Reaction progress of this mixture upon irradiation with blue LEDs was monitored using ^1^H and ^31^P NMR spectroscopy. Consumption of the starting material occurred cleanly along with formation of TEMPO–CH_3_ as the sole observable organic product in all cases (Fig. 1). No reactivity was observed in the absence of light when irradiation was intermittently ceased, demonstrating that the system is well-behaved and inconsistent with a sustained radical chain process.

**Scheme 4:**
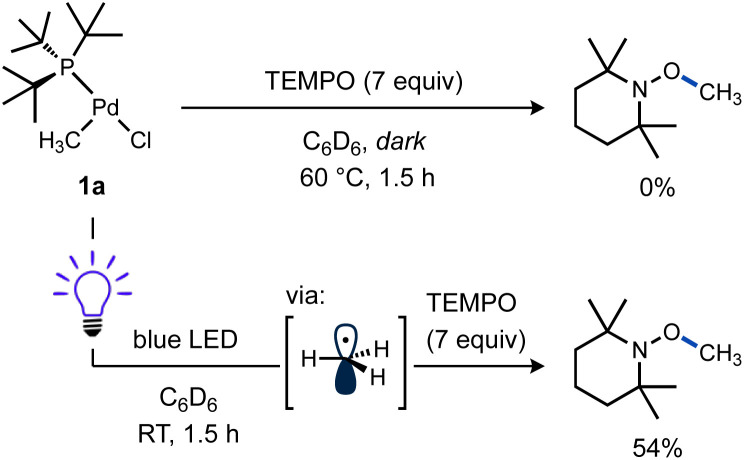
Reactivity of T-shaped complexes under blue LED irradiation with TEMPO. 1,3,5-(CF_3_)_3_C_6_H_3_ and Bu_4_P^+^BF_4_^−^ were included as ^1^H and ^31^P NMR standards. A fan was used to cool the reaction mixture. Yields determined by NMR. The photochemical reaction was run to 64% yield, as determined by ^31^P NMR.

Additional experiments were performed to test the possibility of thermal reaction caused by heat from the LEDs, despite the use of cooling fans in these reactions. A mixture of 1a and TEMPO remained unchanged in the dark at RT after 5 h as observed by ^1^H and ^31^P NMR. Even heating at 60 °C for 1.5 h gave no significant spectroscopic changes (Scheme 4, top). In contrast, another sample of 1a and TEMPO kept at RT while irradiating for 1.5 h yielded a comparable amount of TEMPO–CH_3_ as the previous reactions (Scheme 4, bottom). These results support the requirement for visible light to form methyl radicals from 1a and the absence of a significant thermal background reaction.

**Scheme 5:**
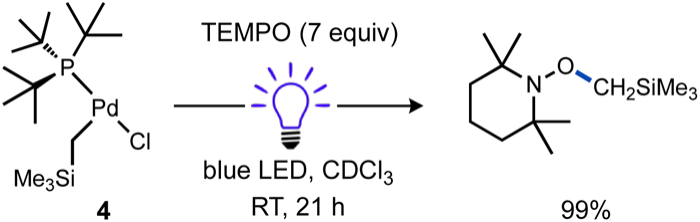
Reactivity of T-shaped Pd complex with a weaker Pd–C bond. 1,3,5-(CF_3_)_3_C_6_H_3_ and Bu_4_P^+^BF_4_^−^ were included as ^1^H and ^31^P NMR standards. A fan was used to cool the reaction mixture. Yield determined by NMR.

Considering the strong Pd(ii)–CH_3_ bond^98–100^ was effectively cleaved, it should be expected other (weaker) Pd(ii)–C(sp^3^) bonds would also be susceptible to homolysis under blue light irradiation. To test this idea, a new T-shaped complex (4) bearing a neosilyl group was prepared. Complex 4 did indeed react with TEMPO upon blue LED irradiation and generated the corresponding radical trapping product TEMPO–CH_2_SiMe_3_ in nearly quantitative yield as determined by ^1^H NMR (Scheme 5). Formation of an unidentified Pd product 5a (*vide infra*) in high yield was also observed by ^31^P NMR.

**Scheme 6:**
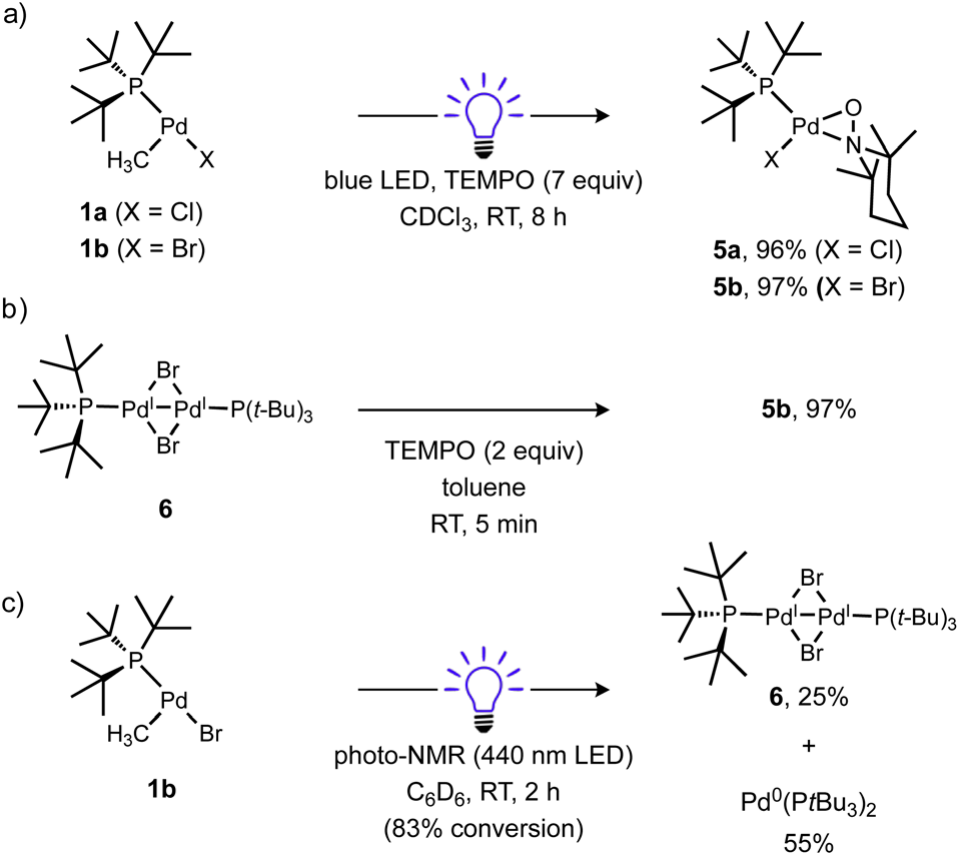
Identity of Pd product of irradiation of T-shaped complexes in the absence and presence of excess TEMPO. In (a), cyclohexane and Bu_4_P^+^BF_4_^−^ were included as NMR standards, the reactions were cooled with a fan. Yields were determined by NMR for (a) and (c). In (b), yield was determined gravimetrically. Yield of 6 based on total Pd.

The fate of the Pd product generated after Pd(ii)–C bond cleavage was also investigated, which presumably involves a one-electron reduction of Pd(ii) upon alkyl radical ejection. Conveniently, [(^*t*^Bu_3_P)Pd^I^(μ-X)]_2_ (X = Br, I) species are stable diamagnetic complexes, although the chloride-bridged congener is unknown.^101,102^ For this reason, the T-shaped complex with a bromide ligand (1b) was also prepared and investigated. Formation of these stable dimers from the putative mononuclear Pd(i) generated under photochemical conditions should be spectroscopically observable, but consumption of Pd(i) species prior to dimerization might also occur, such as by disproportionation. Examination of the ^31^P NMR spectrum of the reaction of 1a (*δ*_P_ 70.0 ppm) with TEMPO under blue light irradiation (Scheme 6a) indicated clean formation of a new phosphorus-containing species 5a (*δ*_P_ 88.1 ppm) in 96% yield. Analogous experiments using either 1b (*δ*_P_ 68.4 ppm) or the Ad_3_P-coordinated congener 2 (*δ*_P_ 53.2 ppm) also generated new ^31^P NMR resonances at *δ*_P_ 90.4 (5b) and 76.9 ppm (S2) in high yields (96% and 95%), respectively. The yield of TEMPO–CH_3_ was also high in all cases (70–89%). Importantly, the ^31^P NMR resonance of product 5b does not match the reported shift for [(^*t*^Bu_3_P)Pd^I^(μ-Br)]_2_6 (*δ*_P_ 86.3 ppm)^103^ indicating a unique Pd product formed under these conditions.

**Fig. 2:**
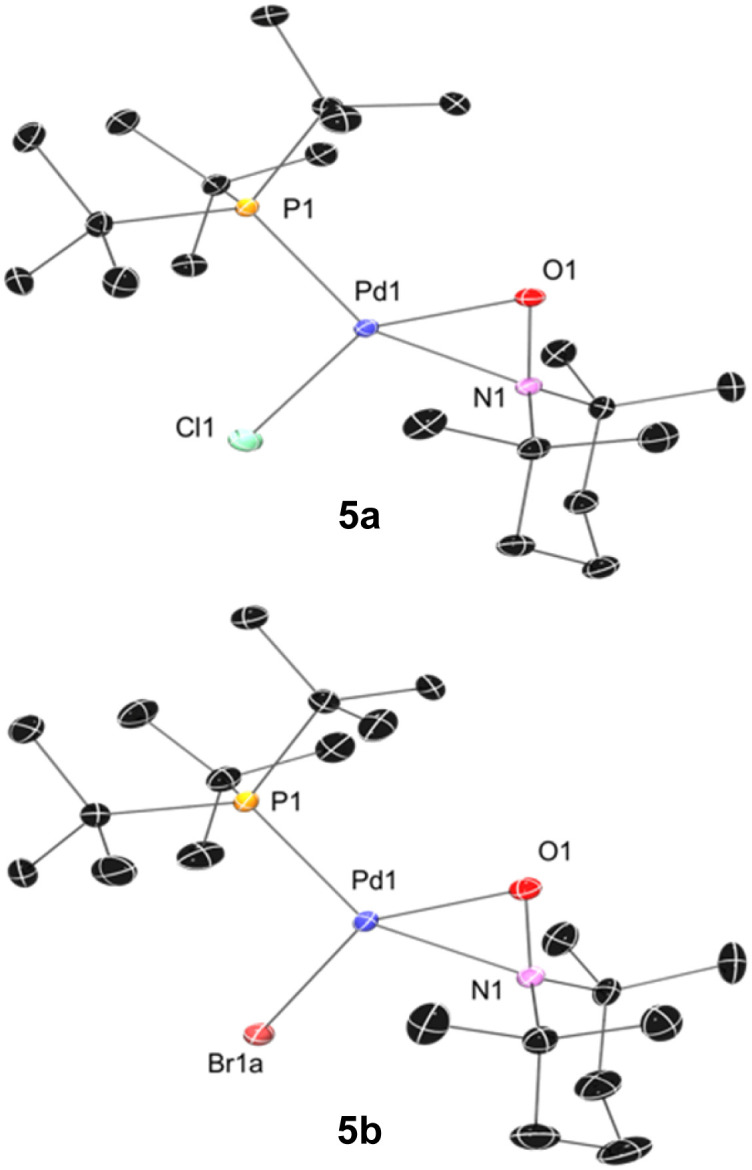
Solid state structure of 5a (left) and 5b (right) (50% thermal ellipsoids; H atoms omitted). Selected bond lengths (Å) and angles (deg) for 5a: Pd1–N1 = 2.0882(6); Pd1–O1 = 2.0415(6); N1–O1 = 1.3611(8); Pd1–P1 = 2.3082(4); Pd1–Cl1 = 2.3461(5); P1–Pd1–O1 = 112.09(2); O1–Pd1–N1 = 38.47(2); N1–Pd1–Cl1 = 106.56(2); Cl1–Pd1–P1 = 102.83(1). For 5b: Pd1–N1 = 2.109(1); Pd1–O1 = 2.036(1); N1–O1 = 1.3642(7); Pd1–P1 = 2.3099(6); Pd1–Br1A = 2.482(2); P1–Pd1–O1 = 110.77(3); O1–Pd1–N1 = 38.38(3); N1–Pd1–Br1a = 106.28(5); Br1a–Pd1–P1 = 104.34(4) (disorder in Br and P methyl carbons omitted).

We subsequently found that the combination of Pd(i) complex 6 and TEMPO led to an immediate colour change upon mixing and formed 5b in 97% yield (Scheme 6b). The solid-state structure of 5b subsequently revealed this complex to be a Pd complex coordinated by TEMPO in an η^2^-*N*,*O* fashion, which has been observed in a few other cases and comparable to known η^2^-nitrosoarene adducts.^69,104–106^ The solid-state structures of 5a (as illustrated in Fig. 2) and an analogous Ad_3_P-coordinated complex S2 (see Fig. S206†) were obtained from photochemical reactions of 1a and 2 respectively. Each of these complexes is indefinitely stable under air at room temperature. Based on the similarity of Pd–N and Pd–O bond lengths (±0.04 Å) and N–Pd–O bond angles (±0.2°) in the η^2^-TEMPO complexes 5a, 5b, and S2, and the reference compounds with three membered rings of Pd(ii), N and O reported by Figueroa and Harth, we likewise assign these as Pd(ii) complexes.^69,106^ This is consistent with our hypothesis that a Pd(i) species forms as the immediate transition metal product following light-induced Pd(ii)–C homolytic cleavage, which then undergoes rapid one-electron oxidation by the excess TEMPO present under these reaction conditions.

A different product distribution was formed when 1b was irradiated in the absence of TEMPO. Under these conditions formation of the Pd(i) complex 6 can be detected by ^31^P NMR (Scheme 6c). Complex 6 gradually disappears with concomitant formation of (^*t*^Bu_3_P)_2_Pd(0) over time, possibly due to a disproportionation side reaction. The latter complex eventually becomes the dominant species observable by ^31^P NMR.

**Scheme 7:**
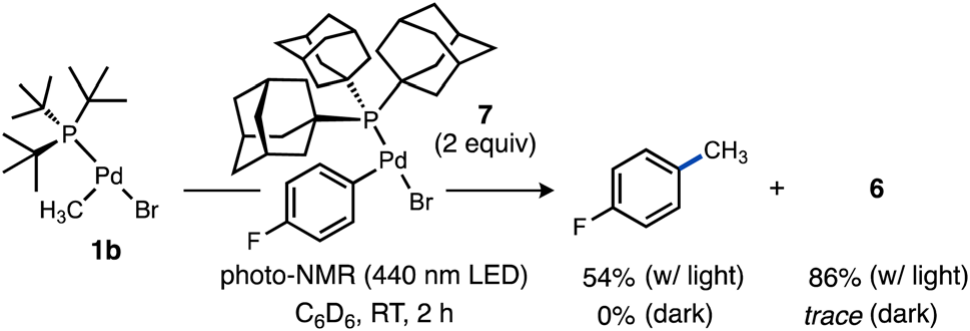
Observation of light-induced, Pd-to-Pd radical-based transmetalation. Additionally, 1,4-dioxane and Bu_4_P^+^BF_4_^−^ were included as NMR standards. Yields determined by NMR. Yield of 6 based on total Pd.

We were curious to test if alkyl radicals generated from Pd–C photocleavage could be trapped not only by radical scavengers but by another organometallic complex leading eventually to formation of a C–C cross-coupling product from the putative higher-valent metal species generated upon radical attack. We selected a candidate complex (Ad_3_P)Pd(*p*-C_6_H_4_F)Br (7) featuring a stronger Pd–C(sp^2^) bond that should be less susceptible to photolysis. Upon irradiation of 1b in the presence of 7, formation of 4-fluorotoluene (54%) was observed (Scheme 7). Interestingly, the yield of the Pd(i) dimer 6 was significantly higher (86%) under these conditions, likely due to complex 7 being a much weaker oxidant than TEMPO. Because no 4-fluorotoluene formation was observed in the absence of light, we propose methyl radical generated from photoexcited 1b is captured by 7 to generate a transient Pd(iii) intermediate that undergoes facile reductive elimination of 4-fluorotoluene. This light-enabled Pd-to-Pd transmetalation highlights the potential for visible light to stimulate intermetallic reactivity not observed in ground state Pd chemistry. This experiment also validates that the reduced Pd species can be generated efficiently under certain photolytic conditions, which bodes well for the potential for integrating this light-driven chemistry into novel catalytic reactions considering the established intermediacy of Pd(i) species in numerous Pd-catalysed reactions.^107–112^

Methyl radical generation from these monophosphine-ligated T-shaped complexes might be expected to occur with lower photochemical efficiency due to the lack of typical low-lying ligand-based acceptor orbitals (*e.g.*, π*) commonly associated with MLCT in transition metal photochemistry.^13,113–117^ Nevertheless, quantum yield measurements for reactions of 1a and related T-shaped complexes revealed light-induced Pd(ii)–C homolysis from these species can in fact be more efficient as compared to prototypical photoactive compounds, such as (α-diimine)Pd(ii) complexes.^67^

Quantum yields for Pd(ii)–C photocleavage were determined using photo-NMR actinometry (see ESI† for full experimental details) according to the method reported by Ji and co-workers.^118^ Reactions were performed using either of two representative complexes, 1a or [ArN = C(Me)C(Me) = NAr]Pd(CH_3_)Cl 3 (Ar = 2,6-^*i*^Pr_2_C_6_H_3_). The similarity of the reaction rates conducted at two different concentrations ([Pd]_o_ = 60 mM or 120 mM for 1a, [Pd]_o_ = 30 mM or 60 mM for 3) in the presence of TEMPO (7 equiv.) were consistent with zeroth-order dependence on [Pd] and consequently occurs within a photon-limited kinetic regime inside this concentration range. The quantum yield (*Φ*_obs_ = *k*_pc,0_/*I*_0_ where *k*_pc,0_ is the observed initial rate of Pd–C photocleavage, and *I*_0_ is the photon flux determined by actinometry, see ESI† for full details) for 1a under these conditions was calculated to be 0.67 (Table 1, entry 1). Analogous experiments conducted in C_6_D_6_ or MeCN-d_3_ indicated a weak dependence of quantum yield on solvent identity (see ESI,† page S86). The related Ad_3_P–ligated complex 2 gave *Φ* = 0.36 for formation of TEMPO–CH_3_ in CDCl_3_ (Table 1, entry 3). Importantly, the quantum yields for both T-shaped complexes are higher compared to the reference complex 3 (*Φ* = 0.11) under otherwise identical conditions (Table 1, entry 5). Note that repetition of these measurements in the absence of TEMPO, which absorbs light in the visible region^119^ and might interfere with quantum yield measurements, gave only a modest increase for T-shaped 1a (*Φ* = 0.67–0.73) or α-diimine complex 3 (*Φ* = 0.11–0.16) as shown in Table 1, entries 1 *vs.* 2 or 4 *vs.* 5. We conducted several additional experiments and computations to understand the origin of the counterintuitive trend of greater quantum efficiency for the T-shaped complexes compared to the diimine–ligated complex.

**Table 1:** Determination of quantum yield for Pd(ii)–CH_3_ photocleavage^a^

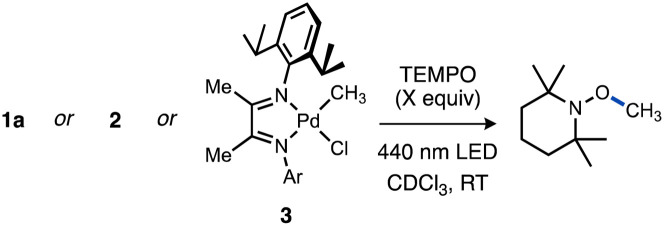			
Entry	Complex	*X*	*Φ*_obs_
1	1a	7	0.67
2	1a	0	0.73
3	2	7	0.36
4	3	7	0.11
5	3	0	0.16

a 1,3,5-Tris(trifluoromethyl)benzene was included as NMR standard. Reactions monitored by NMR. In the reactions with TEMPO, the rate of TEMPO–CH_3_ evolution was used to calculate *Φ*_obs_, while in the reactions without TEMPO, the rate of consumption of Pd–CH_3_ was used to calculate *Φ*_obs_.

It could be the case that differences in the excited state energy profiles of 1a and 3 might give rise to a discrepancy in activation energy for metal–carbon bond homolysis in these two representative species. To probe this possibility, the temperature dependence of photocleavage quantum yield was used to calculate an activation energy barrier for each photochemical reaction.1
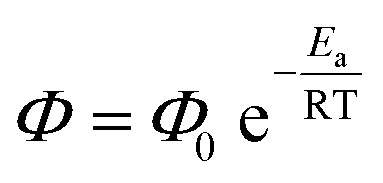


**Fig. 3:**
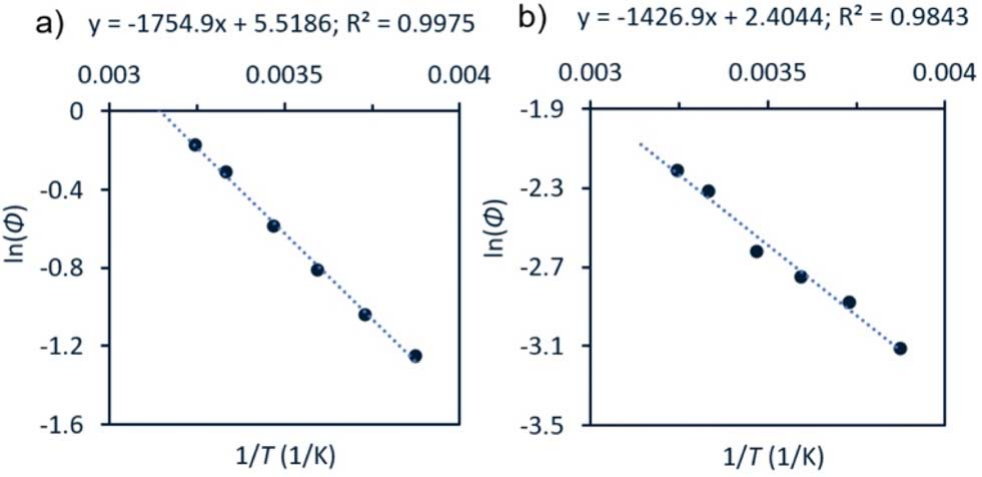
Arrhenius-type analyses for photochemical reaction of 1a or 3 with TEMPO. Conditions: a solution of Pd complex (60 mM for 1a, (a); 30 mM for 3, (b)) and TEMPO (7 equiv.) in CDCl_3_ were irradiated at room temperature using photo-NMR (440 nm LED). The initial rate was taken from each reaction and used to calculate *Φ*. See ESI,† page S111 for full details.

An Arrhenius-type plot (Fig. 3) was constructed according to eqn (1) for reactions of 1a or 3 with TEMPO monitored by photo-NMR spectroscopy (440 nm LED) at six different temperatures.^117^ The calculated activation energies for these reactions were *E*_a_ = 4 kcal mol^−1^ and 3 kcal mol^−1^ for 1a and 3, respectively. The similarity of these small energy barriers for Pd(ii)–C cleavage at the excited state therefore does not account for the observed differences in quantum yield between the two types of organo–Pd complex. Additional computational and spectroscopic investigations were informative in parsing other possible origins of efficiency differences.

### Excited state analysis by DFT calculations

**Fig. 4:**
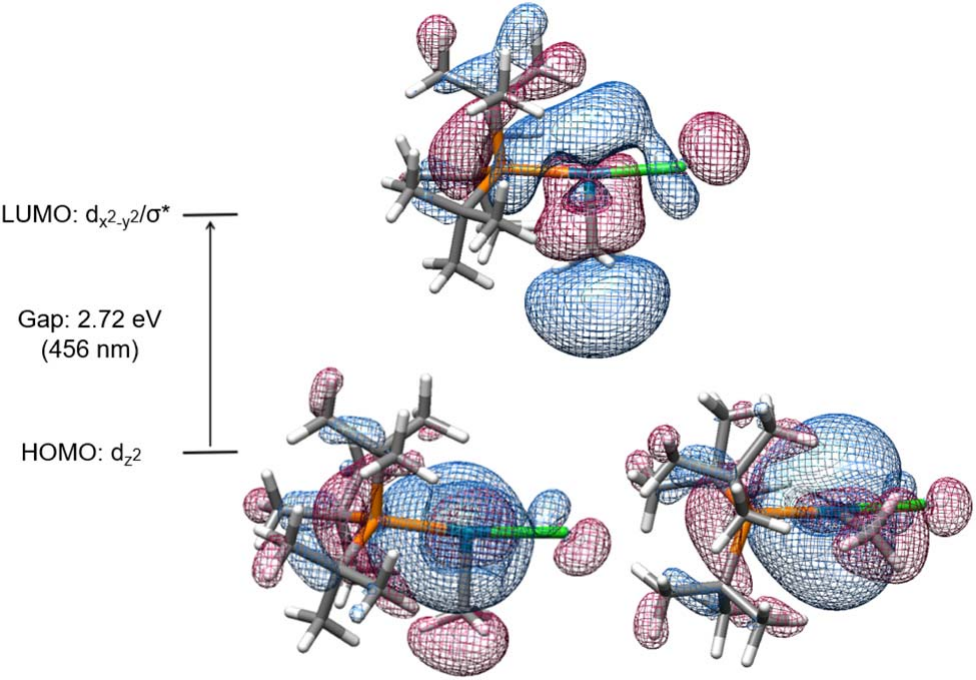
Simplified energy level diagram of HOMO (two views) and LUMO of 1a.

The electronic transitions that could facilitate Pd(ii)–C homolytic cleavage were investigated by density functional theory (DFT) calculations using complex 1a as a representative case. Calculations were performed using various functionals (*vide infra*), the 6-311G++(d,p)/LANL2DZ(Pd) GD3 basis set and solvent corrected with the SMD(CHCl_3_) solvent model. Agreement between simulated and experimental (Fig. S172†) UV-vis spectra was used as a criterion for computational method optimization.^120^ The general features of the spectrum are matched qualitatively in the simulated spectrum using CAM-B3LYP, including the absorbance in the 400–500 nm region,^121^ that overlaps the spectral range of blue LED output. A similar absorption spectrum was observed for 2 (Fig. S187†). The HOMO and LUMO of 1a are illustrated in Fig. 4, which suggests the HOMO orbital is predominantly dz^2^(Pd) in character and the LUMO features d_*x*^2^−*y*^2^_(Pd) and σ*(Pd(ii)–CH_3_) character.

Linear time-dependent density-functional theory (TD-DFT) calculations were also performed. From these calculations the HOMO–LUMO gap was calculated to be *ca.* 2.72 eV, equivalent to the energy of a 456 nm photon. We postulate the relatively low energy of a d_*x*^2^−*y*^2^_/σ* orbital in these complexes might be facilitated by the T-shaped geometry due to the absence of a ligand *trans* to the methyl ligand (and associated destabilizing *trans* influence). Excitation leading to population of an orbital with anti-bonding character would be expected to weaken the metal–ligand bond.

**Fig. 5:**
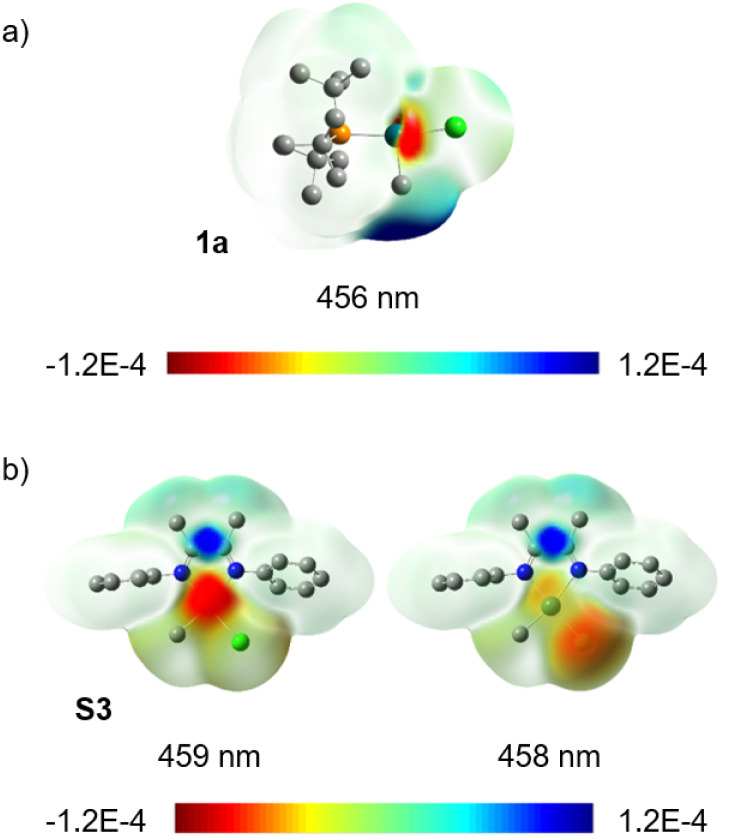
Difference density isosurfaces for transitions occurring at 459 nm and 458 nm for (a) 1a and (b) at 456 nm for S3. Units for scale: electrons per au^3^ (au is Hartree atomic units). Negative/red represents electron loss, positive/blue represents electron gain.

Population analysis using TD-DFT calculations indicates that excitation of 1a at 456 nm is accompanied by a decrease of the Pd(ii)–C bond order by 0.43. The difference density isosurface for this electronic transition (Fig. 5a) illustrates that the decrease in bond order is correlated to a decrease in electron density at Pd with a concomitant increase in electron density at the CH_3_ group by 0.32 e and 0.25 e, respectively (see ESI,† Table S22 for tabular data). We interpret these data as indicative of an uncommon Pd-to-CH_3_ MLCT-like process. We also note that extinction coefficient measurements at *λ*_ma*x*_ for this absorption (370 nm) indicate *ε* = 2.2 × 10^3^ M^−1^ cm^−1^, which is within a generally accepted range for transitions involving significant charge separation character (for 3, *ε* = 4.3 × 10^3^ M^−1^ cm^−1^, see ESI page S184†).^122–127^ This contrasts what has been proposed for the electronic transition underpinning the photochemistry of (α-diimine)Pd complexes, such as 3,^67,68^*via* traditional d-to-π* MLCT that populates an ancillary ligand-centred orbital. The potential to stimulate charge transfer to unactivated (*i.e.* lacking groups that stabilize radicals) alkyl groups of an organometallic complex highlights that π-based ancillary ligands are not essential for achieving mild, efficient photochemistry in Pd complexes. It may also be inferred that there may exist a reservoir of untapped photochemical reactivity in organo–Pd(ii) complexes previously presumed to be photochemically inert due to a lack of ancillary ligand-based chromophores.

For comparative purposes the truncated complex [PhN=C(Me)C(Me)=NPh]Pd(CH_3_)Cl (S3) was also examined. The optimal functional, based again on best agreement of experimental and calculated absorption spectra (see ESI,† Fig. S171), was M06 in this case. Difference density isosurface calculations for S3 confirmed an expected d-to-π* MLCT transition at 459 nm along with another transition at 458 nm which appears to have Cl-to-π* character (Fig. 5b). Population analysis of this (α-diimine)Pd(ii) system showed an average loss of 0.29 e at Pd and gain of 0.5 e on the diimine ligand (see ESI,† Table S20 for tabular data). A significant change in Pd(ii)–C bond order was not observed for this complex, which is not yet understood. One rationalization could be the need for thermalization of the Franck–Condon state, intersystem crossing and/or a change in geometry needed to reach another excited state (*e.g.*, S_1_ or T_1_) prior to weakening of the Pd(ii)–C bond. If true this would shorten the window of time where Pd–C cleavage is possible, and would therefore contribute to the difference in quantum yield between 3 and 1a.

**Fig. 6:**
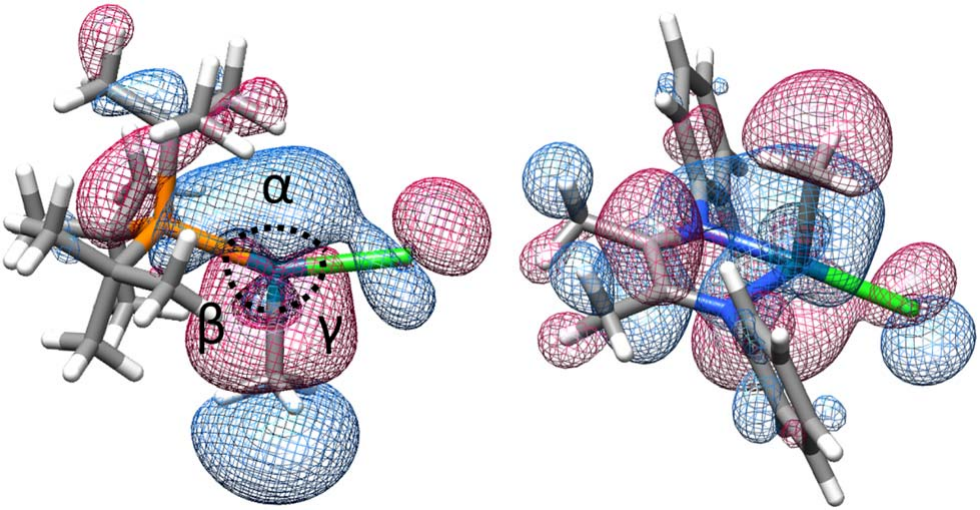
Optimized triplet excited state geometries (T_1_) for 1a (left) and S3 (right) showing the higher-energy SOMO.

Examination of excited state optimized geometries of representative T-shaped (1a) and α-diimine (S3) complexes revealed considerable differences in the type and degree of distortion between the two classes. The optimized geometry for the triplet excited state of 1a only subtly distorts from the ground state T-shaped geometry (*α* = 172°, *β* = 97° and *γ* = 91°)^128^ toward Y-shaped (*α* = 150°, *β* = 112° and *γ* = 98°). On the other hand, S3 shows a significant geometry change from square planar in the ground state (*τ*_4_ = 0.10) toward tetrahedral (*τ*_4_ = 0.58).^129^ The triplet state geometries and overlaid higher energy SOMO orbitals are illustrated in Fig. 6. The change in bond order between the S_0_ and T_1_ states was calculated to decrease by 0.49 and 0.11 for 1a and S3, respectively (see ESI,† Table S19 for tabular data). While the bond order change is modest for the (α-diimine)Pd complex, it may be consistent with the notion that bond weakening in these complexes may occur after thermalization from the Franck–Condon state. Unfortunately, extensive efforts to optimize the geometries of the lowest singlet excited state for 1a and S3 were unsuccessful. Considering the experimentally small activation barrier of Pd(ii)–C homolysis upon photoexcitation, a conical intersection between the potential energy surfaces between S_1_ and S_0_ may exist, which is a documented challenge for TD-DFT calculations.^130,131^ In any case, the optimized triplet states were subsequently used to estimate the free energy of bond weakening.

2 1a or 1b → ^*t*^Bu_3_Pd^I^X + H_3_C˙

3
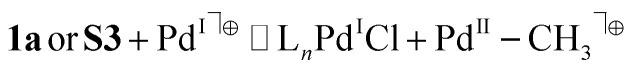
4
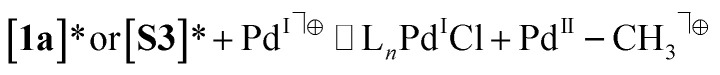


Based on homolytic Pd(ii)–C cleavage from the ground state T-shaped complexes summarized in eqn (2), the bond dissociation free energy and enthalpy were calculated to be BDFE (BDE) = 34.3 (47.0) and 33.5 (46.5) kcal mol^−1^ for T-shaped complexes 1a and 1b, respectively (DLPNO-CCSD(T)/def2-TZVPP//B3LYP-D3/6-311G++(d,p)/LANL2DZ(Pd); see ESI† for full details). These data are consistent with experimental observations that homolysis does not occur from the ground state under thermal conditions alone. Isodesmic reactions (eqn (3) and (4)) were also used to estimate the free energy change in the Pd(ii)–CH_3_ bond upon excitation from the ground to triplet state (see ESI,† page S139 for full details). These calculations used the experimentally determined BDFE for [Pd(ii)–CH_3_]^+^ (59 kcal mol^−1^) based on ion beam reactive scattering.^132^ Experimental BDFEs of more closely related Pd(ii)–CH_3_ complexes were not found, but relative energy changes benchmarked against this gas phase species are still informative. By this method the Pd(ii)–C BDFE in T-shaped 1a is estimated to weaken by 20 kcal mol^−1^ in the triplet excited state. Similarly, the Pd(ii)–C bond in α-diimine complex S3 is estimated to weaken by 28 kcal mol^−1^ in the triplet excited state. These data further corroborate the feasibility of facile homolysis of otherwise strong Pd(ii)–C(sp^3^) bonds upon visible light excitation at ambient temperature.

### Transient absorption spectroscopic measurements

The relevant excited state for T-shaped and α-diimine Pd complexes can potentially decay through radiative (luminescence) or other nonradiative decay (NRD) pathways (*i.e.*, solvent-mediated) that do not lead to radical formation, in addition to the pathway resulting in Pd(ii)–C homolytic cleavage. One of the former two processes might explain the discrepancies in quantum yield we observe for methyl radical formation from either 1a or 3 if its relative rate is fast enough to dominate the fate of the complex's excited state. We investigated the luminescence properties of these complexes to test this hypothesis. Luminescence measurements were complicated by sample decomposition due to the Pd(ii)–C homolysis process, as determined by changes in the absorption spectra even after a single scan. As such, data were collected using low beam power and the sample solution was replenished between each scan to minimize the influence of sample decomposition.^133^ Features consistent with luminescence emission were not detected from either 1a or the α-diimine complex 3. These data suggest radiative decay is a negligible relaxation pathway for both types of complexes.

**Fig. 7:**
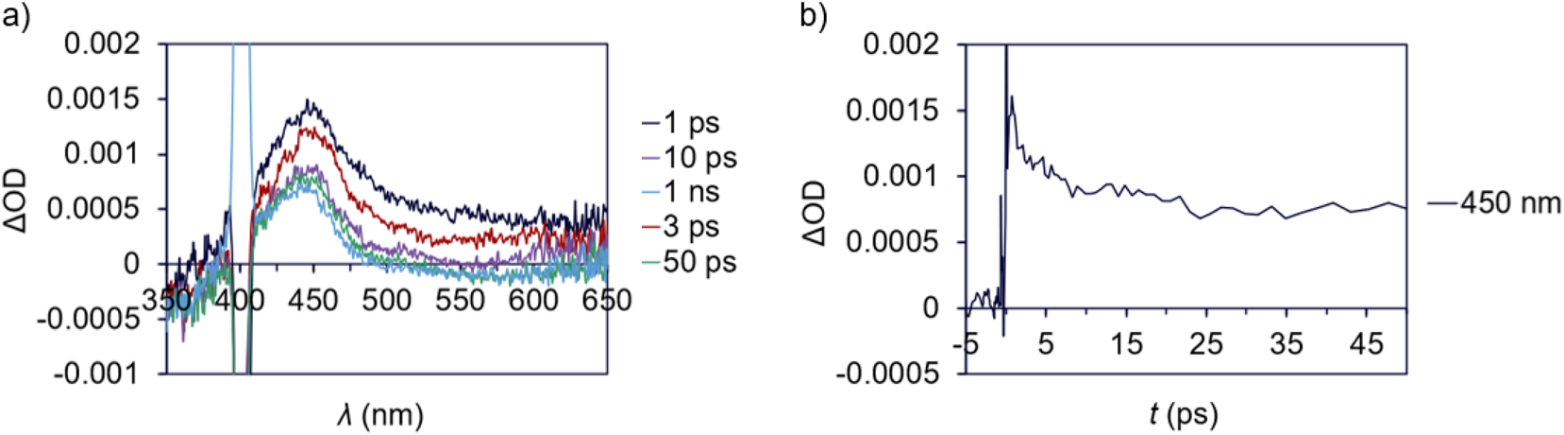
Transient absorption data for 1a (ΔOD = change in optical density). (a) TA difference spectrum for 1a in deoxygenated CDCl_3_, pumping at 400 nm (100 μW) at various delay times. Note the scattering feature around 400 nm. (b) Transient behaviour at 450 nm from 0 to 50 ps.

Transient absorption (TA) spectroscopy was used to study the excited state lifetimes of 1a and 3. These measurements were used to assess the relative rates of NRD between the T-shaped and α-diimine Pd complexes; since both complexes appear to have similar barriers to Pd–C cleavage and do not undergo significant luminescence decay, we hypothesize that differences in their excited state lifetimes are likely due to different rates of unproductive NRD. In addition, TA measurements can also provide information about the magnitude of charge transfer between ground and excited states.^134,135^ To ensure that the amount of photodegradation was minimized to the extent possible, the intensity of the laser was optimized and pre- and post-scan absorption measurements were performed. A ground state bleach from 375–410 nm is apparent for 1a (Fig. 7a) coinciding with the absorptive region in the absorption spectrum. A broad excited state absorption is also evident from 410 to beyond 610 nm, which decays within *ca.* 50 ps (Fig. 7b). This latter feature was assigned as the excited state decay of 1a. A long-lived feature peaked at 450 nm follows, which persists out to the nanosecond timescale. This is consistent with the difference absorption obtained from pre- and post-scan steady-state measurements (Fig. S199†). This feature is consequently assigned to a photodegradation process.

**Fig. 8:**
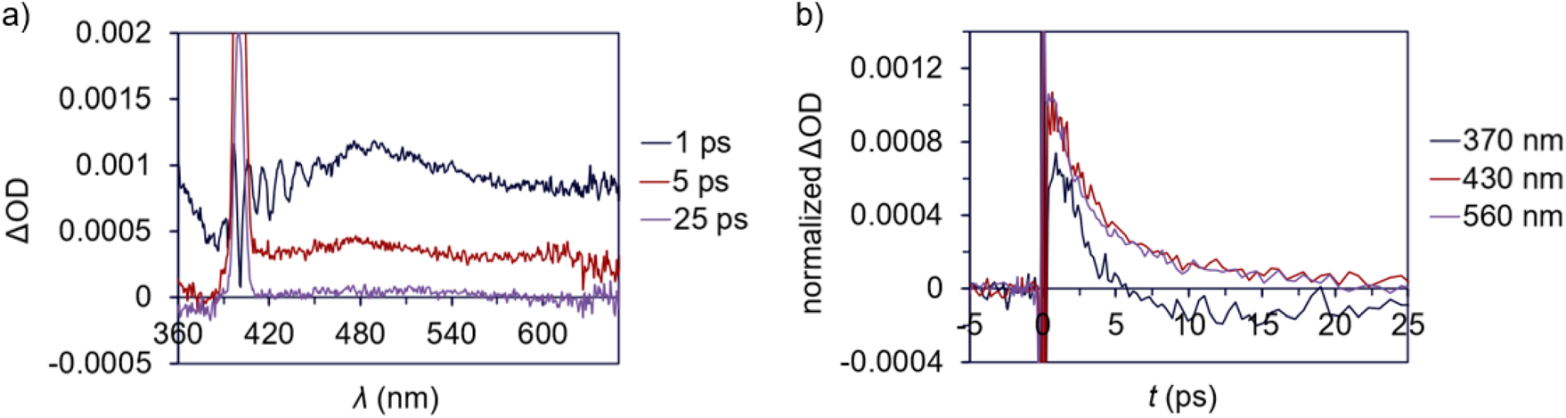
Transient absorption data for 3 (ΔOD = change in optical density). (a) TA difference spectrum for 3 in deoxygenated CDCl_3_, pumping at 400 nm (100 μW) at various delay times. Note the scattering feature around 400 nm. (b) Transient behaviour at 370, 430 and 560 nm from 0 to 25 ps.

A ground state bleach around 360–480 nm is apparent in the TA spectrum of 3 (Fig. 8a), which represents absorption into the MLCT band. The MLCT absorption is convoluted with a broad excited state absorption across the spectral range. The excited state absorption decays to baseline within 25 ps (Fig. 8b) suggesting a relatively low degree of photodegradation and is consistent with the modest quantum yield for Pd(ii)–C homolysis in 3*vs.*1a. This fast absorption decay for 3 also indicates a large proportion of the sample returns to the ground state.

The excited state lifetime of T-shaped complex 1a is about twofold longer than for α-diimine complex 3 based on the time constants of 6.4 and 3.1 ps, respectively, determined from TA analyses in CDCl_3_. Measurements taken in less polar solvent (benzene) yielded time constants of 14 ps for 1a and 3.9 ps for 3 (Fig. S197 and S195† respectively). There are no significant differences in the absorption spectra for either complex in CDCl_3_ compared to C_6_H_6_ (Fig. S198†). The higher sensitivity of the excited state lifetime of 1a to solvent polarity could reflect significant charge separation between the ground and excited state,^134–136^ which would be consistent with the substantial charge transfer suggested by difference density isosurface calculations for this complex (*vide supra*). Considering the absence of significant radiative decay for either complex and similarity of energy barriers toward Pd(ii)–C homolysis, we postulate the longer excited state lifetime of 1a may result from lower rates of unproductive NRD compared to 3 (*i.e.*, NRD that does not result in Pd(ii)–C homolysis). Longer excited state persistence would then account for higher quantum yield in the T-shaped complexes by extending the window of time in which thermal homolysis of the Pd(ii)–C bond can occur.

**Fig. 9:**
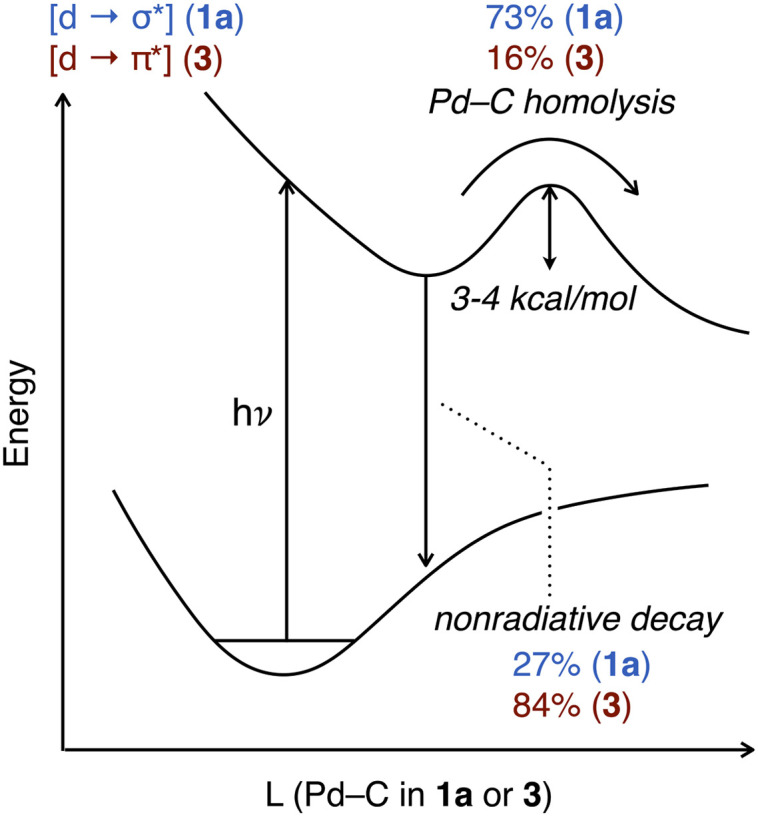
Proposed excited state potential energy curves for 1a and 3.

A qualitative diagram shown in Fig. 9 summarizes our current understanding of the evolution of the excited states for representative T-shaped and α-diimine organo–Pd(ii) complexes. Spin state and associated intersystem crossing details are omitted from this diagram; the dynamics of the TA transients are well-described by single time constants and are thus consistent with Pd(ii)–C cleavage from one excited state, but unambiguous assignment of the spin state based on the currently available data was precluded. Visible light excitation of 1a promotes an electron from a nonbonding orbital to one with σ*(Pd(ii)–C) character. Rupture of the Pd(ii)–C bond constitutes *ca.* 73% of the decay from the [Pd d → C(sp^3^) σ*] excited state based on quantum yield (*vide supra*). For this T-shaped complex the productive NRD effectively outcompetes other unproductive NRD pathways (*ca.* 27%) that recover the ground state.

Excitation of the α-diimine complex 3 into the MLCT band (∼350–500 nm) produces an excited state by population of the π* orbitals on the diimine. At present it is not definitively established whether this state leads to Pd–C cleavage, or whether this occurs after conversion to a lower-lying excited state, as has been proposed for (bpy)Ni aryl complexes.^122,137^ Our computational investigations suggest the Pd–C bond is not weakened in the Franck–Condon state of 3. Rather, some other processes such as thermalization, intersystem crossing and/or geometric changes occur prior to bond cleavage. We did not find this to be the case for 1a, which could be another contributing factor rationalizing the quantum yield differences between the two types of complexes. Homolysis of the Pd(ii)–C bond represents *ca.* 16% of the excited state decay based on quantum yield data with the remainder assigned to other unproductive NRD (*ca.* 84%). The origin of the discrepancy in unproductive NRD between the two types of organo–Pd(ii) complexes will require further investigation to understand. It is plausible 3 could possess stronger vibronic coupling to the solvent molecules relative to 1a, which would facilitate the dissipation of the energy of its excited state by unproductive NRD. It would also be interesting to understand what aspects of the ligand choice (alkylphosphine *vs.* diimine) and/or Pd(ii) geometry (T-shaped *vs.* square planar) influence the extent of unproductive NRD, which could guide the design of improved quantum efficiency in photochemical reactions of catalytically relevant organo–Pd(ii) complexes.

## Conclusions

We have found that visible light can promote mild yet efficient homolytic cleavage of the strong Pd(ii)–C bond in T-shaped organo–Pd(ii) compounds. Stoichiometric reactions and quantum yield measurements are consistent with free radical formation by a blue light-promoted, non-chain process. Kinetic data from photo-NMR analyses indicate the photophysical process occurs with a small thermal barrier (3–4 kcal mol^−1^). Electronic structure calculations of a model T-shaped (^*t*^Bu_3_P)Pd(CH_3_)Cl complex point to a mechanism involving charge transfer from Pd to the CH_3_ ligand. The strength of the metal–carbon bond in the resulting [Pd d → C(sp^3^) σ*] excited state is predicted to significantly weakened such that homolytic cleavage of an otherwise a strong organometallic bond occurs easily under ambient conditions. The result of this photochemical pathway is the formation of an alkyl radical along with a reduced Pd(i) intermediate.

A comparative analysis using a prototypical photoactive square planar (α-diimine)Pd(ii) complex also yielded the surprising observation of significantly lower quantum yield for Pd(ii)–C homolytic cleavage even though it possesses conjugated dative ligands typically associated with easily accessible MLCT states (*i.e.*, [Pd d → diimine π*]) previously shown to facilitate free radical formation. The higher quantum efficiency in the T-shaped complexes that otherwise lack the classic ligand-based chromophores typically found in photoactive late transition metal complexes was a surprising finding. These data thus demonstrate a latent one-electron reaction manifold is readily accessible in T-shaped organo–Pd(ii) complexes – some of the most active organometallic species in modern Pd catalysis – using visible light as an external stimulus. The findings of this study may help to expand the synthetic toolbox of this central catalytic metal by shunting Pd away from its strong preference toward two-electron mechanisms.

## Data availability

Crystallographic data for 1b, 2, 4, 5a, 5b and S2 have been deposited at the CCDC under 2264687–2264692 and can be obtained from https://www.ccdc.cam.ac.uk/structures. Energies and Cartesian coordinates for computational datasets supporting this article have been uploaded as part of the ESI.†

## Author contributions

P. M. W. and B. P. C. conceived the idea for this work. P. M. W. and L. V. carried out experiments. B. P. C. provided direction for the scientific strategy. L. T. and G. D. S. designed and carried out transient absorption spectroscopic and computational analyses. A. R. S. performed coupled cluster calculations. P. M. W. and B. P. C. wrote the manuscript and ESI† with input from all authors.

## Conflicts of interest

The authors declare no competing interests.

## Supplementary Material

SC-014-D3SC02588H-s001

SC-014-D3SC02588H-s002

## References

[cit1] McConnell I., Li G. H., Brudvig G. W. (2010). Chem. Biol..

[cit2] Arias-Rotondo D. M., McCusker J. K. (2016). Chem. Soc. Rev..

[cit3] Narayanam J. M., Stephenson C. R. (2011). Chem. Soc. Rev..

[cit4] Prier C. K., Rankic D. A., MacMillan D. W. (2013). Chem. Rev..

[cit5] Dadashi-Silab S., Tasdelen M. A., Yagci Y. (2014). J. Polym. Sci., Part A: Polym. Chem..

[cit6] Twilton J., Le C., Zhang P., Shaw M. H., Evans R. W., MacMillan D. W. C. (2017). Nat. Rev. Chem.

[cit7] Zhou Q. Q., Zou Y. Q., Lu L. Q., Xiao W. J. (2019). Angew. Chem., Int. Ed..

[cit8] Troian-Gautier L., Turlington M. D., Wehlin S. A. M., Maurer A. B., Brady M. D., Swords W. B., Meyer G. J. (2019). Chem. Rev..

[cit9] Fleetham T., Li G., Li J. (2017). Adv. Mater..

[cit10] Crosby G. A., Perkins W. G., Klassen D. M. (2004). J. Chem. Phys..

[cit11] Anderson P. A., Richard Keene F., Meyer T. J., Moss J. A., Strouse G. F., Treadway J. A. (2002). J. Chem. Soc., Dalton Trans..

[cit12] Campagna S., Puntoriero F., Nastasi F., Bergamini G., Balzani V. (2007). Top. Curr. Chem..

[cit13] Vlcek A. (1998). Coord. Chem. Rev..

[cit14] McCusker J. K. (2003). Acc. Chem. Res..

[cit15] Wrighton M. (1974). Chem. Rev..

[cit16] Geoffroy G. L. (1983). J. Chem. Educ..

[cit17] Beach N. A., Gray H. B. (1968). J. Am. Chem. Soc..

[cit18] Graham M. A., Poliakoff M., Turner J. J. (1971). J. Chem. Soc. A.

[cit19] Stolz I. W., Sheline R. K., Dobson G. R. (1962). J. Am. Chem. Soc..

[cit20] Strohmeier W., Gerlach K. (1961). Chem. Ber..

[cit21] Wenger O. S. (2021). Chem. –Eur. J..

[cit22] Chan A. Y., Perry I. B., Bissonnette N. B., Buksh B. F., Edwards G. A., Frye L. I., Garry O. L., Lavagnino M. N., Li B. X., Liang Y. F., Mao E., Millet A., Oakley J. V., Reed N. L., Sakai H. A., Seath C. P., MacMillan D. W. C. (2022). Chem. Rev..

[cit23] Beil S. B., Chen T. Q., Intermaggio N. E., MacMillan D. W. C. (2022). Acc. Chem. Res..

[cit24] Kalyani D., McMurtrey K. B., Neufeldt S. R., Sanford M. S. (2011). J. Am. Chem. Soc..

[cit25] Neufeldt S. R., Sanford M. S. (2012). Adv. Synth. Catal..

[cit26] Zoller J., Fabry D. C., Ronge M. A., Rueping M. (2014). Angew. Chem., Int. Ed..

[cit27] Zhang H. H., Zhao J. J., Yu S. Y. (2020). ACS Catal..

[cit28] Zheng J., Nikbakht A., Breit B. (2021). ACS Catal..

[cit29] Zheng J., Tang N. N., Xie H., Breit B. (2022). Angew. Chem., Int. Ed..

[cit30] Song C. H., Zhang H. H., Yu S. Y. (2022). ACS Catal..

[cit31] Till N. A., Tian L., Dong Z., Scholes G. D., MacMillan D. W. C. (2020). J. Am. Chem. Soc..

[cit32] Welin E. R., Le C., Arias-Rotondo D. M., McCusker J. K., MacMillan D. W. C. (2017). Science.

[cit33] Heitz D. R., Tellis J. C., Molander G. A. (2016). J. Am. Chem. Soc..

[cit34] Tian L., Till N. A., Kudisch B., MacMillan D. W. C., Scholes G. D. (2020). J. Am. Chem. Soc..

[cit35] Kudisch M., Lim C. H., Thordarson P., Miyake G. M. (2019). J. Am. Chem. Soc..

[cit36] Zheng C., Cheng W. M., Li H. L., Na R. S., Shang R. (2018). Org. Lett..

[cit37] Shimomaki K., Murata K., Martin R., Iwasawa N. (2017). J. Am. Chem. Soc..

[cit38] Toriumi N., Shimomaki K., Caner J., Murata K., Martin R., Iwasawa N. (2021). Bull. Chem. Soc. Jpn..

[cit39] Chuentragool P., Kurandina D., Gevorgyan V. (2019). Angew. Chem., Int. Ed..

[cit40] Parasram M., Chuentragool P., Sarkar D., Gevorgyan V. (2016). J. Am. Chem. Soc..

[cit41] Ratushnyy M., Parasram M., Wang Y., Gevorgyan V. (2018). Angew. Chem., Int. Ed..

[cit42] Wang G. Z., Shang R., Cheng W. M., Fu Y. (2017). J. Am. Chem. Soc..

[cit43] Kurandina D., Parasram M., Gevorgyan V. (2017). Angew. Chem., Int. Ed..

[cit44] Wang G. Z., Shang R., Fu Y. (2018). Org. Lett..

[cit45] Koy M., Sandfort F., Tlahuext-Aca A., Quach L., Daniliuc C. G., Glorius F. (2018). Chem. –Eur. J..

[cit46] Kancherla R., Muralirajan K., Maity B., Zhu C., Krach P. E., Cavallo L., Rueping M. (2019). Angew. Chem., Int. Ed..

[cit47] Abdiaj I., Huck L., Mateo J. M., de la Hoz A., Gomez M. V., Diaz-Ortiz A., Alcazar J. (2018). Angew. Chem., Int. Ed..

[cit48] Zhou W. J., Cao G. M., Shen G., Zhu X. Y., Gui Y. Y., Ye J. H., Sun L., Liao L. L., Li J., Yu D. G. (2017). Angew. Chem., Int. Ed..

[cit49] Jiao Z. W., Lim L. H., Hirao H., Zhou J. S. (2018). Angew. Chem., Int. Ed..

[cit50] Wang G. Z., Shang R., Fu Y. (2018). Synthesis.

[cit51] Parasram M., Gevorgyan V. (2017). Chem. Soc. Rev..

[cit52] Sumino S., Fusano A., Fukuyama T., Ryu I. (2014). Acc. Chem. Res..

[cit53] Chuentragool P., Kurandina D., Gevorgyan V. (2019). Angew. Chem., Int. Ed..

[cit54] Mühlfenzl K. S., Sardana M., Skrydstrup T., Elmore C. S. (2022). ChemistrySelect.

[cit55] Zhao G., Yao W., Mauro J. N., Ngai M. Y. (2021). J. Am. Chem. Soc..

[cit56] Pak Shing Cheung K., Fang J., Mukherjee K., Mihranyan A., Gevorgyan V. (2022). Science.

[cit57] Marchese A. D., Durant A. G., Reid C. M., Jans C., Arora R., Lautens M. (2022). J. Am. Chem. Soc..

[cit58] Czyz M. L., Weragoda G. K., Horngren T. H., Connell T. U., Gomez D., O'Hair R. A. J., Polyzos A. (2020). Chem. Sci..

[cit59] Toriumi N., Inoue T., Iwasawa N. (2022). J. Am. Chem. Soc..

[cit60] Fafard C. M., Adhikari D., Foxman B. M., Mindiola D. J., Ozerov O. V. (2007). J. Am. Chem. Soc..

[cit61] Smoll K. A., Kaminsky W., Goldberg K. I. (2017). Organometallics.

[cit62] Burns C. T., Shen H., Jordan R. F. (2003). J. Organomet. Chem..

[cit63] Khusnutdinova J. R., Rath N. P., Mirica L. M. (2010). J. Am. Chem. Soc..

[cit64] Torres G. M., Liu Y., Arndtsen B. A. (2020). Science.

[cit65] Vanleeuwen P. W. N. M., Roobeek C. F., Huis R. (1977). J. Organomet. Chem..

[cit66] Liu Y., Zhou C., Jiang M., Arndtsen B. A. (2022). J. Am. Chem. Soc..

[cit67] Keyes A., Alhan H. E. B., Ha U., Liu Y. S., Smith S. K., Teets T. S., Beezer D. B., Harth E. (2018). Macromolecules.

[cit68] Keyes A., Dau H., Alhan H. E. B., Ha U., Ordonez E., Jones G. R., Liu Y. S., Tsogtgerel E., Loftin B., Wen Z. L., Wu J. I., Beezer D. B., Harth E. (2019). Polym. Chem..

[cit69] Dau H., Keyes A., Alhan H. E. B., Ordonez E., Tsogtgerel E., Gies A. P., Auyeung E., Zhou Z., Maity A., Das A., Powers D. C., Beezer D. B., Harth E. (2020). J. Am. Chem. Soc..

[cit70] Christmann U., Vilar R. (2005). Angew Chem. Int. Ed. Engl..

[cit71] Barrios-Landeros F., Carrow B. P., Hartwig J. F. (2009). J. Am. Chem. Soc..

[cit72] Littke A. F., Dai C. Y., Fu G. C. (2000). J. Am. Chem. Soc..

[cit73] Surawatanawong P., Hall M. B. (2008). Organometallics.

[cit74] Fleckenstein C. A., Plenio H. (2010). Chem. Soc. Rev..

[cit75] Hartwig J. F. (2007). Inorg. Chem..

[cit76] Yamashita M., Hartwig J. F. (2004). J. Am. Chem. Soc..

[cit77] Shaughnessy H. K. (2020). Curr. Org. Chem..

[cit78] Fu G. C. (2008). Acc. Chem. Res..

[cit79] Christmann U., Vilar R. (2005). Angew. Chem., Int. Ed..

[cit80] Fleckenstein C. A., Plenio H. (2010). Chem. Soc. Rev..

[cit81] Dubbaka S. R. (2005). Synlett.

[cit82] Shaughnessy K. H. (2020). Isr. J. Chem..

[cit83] Heravi M. M., Kheilkordi Z., Zadsirjan V., Heydari M., Malmir M. (2018). J. Organomet. Chem..

[cit84] Dorel R., Grugel C. P., Haydl A. M. (2019). Angew. Chem., Int. Ed..

[cit85] Seifinoferest B., Tanbakouchian A., Larijani B., Mahdavi M. (2021). Asian J. Org. Chem..

[cit86] Chen L. Y., Ren P., Carrow B. P. (2016). J. Am. Chem. Soc..

[cit87] Chen L., Francis H., Carrow B. P. (2018). ACS Catal..

[cit88] Lau S. H., Yu P., Chen L., Madsen-Duggan C. B., Williams M. J., Carrow B. P. (2020). J. Am. Chem. Soc..

[cit89] Zhao S., Gensch T., Murray B., Niemeyer Z. L., Sigman M. S., Biscoe M. R. (2018). Science.

[cit90] Kocen A. L., Brookhart M., Daugulis O. (2019). Nat. Commun..

[cit91] Kubota K., Takahashi R., Uesugi M., Ito H. (2020). ACS Sustain. Chem. Eng..

[cit92] Takahashi R., Seo T., Kubota K., Ito H. (2021). ACS Catal..

[cit93] Yamashita M., Takamiya I., Jin K., Nozaki K. (2006). Organometallics.

[cit94] Kim D.-G., Bell A., Register R. A. (2015). ACS Macro Lett..

[cit95] Bermeshev M. V., Chapala P. P. (2018). Prog. Polym. Sci..

[cit96] Yamashita M., Takamiya I., Jin K., Nozaki K. (2006). Organometallics.

[cit97] Khusnutdinova J. R., Rath N. P., Mirica L. M. (2010). J. Am. Chem. Soc..

[cit98] Siegbahn P. E. M. (1995). J. Phys. Chem..

[cit99] Evans M. E., Li T., Vetter A. J., Rieth R. D., Jones W. D. (2009). J. Org. Chem..

[cit100] Wick D. D., Jones W. D. (1999). Organometallics.

[cit101] Vilar R., Mingos D. M. P., Cardin C. J. (1996). J. Chem. Soc., Dalton Trans..

[cit102] Burrows A. D., Mingos D. M. P., Menzer S., Vilar R., Williams D. J. (1995). J. Chem. Soc., Dalton Trans..

[cit103] Durà-Vilà V., Mingos D. M. P., Vilar R., White A. J. P., Williams D. J. (2000). J. Organomet. Chem..

[cit104] Okunaka M., Matsubayashi G., Tanaka T. (1977). Bull. Chem. Soc. Jpn..

[cit105] Tomson N. C., Labios L. A., Weyhermuller T., Figueroa J. S., Wieghardt K. (2011). Inorg. Chem..

[cit106] Barnett B. R., Labios L. A., Moore C. E., England J., Rheingold A. L., Wieghardt K., Figueroa J. S. (2015). Inorg. Chem..

[cit107] Bonney K. J., Proutiere F., Schoenebeck F. (2013). Chem. Sci..

[cit108] Kalvet I., Bonney K. J., Schoenebeck F. (2014). J. Org. Chem..

[cit109] Proutiere F., Aufiero M., Schoenebeck F. (2012). J. Am. Chem. Soc..

[cit110] Aufiero M., Proutiere F., Schoenebeck F. (2012). Angew. Chem., Int. Ed..

[cit111] Seechurn C. C. C. J., Sperger T., Scrase T. G., Schoenebeck F., Colacot T. J. (2017). J. Am. Chem. Soc..

[cit112] Fricke C., Sperger T., Mendel M., Schoenebeck F. (2021). Angew. Chem., Int. Ed..

[cit113] Gabrielsson A., Blanco-Rodriguez A. M., Matousek P., Towrie M., Vlcek A. (2006). Organometallics.

[cit114] Vlcek A. (2000). Coord. Chem. Rev..

[cit115] Virrels I. G., George M. W., Turner J. J., Peters J., Vlcek A. (1996). Organometallics.

[cit116] Kleverlaan C. J., Stufkens D. J., Clark I. P., George M. W., Turner J. J., Martino D. M., van Willigen H., Vlcek A. (1998). J. Am. Chem. Soc..

[cit117] Vichova J., Hartl F., Vlcek A. (1992). J. Am. Chem. Soc..

[cit118] Ji Y. N., DiRocco D. A., Hong C. M., Wismer M. K., Reibarkh M. (2018). Org. Lett..

[cit119] Wilcox D. A., Snaider J., Mukherjee S., Yuan L., Huang L. B., Savoie B. M., Boudouris B. W. (2019). Soft Matter.

[cit120] Shields B. J., Kudisch B., Scholes G. D., Doyle A. G. (2018). J. Am. Chem. Soc..

[cit121] For the simulated UV-vis spectrum of 3, see the ESI†

[cit122] Ting S. I., Garakyaraghi S., Taliaferro C. M., Shields B. J., Scholes G. D., Castellano F. N., Doyle A. G. (2020). J. Am. Chem. Soc..

[cit123] Chow P.-K., Cheng G., Tong G. S. M., Ma C., Kwok W.-M., Ang W.-H., Chung C. Y.-S., Yang C., Wang F., Che C.-M. (2016). Chem. Sci..

[cit124] Mancilha F. S., Barloy L., Rodembusch F. S., Dupont J., Pfeffer M. (2011). Dalton Trans..

[cit125] Lai S.-W., Cheung T.-C., Chan M. C. W., Cheung K.-K., Peng S.-M., Che C.-M. (2000). Inorg. Chem..

[cit126] Craig C. A., Watts R. J. (1989). Inorg. Chem..

[cit127] von der Stück R., Krause M., Brünink D., Buss S., Doltsinis N. L., Strassert C. A., Klein A. (2022). Z. Anorg. Allg. Chem..

[cit128] Davis T. L., Watts J. L., Brown K. J., Hewage J. S., Treleven A. R., Lindeman S. V., Gardinier J. R. (2015). Dalton Trans..

[cit129] Yang L., Powell D. R., Houser R. P. (2007). Dalton Trans..

[cit130] Gozem S., Melaccio F., Valentini A., Filatov M., Huix-Rotllant M., Ferre N., Frutos L. M., Angeli C., Krylov A. I., Granovsky A. A., Lindh R., Olivucci M. (2014). J. Chem. Theory Comput..

[cit131] Levine B. G., Ko C., Quenneville J., Martinez T. J. (2006). Mol. Phys..

[cit132] Mandich M. L., Halle L. F., Beauchamp J. L. (1984). J. Am. Chem. Soc..

[cit133] It was still the case that no signals consistent with luminescent emission were observed even when higher beam power and greater concentrations were used

[cit134] Kjaer K. S. (2018). et al.. Phys. Chem. Chem. Phys..

[cit135] Kitamura N., Sakuda E. (2005). J. Phys. Chem. A.

[cit136] Caspar J. V., Meyer T. J. (1983). J. Am. Chem. Soc..

[cit137] Cagan D. A., Bím D., Silva B., Kazmierczak N. P., McNicholas B. J., Hadt R. G. (2022). J. Am. Chem. Soc..

