# Supplementary material for: Visible light-induced palladium–carbon bond weakening in catalytically relevant T-shaped complexes
Source: Chem Sci. 2023 Nov 28;14(48):14217–28. doi: 10.1039/d3sc02588h (PMC10717500; doi:10.1039/d3sc02588h)
Supplement: SC-014-D3SC02588H-s001 [file SC-014-D3SC02588H-s001.pdf]

Electronic Supplementary Material (ESI) for Chemical Science.
This journal is © The Royal Society of Chemistry 2023

Supporting Information

Visible Light-Induced Bond Weakening in Catalytically Relevant T-Shaped Organopalladium(II) Complexes

Peter M. Waddell[1], Lei Tian[1], Anthony R. Scavuzzo,[1] Lalu Venigalla,[2] Gregory D. Scholes[1] and Brad P. Carrow[2]*

[1]Department of Chemistry, Princeton University, Princeton, NJ 08544, United States

[2]Department of Chemistry, University of Houston, Houston, Texas 77204, United States

*E-mail: bcarrow@central.uh.edu

General remarks......S2
Preparation and characterization data of compounds S3
NMR spectra of compounds......S7
Procedures for observing light reactivity S27
Quantum yield determination details......S72
DFT calculations......S127
UV-vis spectroscopy......S161
Fluorescence spectroscopy S168
Transient absorption spectroscopy S173
X-ray crystallographic data S179
References S188

General remarks

All manipulations were conducted in a dry nitrogen filled dry box or using standard Schlenk techniques unless otherwise specified. All non-deuterated solvents were purchased from Aldrich or Fisher and purified in a solvent purification system by percolation through neutral alumina under positive pressure of nitrogen. $CDCl_3$ and C_6D_6 were ordered from Cambridge Isotope Laboratories, Inc. and dried over CaH_2, then distilled and degassed by at least three freeze-pump-thaw cycles. CD_2Cl_2 was ordered from Cambridge Isotope Laboratories, Inc. in ampoules which were broken in the glovebox and subsequently used. Tri-*tert*-butylphosphine, tetrabutylphosphonium tetrafluoroborate, (2,2,6,6-tetramethylpiperidin-1-yl)oxyl (TEMPO), cyclohexane, 1,3,5-tris(trifluoromethyl)benzene, 1,4-dioxane, tetramethyltin, 2,4-dinitrobenzaldehyde, and 1,4-cyclohexadiene were commercially obtained and used as received. (COD)$PdCl_2$,[1] (COD)$PdBr_2$,[2] (COD)Pd(CH_3)Cl,[3, 4] tri-1-adamantylphosphine,[5] **1b**,[6] **3**,[3] **6**,[7] **7**,[8] (COD)bis(trimethylsilylmethyl)Pd(II)[9], and $[t\text{-}Bu_3PH]^+[Cl]^-$[10], were prepared according to literature procedures. ^1H and ^{13}C, and ^{31}P nuclear magnetic resonance spectra (NMR) were obtained on a Bruker 300 MHz or 500 MHz recorded in ppm (δ). ^1H spectra were referenced against residual $CHCl_3$, $CHDCl_2$, etc. Signal splitting patterns were described as singlet (s), doublet (d), triplet (t), quartet (q), quintet (quint), broad (br) or multiplet (m), with coupling constants (*J*) in Hz.

UV-vis spectroscopy information -- see page S161
Fluorescence spectroscopy information – see page S168
Transient absorption spectroscopy information – see page S173
X-ray crystallography information – see page S179

Preparation and characterization data of compounds

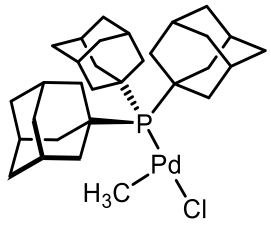

2. This compound was prepared analogously to **1a**.[6] DCM (5 mL) was added to a mixture of tri-1-adamantylphosphine (50 mg, 0.115 mmol) and (COD)Pd(CH_3)Cl (30 mg, 0.115 mmol) in a 4-dram vial and a stir bar was added. The reaction mixture turned yellow and was stirred at room temperature for 16 hours, then the reaction mixture was filtered through Celite and the filter was washed with additional DCM until the yellow color was fully washed through. The filtrate was concentrated in vacuo, then diethyl ether was added to crash out the solid product. The supernatant was decanted and the solids were washed with ether. After recrystallization from DCM/pentane, **2** was obtained as yellow crystals (41 mg, 60%).

^{1}H NMR (300 MHz, $CDCl_3$) δ_H 2.40 (br, 18H), 2.07 (br, 9H), 1.88-1.66 (m, 18H), 1.74 (s, 3H).

^{13}C{^{1}H} NMR (125 MHz, $CDCl_3$) δ_C 47.84 (J = 7.7 Hz), 42.17 (br), 36.52, 29.09 (J = 7.7 Hz), -2.81.

^{31}P{^{1}H} NMR (121 MHz, $CDCl_3$) δ_P 53.03 (s).

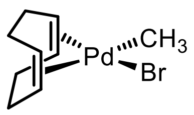

S1. This compound was prepared in an analogous manner to (COD)Pd(CH_3)Cl.[3] In a 4-dram vial, a slurry of (COD)PdBr_2 (118 mg, 0.315 mmol) was prepared in DCM (6 mL) and $SnMe_4$ (68 mg, 0.378 mmol) was added. The reaction mixture was stirred at room temperature for 48 hours. The reaction mixture was filtered through Celite and washed with DCM, resulting in a clear yellow filtrate. The filtrate was reduced in vacuo. The resulting material was washed with hexanes until ^{1}H NMR indicated that no tin compounds were present. The product was thus obtained as bright orange solids and carried forward without additional purification (53 mg, 54%).

^{1}H NMR (500 MHz, $CDCl_3$) δ_H 5.92 (m, 2H), 5.23 (m, 2H), 2.71-2.40 (m, 8H), 1.20 (s, 3H).

^{13}C{^{1}H} NMR (125 MHz, $CDCl_3$) δ_C 123.36, 103.18, 31.09, 28.01, 10.32.

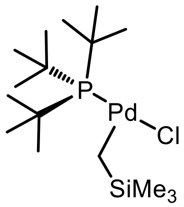

4. A solution of (COD)bis(trimethylsilylmethyl)Pd(II) (0.845 g, 2.17 mmol) in DCM was prepared. To this, a solution of $[t\text{-}Bu_3PH]^+[Cl]^-$ (0.519 mg, 2.17 mmol) in DCM was added at room temperature. After stirring 3 hours, the reaction mixture was filtered through Celite and the filtrate was reduced in vacuo. The resulting material was purified by silica gel chromatography (DCM). Bright yellow fractions were recovered, which were concentrated *in vacuo* to obtain **4** as a yellow-orange crystalline solid material (123 mg, 13%). A concentrated DCM solution of the recovered product was prepared, to which a large volume of pentane was added. The supernatant that resulted was yellow. This supernatant was decanted and cooled to -35 ^{o}C, which allowed X-ray quality crystals to be obtained.

^{1}H NMR (500 MHz, $CDCl_3$) δ_H 2.08 (m, 2H), 1.53 (m, 27 H), 0.26 (s, 9H).

$^{13}C\{^{1}H\}$ NMR (125 MHz, $CDCl_3$) δ_C 39.84 (d, J = 11.2 Hz), 32.16 (d, J = 3.0 Hz), 11.04 (d, J = 2.2 Hz), 2.49.

$^{31}P\{^{1}H\}$ NMR (121 MHz, $CDCl_3$) δ_P 71.05 (s).

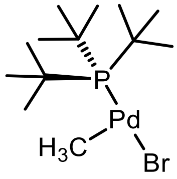

1b. This compound was prepared analogously to **1a**.[6] DCM (5 mL) was added to a mixture of tri-*tert*-butylphosphine (14 mg, 0.068 mmol) and (COD)Pd(CH_3)Br (21 mg, 0.068 mmol) in a 4-dram vial and a stir bar was added. The reaction mixture turned yellow and was stirred at room temperature for 3 hours, then the reaction mixture was filtered through Celite and the filter was washed with additional DCM until the yellow color was fully washed through. The filtrate was concentrated in vacuo, then pentane was added to crash out the solid product. The supernatant was

decanted and the solids were washed with pentane. After recrystallization from DCM/pentane, **1b** was obtained as yellow crystals (11 mg, 40%).

^{1}H NMR (500 MHz, $CDCl_3$) δ_H 1.83 (s, 3H), 1.50 (m, 27H).

^{13}C{^{1}H} NMR (125 MHz, $CDCl_3$) δ_C 40.19 (d, J = 10.9 Hz), 31.99 (d, J = 3.0 Hz), -2.09.

^{31}P{^{1}H} NMR (121 MHz, $CDCl_3$) δ_P 68.35 (s).

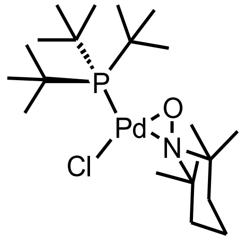

5a. A solution of **1a** (11 mg, 0.031 mmol) and 7 equiv. of TEMPO (32 mg, 0.205 mmol) in $CDCl_3$ in a NMR tube, which was sealed with electrical tape and exported from the glovebox. This reaction mixture was put under blue LED irradiation until complete consumption of **1b** was observed by ^{31}P NMR. The reaction mixture was then opened to air and the volatiles were removed in vacuo. Purification using silica gel chromatography (3% EtOAc in hexanes) yielded the product as a yellow-orange solid (13 mg, 85%). X-ray quality crystals were grown by slow evaporation of a concentrated pentane solution of this compound.

^{1}H NMR (500 MHz, $CDCl_3$) δ_H 2.08 (s, 6H), 1.78-1.55 (m, 6H), 1.57-1.45 (m, 27H), 1.35 (s, 6H).

^{13}C{^{1}H} NMR (125 MHz, $CDCl_3$) δ_C 67.64 (d, J = 1.2 Hz), 39.34 (d, J = 7.7 Hz), 37.72, 32.31 (d, J = 4.0 Hz), 31.82, 25.29 (d, J = 5.0 Hz), 16.93.

^{31}P{^{1}H} NMR (121 MHz, $CDCl_3$) δ_P 88.74 (s).

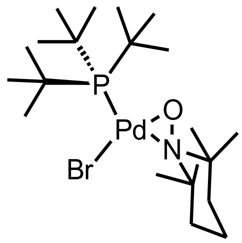

5b. This compound was independently prepared as follows: (COD)$PdBr_2$ (66 mg, 0.176 mmol) and tri-*tert*-butylphoshpine (53 mg, 0.264 mmol) were mixed in a 100 mL roundbottom flask and toluene (25 mL) was added. The reaction mixture was stirred at room temperature for 16 h, during

which time it turned very dark green, indicating the formation of **6**. To this mixture was added a solution of TEMPO (28 mg, 0.176 mmol) in toluene. The reaction mixture was stirred at room temperature for 4 h, during which time it turned orange-red, indicating formation of the product. The reaction mixture was filtered through Celite and the filtrate was reduced in vacuo, resulting in red solids (93 mg, 97%). X-ray quality crystals were grown by slow evaporation of a concentrated pentane solution of this compound.

^{1}H NMR (500 MHz, $CDCl_3$) δ_H 2.06 (s, 6H), 1.86-1.58 (m, 6H), 1.57-1.47 (m, 27 H), 1.36 (s, 6H).

^{13}C{^{1}H} NMR (125 MHz, $CDCl_3$) δ_C 68.18 (d, J = 1.2 Hz), 39.50 (d, J = 7.6 Hz), 37.63, 32.56 (s, J = 3.7 Hz), 31.86, 25.42 (d, J = 5.1 Hz), 16.97.

^{31}P{^{1}H} NMR (121 MHz, $CDCl_3$) δ_P 90.01 (s).

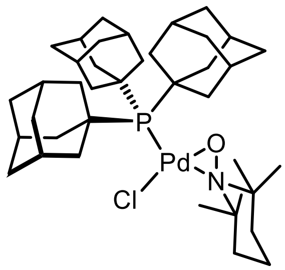

S2. A solution of **2** (11 mg, 0.019 mmol) and 7 equiv. of TEMPO (19 mg, 0.124 mmol) in $CDCl_3$ in a NMR tube, which was sealed with electrical tape and exported from the glovebox. This reaction mixture was put under blue LED irradiation until complete consumption of **2** was observed by ^{31}P NMR. The reaction mixture was then opened to air and the volatiles were removed in vacuo. Purification using silica gel chromatography (3% EtOAc in hexanes) yielded the product as a yellow-orange solid (13 mg, 95%). X-ray quality crystals were grown by slow evaporation of a concentrated pentane solution of this compound.

^{1}H NMR (500 MHz, $CDCl_3$) δ_H 2.51 (br, 18H), 2.15 (s, 6H), 1.96 (br, 9H), 1.90-1.44 (m, 24H), 1.36 (s, 6H).

^{13}C{^{1}H} NMR (125 MHz, $CDCl_3$) δ_C 67.69 (d, J = 1.2 Hz), 46.77 (d, J = 3.5 Hz), 42.03 (br), 37.69, 36.86 (br), 31.91, 29.83, 29.42 (d, J = 7.8 Hz), 25.40 (d, J = 4.6 Hz), 16.99.

^{31}P{^{1}H} NMR (121 MHz, $CDCl_3$) δ_P 77.15 (s).

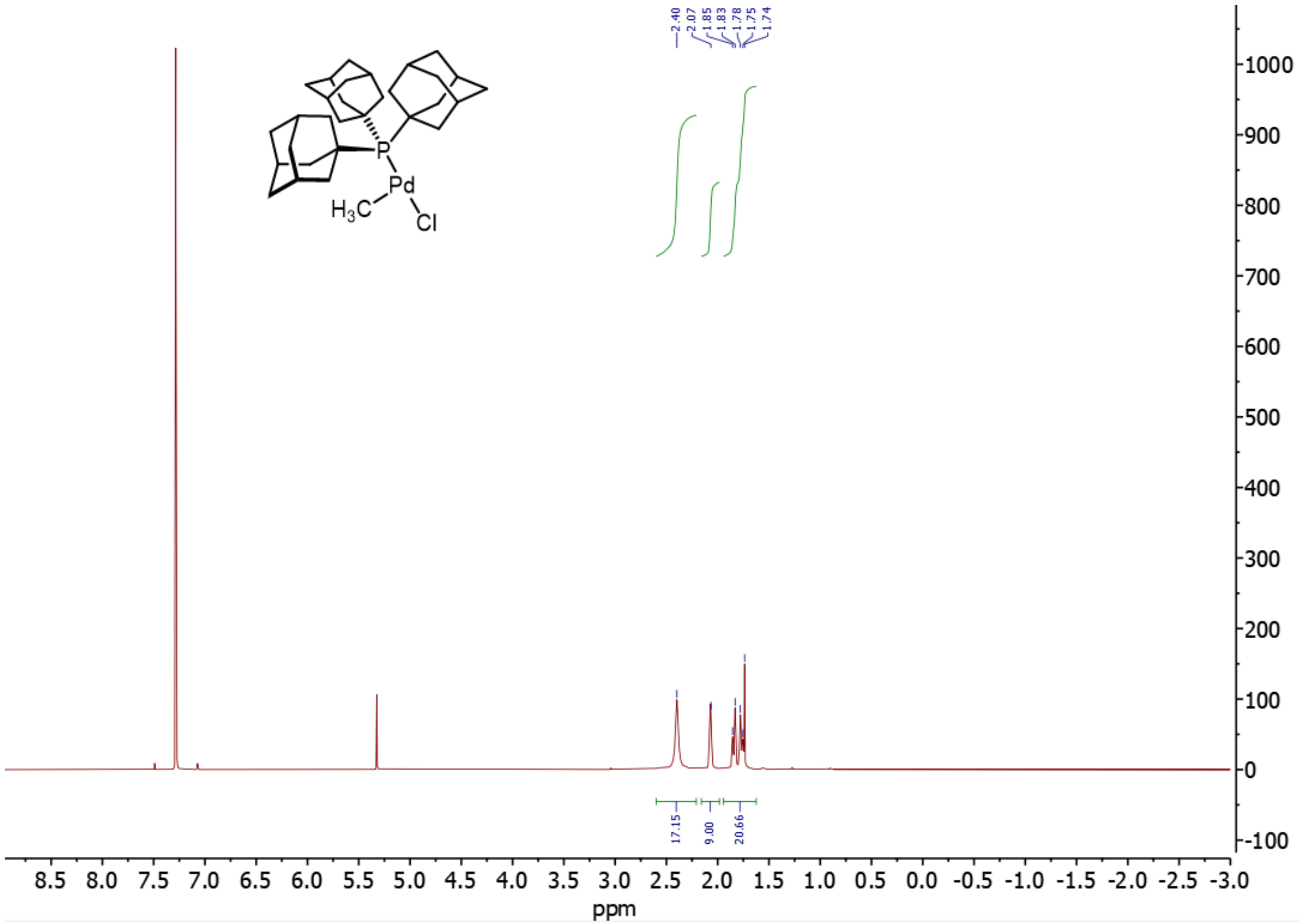

Figure S1. ^{1}H NMR spectrum ($CDCl_3$, 300 MHz) of **2**.

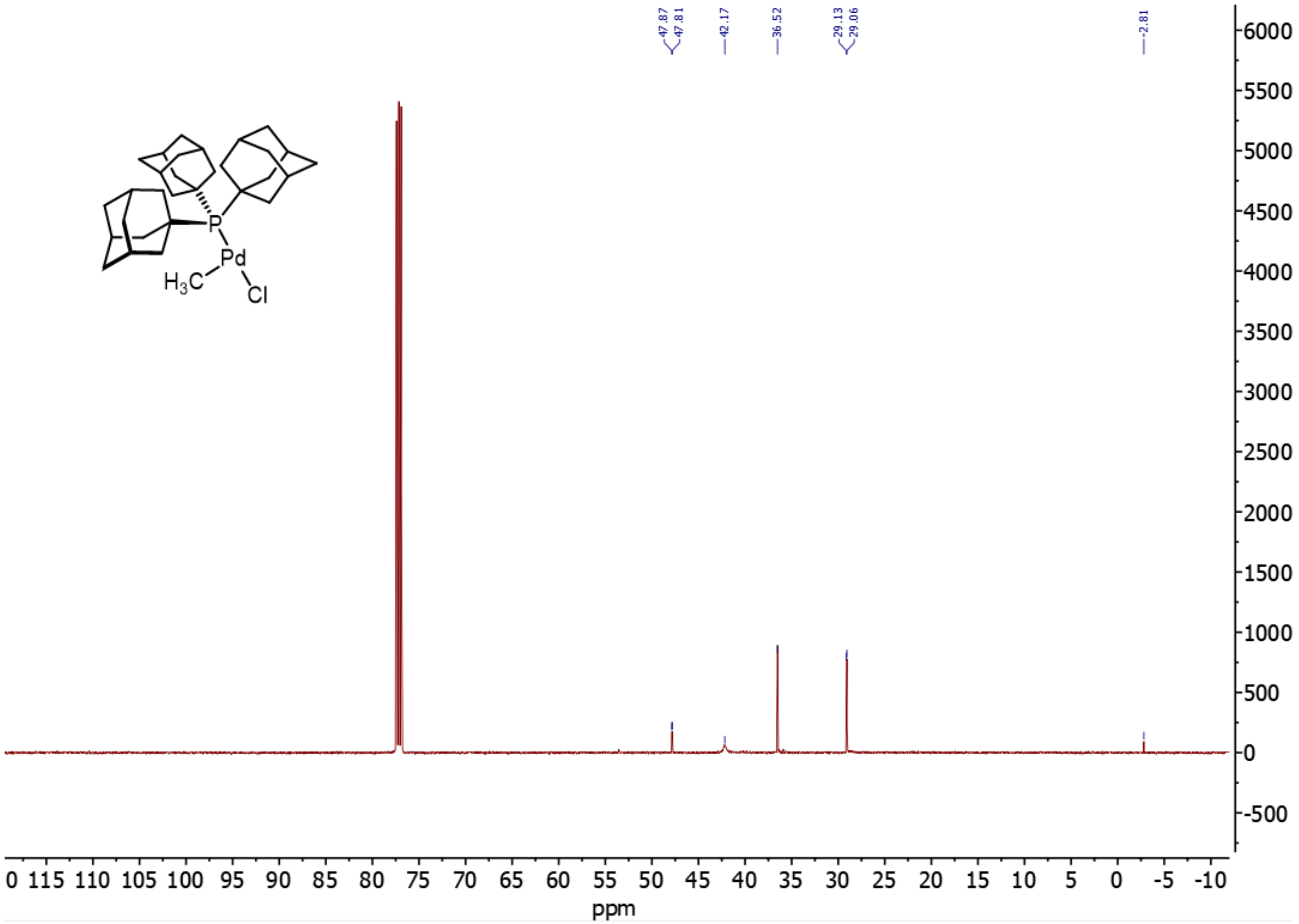

Figure S2. ^{13}C NMR spectrum ($CDCl_3$, 125 MHz) of **2**.

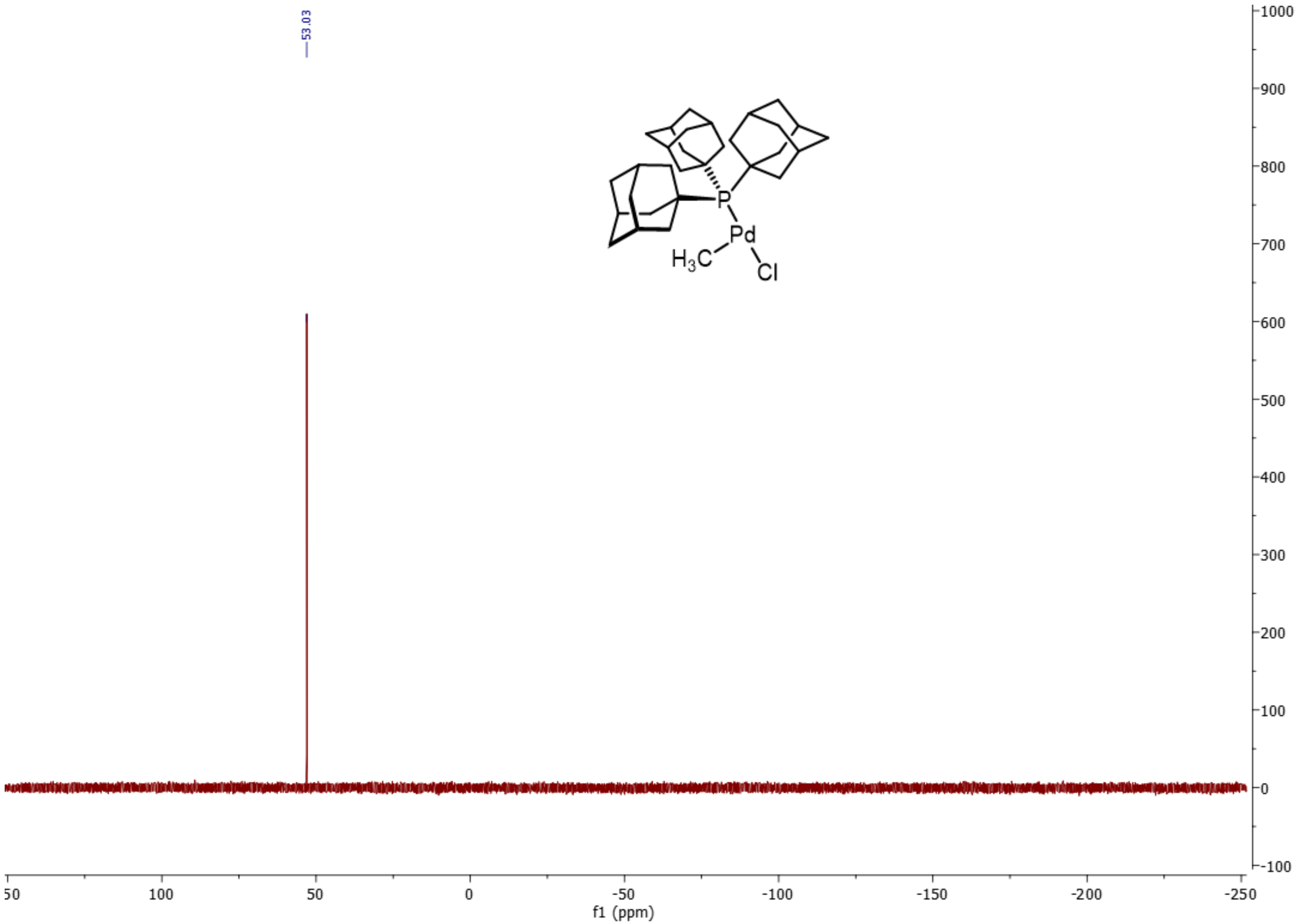

Figure S3. ^{31}P NMR spectrum ($CDCl_3$, 121 MHz) of **2**.

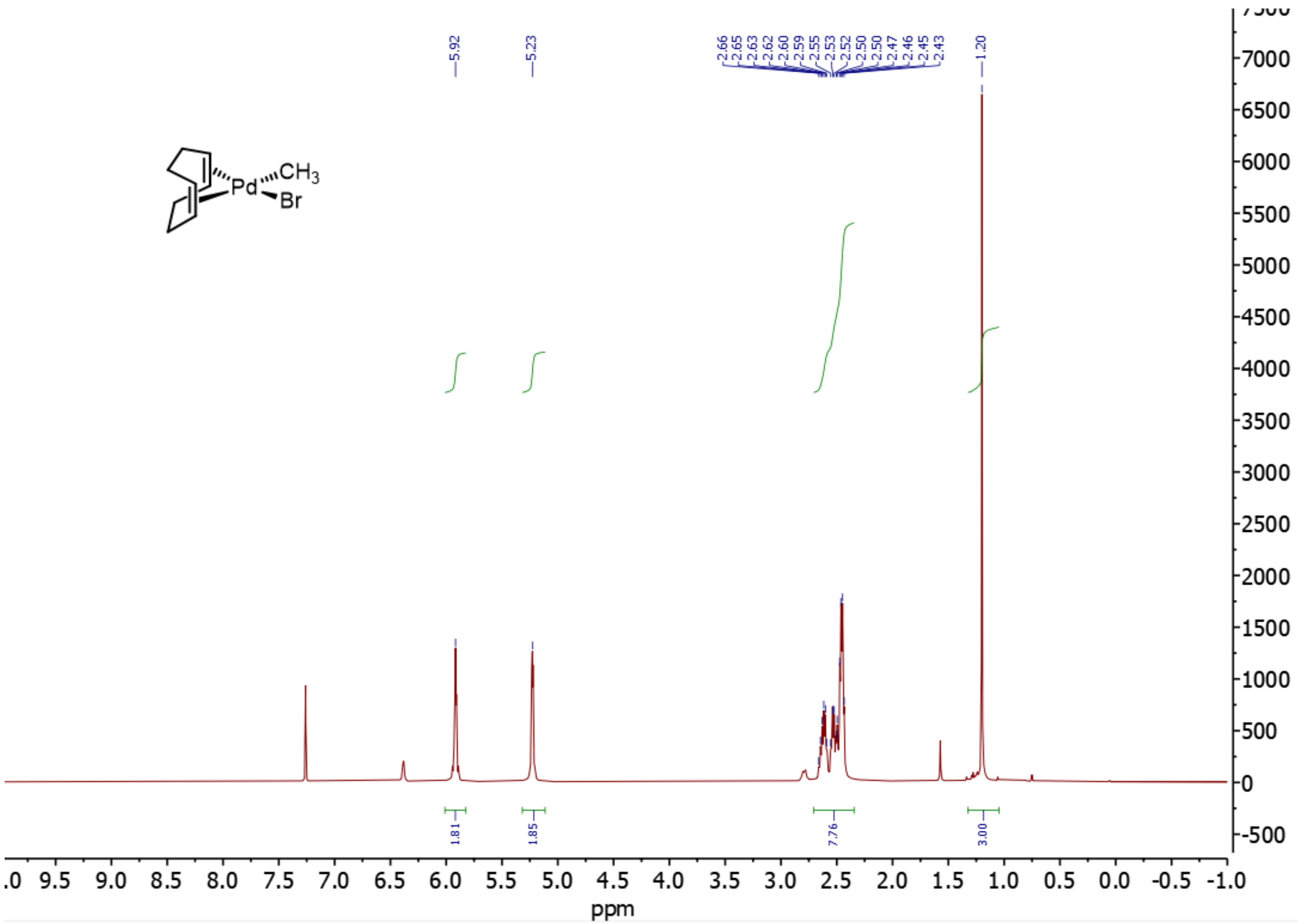

Figure S4. ^{1}H NMR spectrum ($CDCl_3$, 300 MHz) of **S1**.

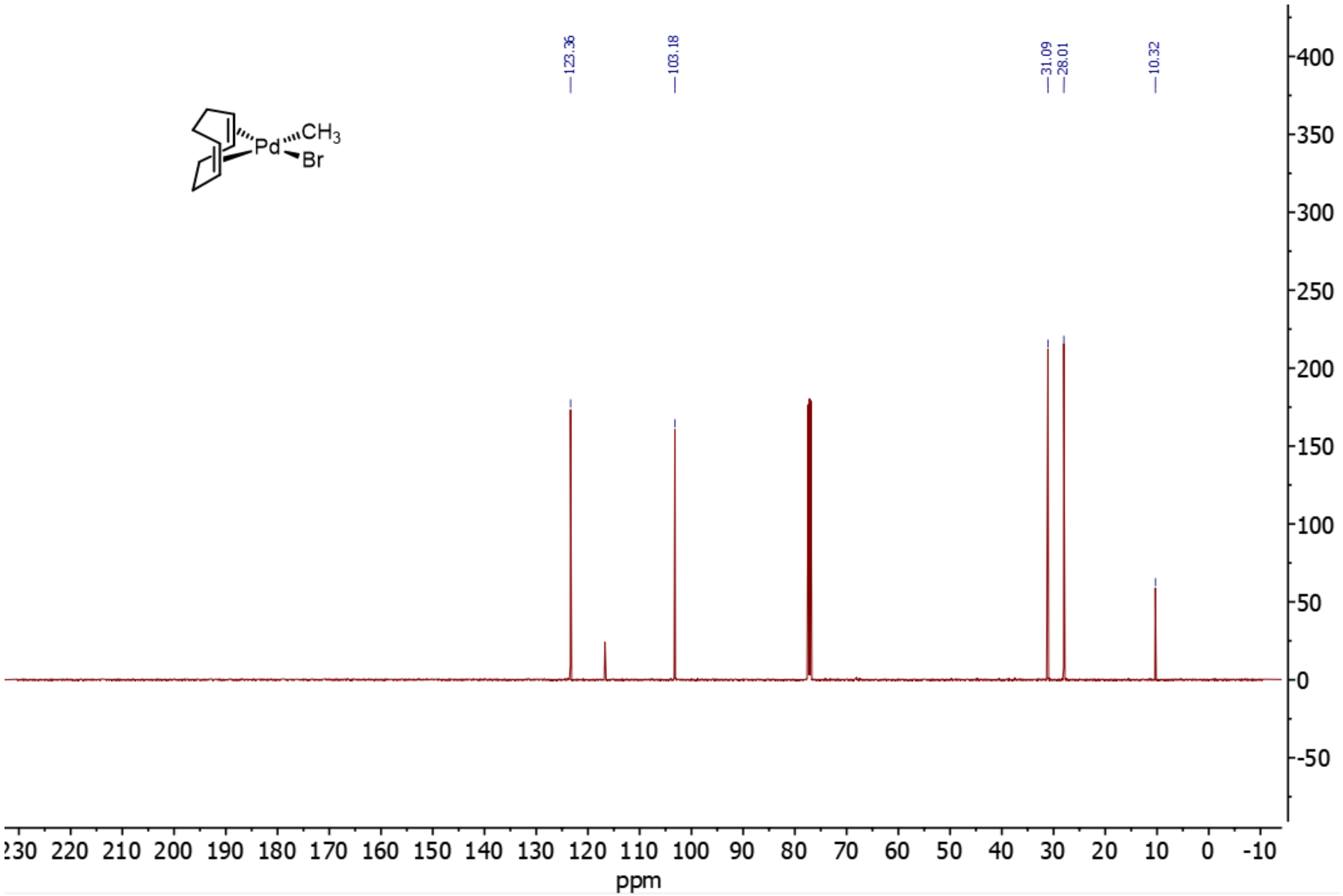

Figure S5. ^{13}C NMR spectrum ($CDCl_3$, 125 MHz) of **S1**.

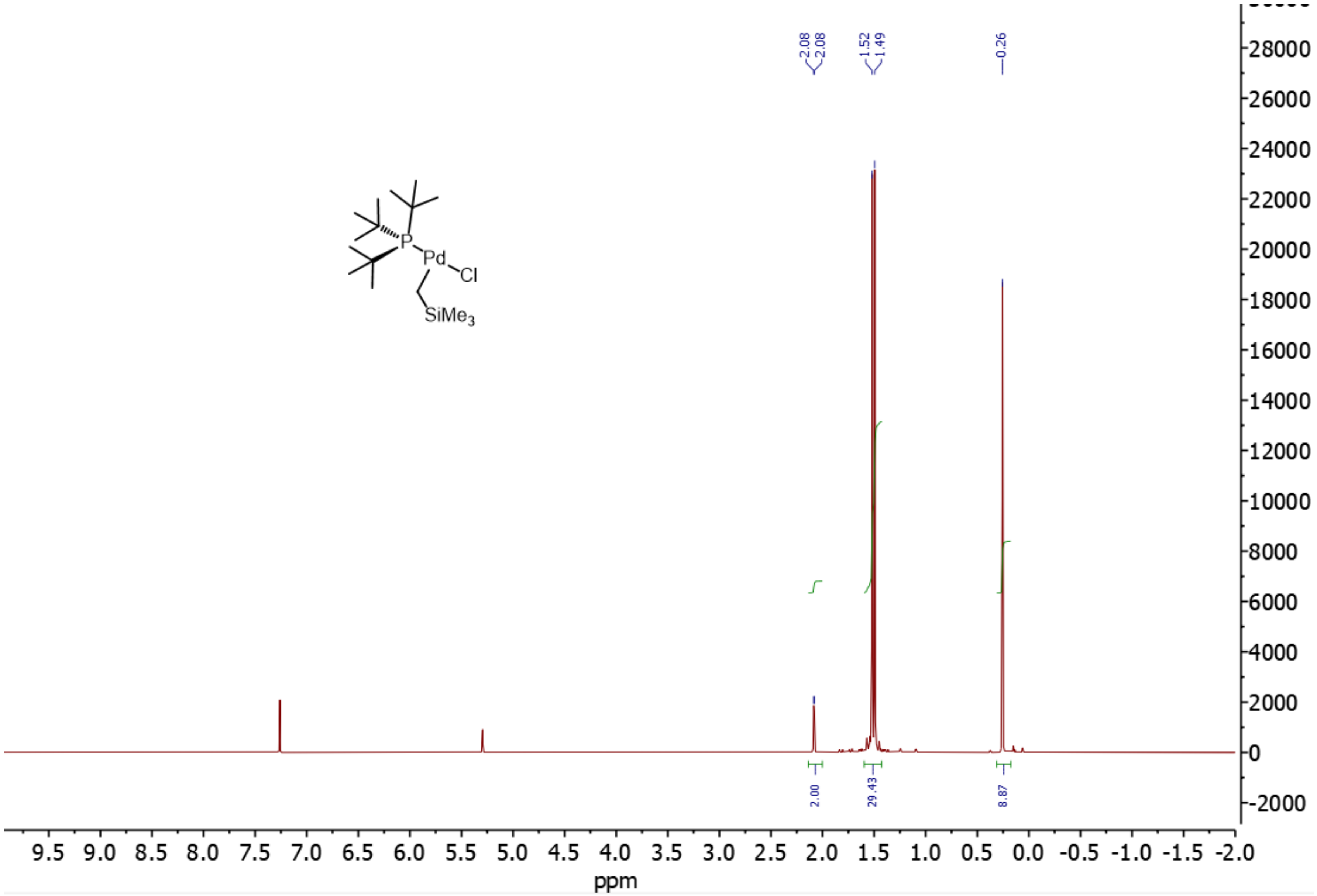

Figure S6. ^{1}H NMR spectrum ($CDCl_3$, 500 MHz) of **4**.

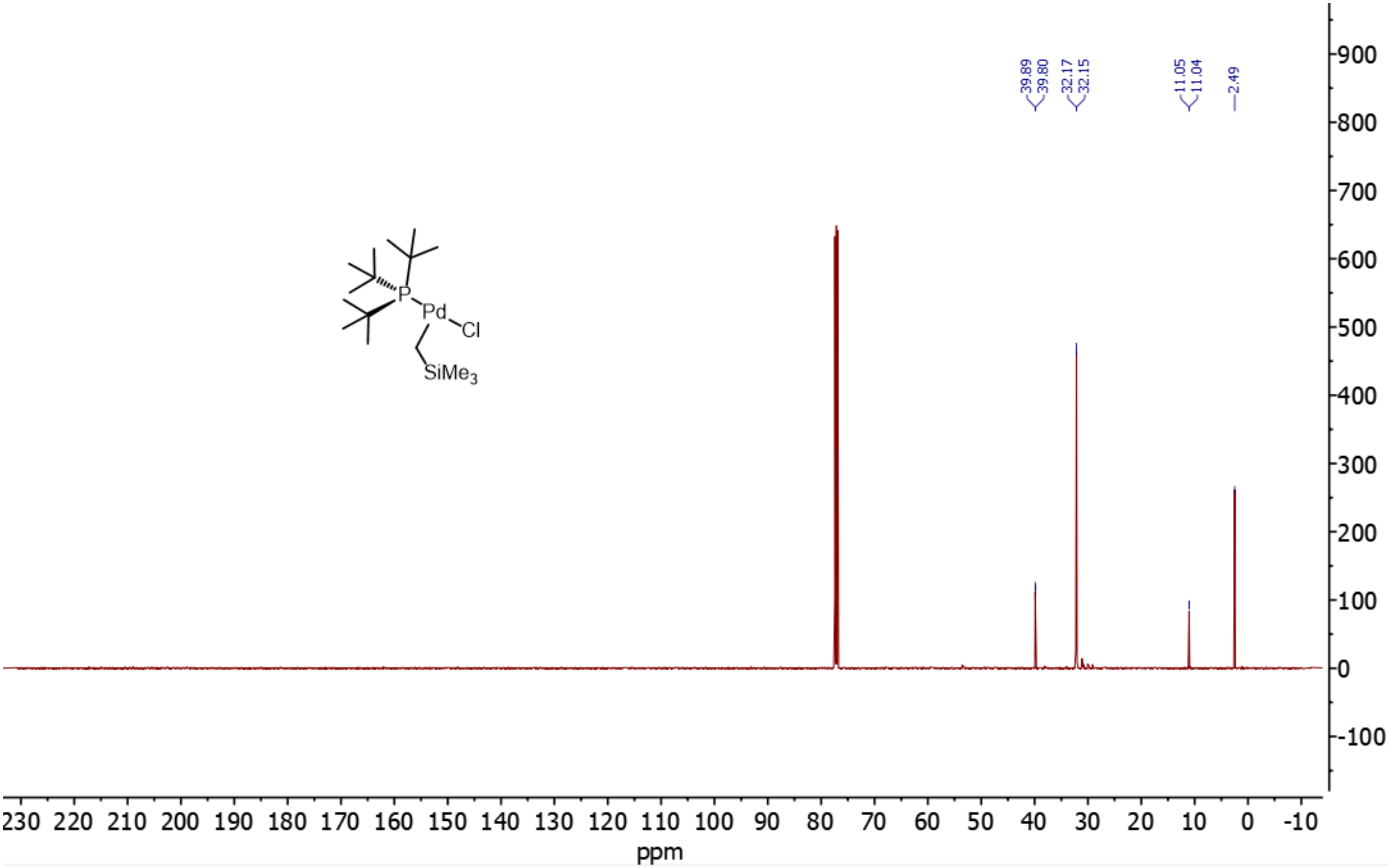

Figure S7. ^{13}C NMR spectrum ($CDCl_3$, 125 MHz) of **4**.

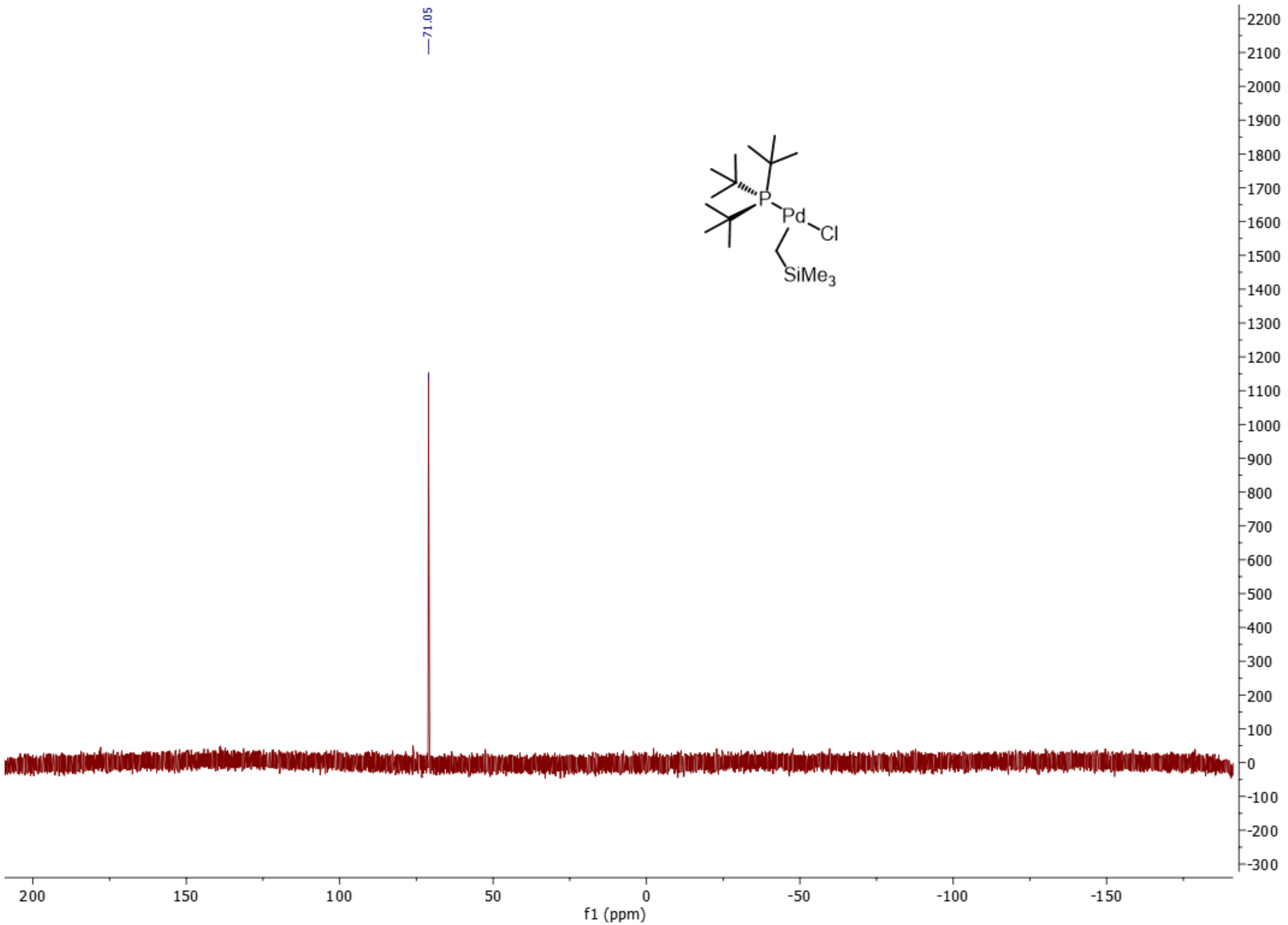

Figure S8. ^{31}P NMR spectrum ($CDCl_3$, 121 MHz) of **4**.

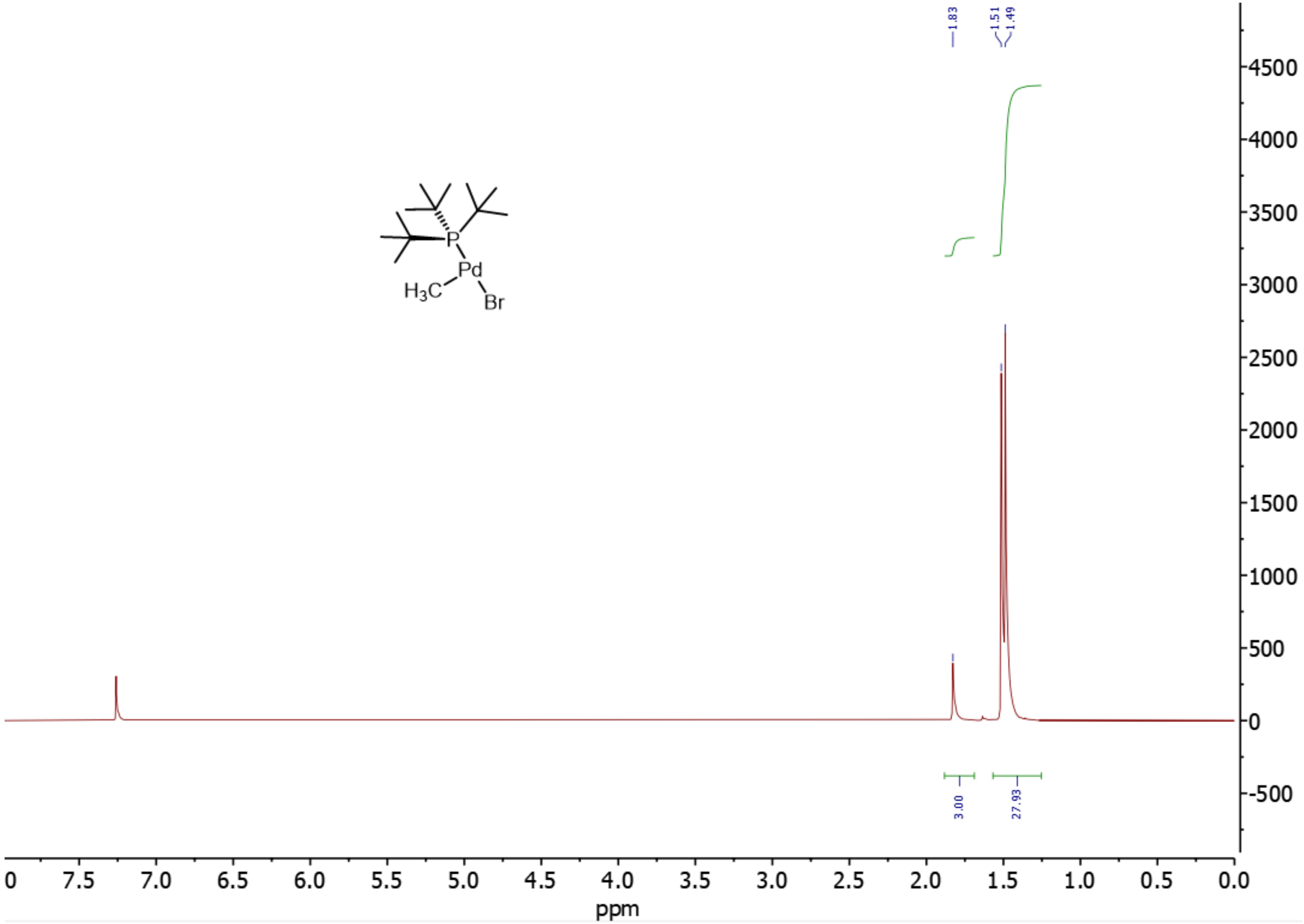

Figure S9. ^{1}H NMR spectrum ($CDCl_3$, 500 MHz) of **1b**.

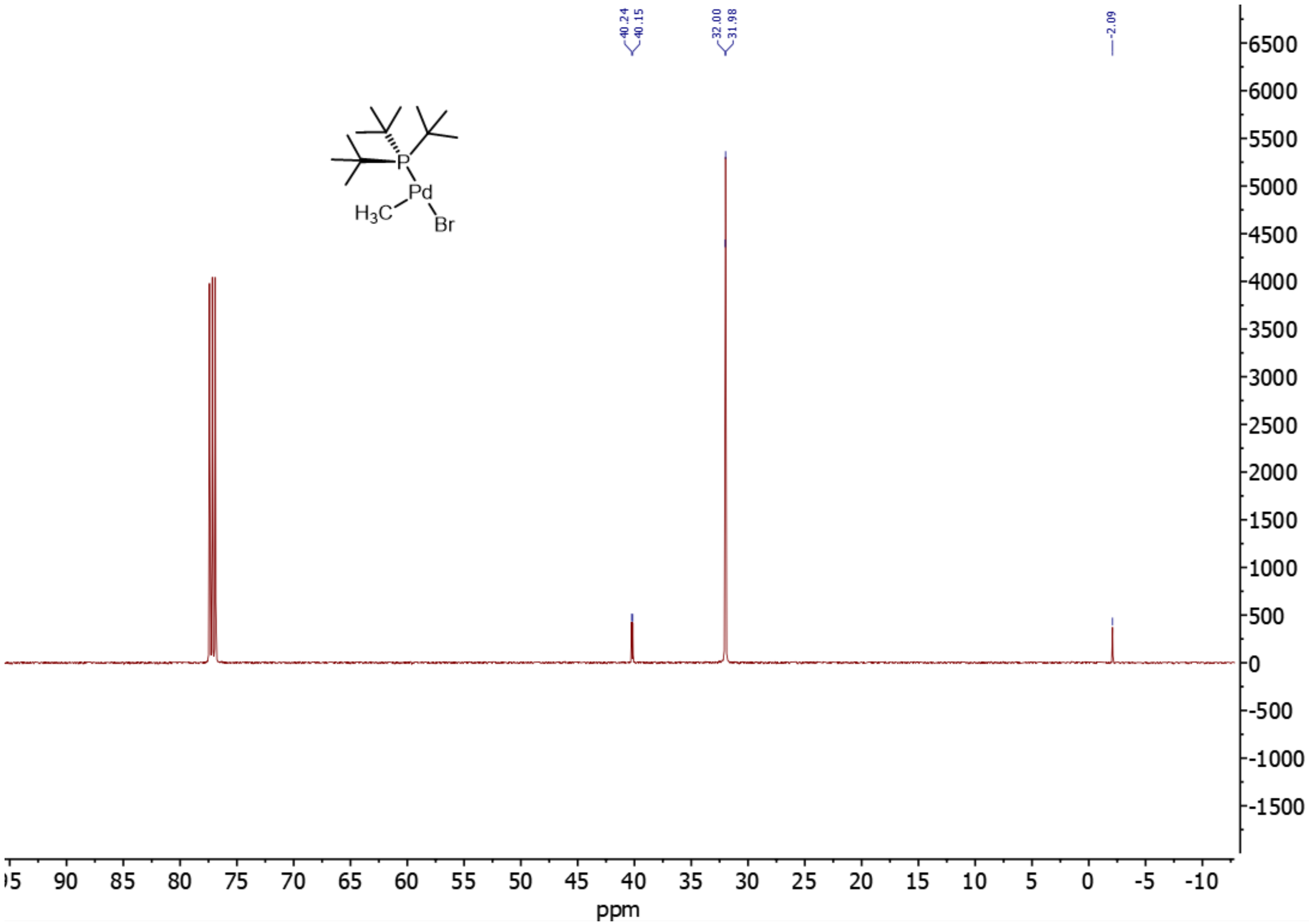

Figure S10. ^{13}C NMR spectrum ($CDCl_3$, 125 MHz) of **1b**.

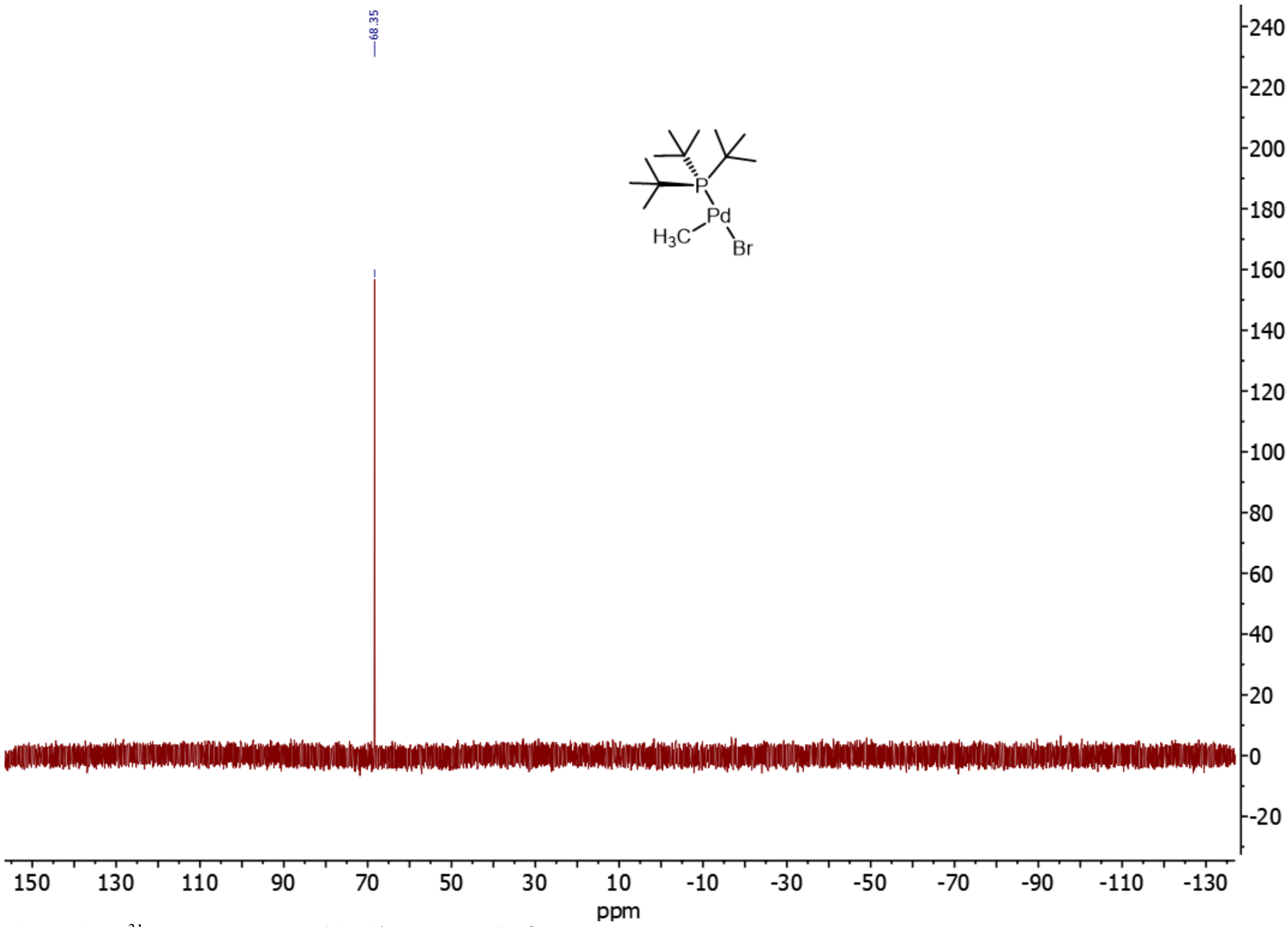

Figure S11. ^{31}P NMR spectrum ($CDCl_3$, 121 MHz) of **1b**.

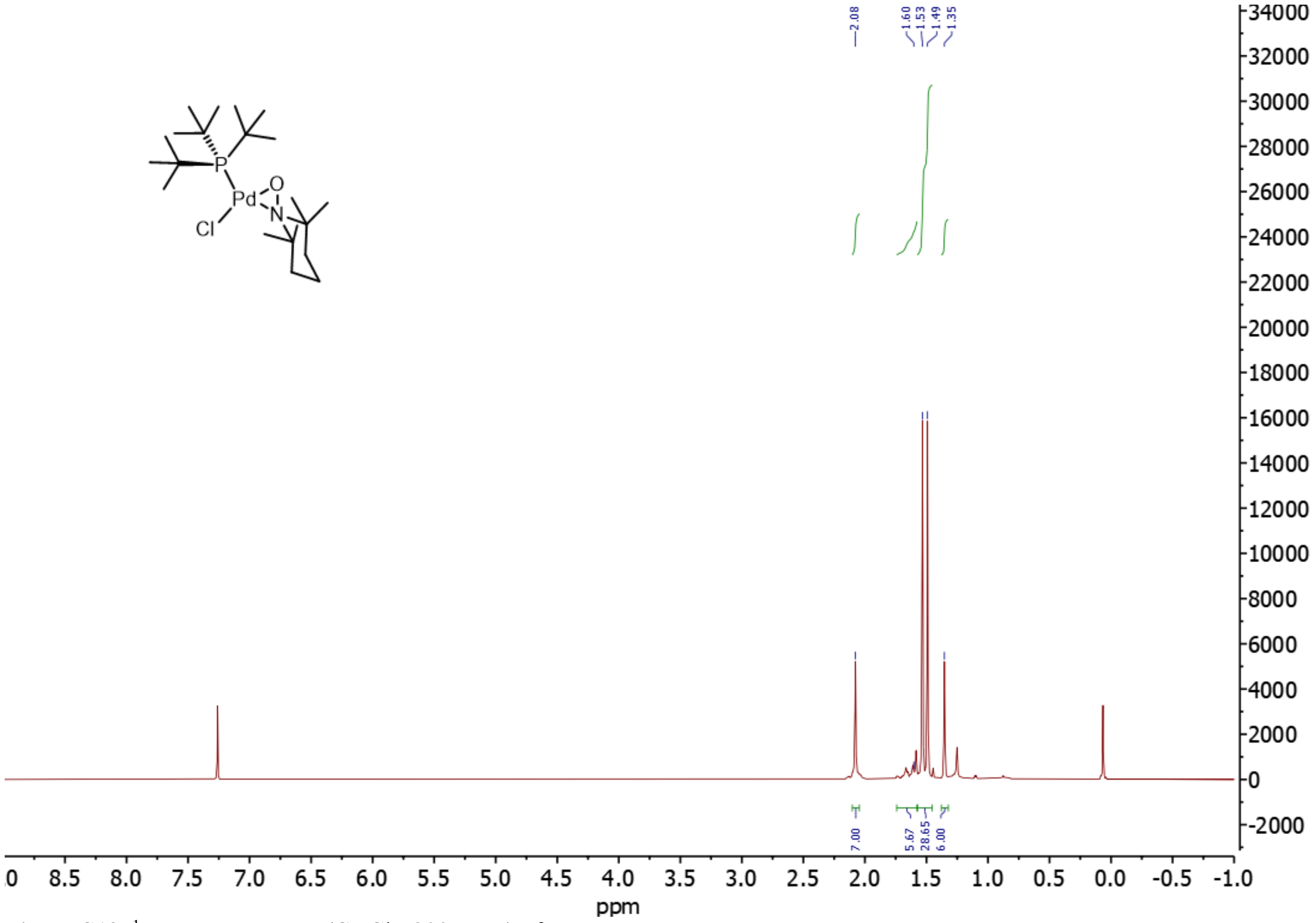

Figure S12. ^{1}H NMR spectrum ($CDCl_3$, 300 MHz) of **5a**.

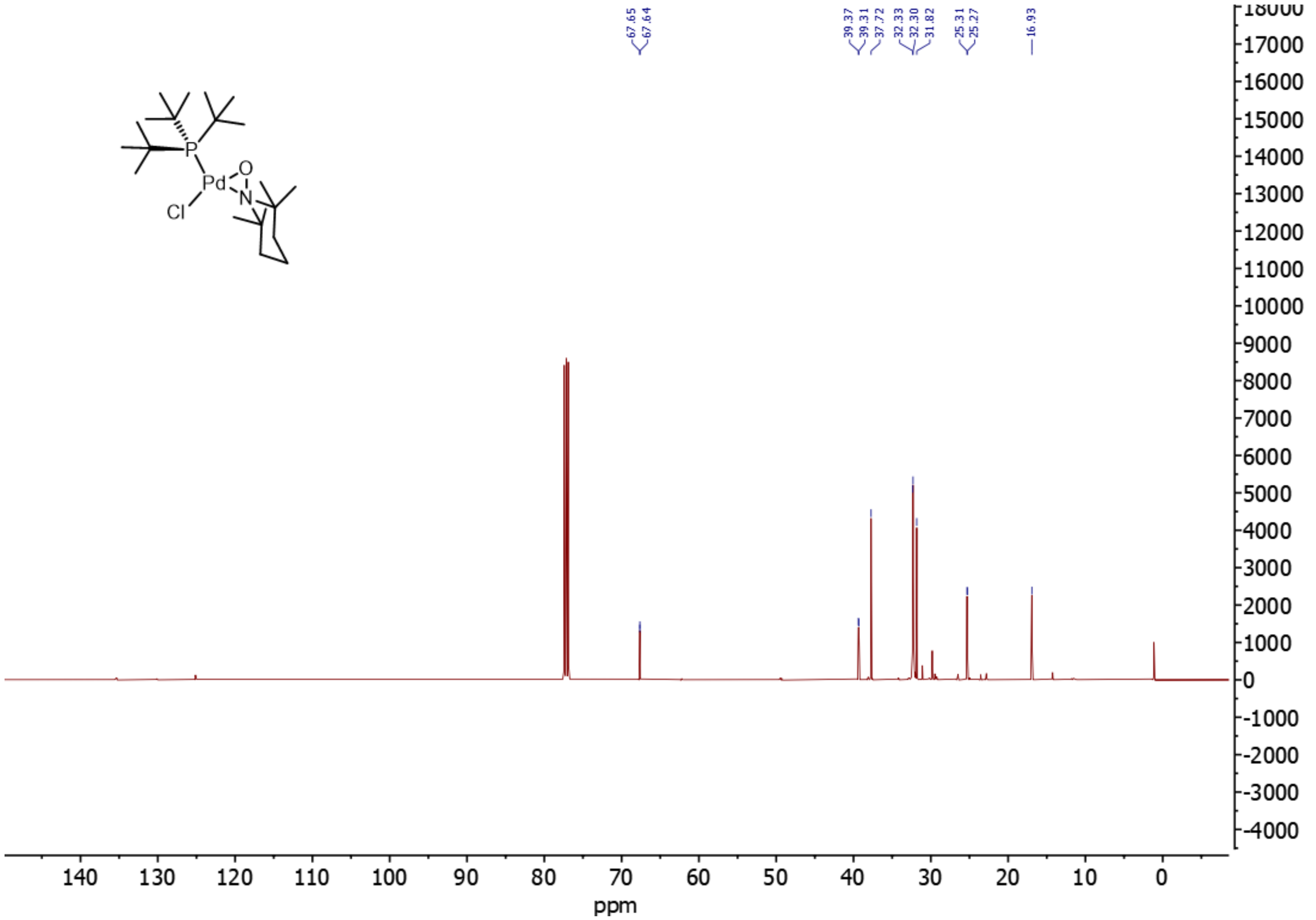

Figure S13. ^{13}C NMR spectrum ($CDCl_3$, 126 MHz) of **5a**.

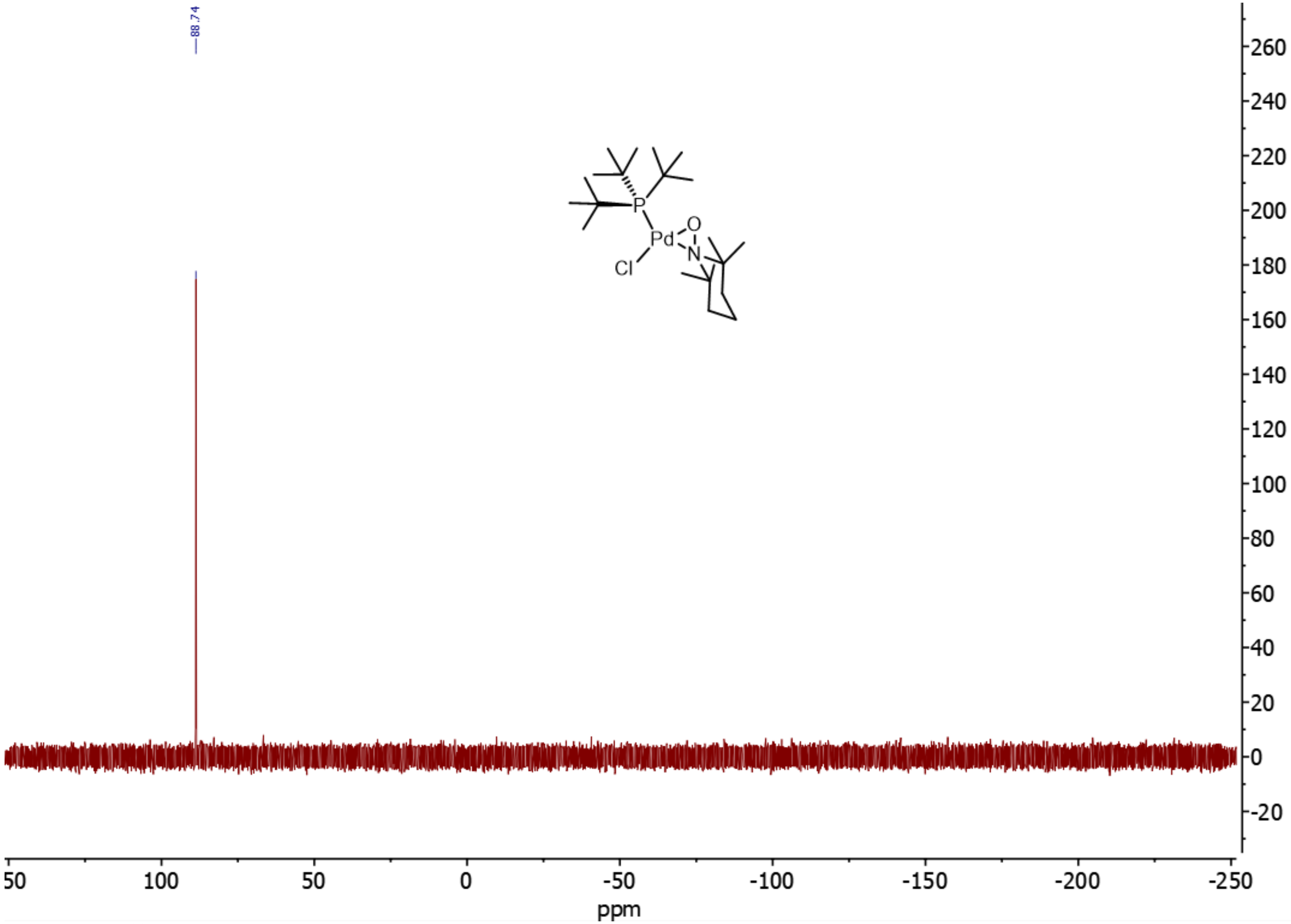

Figure S14. ^{31}P NMR spectrum ($CDCl_3$, 121 MHz) of **5a**.

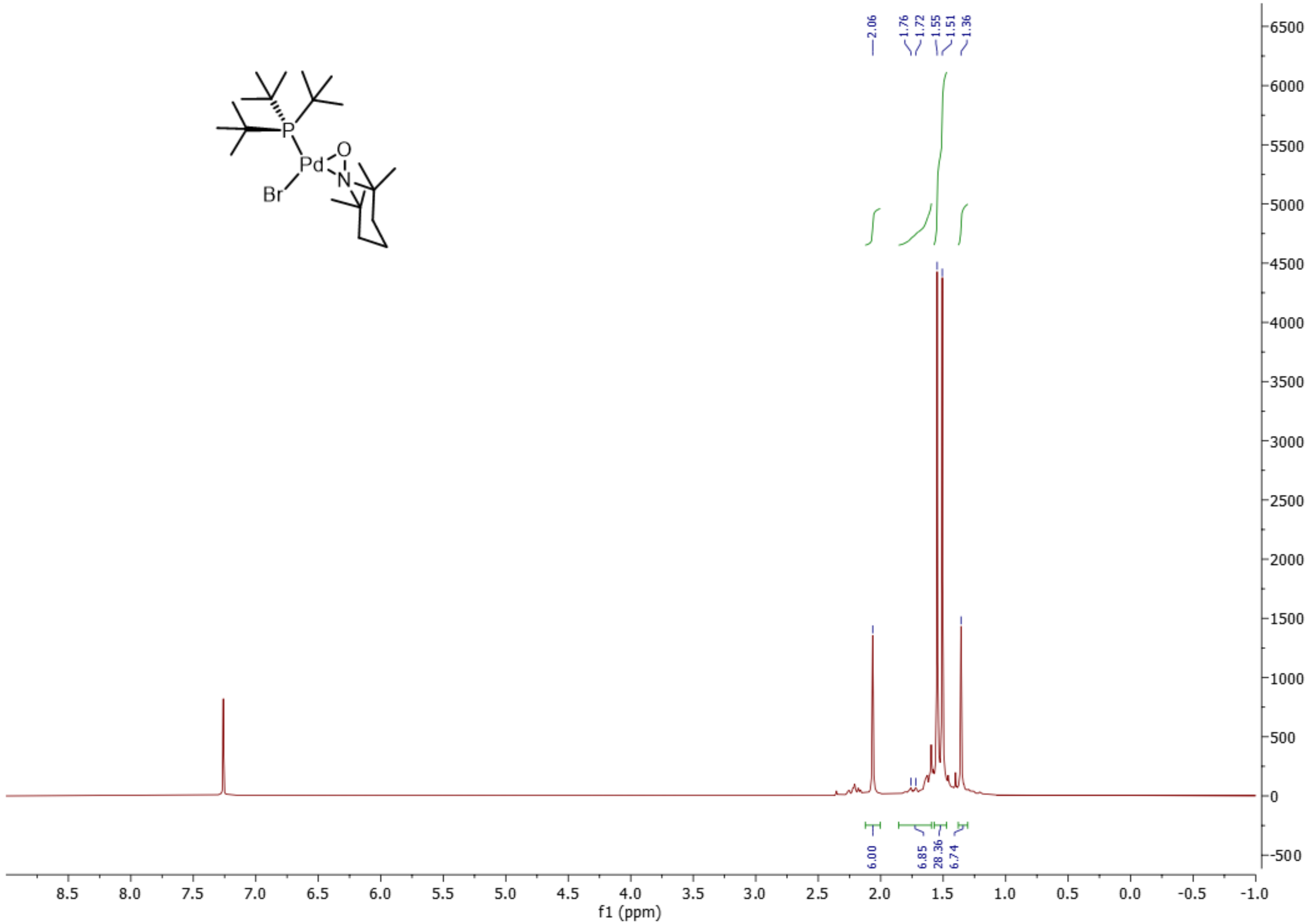

Figure S15. ^{1}H NMR spectrum ($CDCl_3$, 300 MHz) of **5b**.

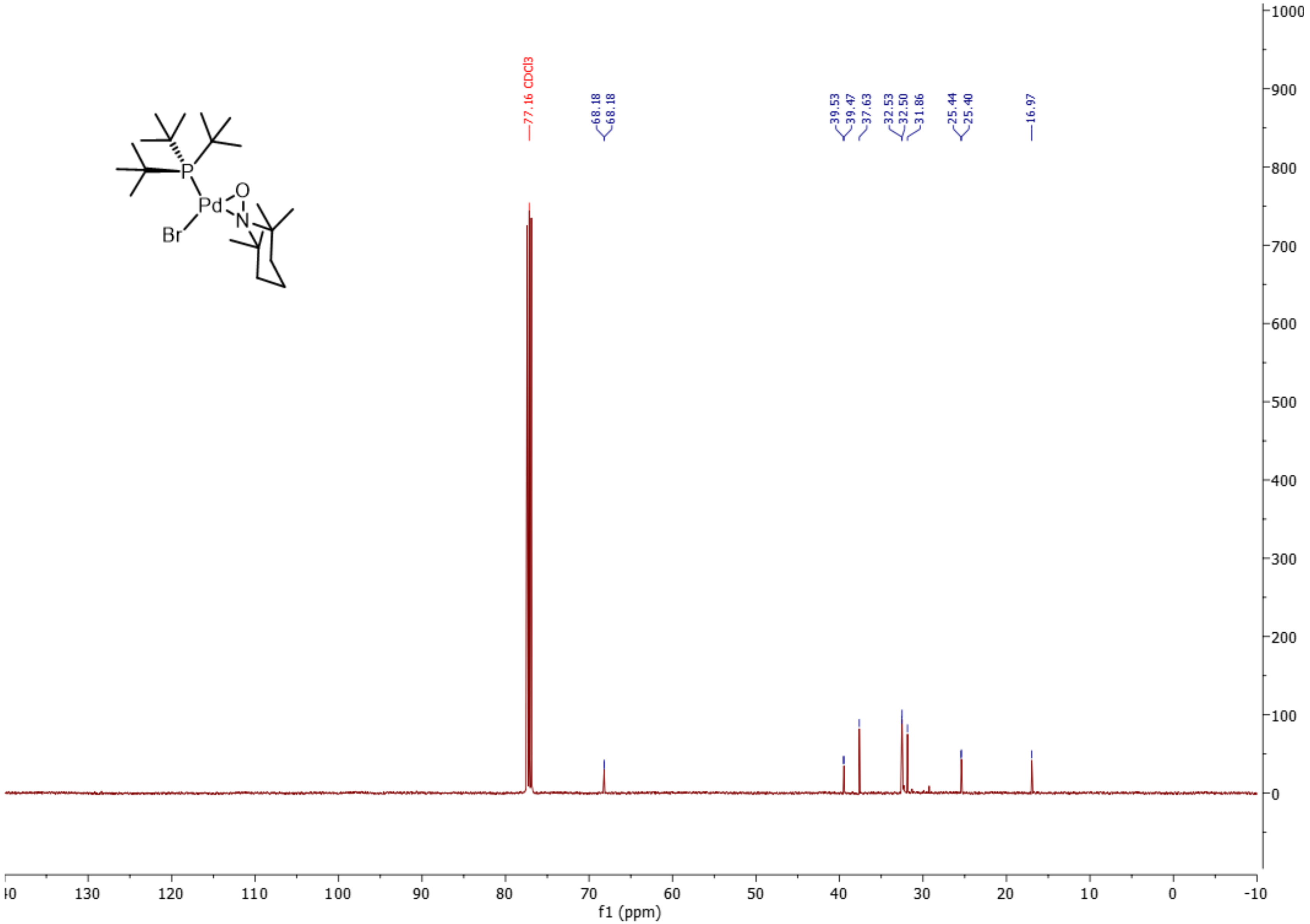

Figure S16. ^{13}C NMR spectrum ($CDCl_3$, 126 MHz) of **5b**.

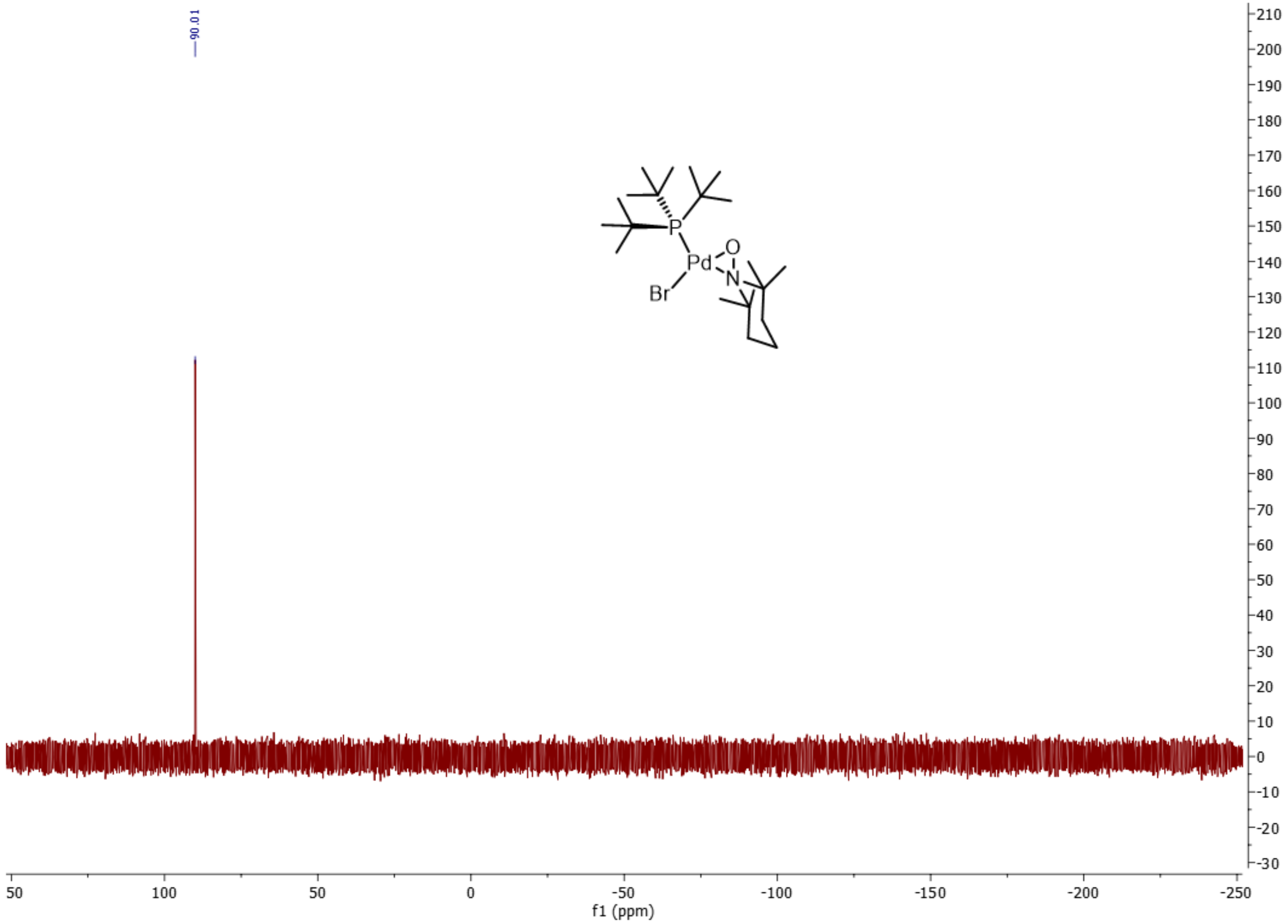

Figure S17. ^{31}P NMR spectrum ($CDCl_3$, 121 MHz) of **5b**.

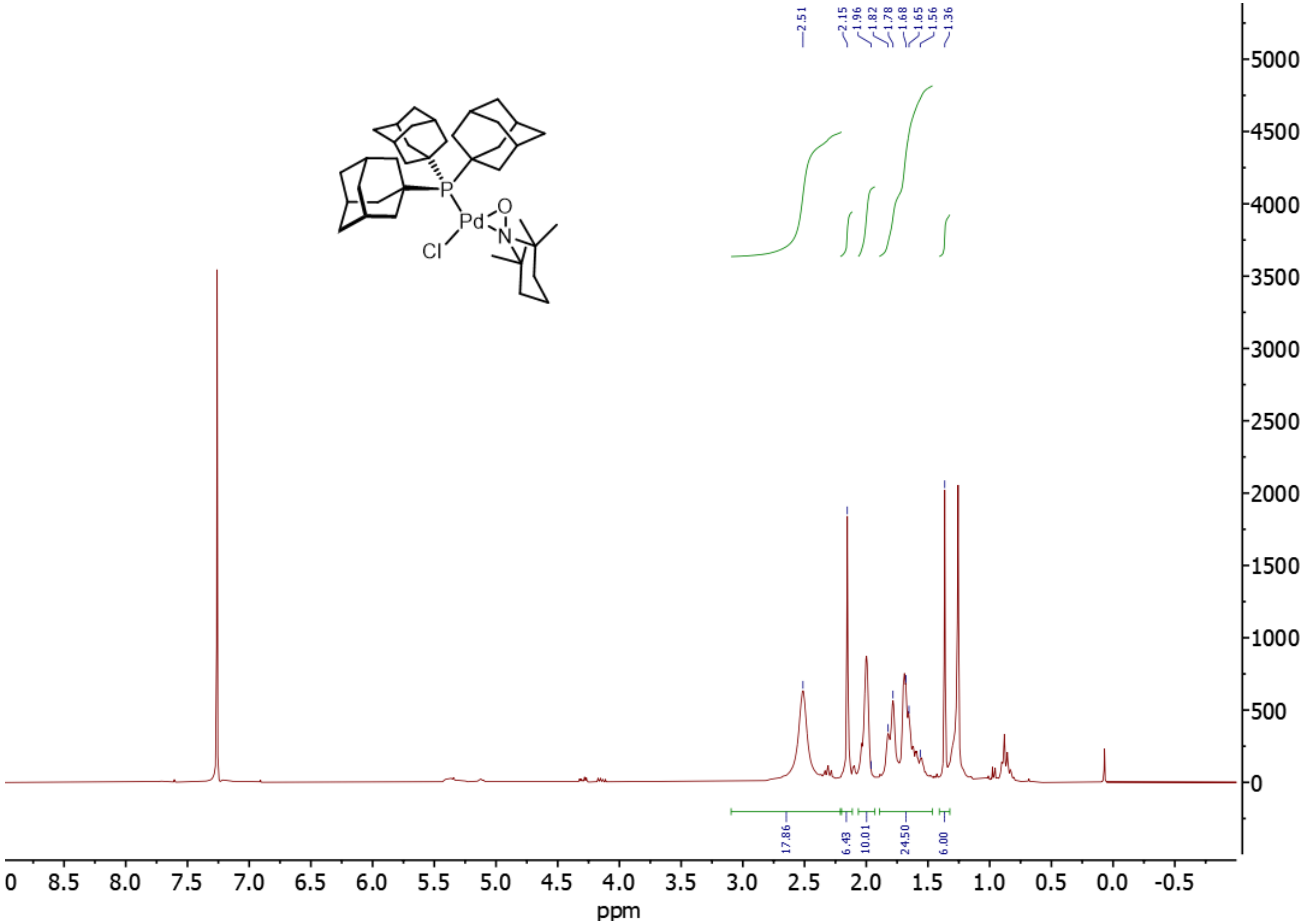

Figure S18. ^{1}H NMR spectrum ($CDCl_3$, 300 MHz) of **S2**.

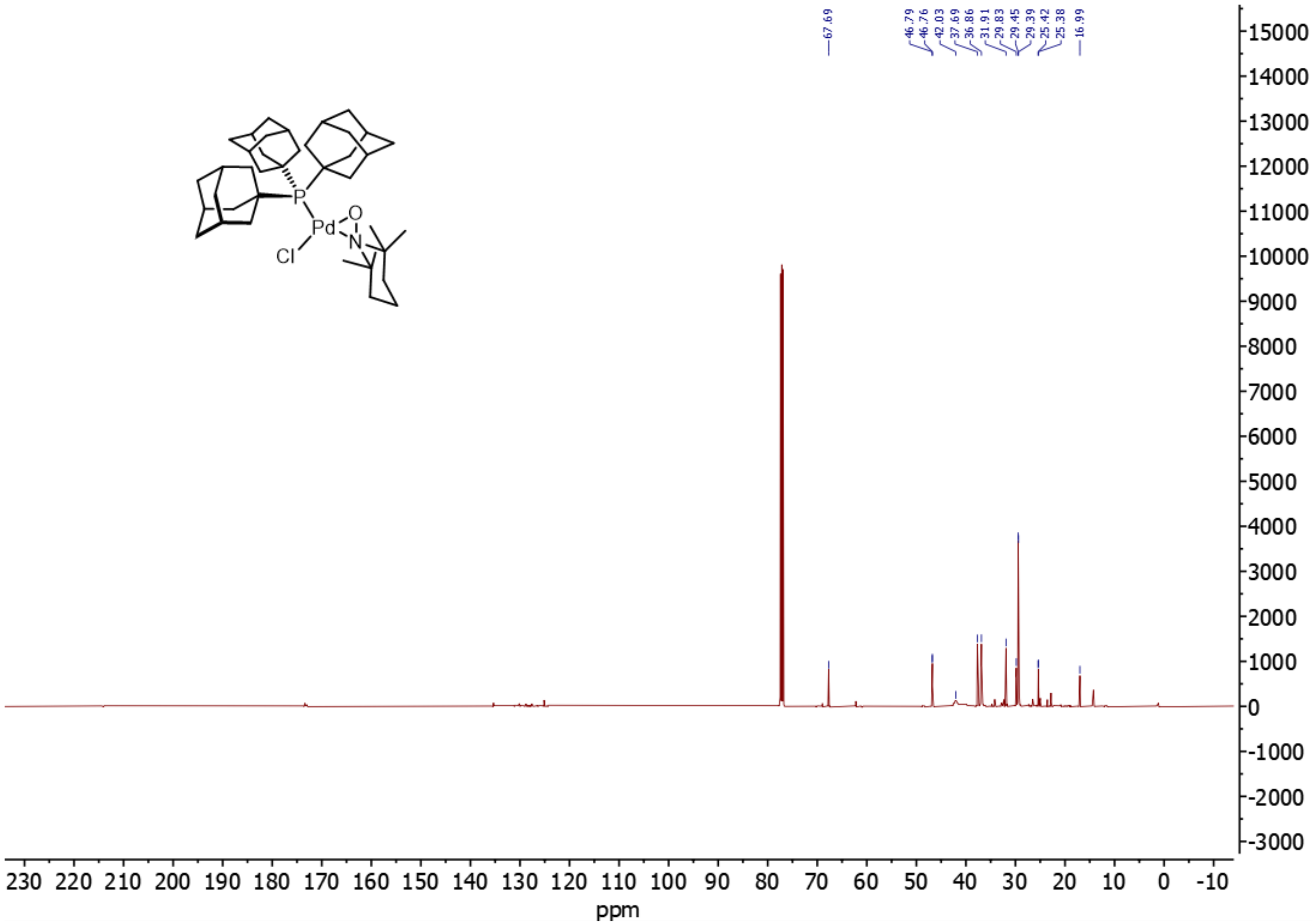

Figure S19. ^{13}C NMR spectrum ($CDCl_3$, 126 MHz) of **S2**.

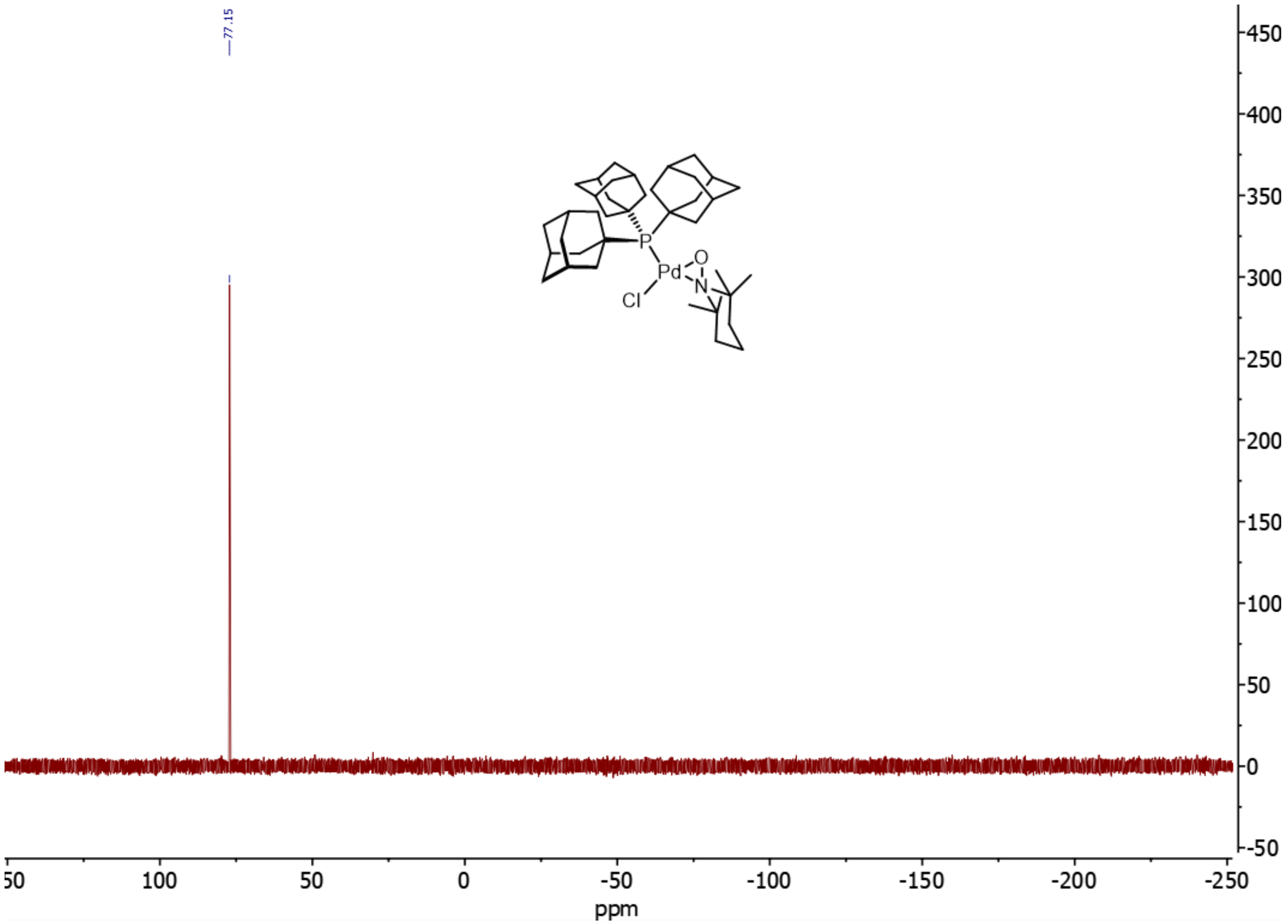

Figure S20. ^{31}P NMR spectrum ($CDCl_3$, 121 MHz) of **S2**.

Procedures for observing light reactivity

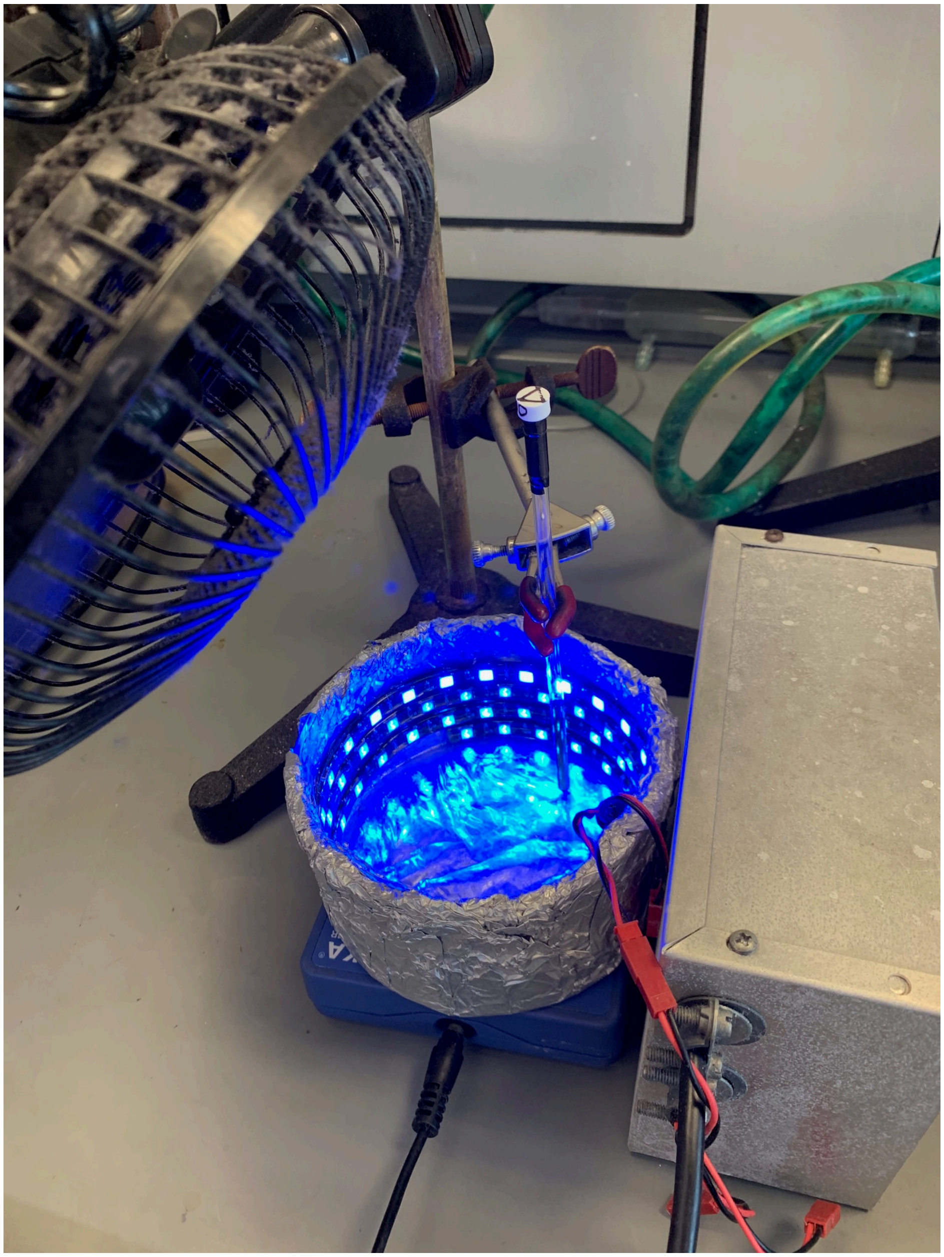

Figure S21. Photograph showing a representative setup of the LED dish, with fan and NMR tube containing reaction mixture.

Irradiation of 1a with blue LEDs in $CDCl_3$

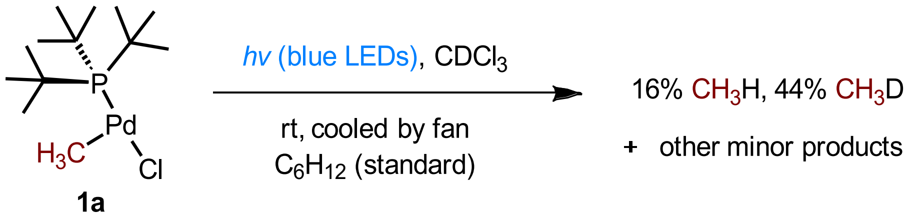

In a nitrogen-filled glovebox, a solution of **1a** (6 mg, 17 μmol) in $CDCl_3$ (0.5 mL) was prepared. 1,3,5-tris(trifluoromethyl)benzene (10 μL, 54 μmol) was added as a standard. This solution was transferred to a J Young NMR tube and additional $CDCl_3$ was added such that the headspace in the tube, after it was sealed with the cap, was minimized (about 2.9 mL total). This was done in order to prevent the volatile products from escaping into the headspace of the tube as much as possible. The J Young tube was then sealed with a cap, then exported from the glovebox. Initial ^{1}H and ^{31}P spectra were recorded, showing essentially no conversion of **1a** before irradiation. The tube was then placed in an LED dish about 2 cm from the LEDs. A fan was placed above the dish and used to cool the reaction mixture. After 12 hours, the tube was removed from the LED dish and ^{1}H and ^{31}P NMR were recorded. Conversion and yields of product were determined by integration of the ^{1}H NMR spectrum, comparing to the initial ^{1}H NMR spectrum and deconvoluting to compensate for the overlap between the CH_4 and CH_3D peaks. Full conversion of **1a** was observed.

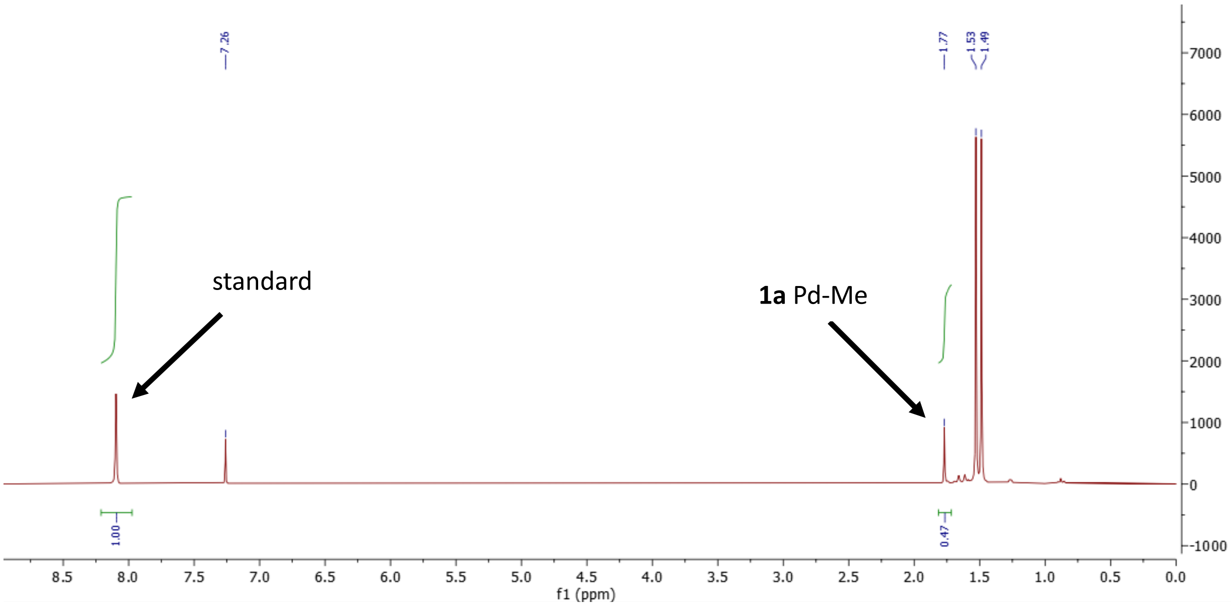

Figure S22. Initial ^{1}H spectrum before irradiation.

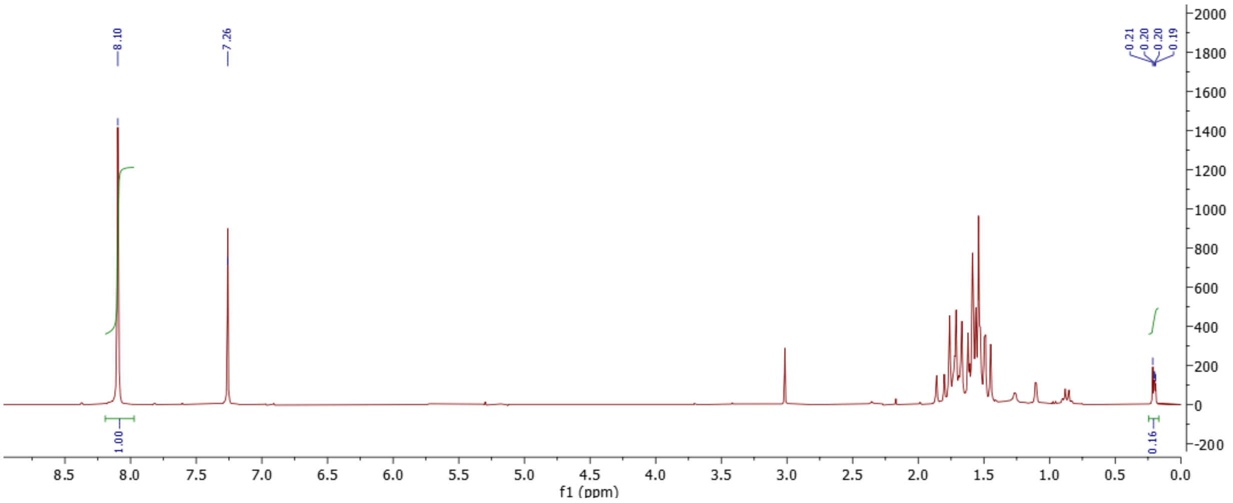

Figure S23. Final ^{1}H spectrum.

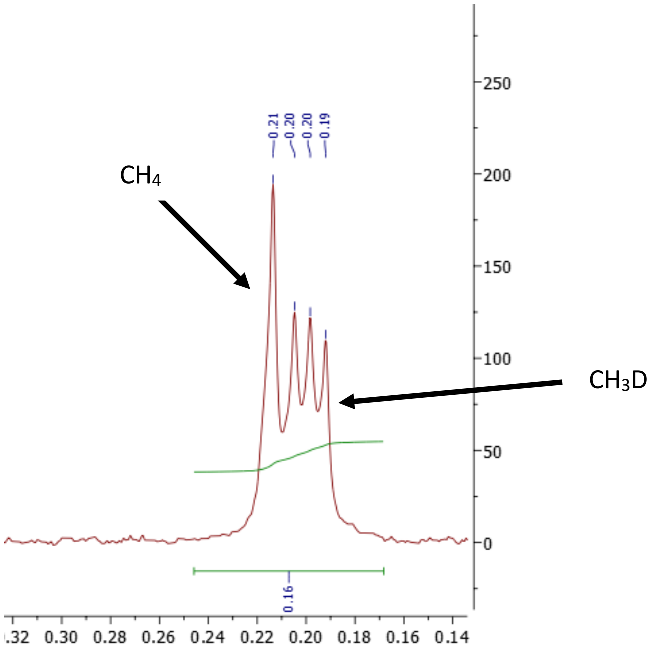

Figure S24. Detail of final ^{1}H spectrum showing peaks for CH_4 and CH_3D.

Irradiation of 1a with blue LEDs in $CDCl_3$ in the presence of 1,4-CHD

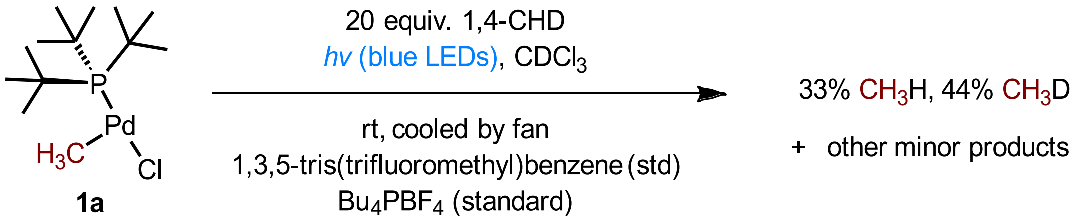

In a nitrogen-filled glovebox, a solution of **1a** (6 mg, 17 µmol) and Bu_4PBF_4 (6 mg, 17 µmol, standard) in $CDCl_3$ (0.5 mL) was prepared. 1,4-cyclohexadiene (1,4-CHD) was added (32 µL, 330 µmol, 20 equiv.) and 1,3,5-tris(trifluoromethyl)benzene (10 µL, 54 µmol) was added as a standard. This solution was transferred to a J Young NMR tube and additional $CDCl_3$ was added such that the headspace in the tube, after it was sealed with the cap, was minimized (about 2.9 mL total). This was done in order to prevent the volatile products from escaping into the headspace of the tube as much as possible. The J Young tube was sealed with a cap, then exported from the glovebox. Initial ^{1}H and ^{31}P spectra were recorded, showing essentially no conversion of **1a** before irradiation. The tube was then placed in an LED dish about 2 cm from the LEDs. A fan was placed above the dish and used to cool the reaction mixture. After 12 hours, the tube was removed from the LED dish and ^{1}H and ^{31}P NMR were recorded. Conversion and yields of product were determined by integration of the ^{1}H NMR spectrum, comparing to the initial ^{1}H NMR spectrum and deconvoluting to compensate for the overlap between the CH_4 and CH_3D peaks. Full conversion of **1a** was observed by ^{31}P NMR.

Note that in this case, the amount of benzene that forms is in a large excess of the amount of Pd-Me in the system, and that the 1,4-CHD is fully consumed. Cyclohexane and cyclohexene are also observed to form on ^{1}H NMR. Currently, the underlying process that leads to the 1,4-CHD disproportionation is not understood.

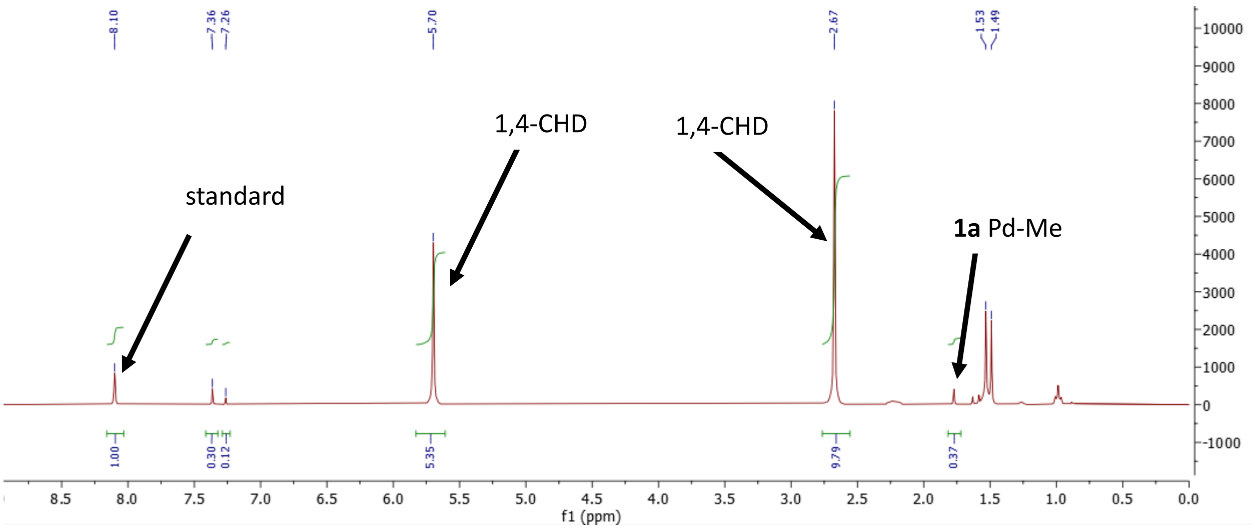

Figure S25. Initial ^{1}H spectrum before irradiation.

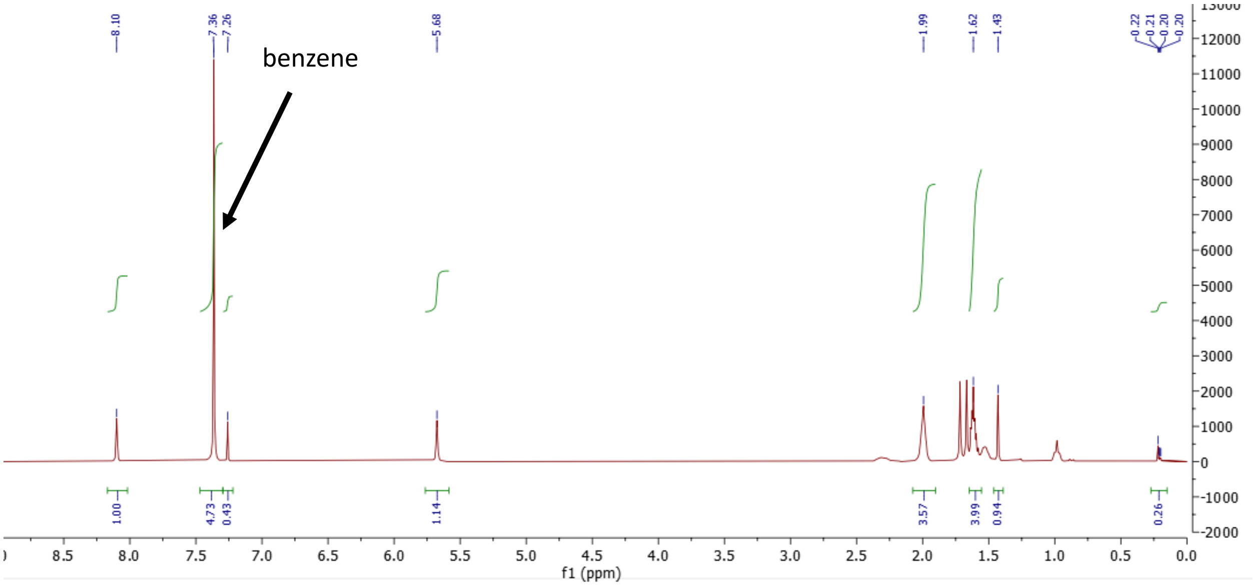

Figure S26. Final ^{1}H spectrum.

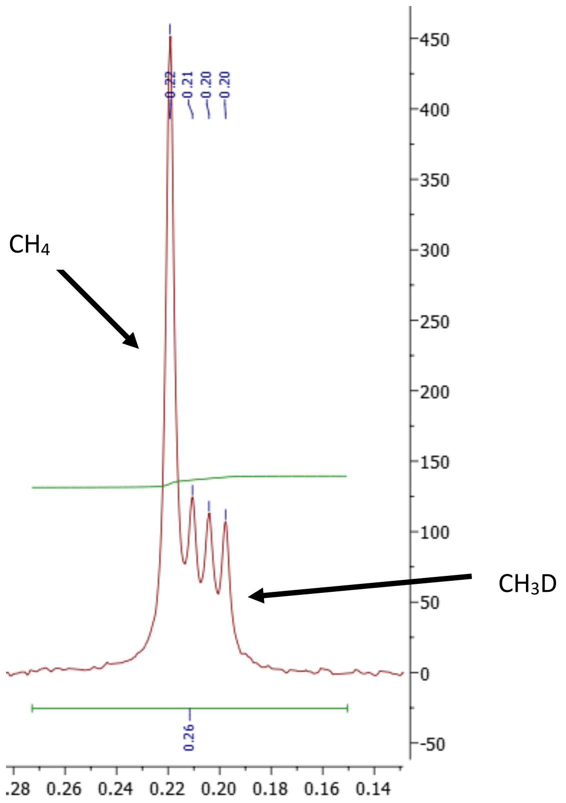

Figure S27. Detail of final ^{1}H spectrum showing peaks for CH_4 and CH_3D.

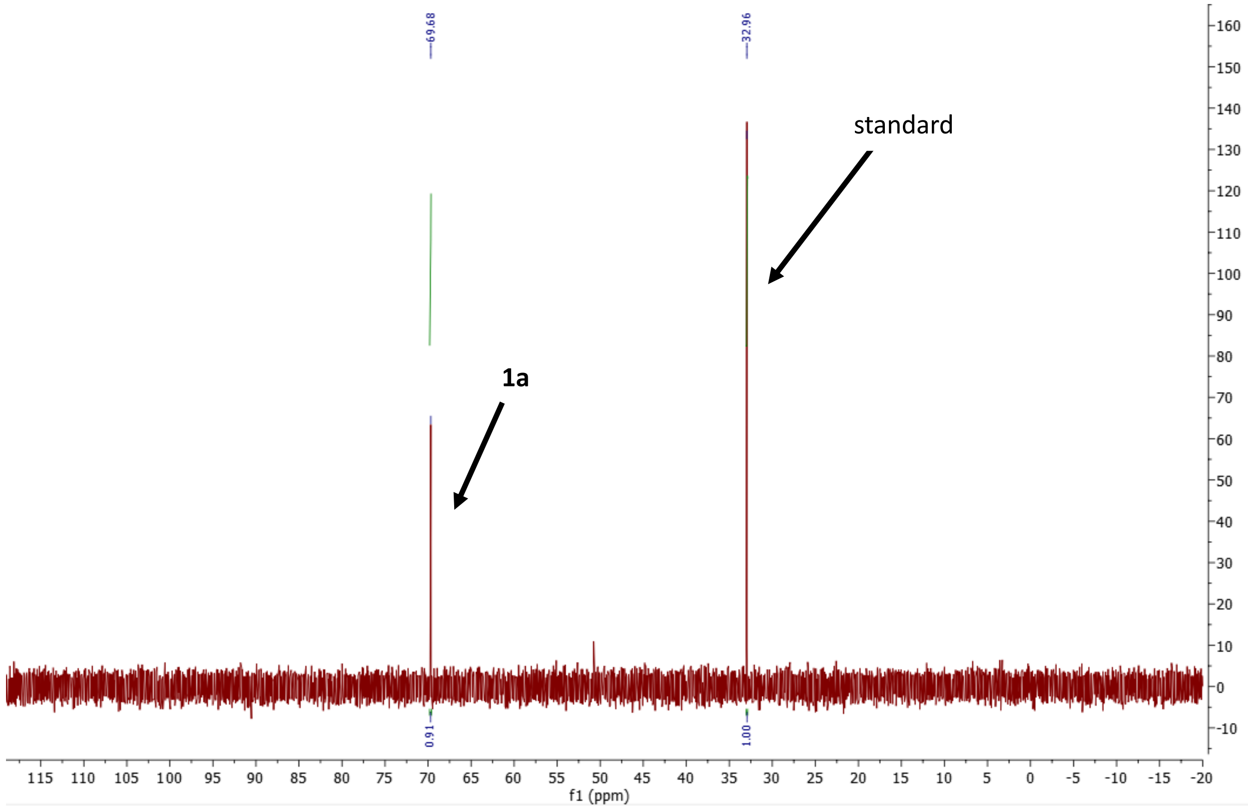

Figure S28. Initial ^{31}P spectrum before irradiation.

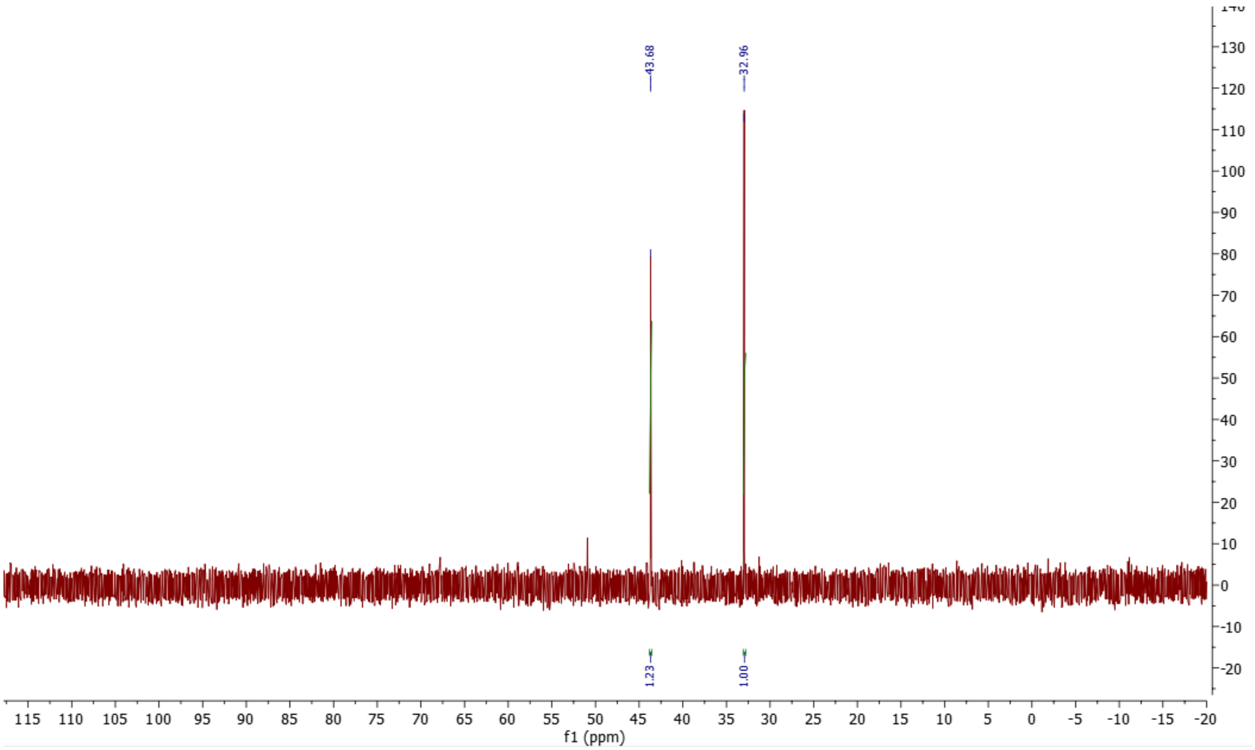

Figure S29. Final ^{31}P spectrum NMR.

Irradiation of 1a with blue LEDs in $CDCl_3$ in the presence of excess TEMPO

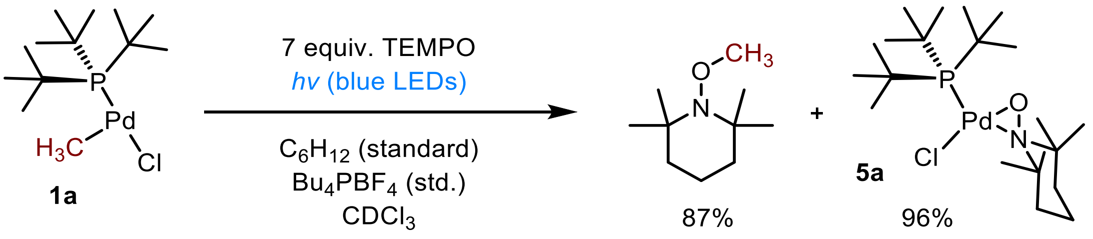

In a nitrogen-filled glovebox, a solution of **1a** (2.4 mg, 6.7 µmol), TEMPO (7.4 mg, 47 µmol, 7 equiv.) and Bu_4PBF_4 (4 mg, 12 µmol, standard) in $CDCl_3$ (756 uL) was prepared. Cyclohexane (4 µL, 37 µmol) was added as a standard. This solution was transferred to an NMR tube and sealed with the cap, then sealed with electrical tape and exported from the glovebox. Initial ^{1}H and ^{31}P spectra were recorded, showing essentially no conversion of **1a** before irradiation. The tube was then placed in an LED dish about 2 cm from the LEDs. A fan was placed above the dish and used to cool the reaction mixture. After 5 hours, the tube was

removed from the LED dish and ^{1}H and ^{31}P NMR were recorded. Conversion and yields of product were determined by integration of the ^{1}H NMR spectrum, comparing to the initial ^{1}H NMR spectrum.

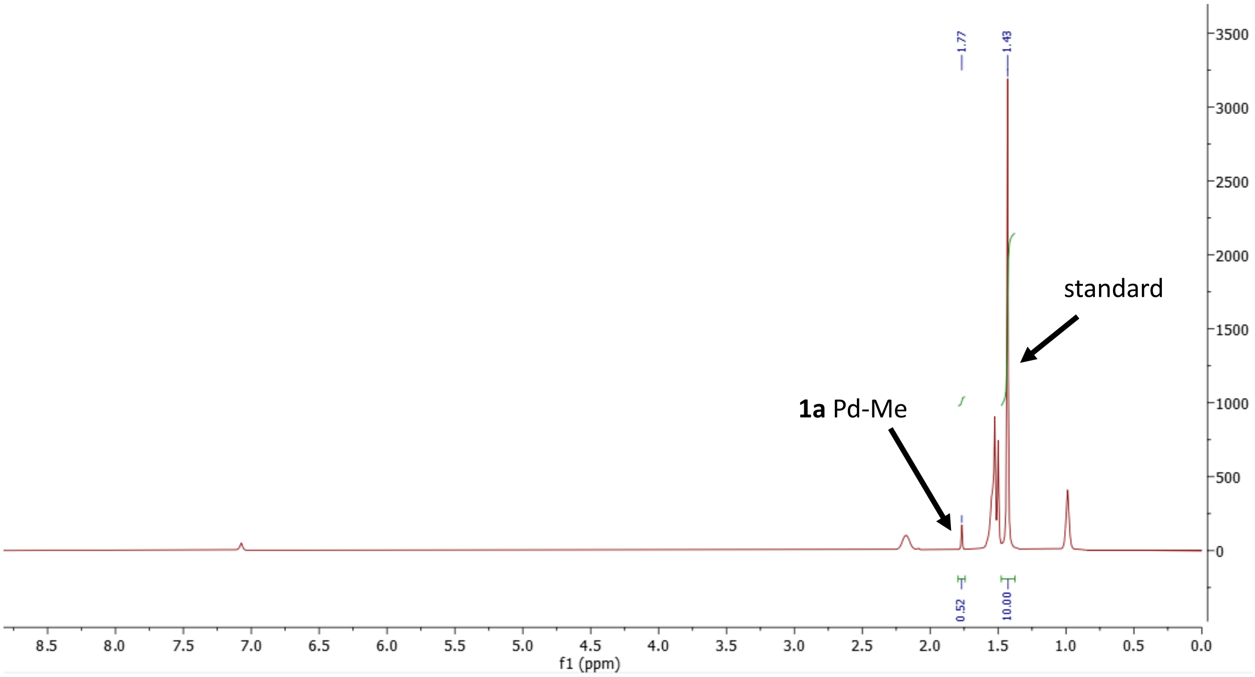

Figure S30. Initial ^{1}H spectrum before irradiation.

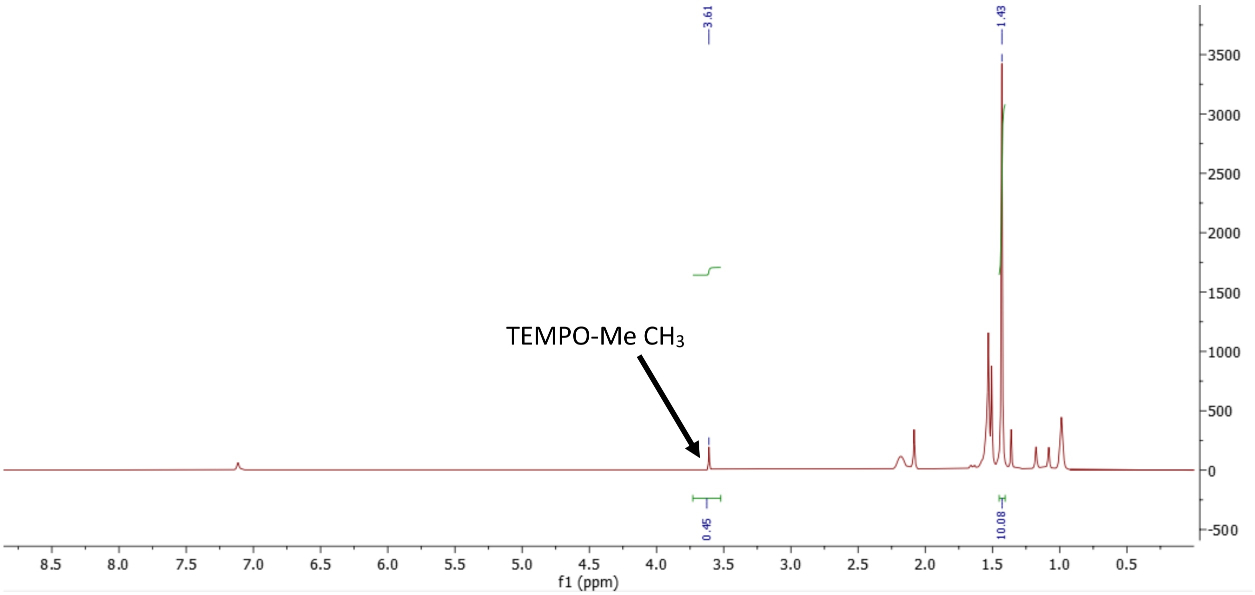

Figure S31. Final ^{1}H spectrum.

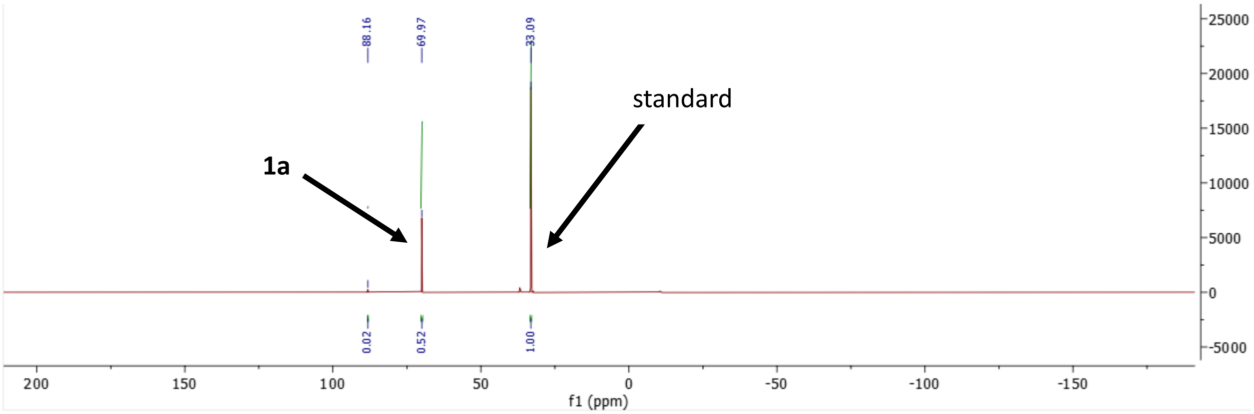

Figure S32. Initial ^{31}P spectrum before irradiation.

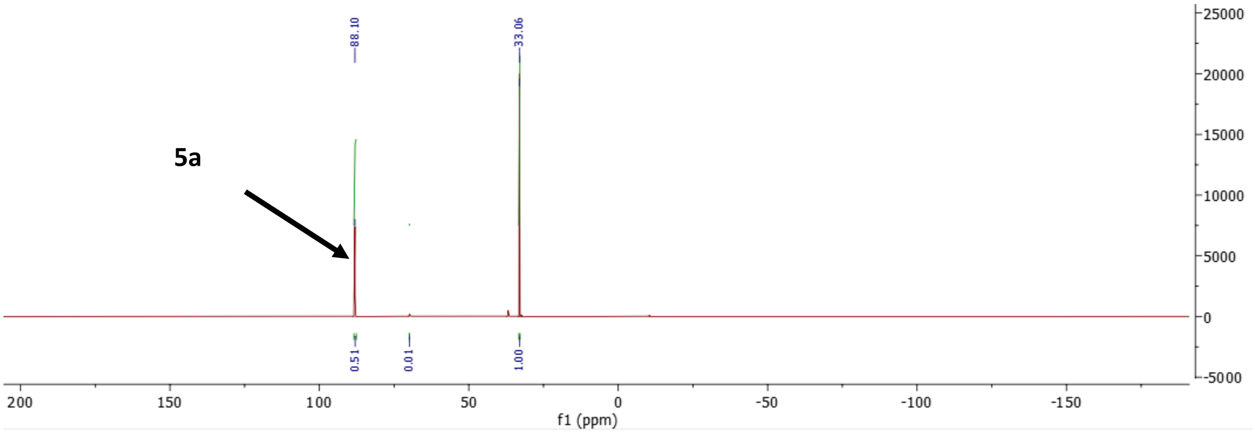

Figure S33. Final ^{31}P spectrum.

Irradiation of 1a with blue LEDs in $CDCl_3$ in the presence of excess TEMPO

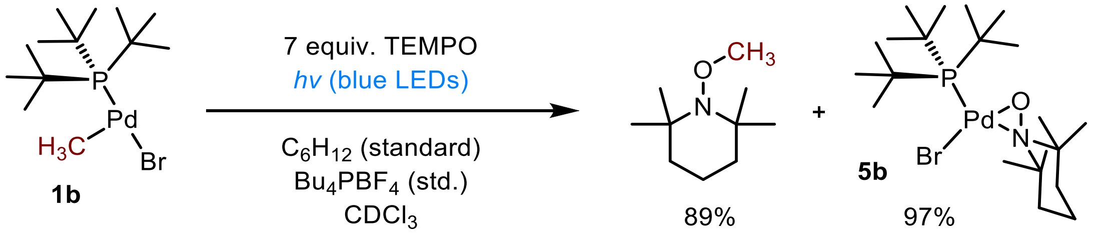

In a nitrogen-filled glovebox, a solution of **1b** (2.6 mg, 6.7 µmol), TEMPO (7 mg, 47 µmol, 7 equiv.) and Bu_4PBF_4 (4 mg, 12 µmol, standard) in $CDCl_3$ (756 uL) was prepared. Cyclohexane (4 µL, 6.4 µmol) was added as a standard. This solution was transferred to an NMR tube and sealed with the cap, then sealed with electrical tape and exported from the glovebox. Initial ^{1}H and ^{31}P spectra were recorded, showing essentially no conversion of **1b** before irradiation. The tube was then placed in an LED dish about 2 cm from the LEDs. A fan was placed above the dish and used to cool the reaction mixture. After 8 hours, the tube was removed from the LED dish and ^{1}H and ^{31}P NMR were recorded. Conversion and yields of product were determined by integration of the ^{1}H NMR spectrum, comparing to the initial ^{1}H NMR spectrum.

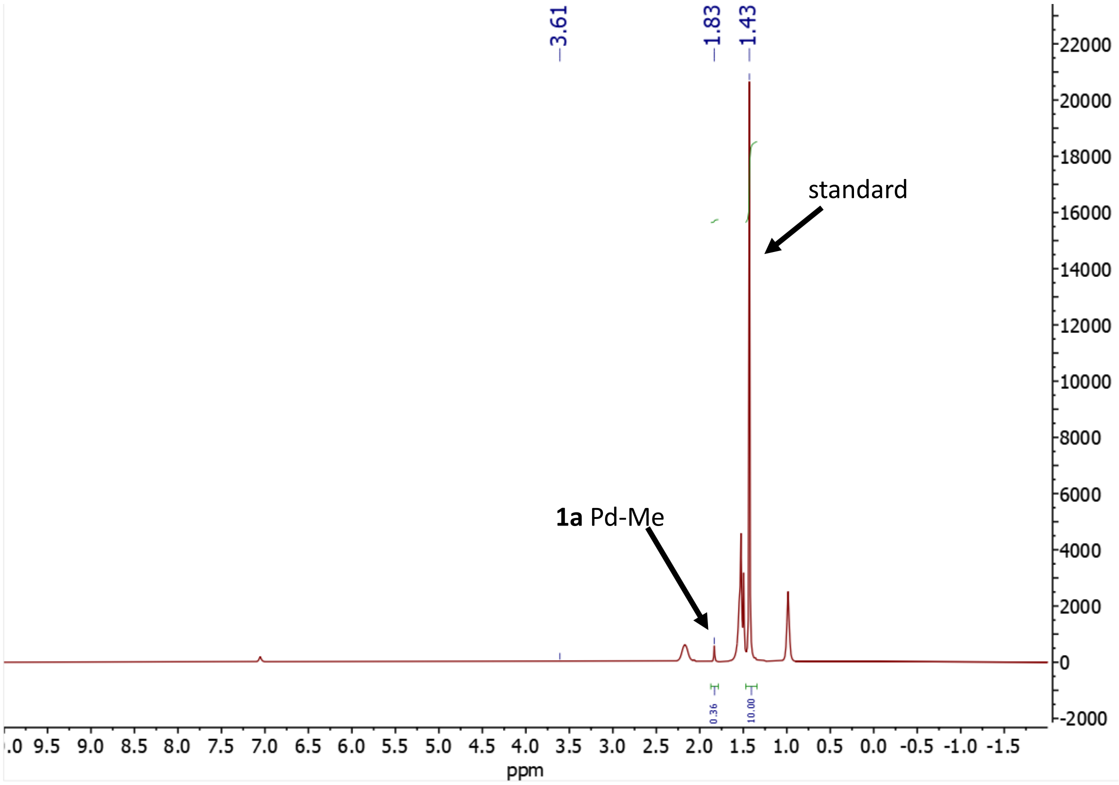

Figure S34. Initial ^{1}H spectrum before irradiation.

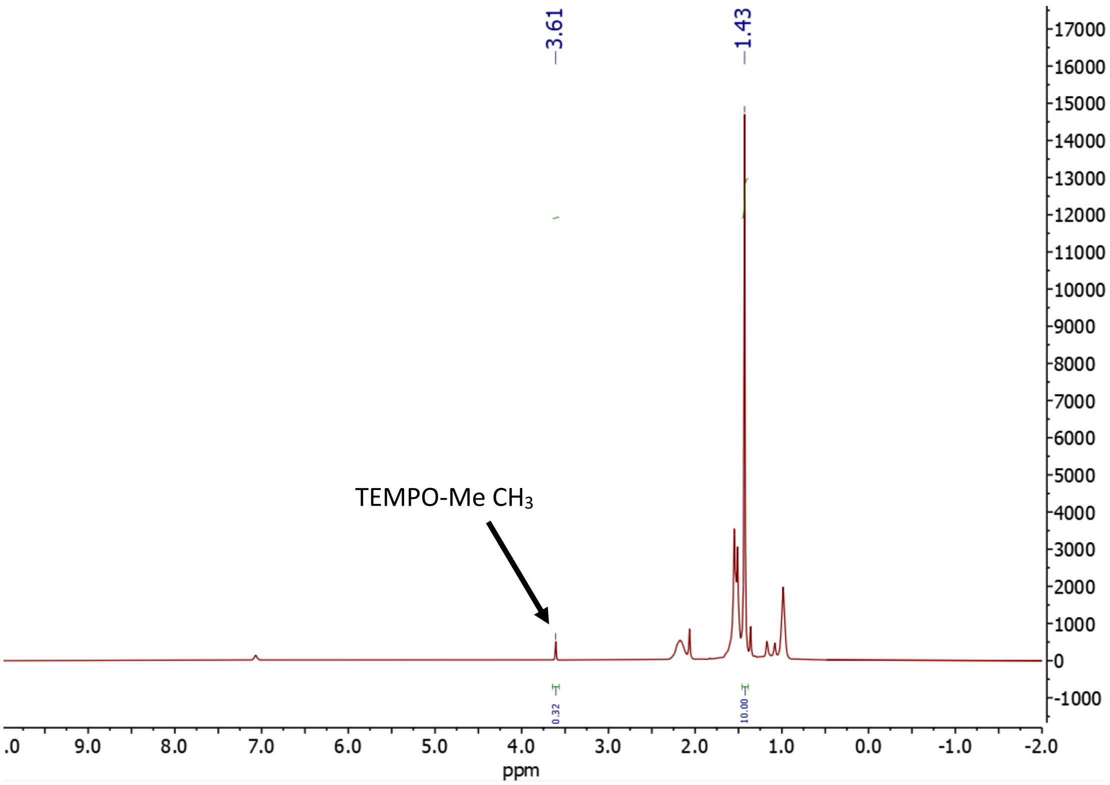

Figure S35. Final ^{1}H spectrum.

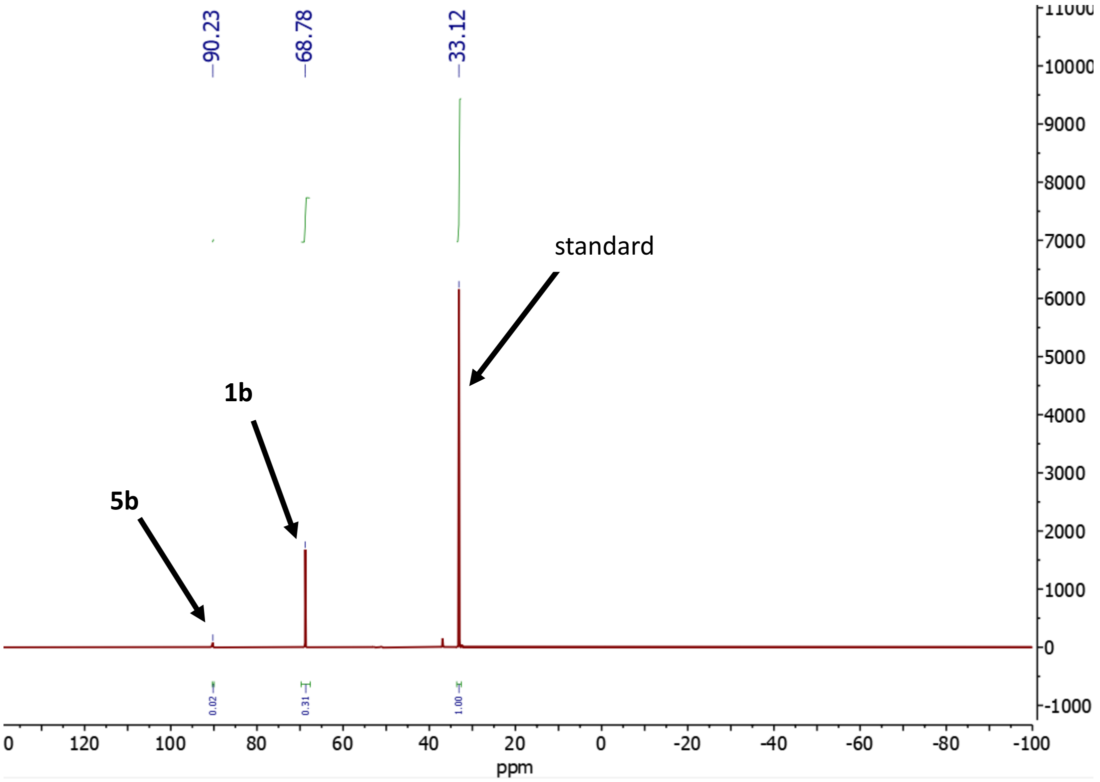

Figure S36. Initial ^{31}P spectrum before irradiation.

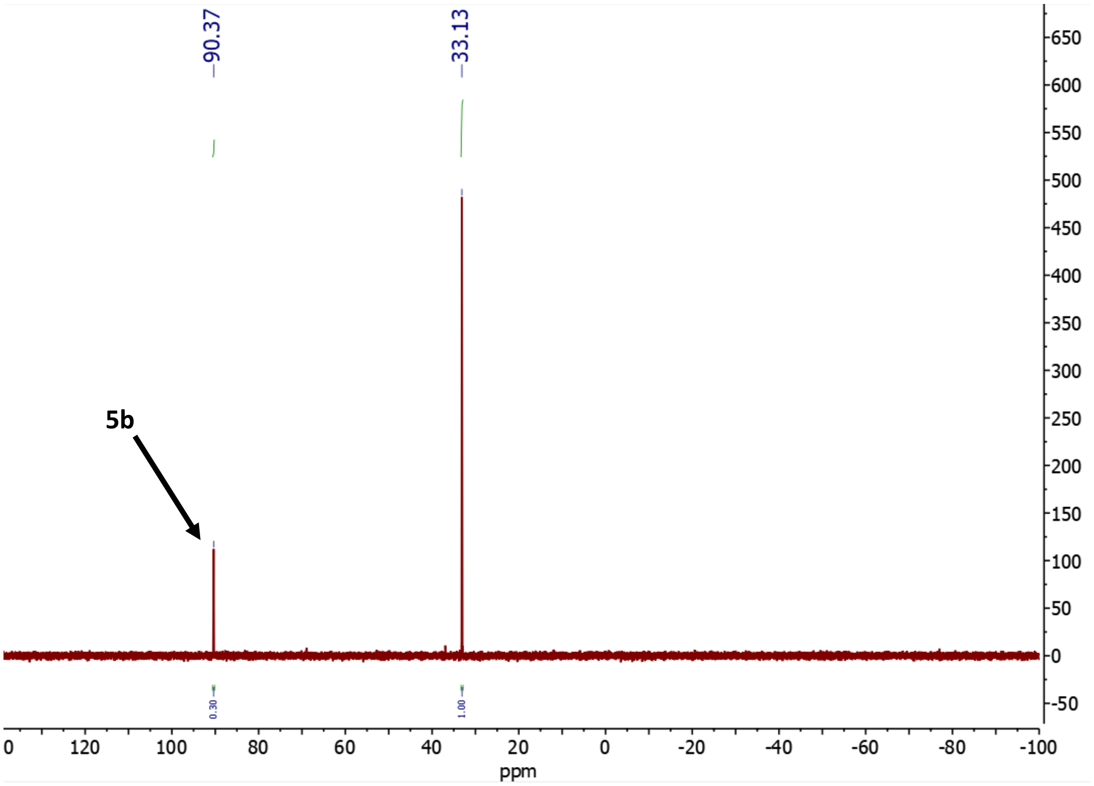

Figure S37. Final ^{31}P spectrum.

Irradiation of 2 with blue LEDs in $CDCl_3$ in the presence of excess TEMPO

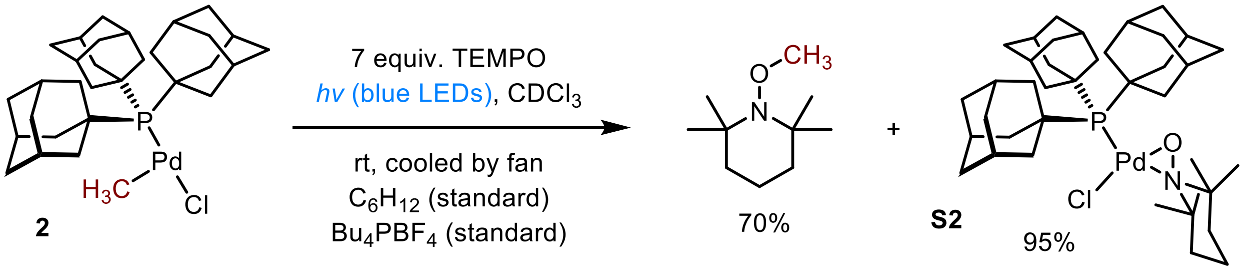

In a nitrogen-filled glovebox, a solution of **2** (4 mg, 6.7 µmol), TEMPO (7 mg, 47 µmol, 7 equiv.) and Bu_4PBF_4 (4 mg, 12 µmol, standard) in $CDCl_3$ (756 µL) was prepared. Cyclohexane (4 µL, 37 µmol) was added as a standard. This solution was transferred to an NMR tube and sealed with the cap, then sealed with electrical tape and exported from the glovebox. Initial ^{1}H and ^{31}P spectra were recorded, showing essentially no conversion of **2** before irradiation. The tube was then placed in an LED dish about 2 cm from the LEDs. A fan was placed above the dish and used to cool the reaction mixture. After 2.5 hours of total irradiation time, the tube was removed from the LED dish and ^{1}H and ^{31}P NMR were recorded. Conversion and yields

of product were determined by integration of the ^{1}H NMR spectrum, comparing to the initial ^{1}H NMR spectrum.

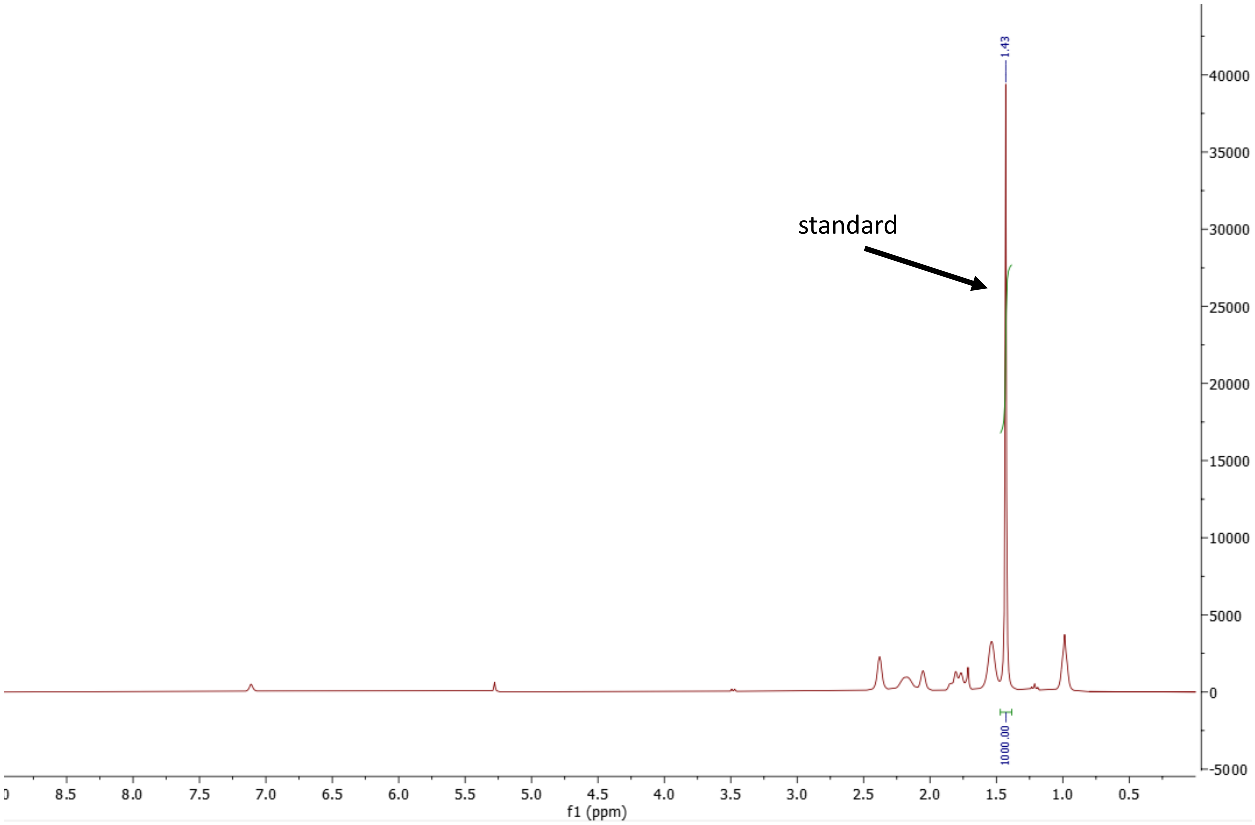

Figure S38. Initial ^{1}H spectrum before irradiation.

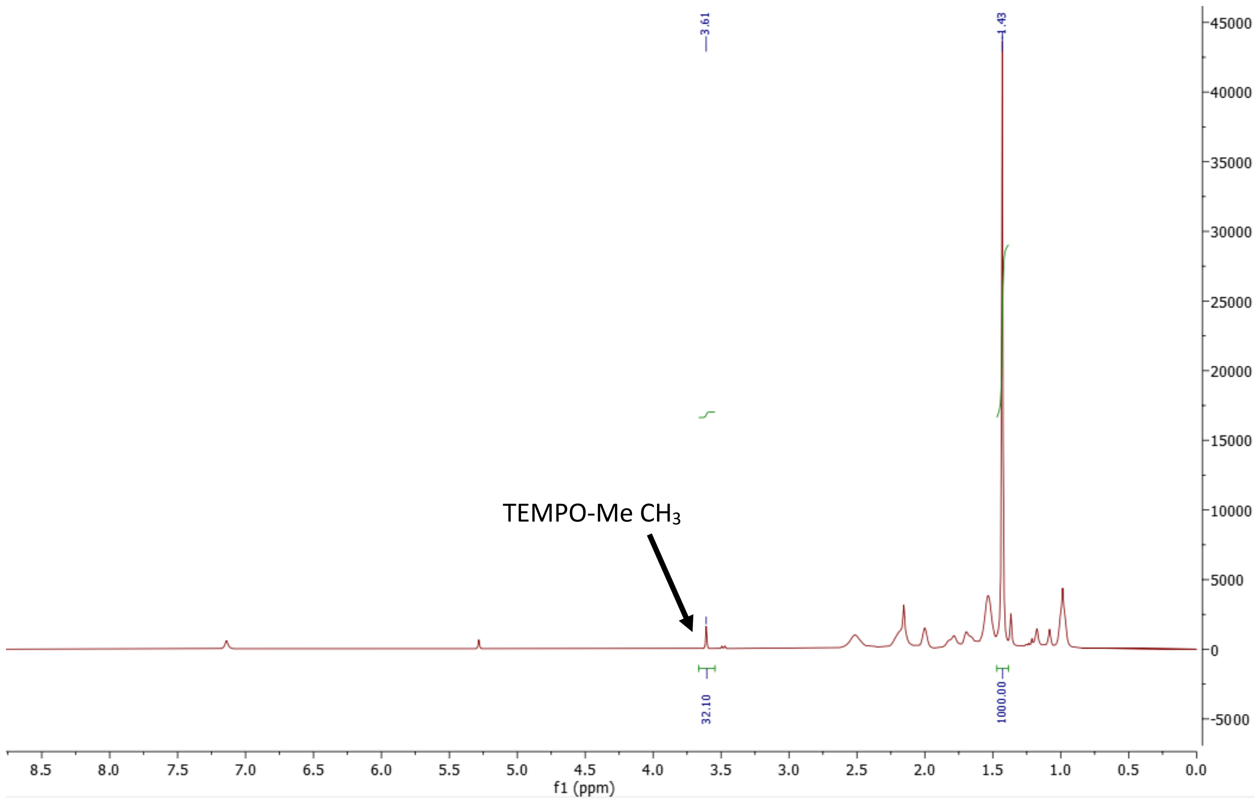

Figure S39. Final ^1H spectrum.

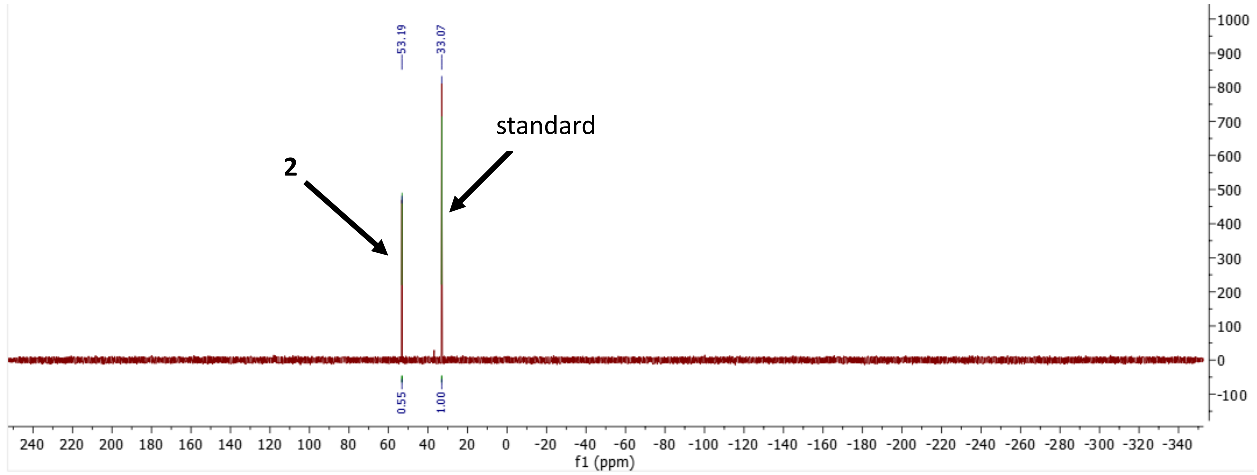

Figure S40. Initial ^{31}P spectrum before irradiation.

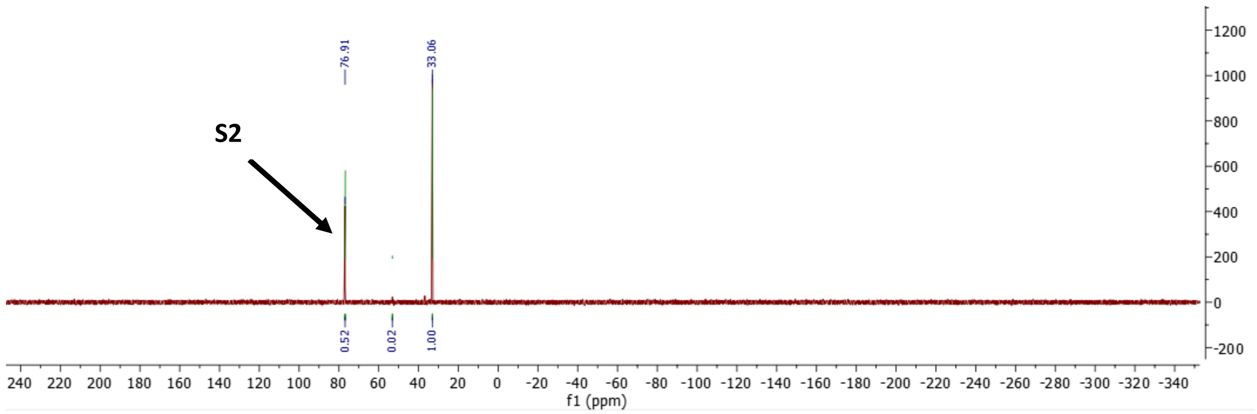

Figure S41. Final ^{31}P spectrum.

Control reaction of mixture of 1a and TEMPO, heated in the dark

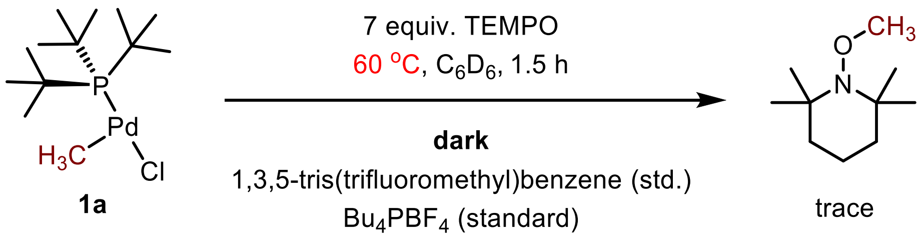

In a nitrogen-filled glovebox with the lights turned off, a solution of **1a** (4 mg, 11 µmol), TEMPO (12 mg, 78 µmol, 7 equiv.) and Bu_4PBF_4 (3.5 mg, 10 µmol, standard) in C_6D_6 (595 µL) was prepared. 1,3,5-tris(trifluoromethyl)benzene (5 µL, 27 µmol) was added as a standard. This solution was transferred to an NMR tube and sealed with the cap, then sealed with electrical tape and aluminum foil around the outside in order to keep light out and exported from the glovebox. Initial ^{1}H and ^{31}P spectra were recorded, showing essentially no conversion of **1a** before irradiation. The tube was then placed in an oil bath heated to 60 °C in the dark. After 1.5 hours, the tube was removed from the oil bath and ^{1}H and ^{31}P NMR were recorded. No significant difference was observed in the spectra before and after heating. The same reaction mixture was then irradiated in the LED dish after 15 hours and showed complete conversion to TEMPO-Me by ^{1}H NMR.

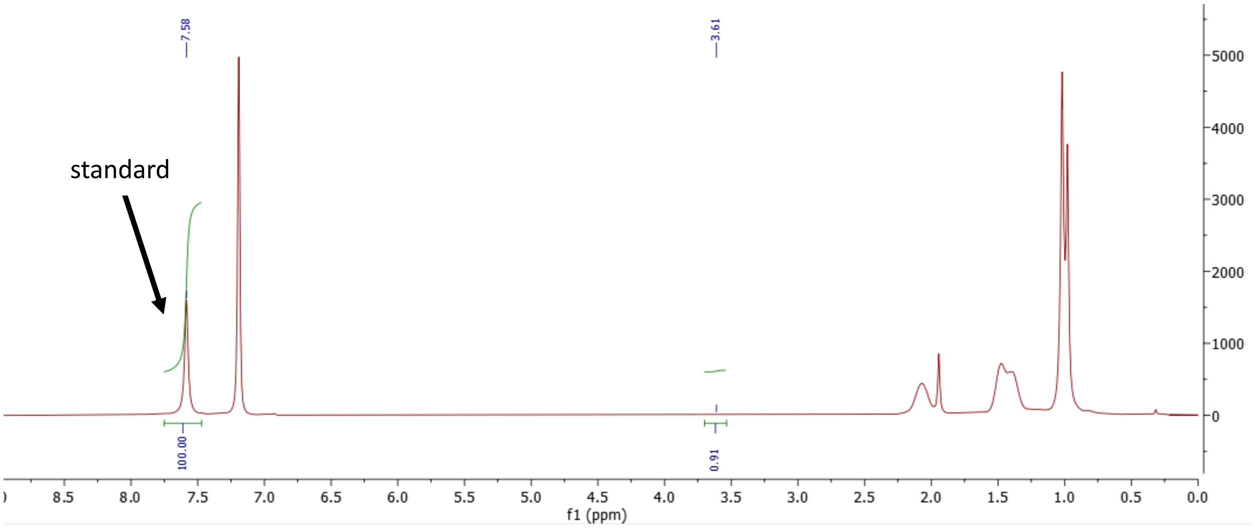

Figure S42. Initial ^{1}H spectrum.

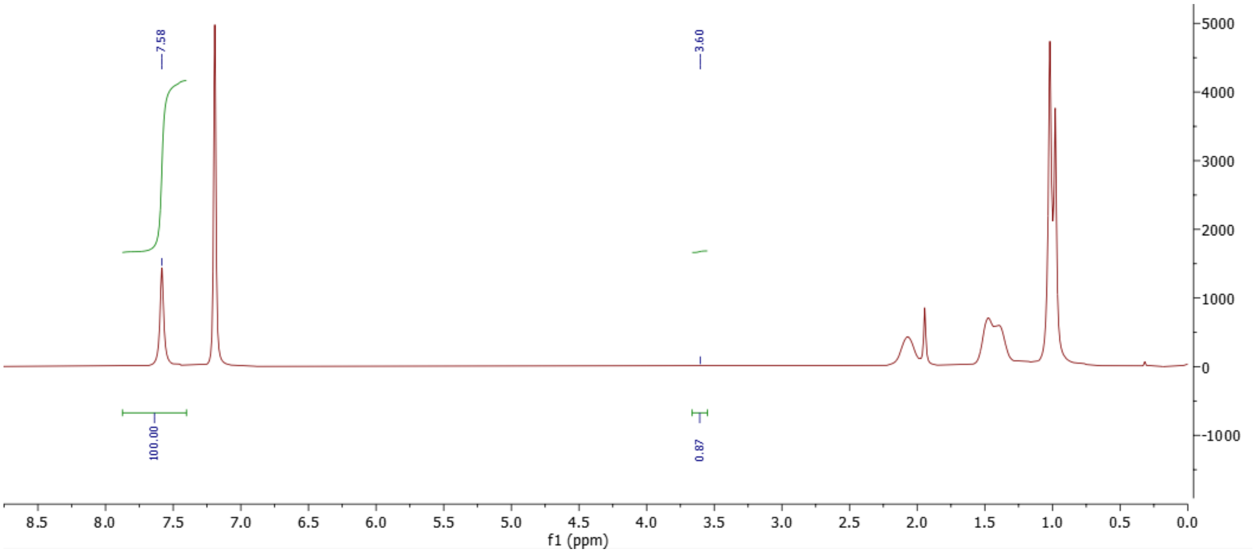

Figure S43. ^{1}H spectrum after 90 minutes heating.

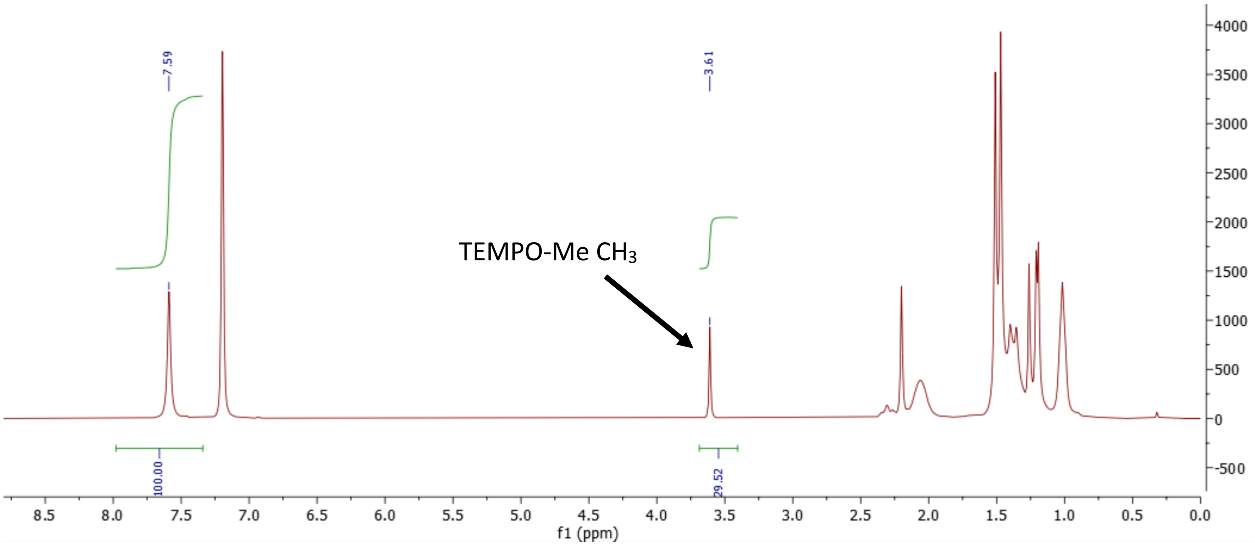

Figure S44. ^{1}H spectrum after 15 hours of irradiation.

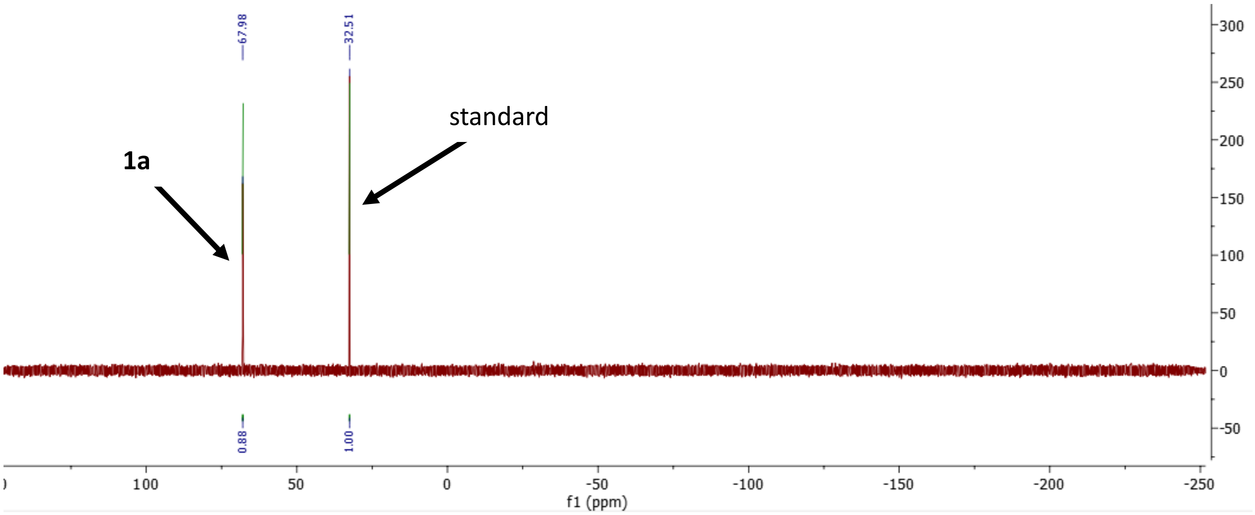

Figure S45. Initial ^{31}P spectrum.

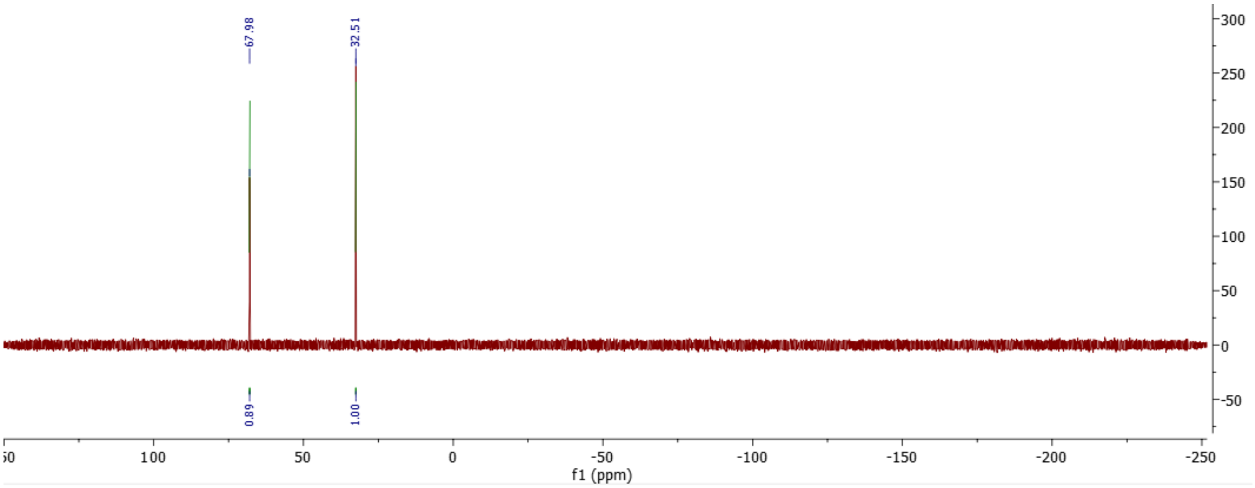

Figure S46. ^{31}P spectrum after 90 minutes of heating.

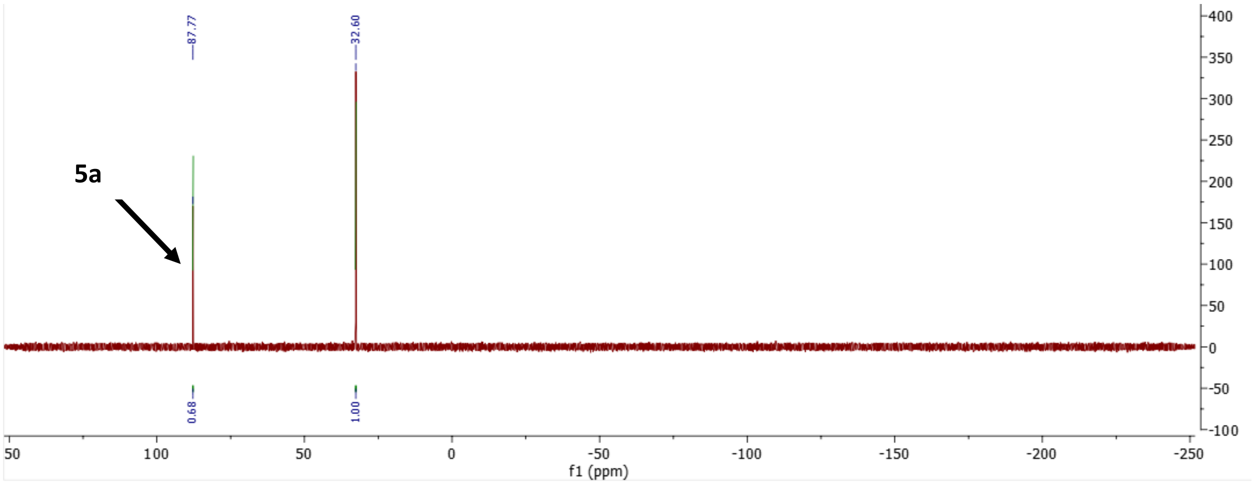

Figure S47. ^{31}P spectrum after 15 hours of irradiation.

Control reaction of mixture of 1a and TEMPO, in LED dish

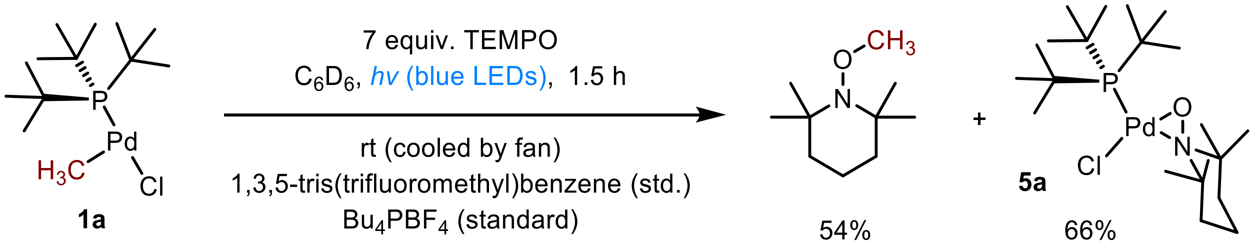

In a nitrogen-filled glovebox with the lights turned off, a solution of **1a** (4 mg, 11 µmol), TEMPO (12 mg, 78 µmol, 7 equiv.) and Bu_4PBF_4 (3.5 mg, 10 µmol, standard) in C_6D_6 (595 µL) was prepared. 1,3,5-tris(trifluoromethyl)benzene (5 µL, 27 µmol) was added as a standard.

This solution was transferred to an NMR tube and sealed with the cap, then sealed with electrical tape and aluminum foil around the outside in order to keep light out and exported from the glovebox. Initial ^{1}H and ^{31}P spectra were recorded, showing essentially no conversion of **1a** before irradiation. The tube was then placed in an LED dish, cooled with a fan. After 1.5 hours, the tube was removed and ^{1}H and ^{31}P NMR were recorded. Yields were determined by integration of ^{1}H and ^{31}P spectra compared to the initial spectra.

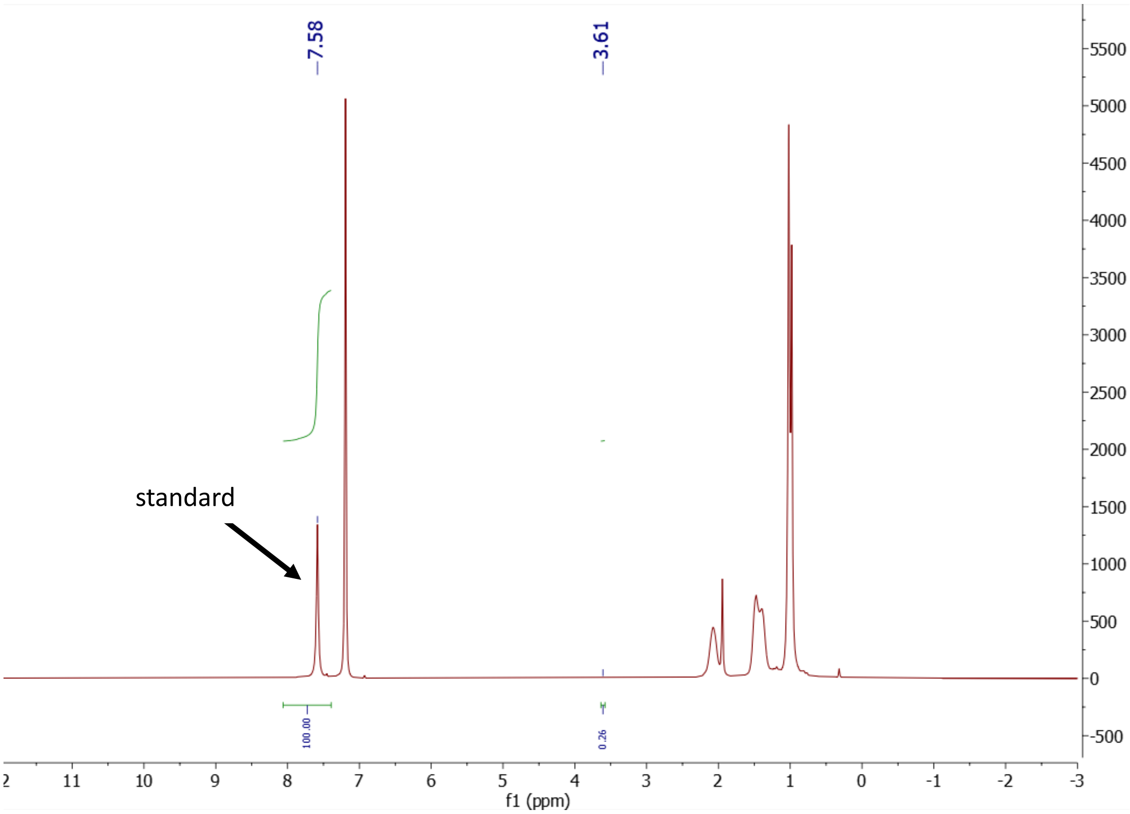

Figure S48. Initial ^{1}H spectrum before irradiation.

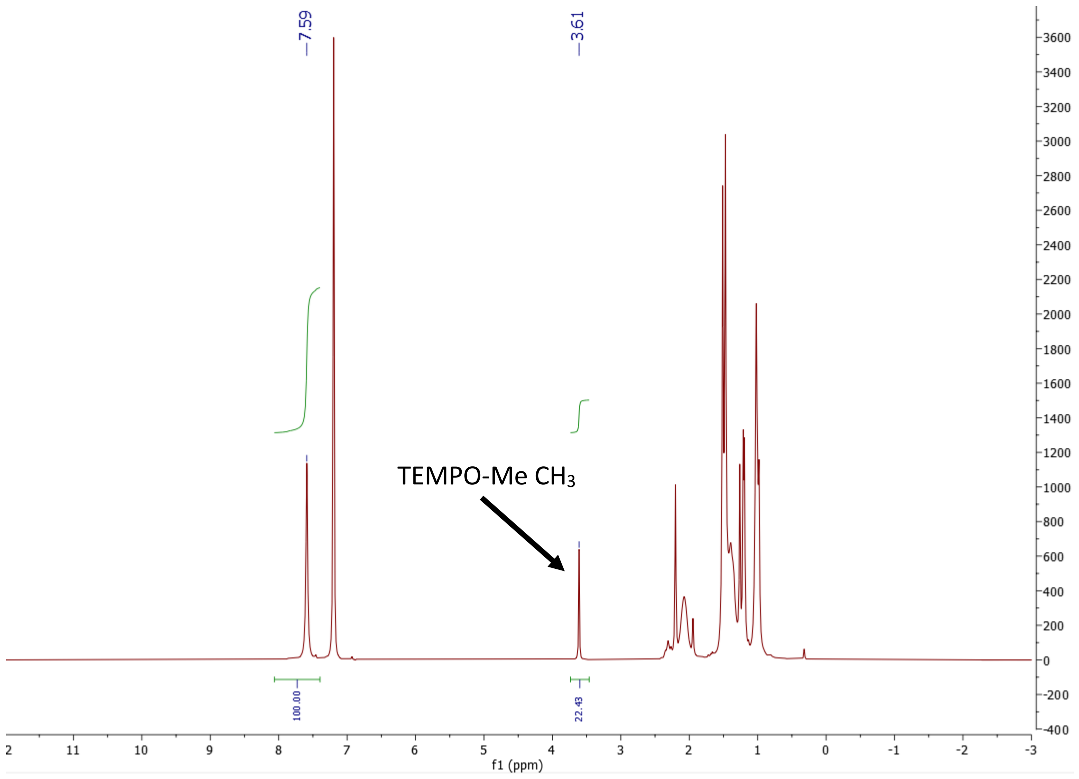

Figure S49. Final ^{1}H spectrum.

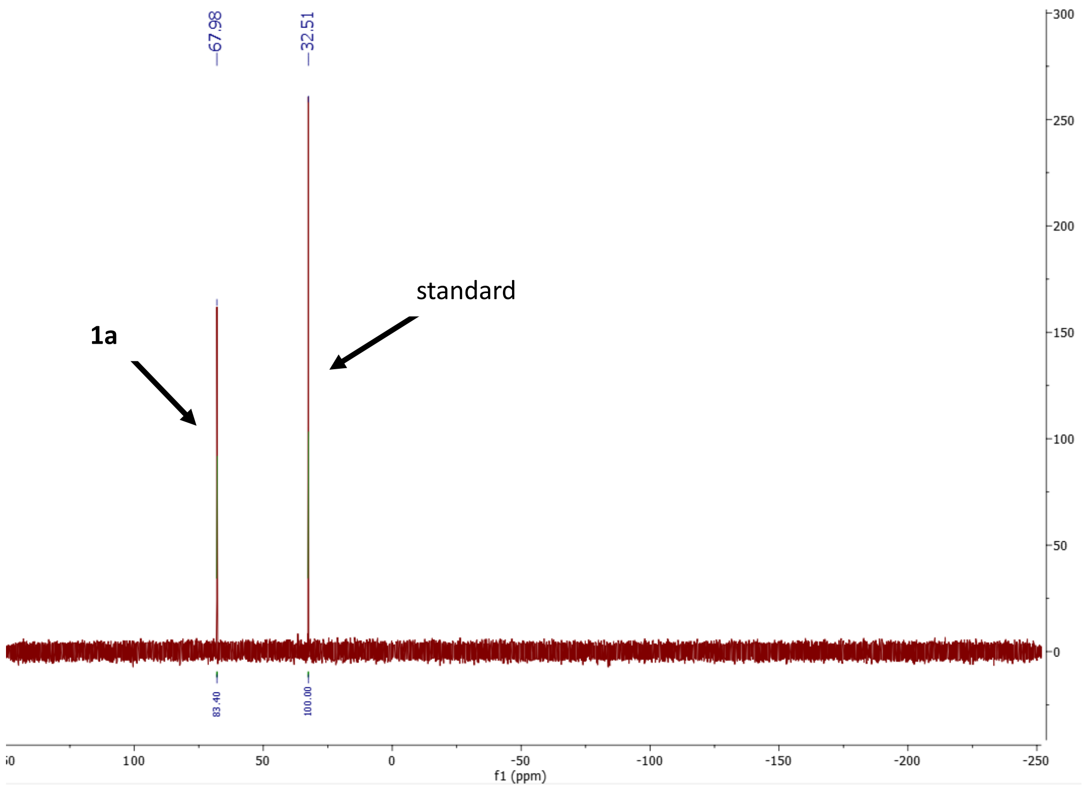

Figure S50. Initial ^{31}P spectrum.

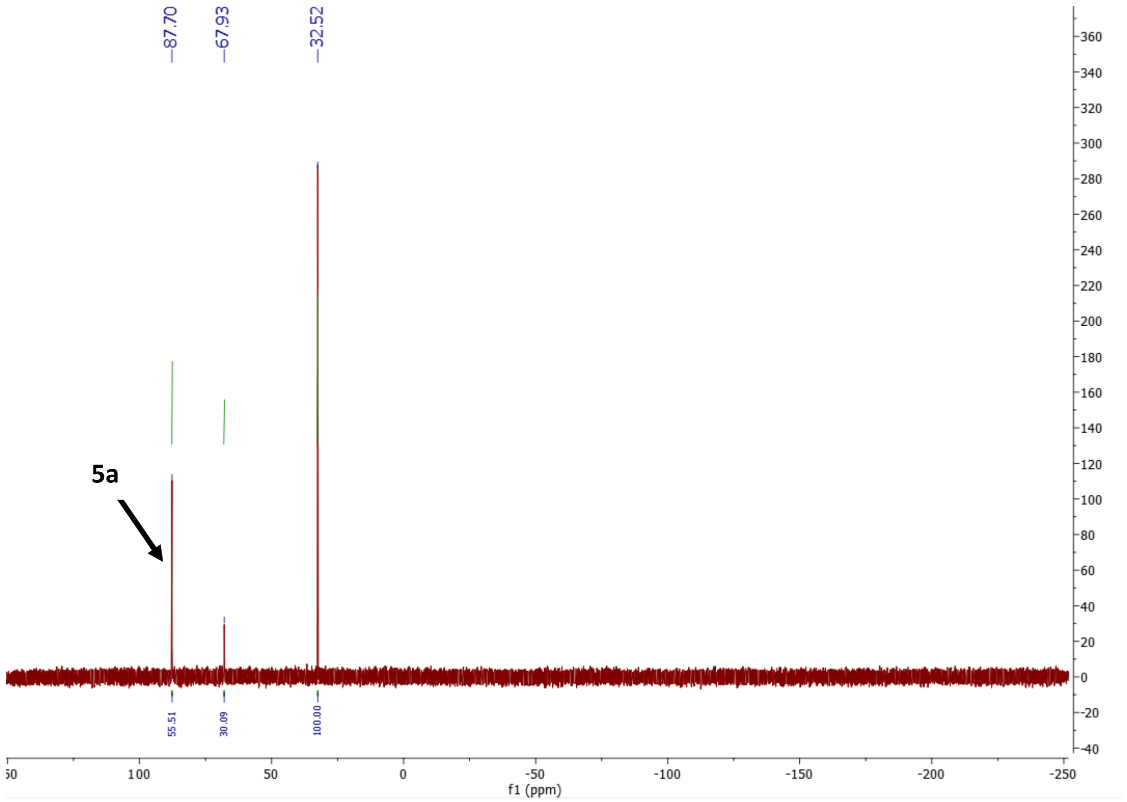

Figure S51. Final ^{31}P spectrum.

Light on-off test of conversion vs. irradiation time for 1a

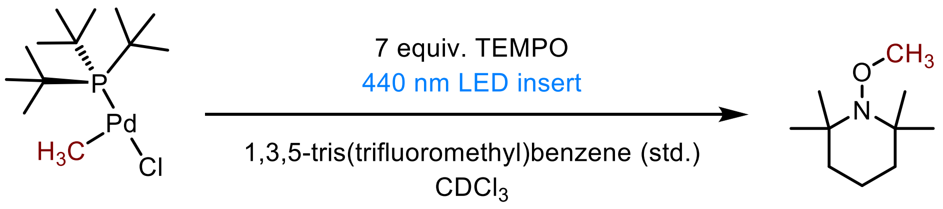

In a nitrogen-filled glovebox, a solution of **1a** (4 mg, 11.1 µmol) and TEMPO (12 mg, 78 µmol, 7 equiv.) in $CDCl_3$ (490 µL) was prepared. 1,3,5-tris(trifluoromethyl)benzene (10 µL, 53 µmol) was added as a standard. This solution was transferred to an NMR tube, a coaxial insert was inserted,[11] and the tube was sealed first with electrical tape and then parafilm. The tube was exported from the glovebox, the fiber optic cable was inserted, and the sample was placed in the spectrometer. An initial dark ^{1}H spectrum was recorded, then ^{1}H NMR spectra began to be continuously recorded. After several dark spectra were recorded, the light source was turned on (set to 5.0 of a maximum of 10) and spectra were continuously recorded for the duration of

the experiment by the steady state technique. Conversion was assessed by ^{1}H NMR. Note that convolution with other peaks reduces the accuracy of the integration of the Pd-CH_3 peak of **1a**.

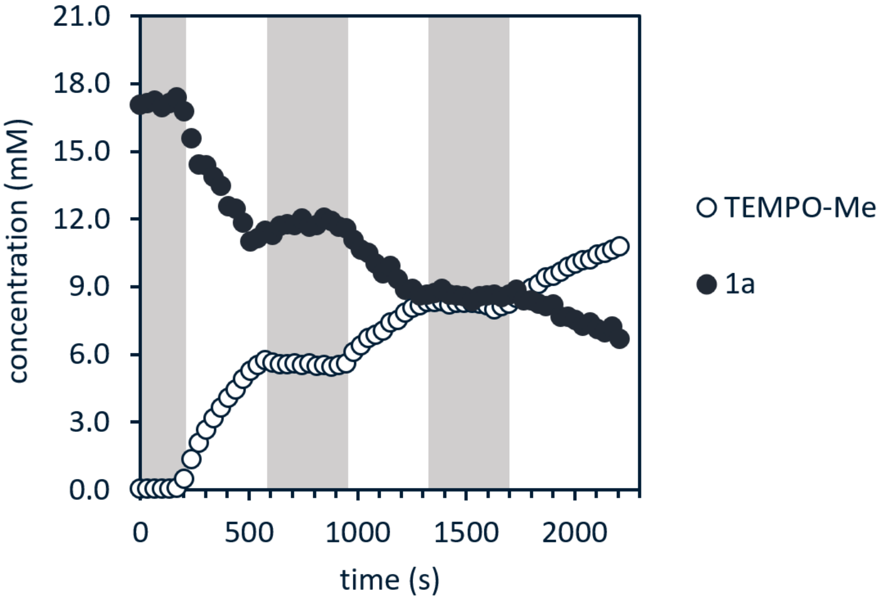

Figure S52. Light on-off study for **1a** in the presence of TEMPO.

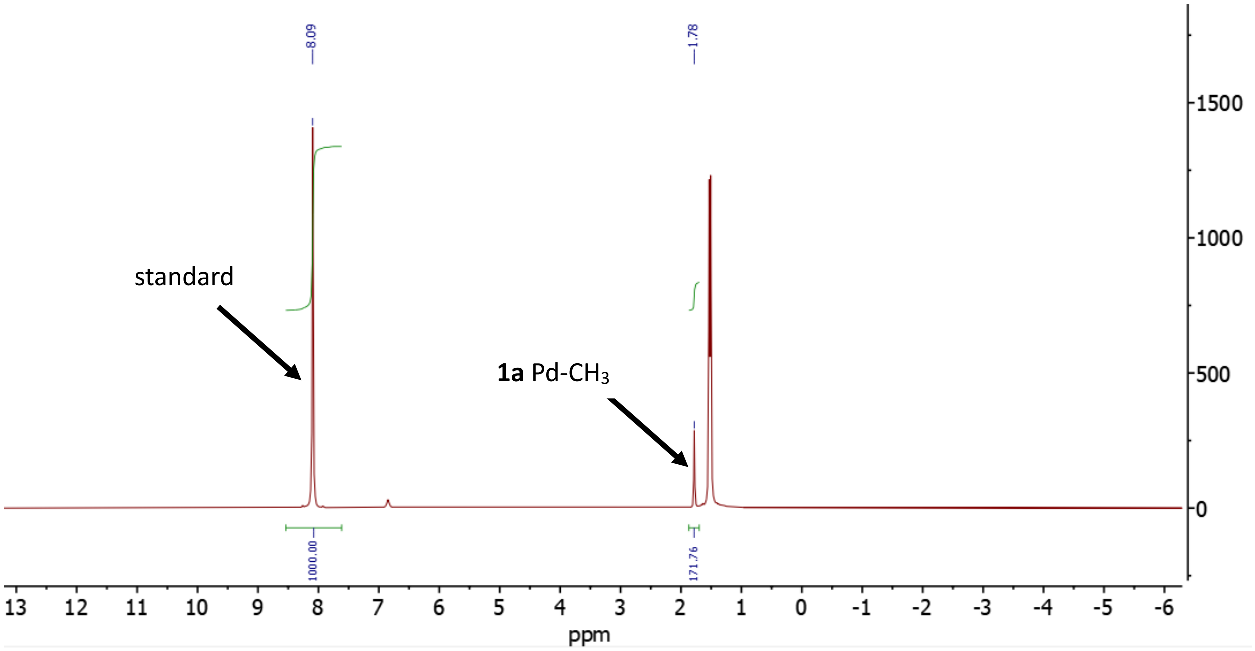

Figure S53. Initial ^{1}H spectrum before irradiation.

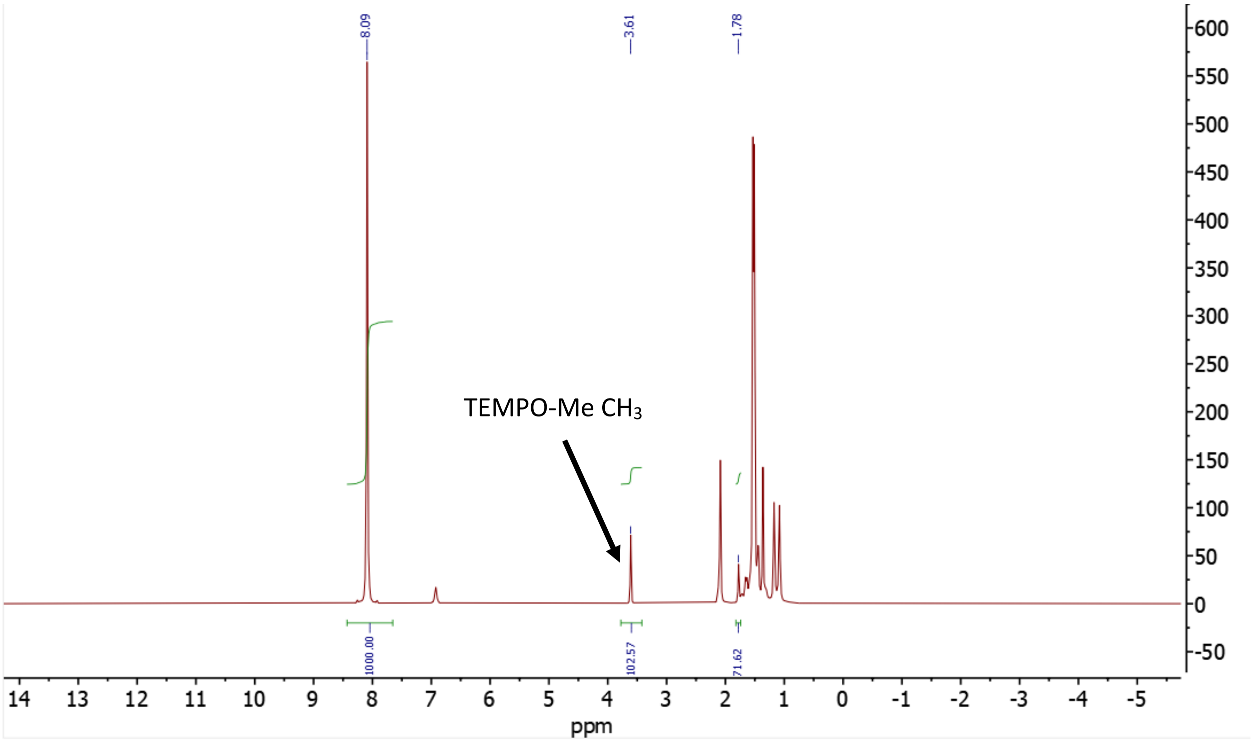

Figure S54. Final ^{1}H spectrum.

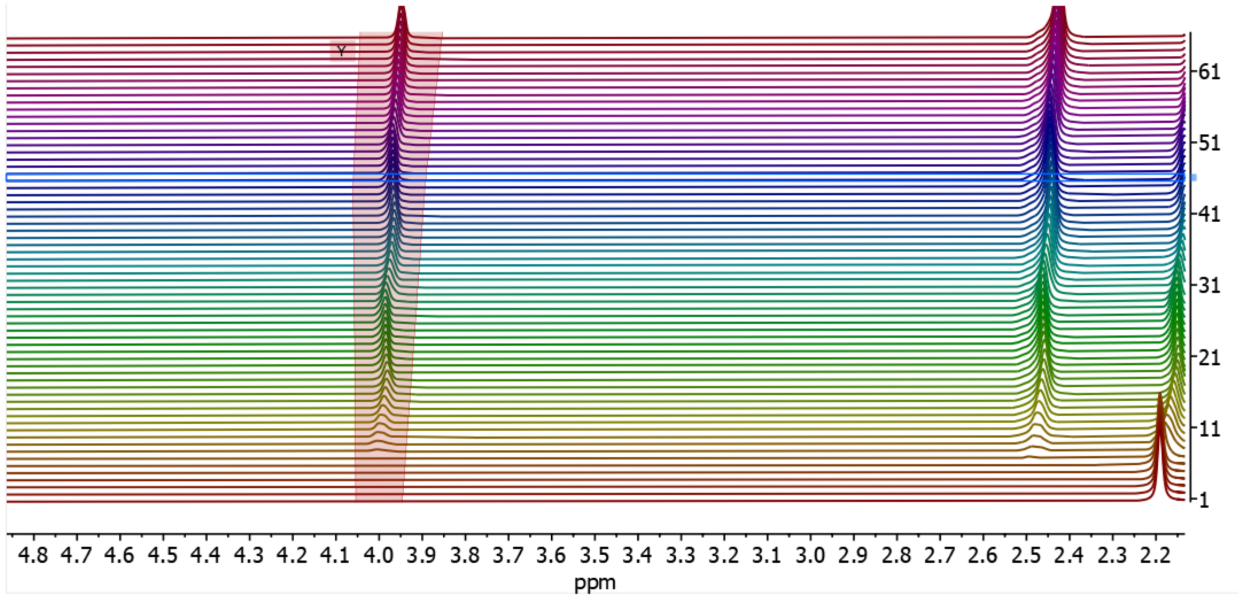

Figure S55. Stack of ^{1}H spectra showing TEMPO-Me peak (note: the chemical shift of these spectra has not been corrected, the highlighted peak should appear at 3.61 ppm).

Light on-off test of conversion vs. irradiation time for 2

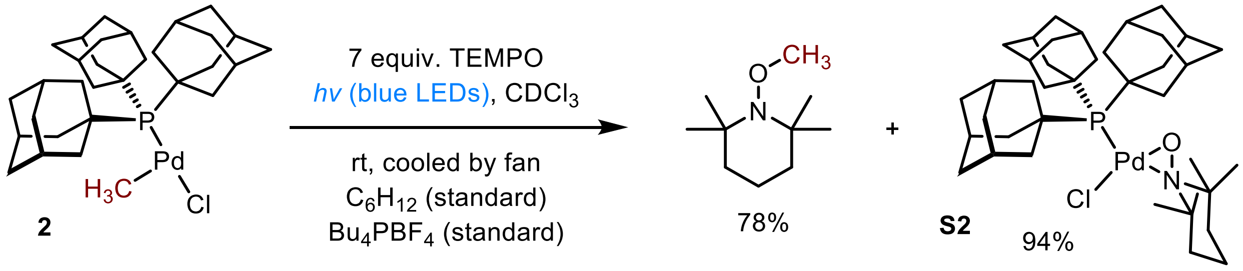

In a nitrogen-filled glovebox, a solution of **2** (4 mg, 6.7 µmol), TEMPO (7 mg, 47 µmol, 7 equiv.) and Bu_4PBF_4 (2.8 mg, 8 µmol, standard) in $CDCl_3$ (756 uL) was prepared. Cyclohexane (4 µL, 37 µmol) was added as a standard. This solution was transferred to an NMR tube and sealed with the cap, then sealed with electrical tape and exported from the glovebox. Initial ^{1}H and ^{31}P spectra were recorded, showing essentially no conversion of **2** before irradiation. The tube was then placed in an LED dish about 2 cm from the LEDs. A fan was placed above the dish and used to cool the reaction mixture. After the desired time, the tube was removed from the LED dish and ^{1}H and ^{31}P NMR were recorded. Conversion and yields of product were determined by integration of the ^{1}H NMR spectrum, comparing to the initial ^{1}H NMR spectrum. No significant change in conversion was observed when the tube was not in the LED dish.

Table S1. Conversion of **2** and yield of TEMPO-Me over time. Note that the time represents the amount of time that the reaction mixture was under blue LED irradiation, and that, as shown in **Figure S56**, there was about a 12 hour period where the reaction mixture was not irradiated after the initial 40 minutes of irradiation.

**total time irradiated (h)**	**conversion of 2 (%)**	**yield of TEMPO-Me (%)**
0	0	0
40	58	43
90	73	59
150	84	66
210	89	71

300	95	78

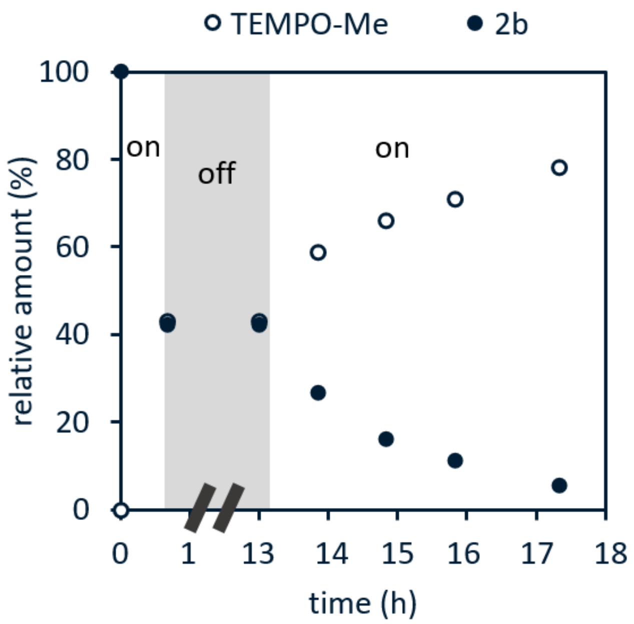

Figure S56. Light on-off study for **2** in the presence of TEMPO. Note there was a ca. 12 hour period where the reaction mixture was not irradiated after the initial 40 minutes of irradiation.

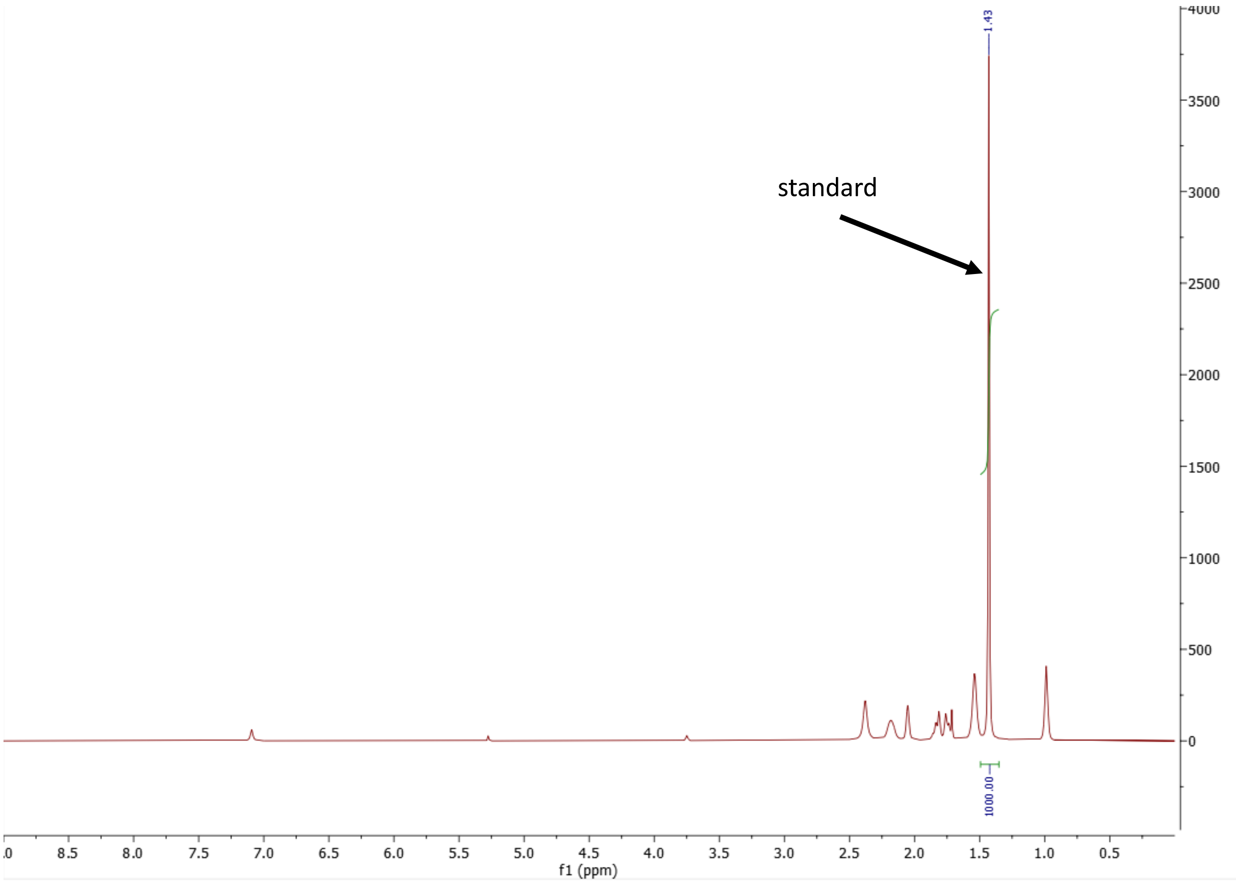

Figure S57. Initial ^{1}H spectrum before irradiation.

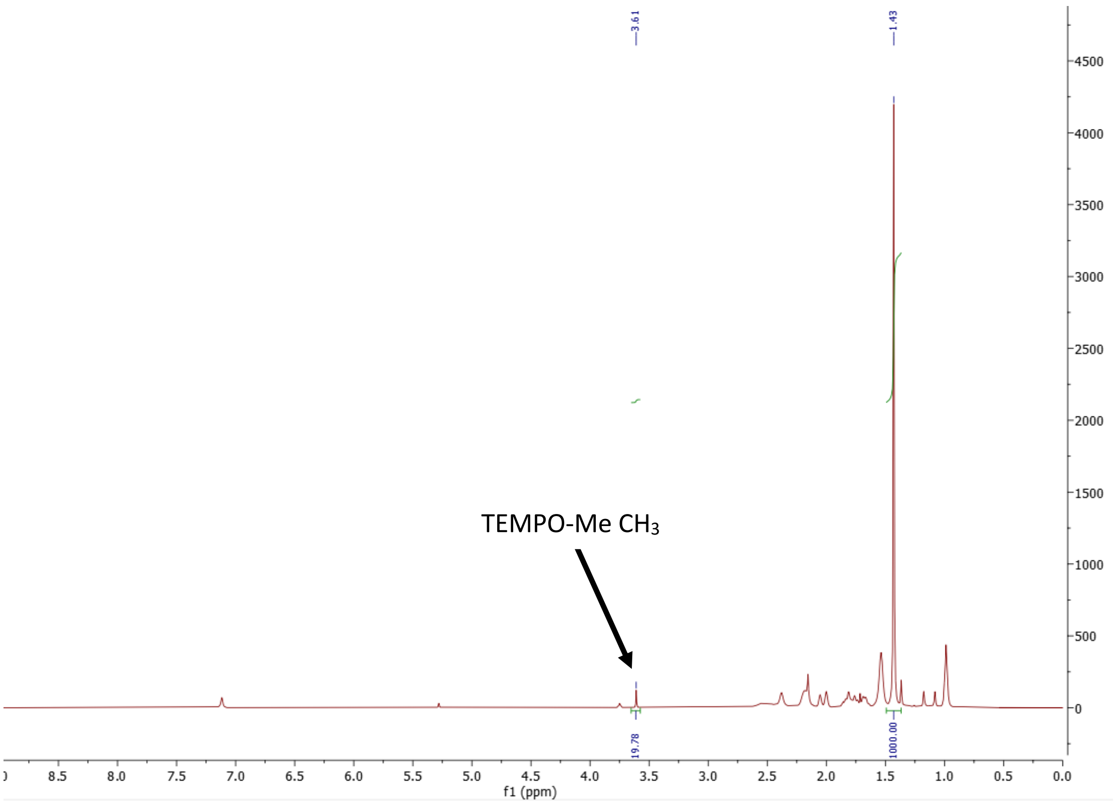

Figure S58. ^{1}H spectrum after 40 minutes of irradiation.

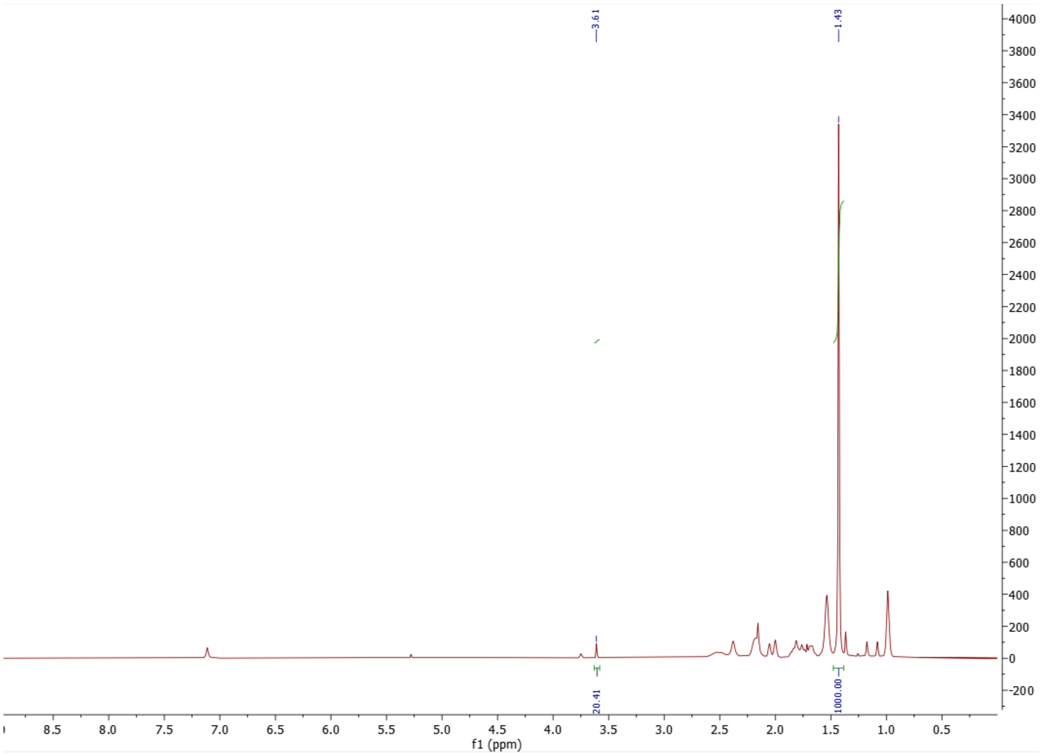

Figure S59. ^{1}H spectrum after 40 minutes of irradiation and 13 subsequent hours in the dark.

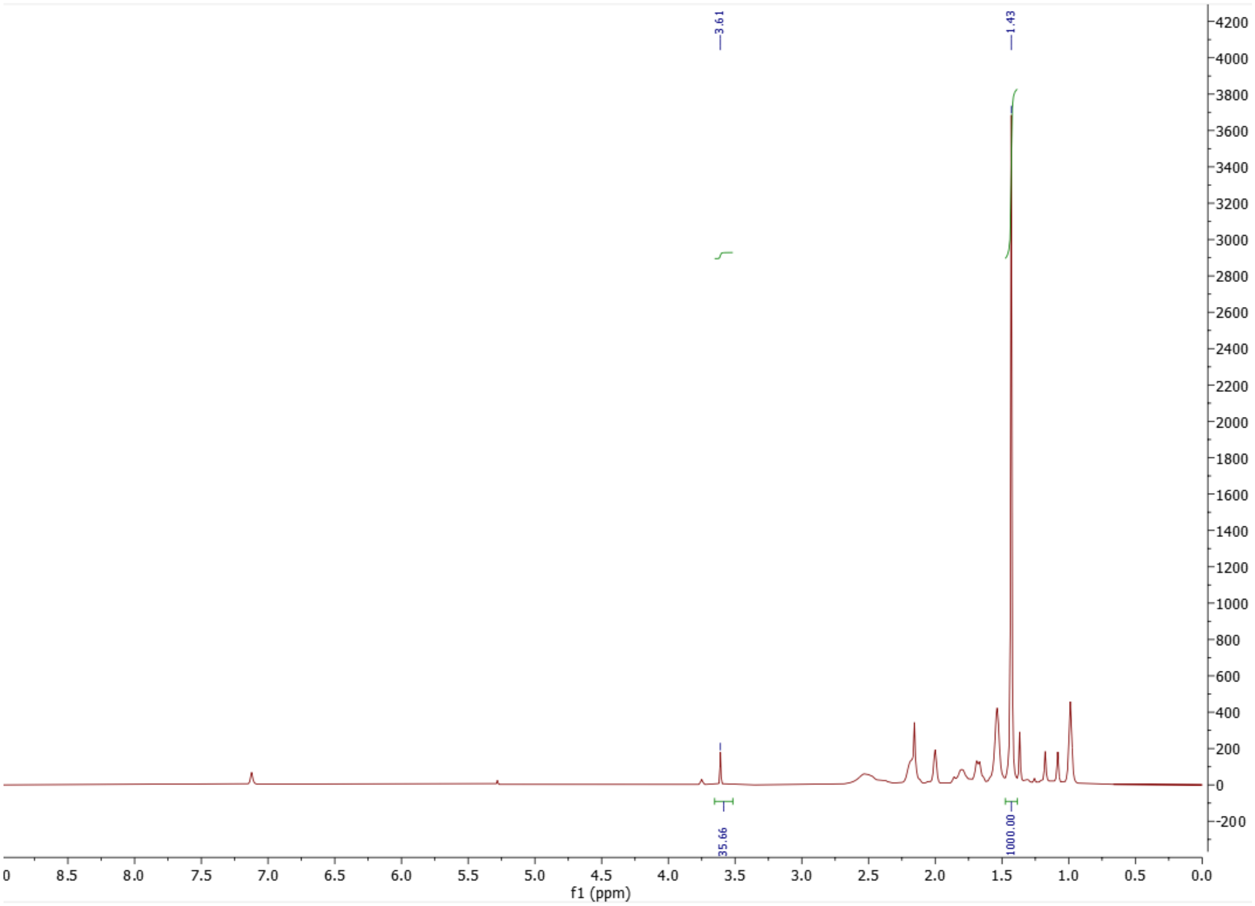

Figure S60. ^{1}H spectrum after 5 hours of irradiation total (final data point).

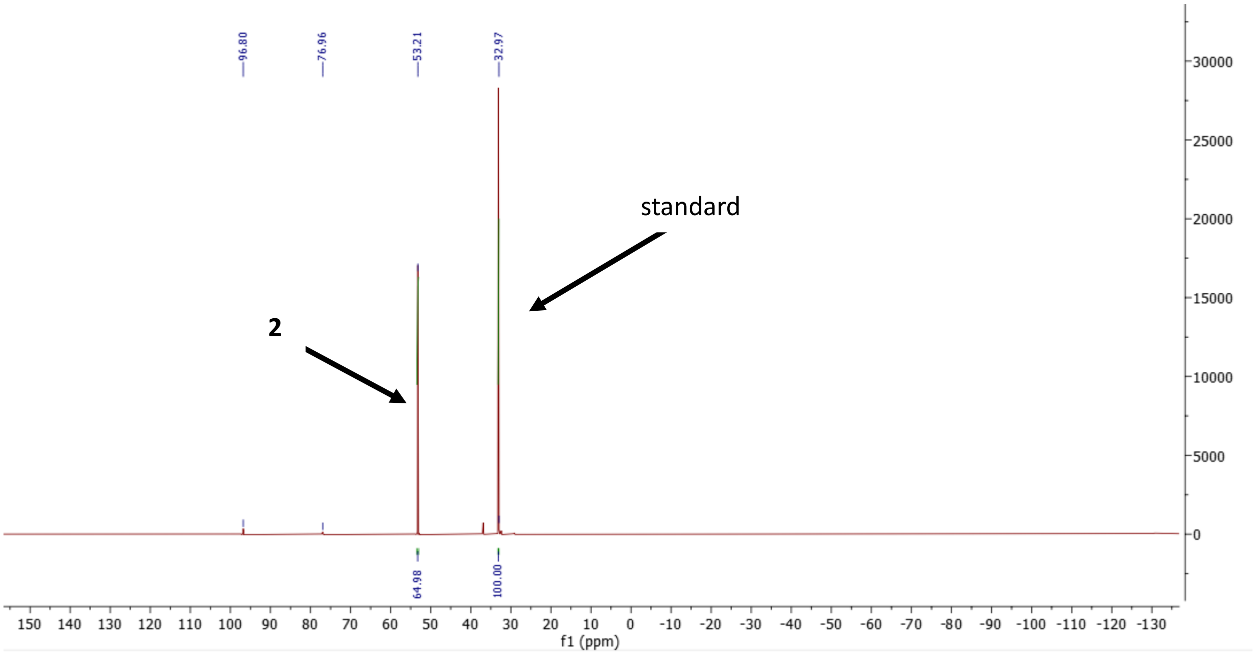

Figure S61. Initial ^{31}P spectrum before irradiation.

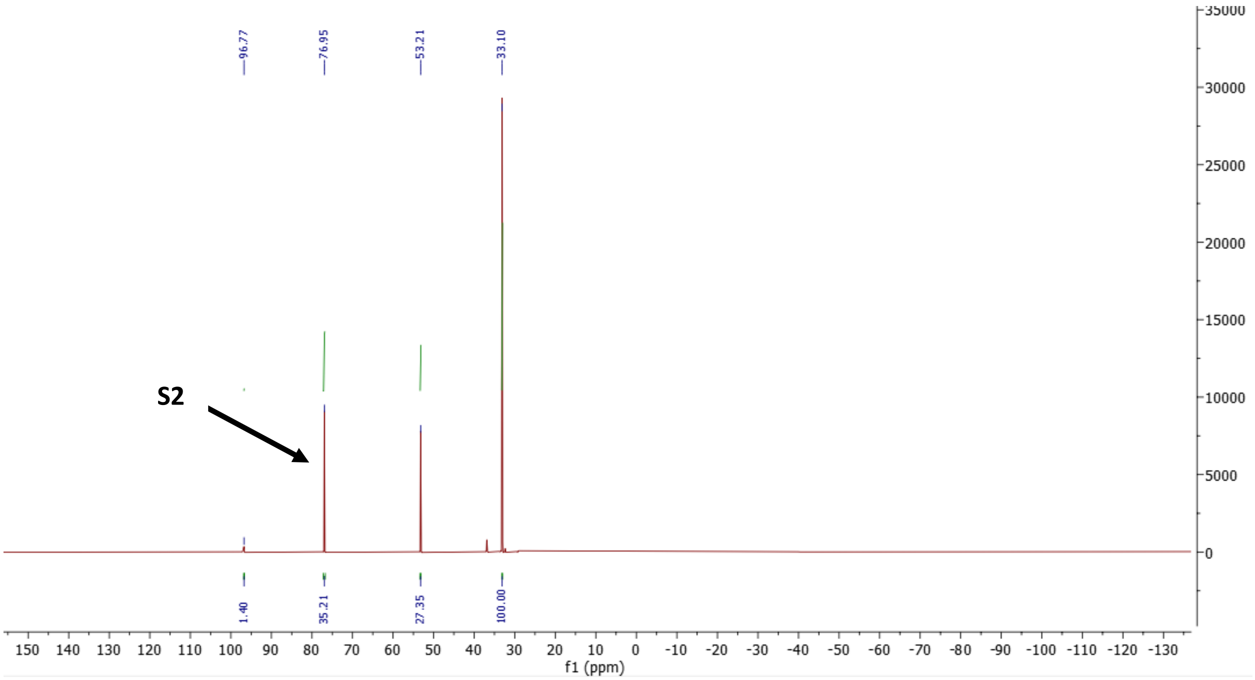

Figure S62. ^{31}P spectrum after 40 minutes of irradiation.

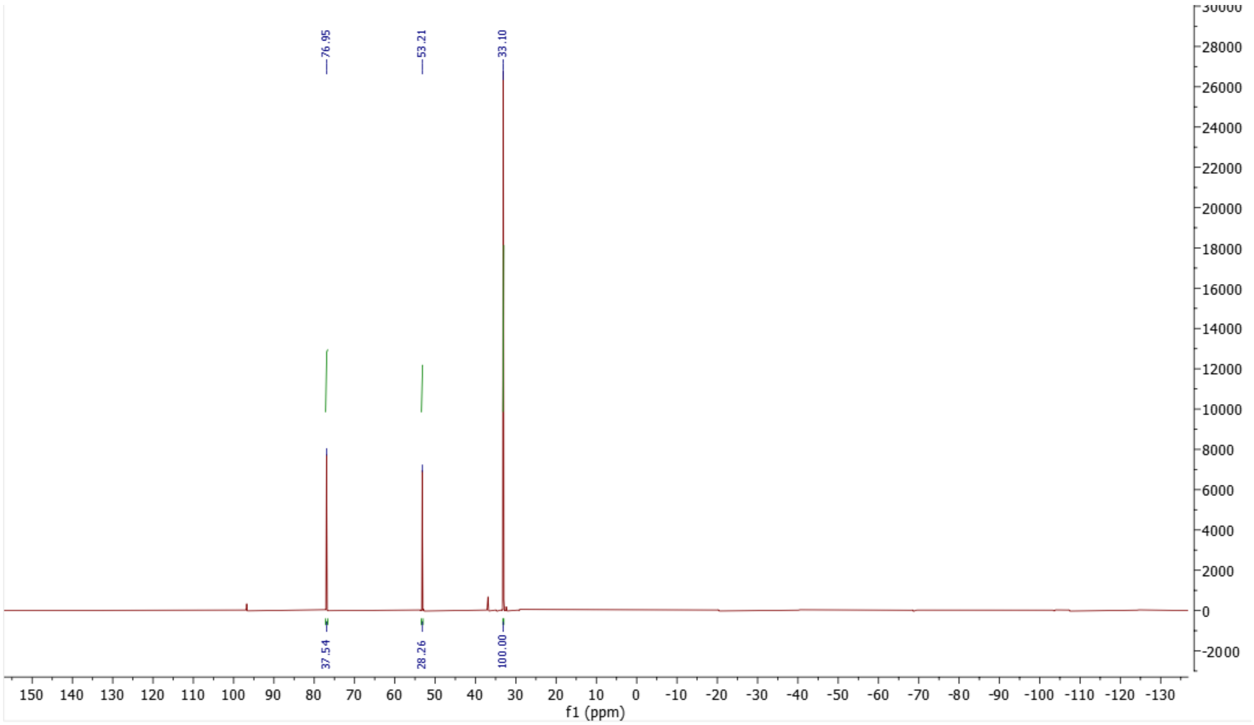

Figure S63. ^{31}P spectrum after 40 minutes of irradiation and 13 subsequent hours in the dark.

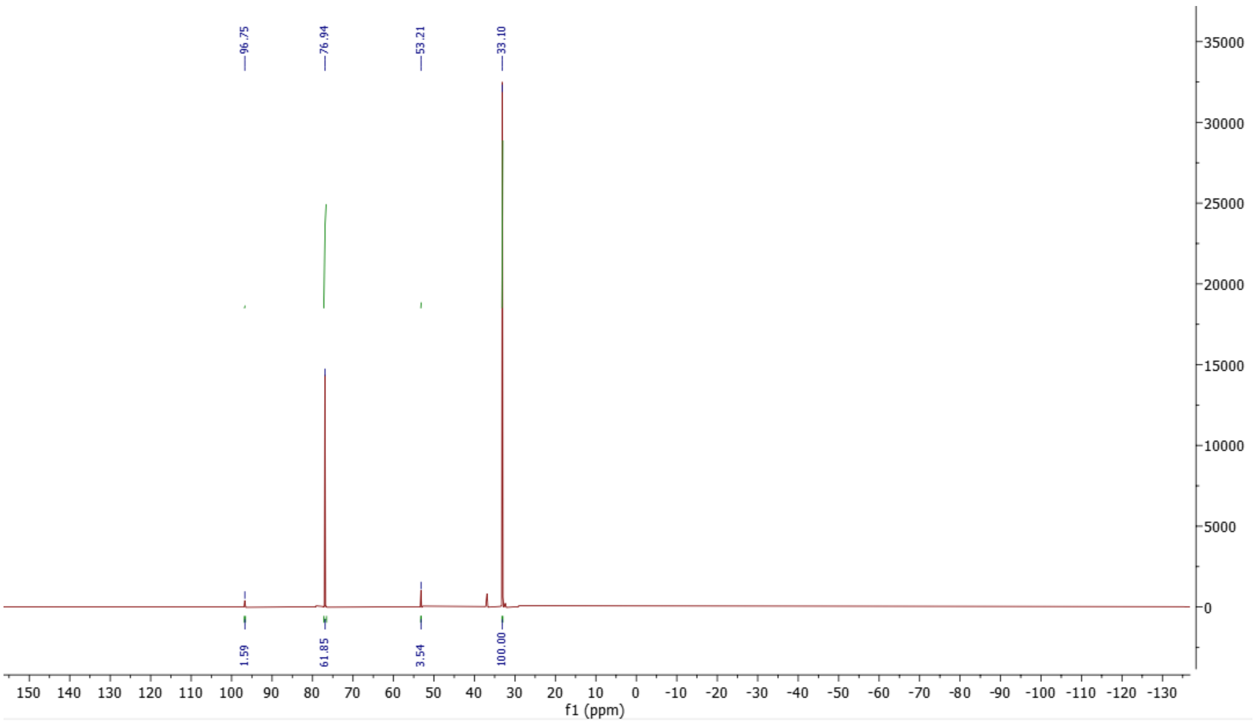

Figure S64. ^{31}P spectrum after 5 total hours of irradiation (final data point)

Irradiation of 1b with blue LEDs in THF in the presence of excess TEMPO: spiking reaction mixture with (*t*-Bu$_3$P)$_2$Pd(0) and 5a.

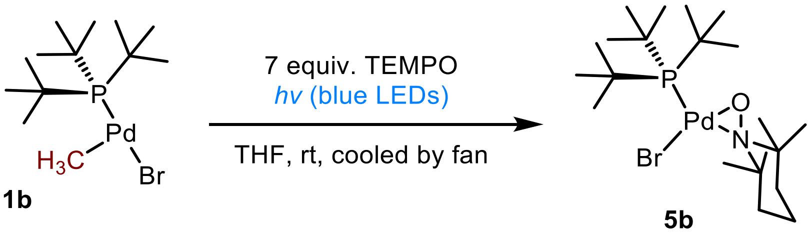

In a nitrogen-filled glovebox, a solution of **1b** (4 mg, 12 µmol) and TEMPO (12 mg, 78 µmol, 7 equiv.) in THF (600 uL) was prepared. This solution was transferred to an NMR tube and sealed with the cap, then sealed with electrical tape and exported from the glovebox. An initial ^{31}P spectrum was recorded, showing essentially no conversion of **1b** before irradiation. The tube was then placed in an LED dish about 2 cm from the LEDs. A fan was placed above the dish and used to cool the reaction mixture. After 2 hours, the tube was removed from the LED dish and ^{31}P NMR was recorded. The formation of **5b** was observed by ^{31}P NMR. A yield was not calculated in this case because no internal standard was present. The reaction mixture was

then spiked with a separately prepared sample of (*t*-$Bu_3P)_2Pd(0)$ as well as a separate, similar reaction mixture containing a sample of 5a that had been generated *in situ*. A ^{31}P NMR spectrum of the resulting mixture showed separate peaks for **5a**, **5b** and (*t*-$Bu_3P)_2Pd(0)$, which confirmed that all three compounds are distinct from one another.

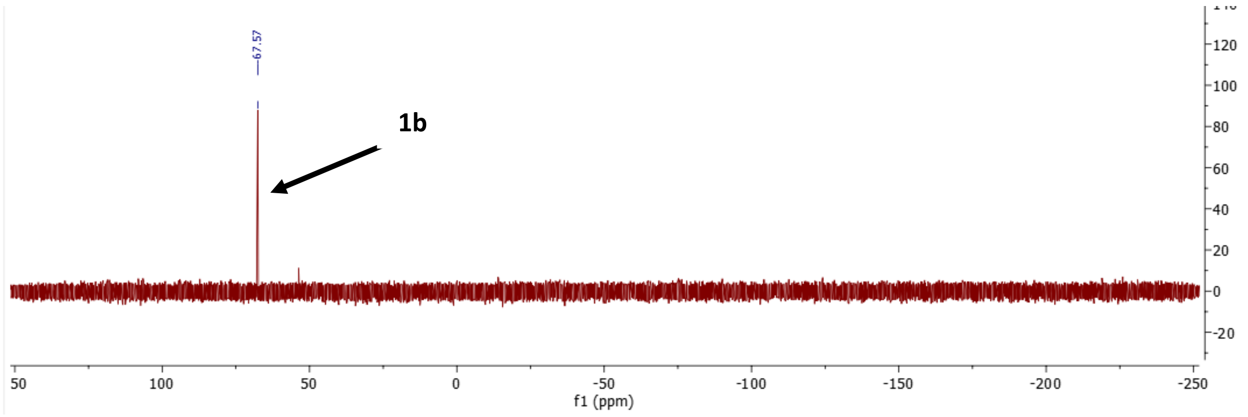

Figure S65. Initial ^{31}P spectrum before irradiation.

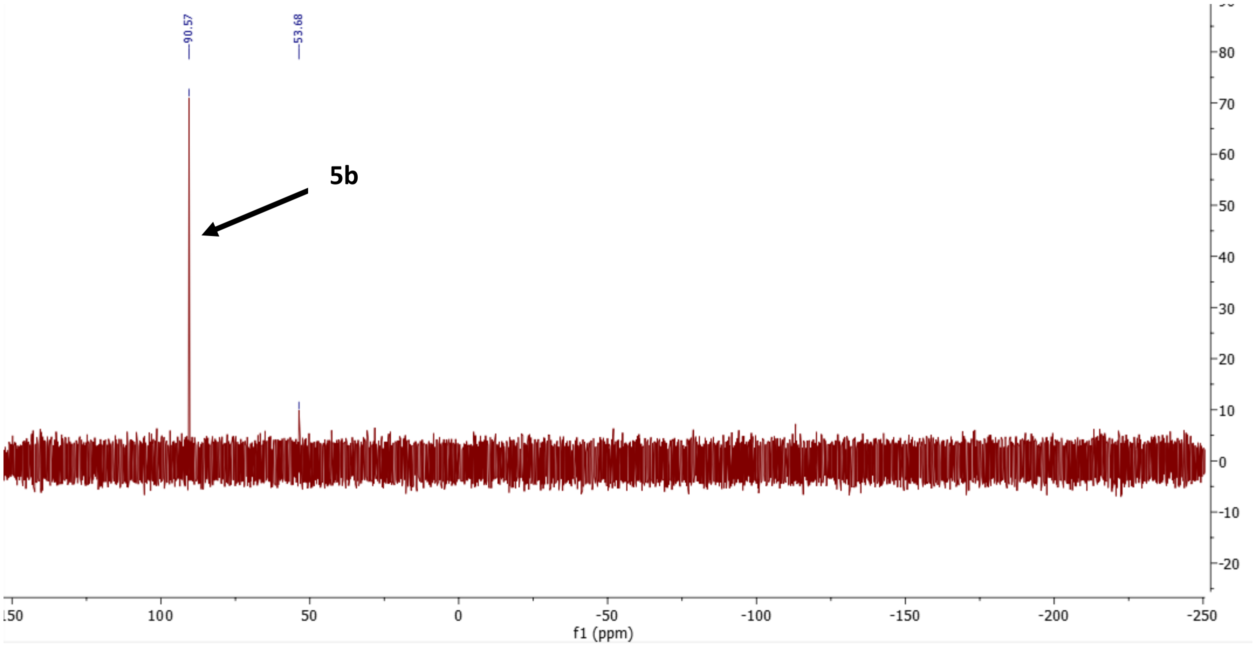

Figure S66. Final ^{31}P spectrum.

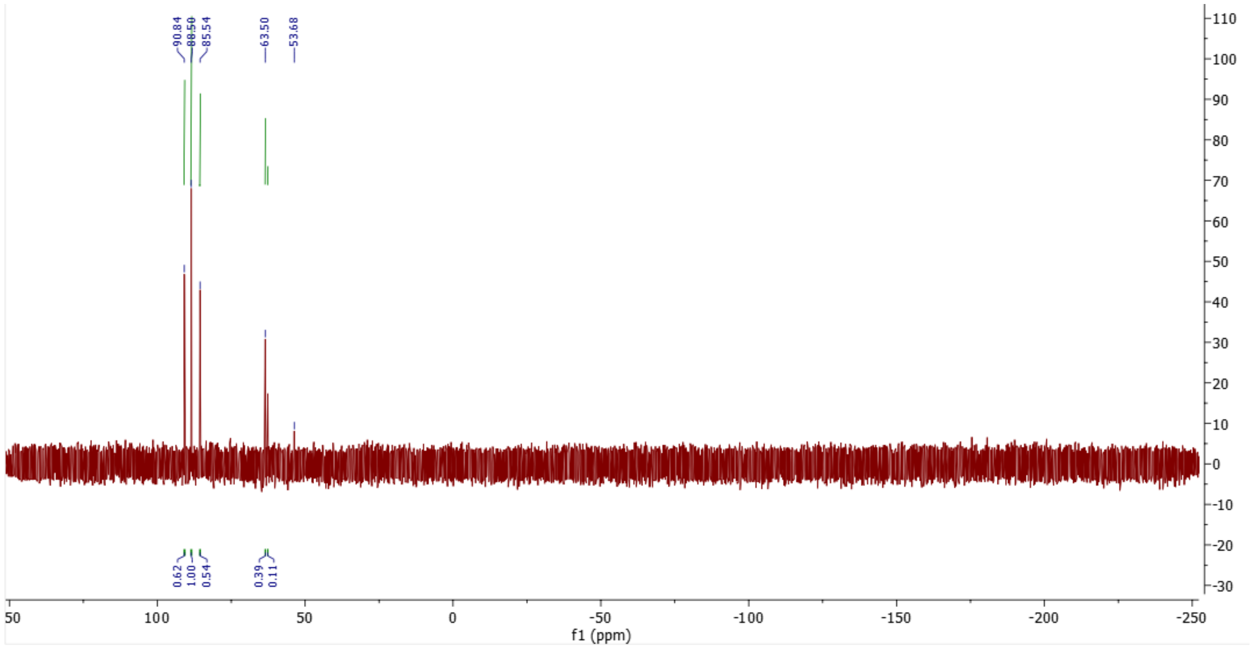

Figure S67. Spiked reaction mixture including **5a** and ((t-Bu)$_3$P)$_2$Pd(0).

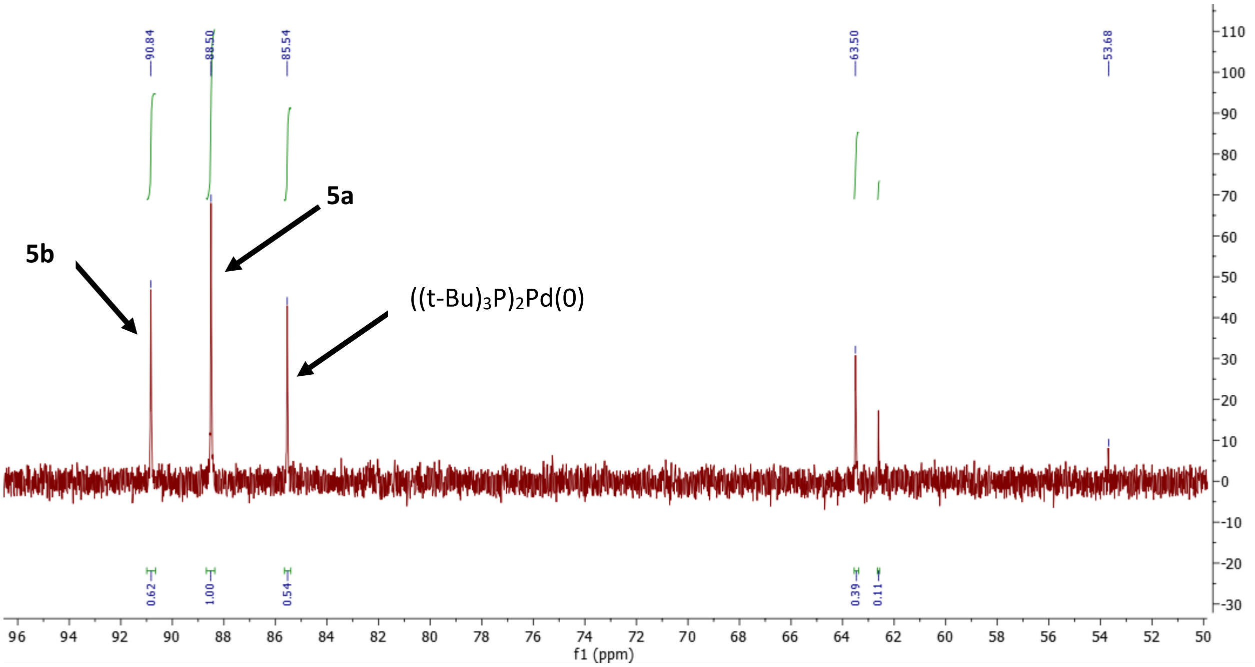

Figure S68. Spiked reaction mixture including **5a** and ((t-Bu)$_3$P)$_2$Pd(0) (detail).

Irradiation of 1b with blue LEDs in $CDCl_3$ without TEMPO: observation of the formation of a Pd(I) product

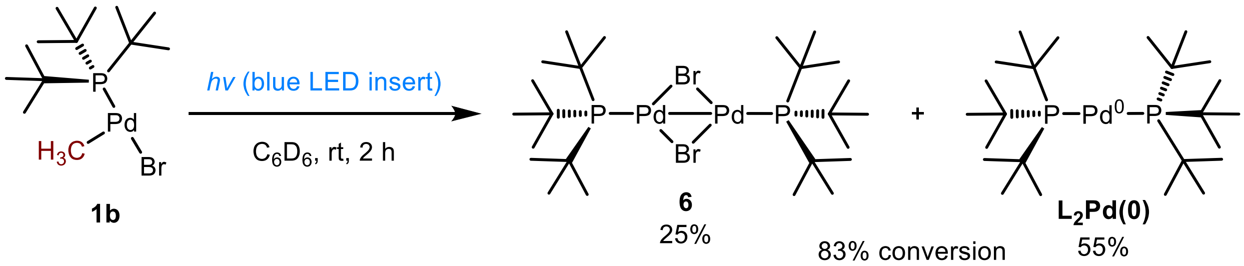

In a nitrogen-filled glovebox, a solution of **1b** (1 mg, 2.5 μmol) in C_6D_6 (500 μL) was prepared by used of a stock solution. This solution was transferred to a specialized J Young NMR tube and sealed with a cap that contained a glass insert,[11] then sealed with additional parafilm and exported from the glovebox. A fiber optic cable was inserted and then the tube was placed into the NMR instrument. An initial ^{31}P spectrum was recorded, showing essentially no conversion of **1b** before irradiation. The light source was then turned on and ^{31}P NMR spectra were recorded periodically. After 2 hours of irradiation, ^{31}P NMR indicated about 83% conversion of **1b** took place, and 25% yield of **6** as well as 55% yield of $L_2Pd(0)$ (conversion/yields were calculated by integration relative to the initial spectrum. These yields/conversions are approximate numbers; an internal standard was not used in order to prevent the formation of side products as much as possible.) **6** is the initial product of irradiation, while $L_2Pd(0)$ begins to appear as conversion increases.

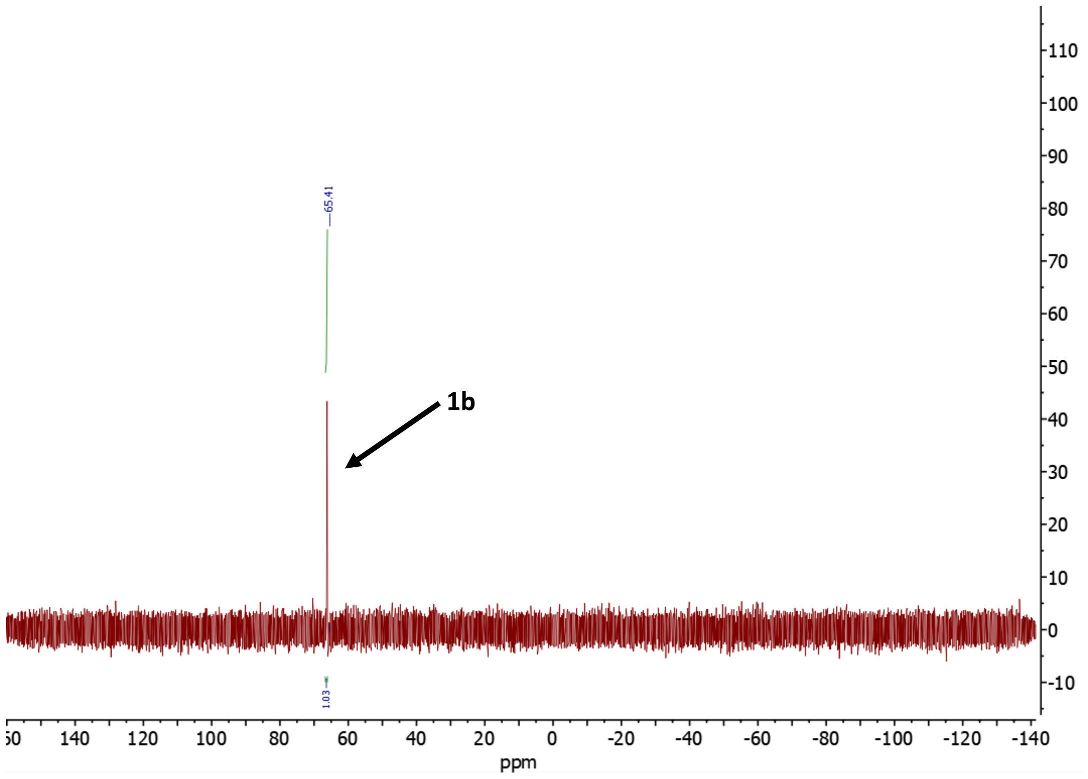

Figure S69. Initial ^{31}P spectrum before irradiation.

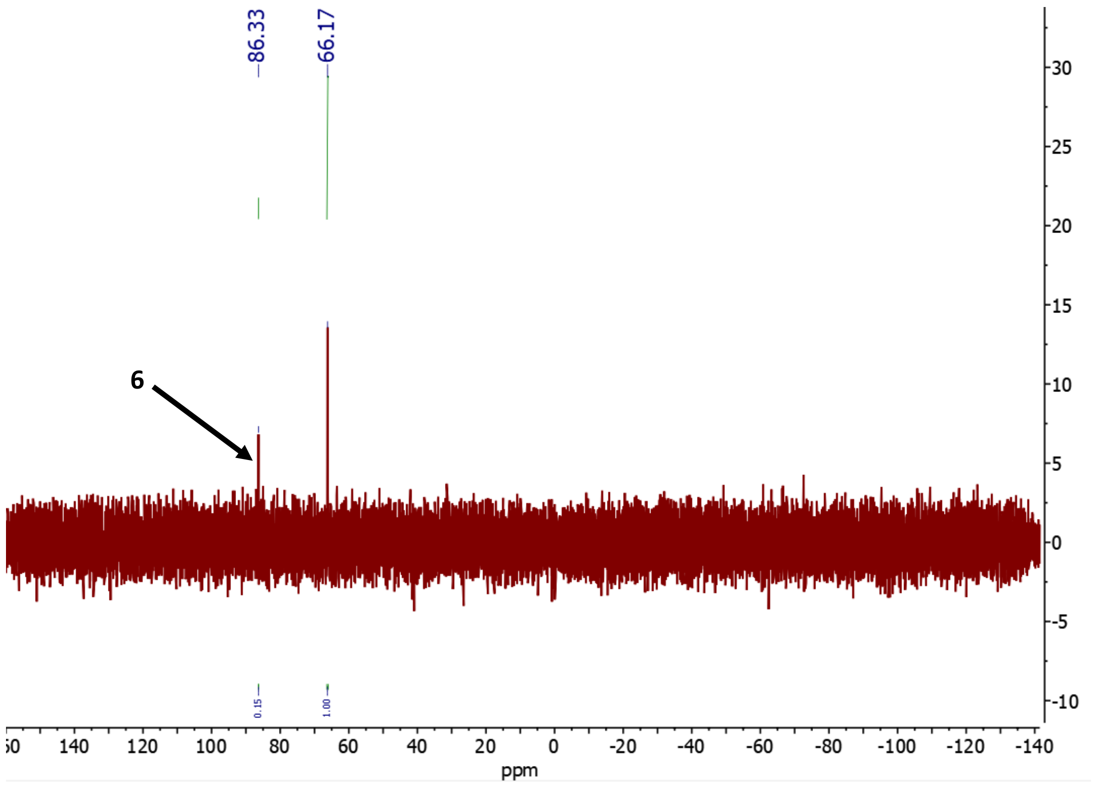

Figure S70. ^{31}P spectrum after 26 minutes of irradiation showing formation of **6** before appearance of $L_2Pd(0)$.

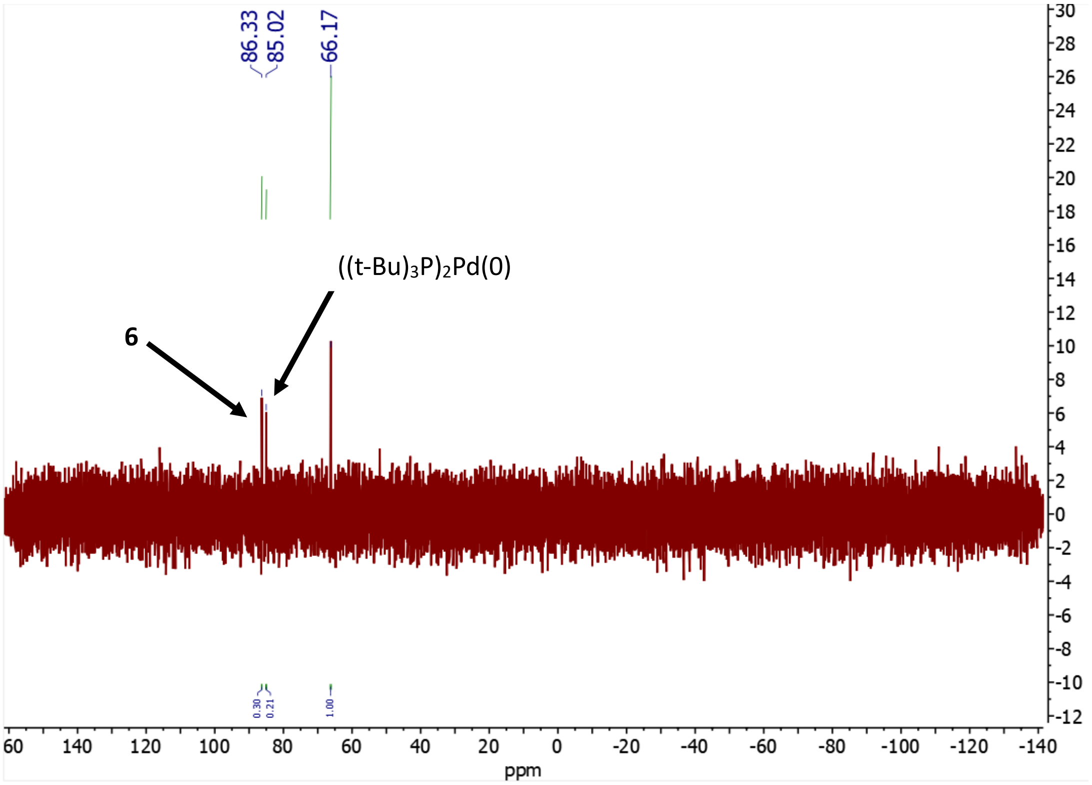

Figure S71. ^{31}P spectrum after 32 minutes of irradiation showing that $L_2Pd(0)$ is beginning to appear.

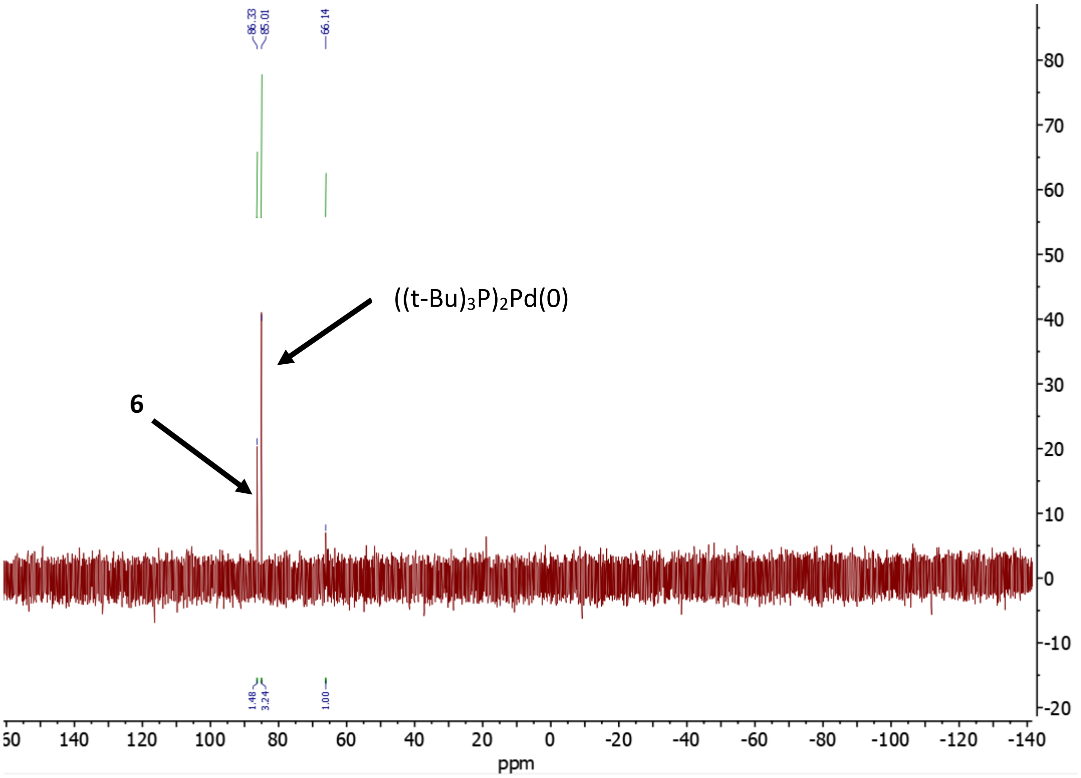

Figure S72. Final ^{31}P spectrum (2 h).

Irradiation of 4 with blue LEDs in $CDCl_3$ in the presence of excess TEMPO

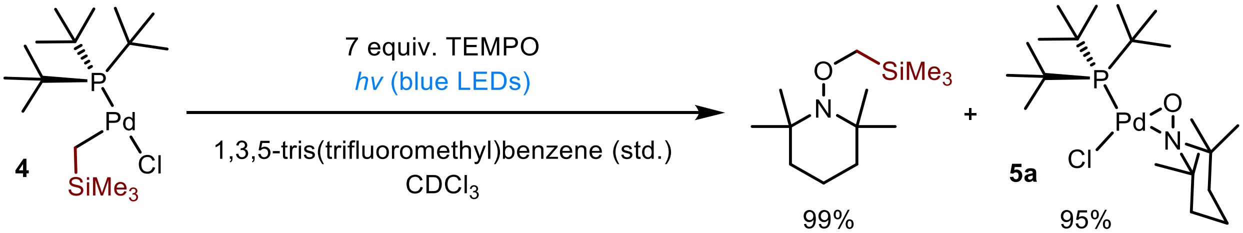

In a nitrogen-filled glovebox, a solution of **4** (4 mg, 9 µmol), TEMPO (10 mg, 65 µmol, 7 equiv.) and Bu_4PBF_4 (4 mg, 12 µmol, standard) in $CDCl_3$ (590 uL) was prepared. 1,3,5-tris(trifluoromethyl)benzene (10 µL, 45 µmol) was added as a standard. This solution was transferred to an NMR tube and sealed with the cap, then sealed with electrical tape and exported from the glovebox. Initial ^{1}H and ^{31}P spectra were recorded, showing essentially no conversion of **4** before irradiation. The tube was then placed in an LED dish about 2 cm from the LEDs. A fan was placed above the dish and used to cool the reaction mixture. After 21 hours, the tube was removed from the LED dish and ^{1}H and ^{31}P NMR were recorded. Conversion and yields of product were determined by integration of the ^{1}H NMR spectrum,

comparing to the initial ^{1}H NMR spectrum. The product matched the previously-reported spectrum in the literature for TEMPO-neosilyl.[12]

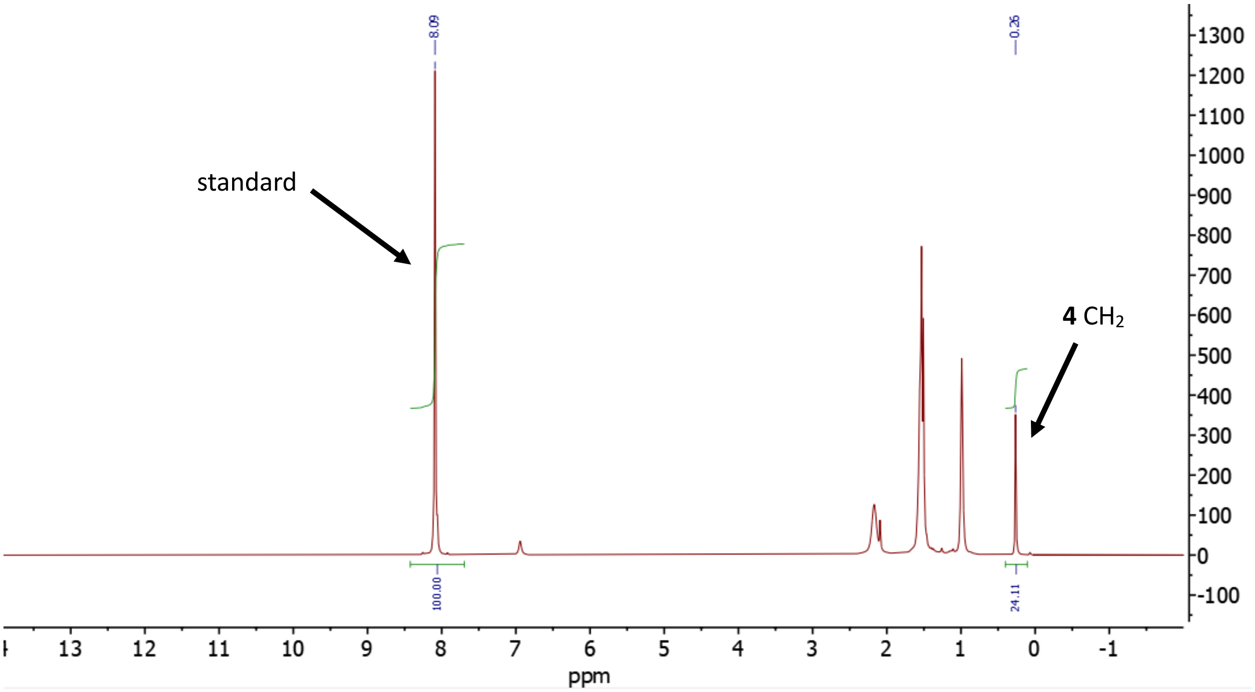

Figure S73. Initial ^{1}H spectrum before irradiation.

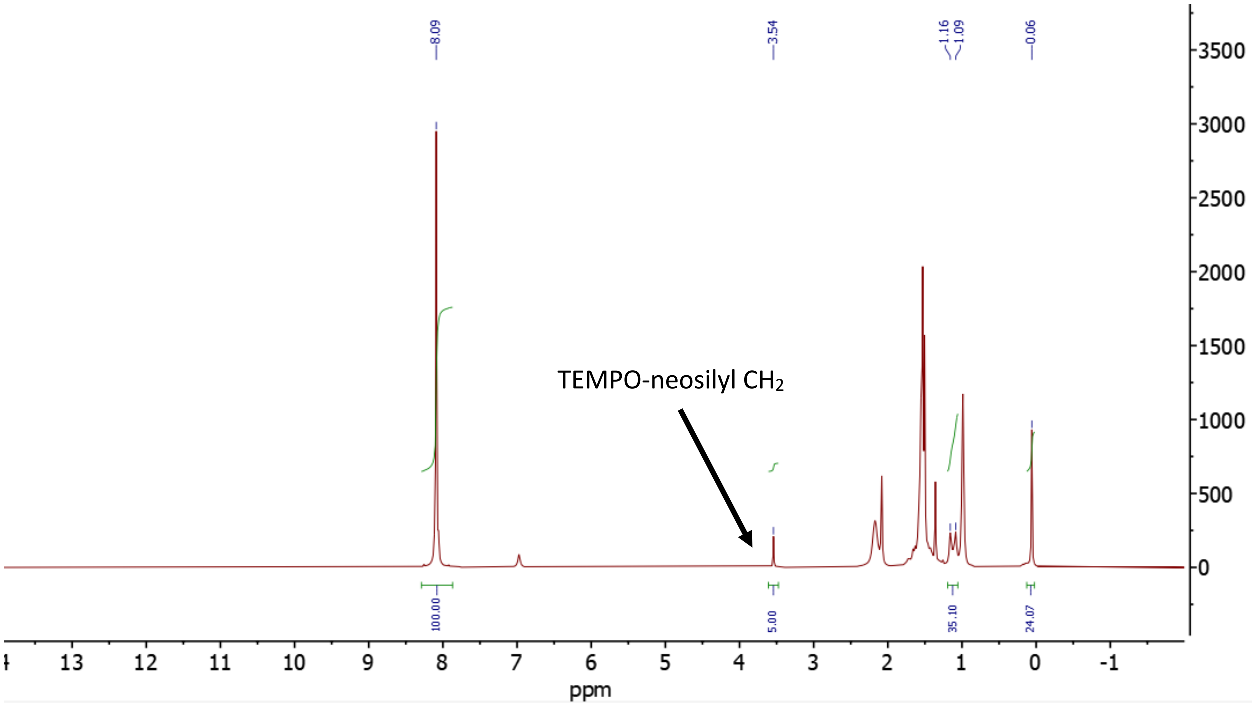

Figure S74. Final ^{1}H spectrum.

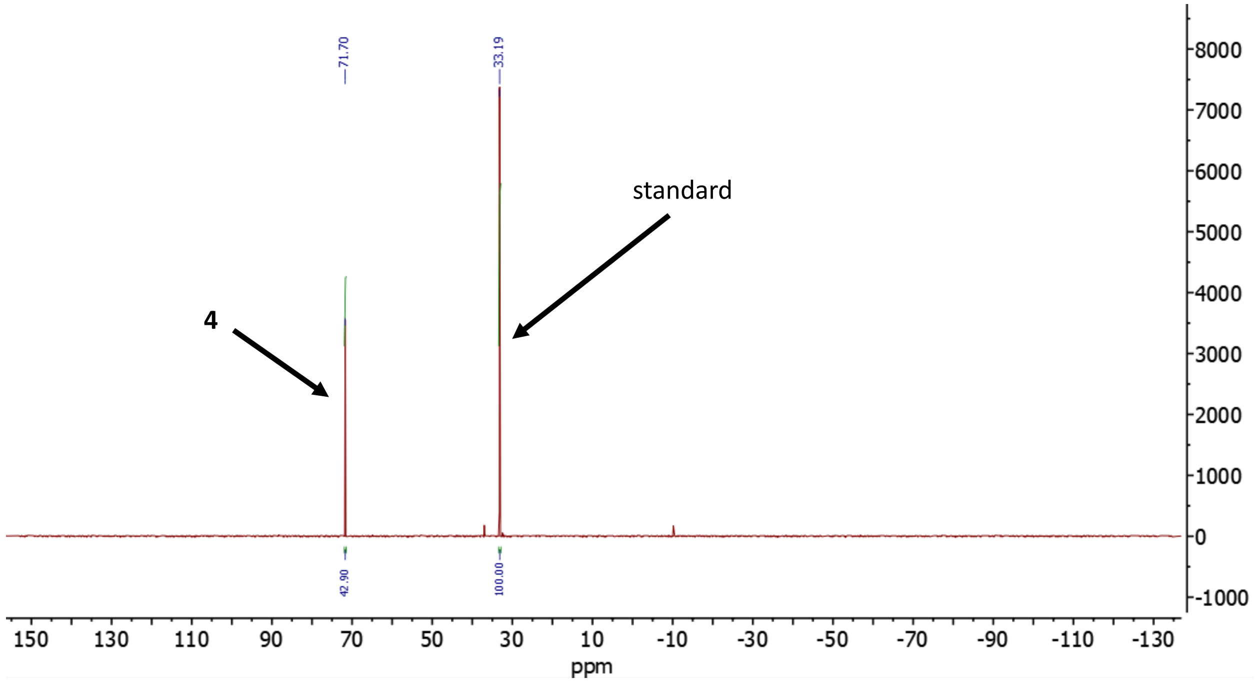

Figure S75. Initial ^{31}P spectrum before irradiation.

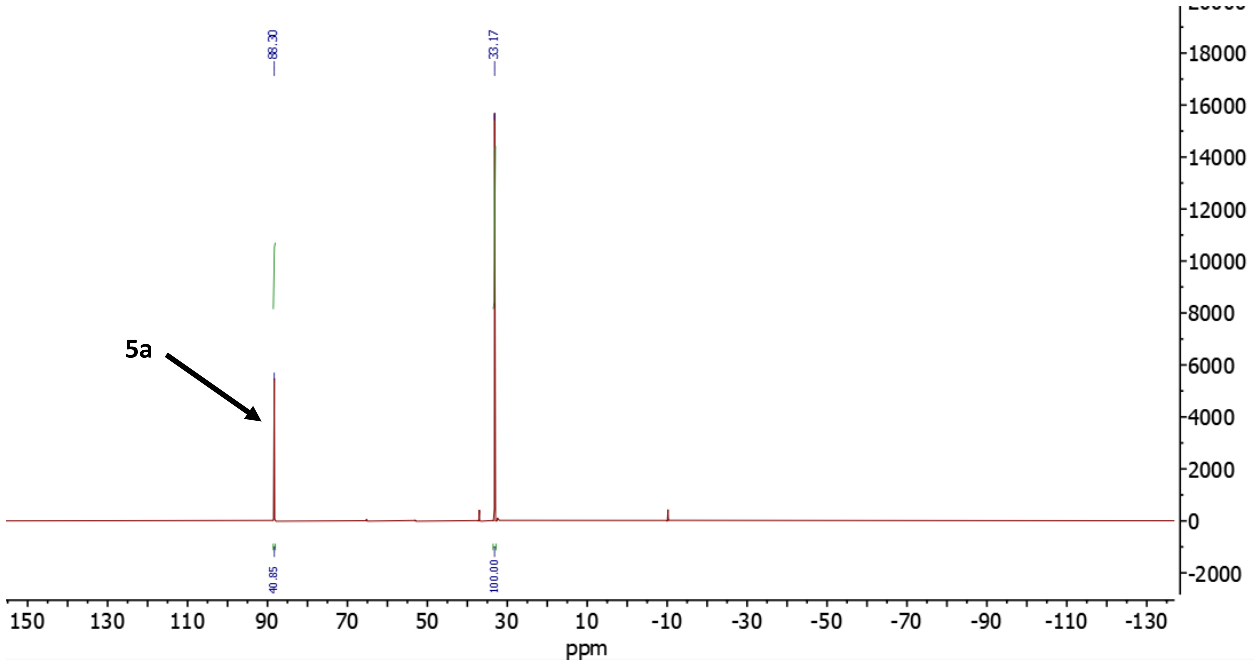

Figure S76. Final ^{31}P spectrum.

Observation of light-induced transmetalation to an arylpalladium complex

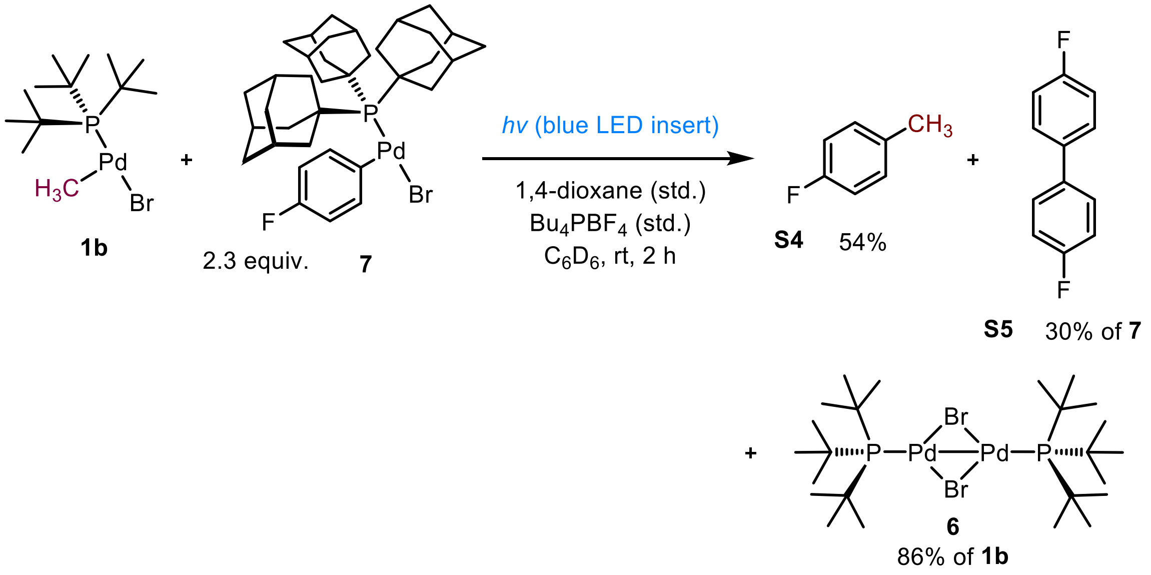

In a nitrogen-filled glovebox, a solution of **7** (4 mg, 5.6 μmol, 2.3 equiv.) in C_6D_6 (490 μL) was prepared. **1b** (1 mg, 2.5 μmol) as well as the standards 1,4-dioxane (1 μL, 12 μmol) and Bu_4PBF_4 (1.2 mg, 3.3 μmol) were added using stock solutions. This reaction mixture was transferred to a specialized J Young NMR tube, sealed with a cap that contained a glass insert,[11] and the tube was sealed with additional parafilm. The tube was exported from the glovebox, a fiber optic cable was inserted, and the sample was placed in the spectrometer. Initial dark ^1H, ^{19}F and ^{31}P spectra were recorded, then the light source was turned on (set to 10 of a maximum of 10) and ^1H, ^{19}F and ^{31}P spectra were intermittently recorded for the duration of the experiment, over a two hour period. Conversion was assessed by ^1H NMR relative to 1,4-dioxane for **S4** and **S5**, as well as by ^{31}P NMR relative to Bu_4PBF_4 for **1b**, **7** and **6**. A dark control reaction did not indicate any 4-fluorotoluene formation.

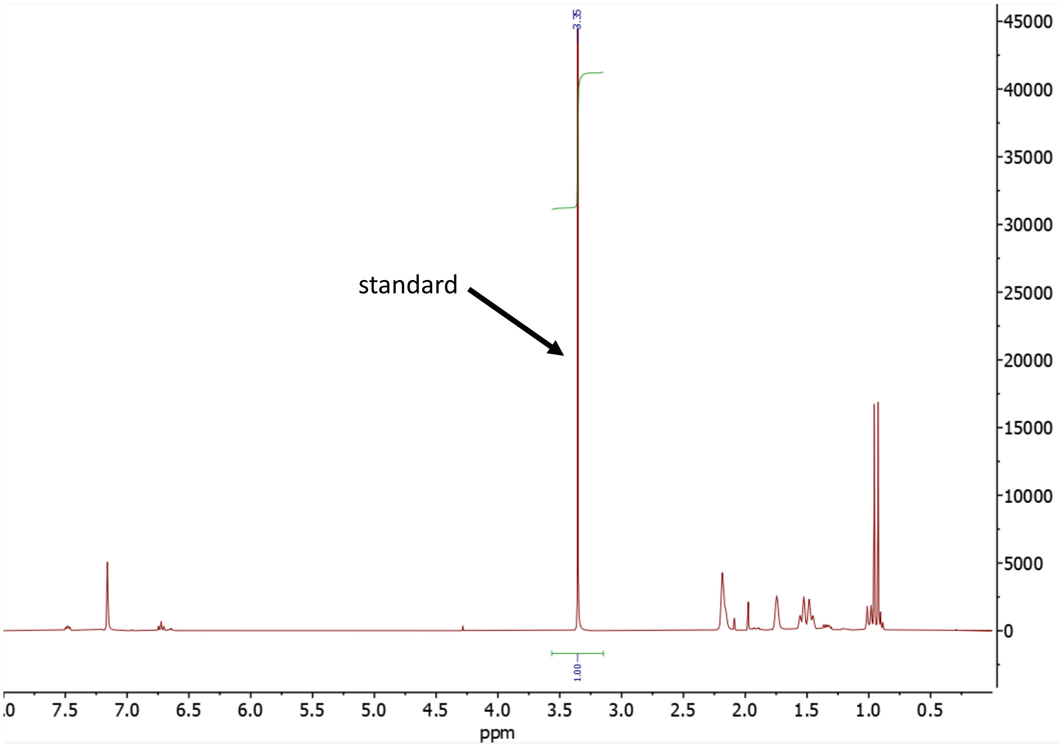

Figure S77. Initial ^{1}H NMR before irradiation.

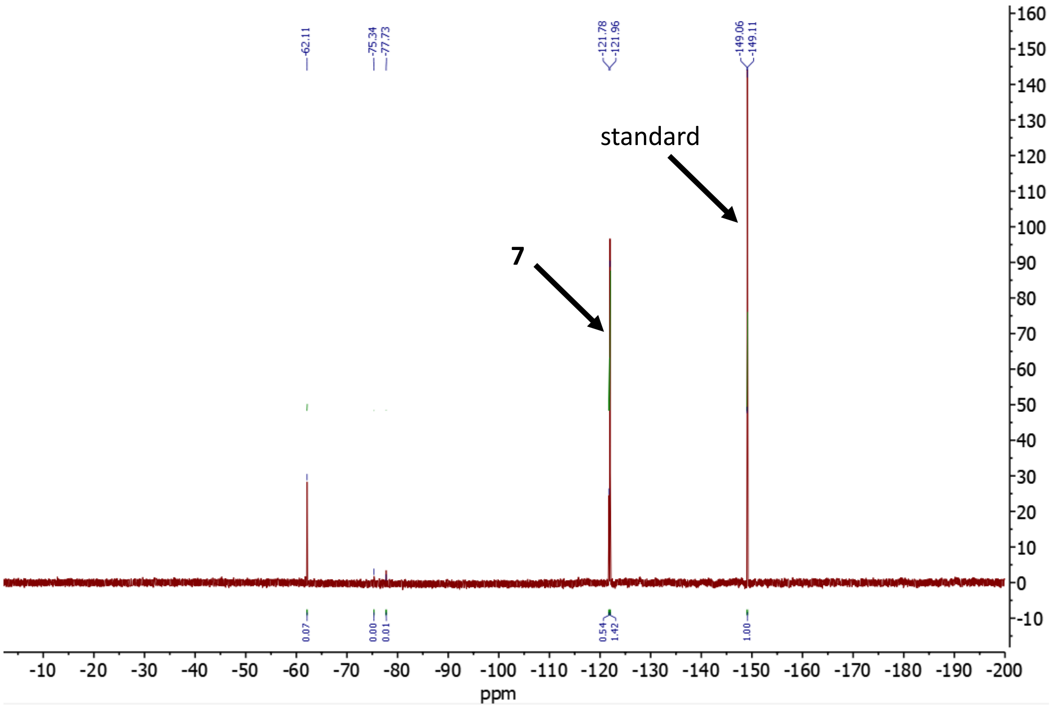

Figure S78. Initial ^{19}F NMR before irradiation.

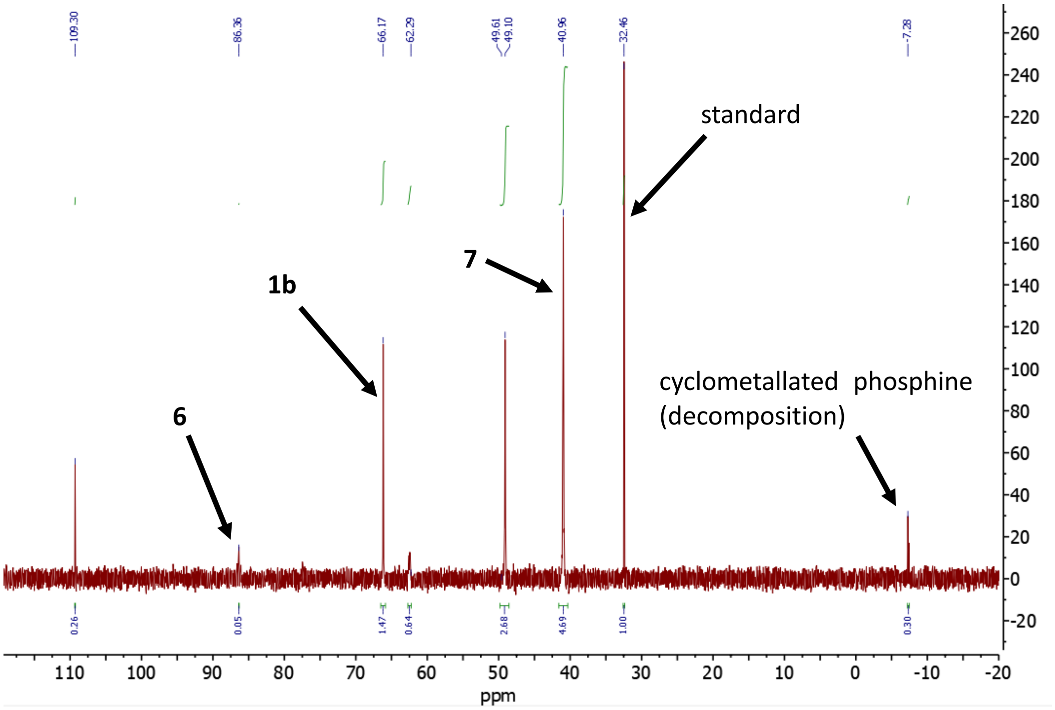

Figure S79. Initial ^{31}P NMR before irradiation.

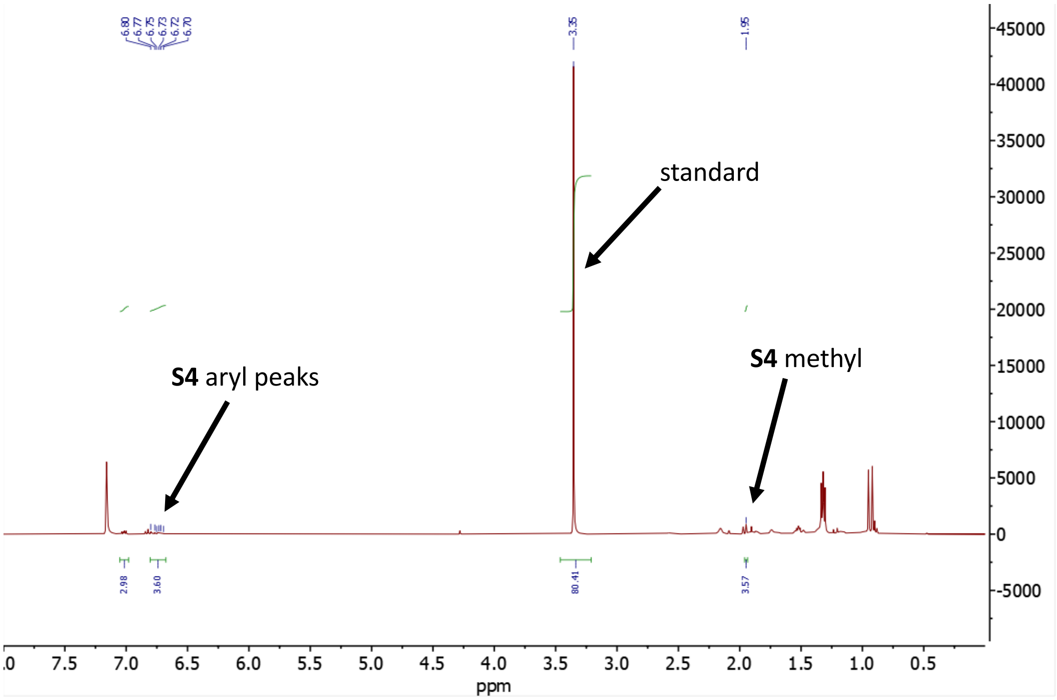

Figure S80. Final ^{1}H NMR (2 h of irradiation).

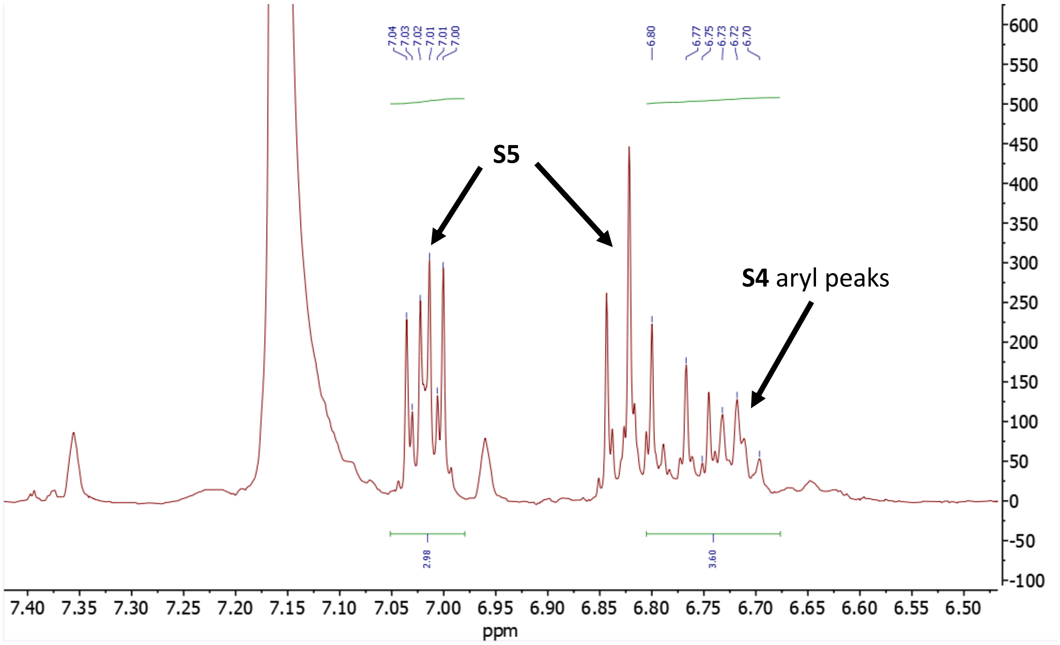

Figure S81. Detail of final ^{1}H NMR (2 h of irradiation) showing aryl peaks of 4-fluorotoluene.

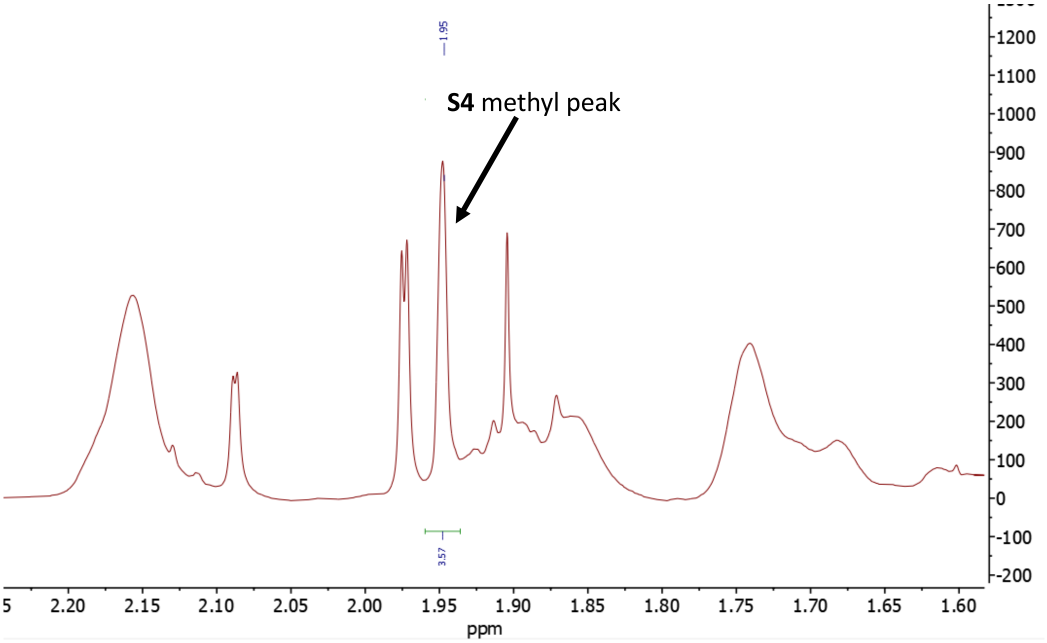

Figure S82. Detail of final ^{1}H NMR (2 h of irradiation) showing methyl peak of 4-fluorotoluene.

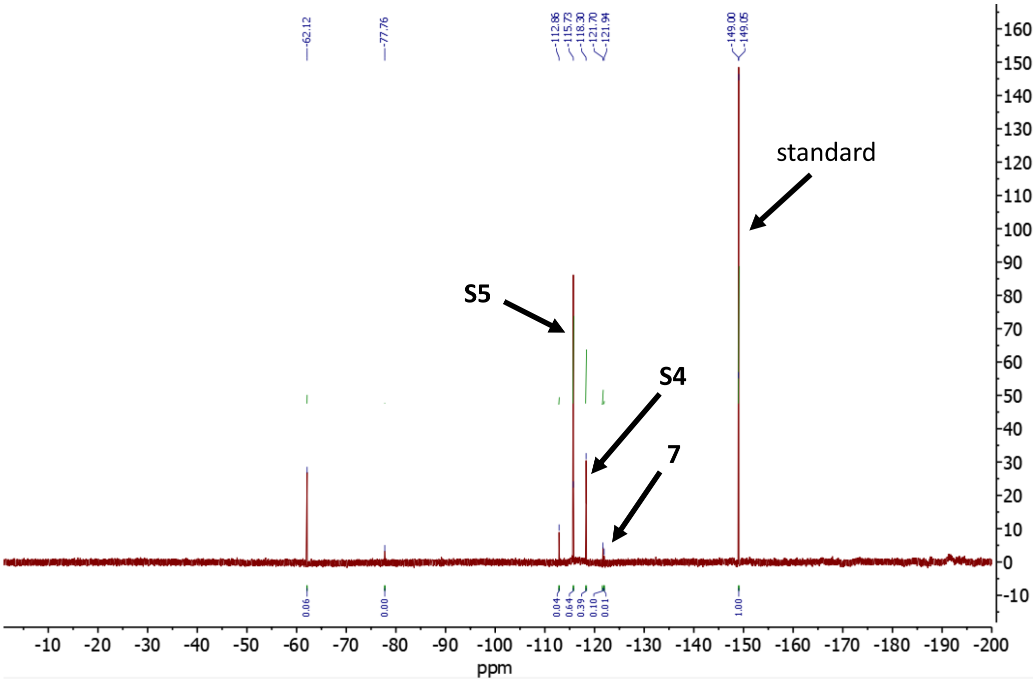

Figure S83. Final ^{19}F NMR (2 h of irradiation).

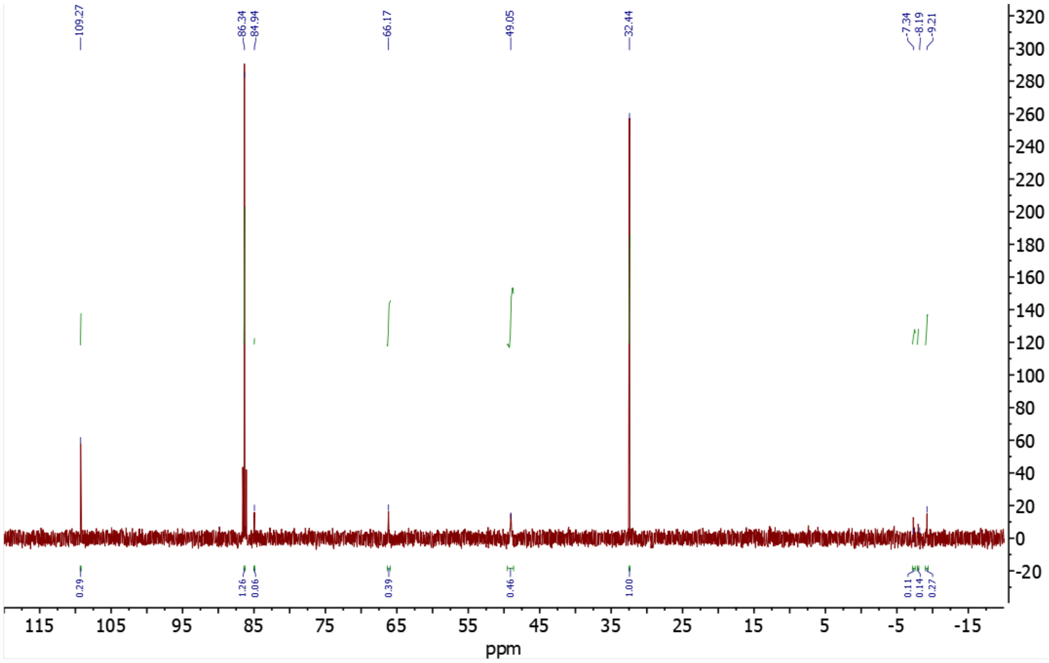

Figure S84. Final ^{31}P NMR (2 h of irradiation).

^{31}P and ^{19}F NMR indicated that some reaction took place after mixing but before irradiation, although 4-fluorotoluene was not generated.

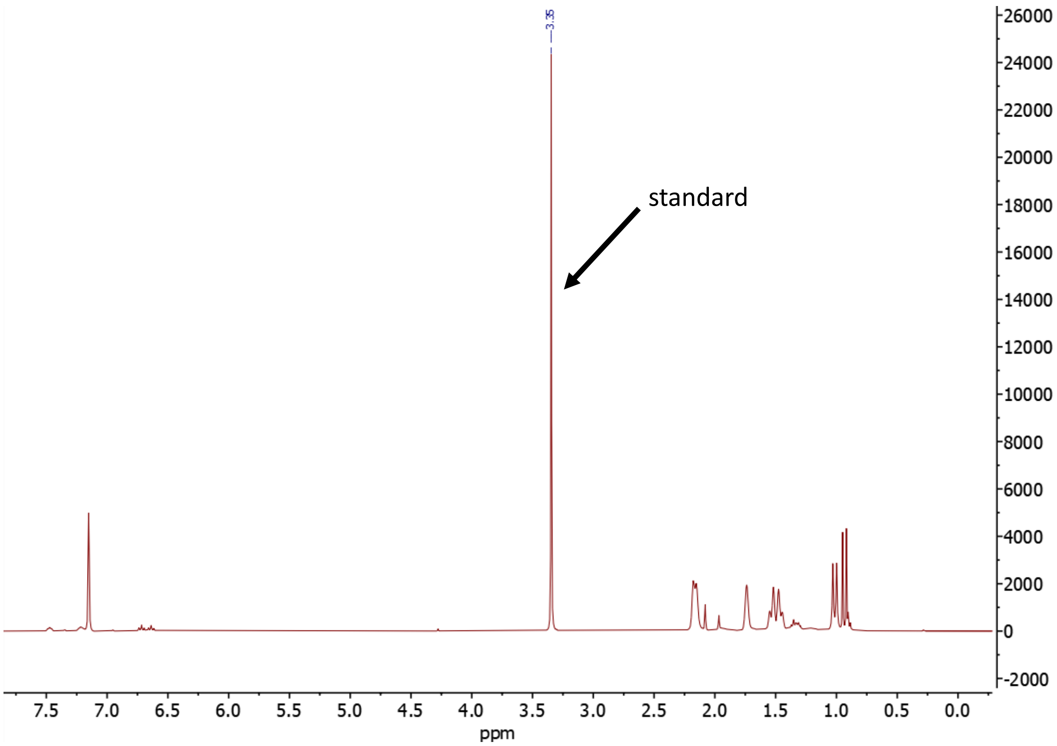

Figure S85. Dark control reaction: final ^{1}H spectrum (dark 2 h).

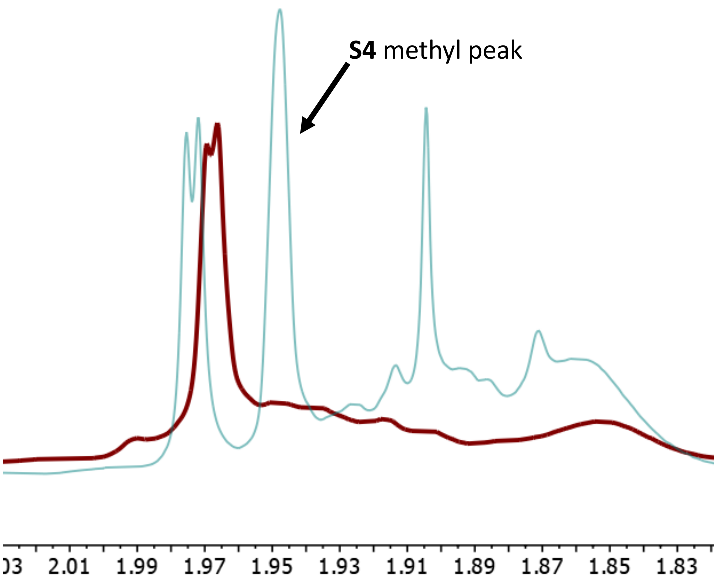

Figure S86. Overlay of ^{1}H NMR spectra: reaction with irradiation (blue) and dark control reaction (red) showing the absence of **S4** methyl peak in the latter spectrum.

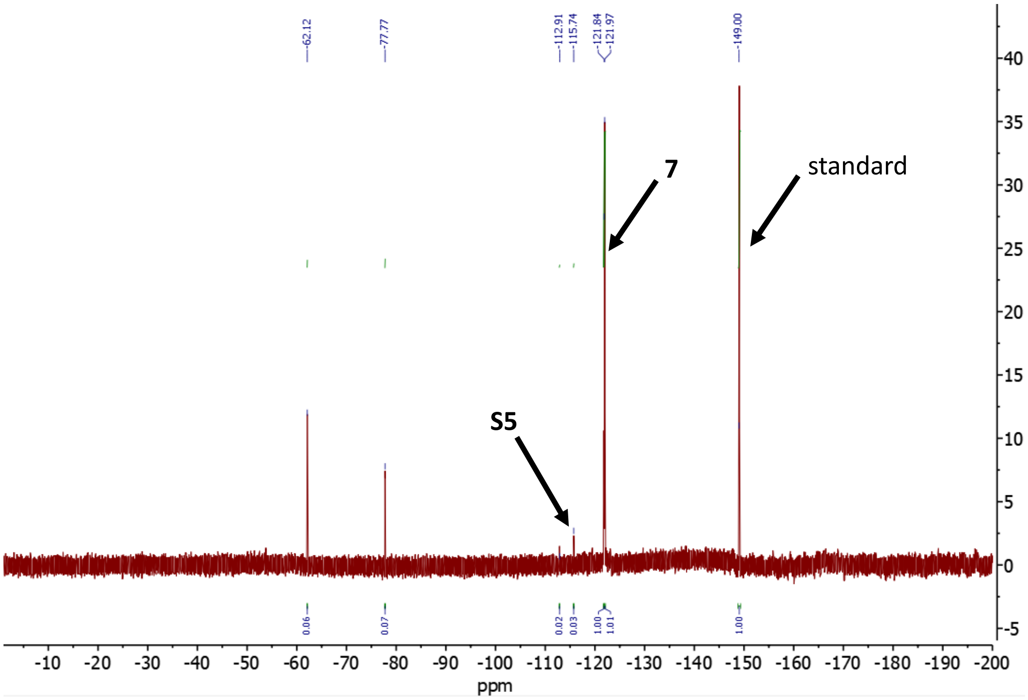

Figure S87. Dark control reaction: final ^{19}F NMR (dark 2 h).

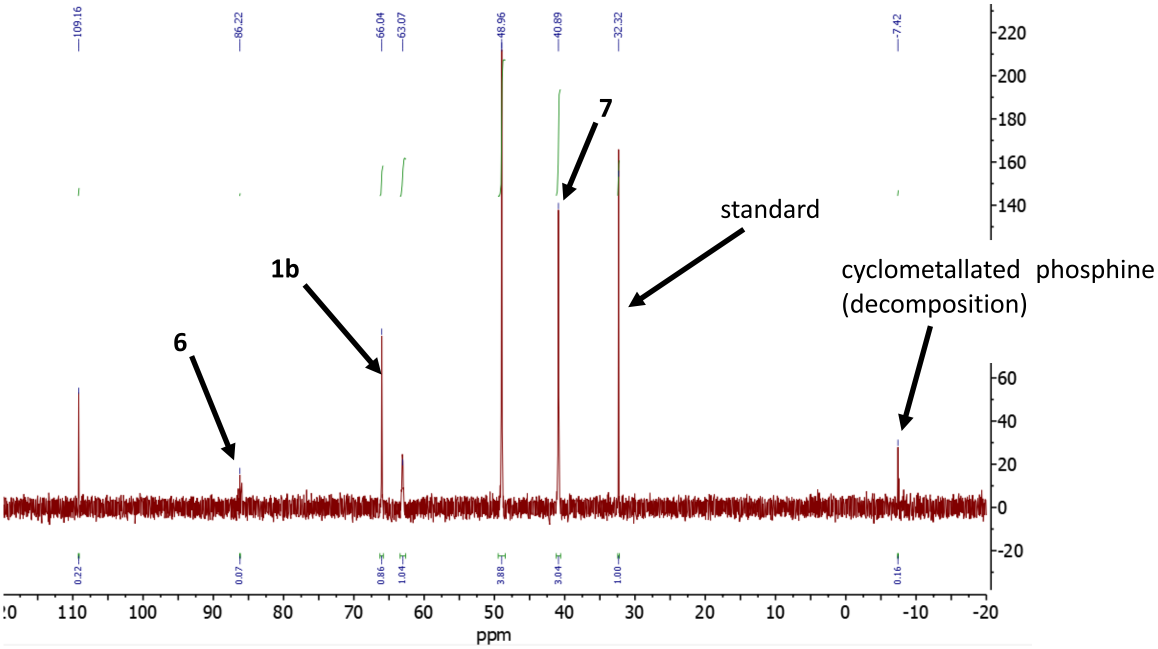

Figure S88. Dark control reaction: final ^{31}P NMR (dark 2 h).

Quantum yield determination details

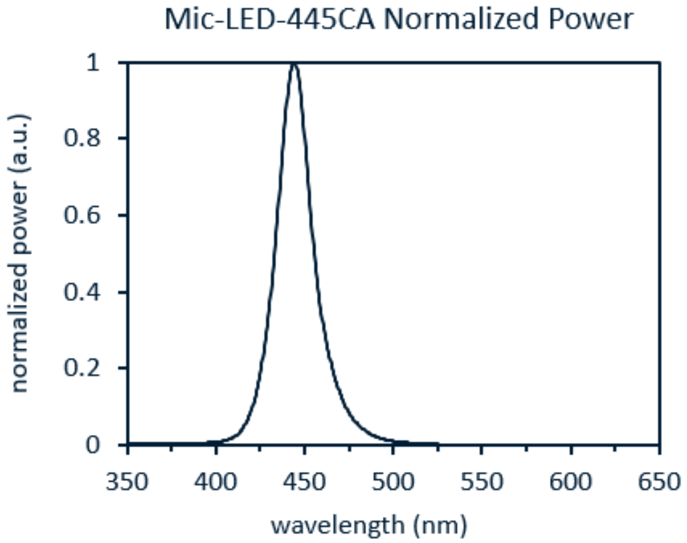

Figure S89. Spectrum of the light source used to conduct the LED-NMR reactions for quantum yield determination, etc.

Construction of a calibration curve to determine the intensity of the light source

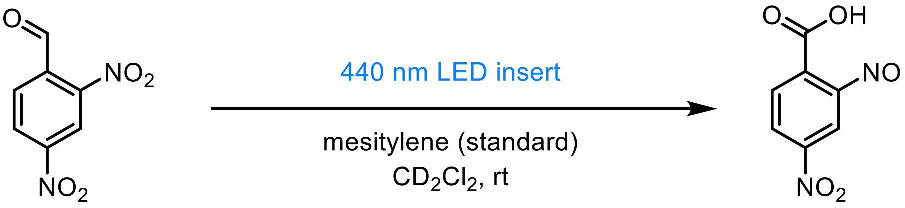

The following was done according to the procedures described by Ji and co-workers.[11] Solutions of different concentrations of 2,4-dinitrobenzaldehyde (2,4,-DNBA) were prepared in CD_2Cl_2 (490 µL) with mesitylene standard (10 µL, 72 µmol) in a nitrogen-filled glovebox. For each, the solution was transferred to an NMR tube and the coaxial insert was inserted. The tube was then sealed first with electrical tape and then with parafilm. The tubes were exported from the glovebox and then the fiber optic cable was inserted and the sample was placed in the spectrometer. An initial dark ^1H spectrum was recorded, then the light source was turned on (set to 1.0 of a maximum of 10) and spectra were continuously recorded for the duration of the experiment by the steady state technique. The initial rates of product formation were extracted and used to build a calibration curve.

Table S2. Initial rates of product formation at different initial concentrations of 2,4-DNBA.

$[2,4\text{-DNBA}]_0$, mM	rate, mM/min
54	0.0290
134	0.0463
148	0.0438
190	0.0547
212	0.0596
310	0.0669
454	0.0771

The initial concentrations were calculated by ^{1}H NMR spectroscopy by integration of the 2,4-DNBA peaks relative to the mesitylene standard.

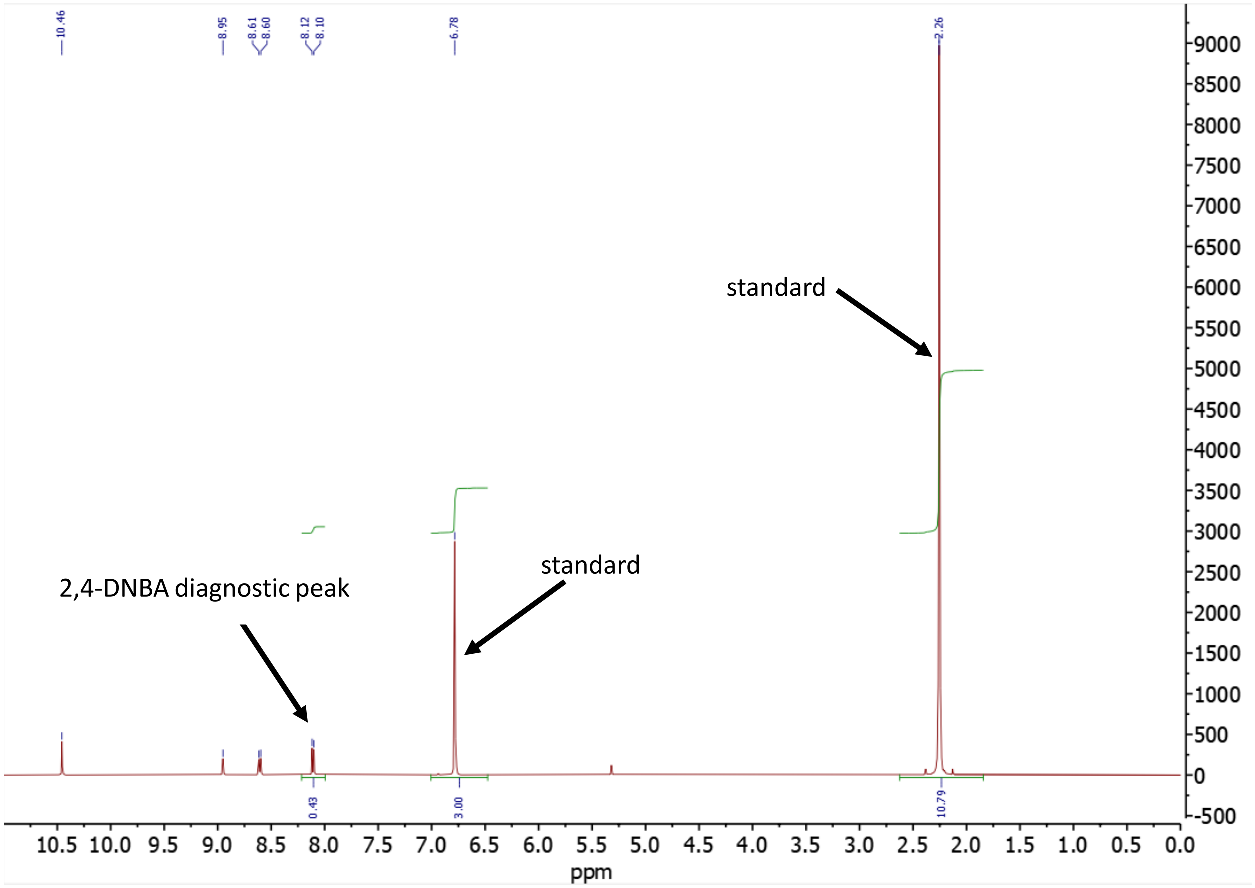

Figure S90. Representative initial ^{1}H spectrum for reactions of 2,4-DNBA with 440 nm LED insert (from Table S2 entry 1).

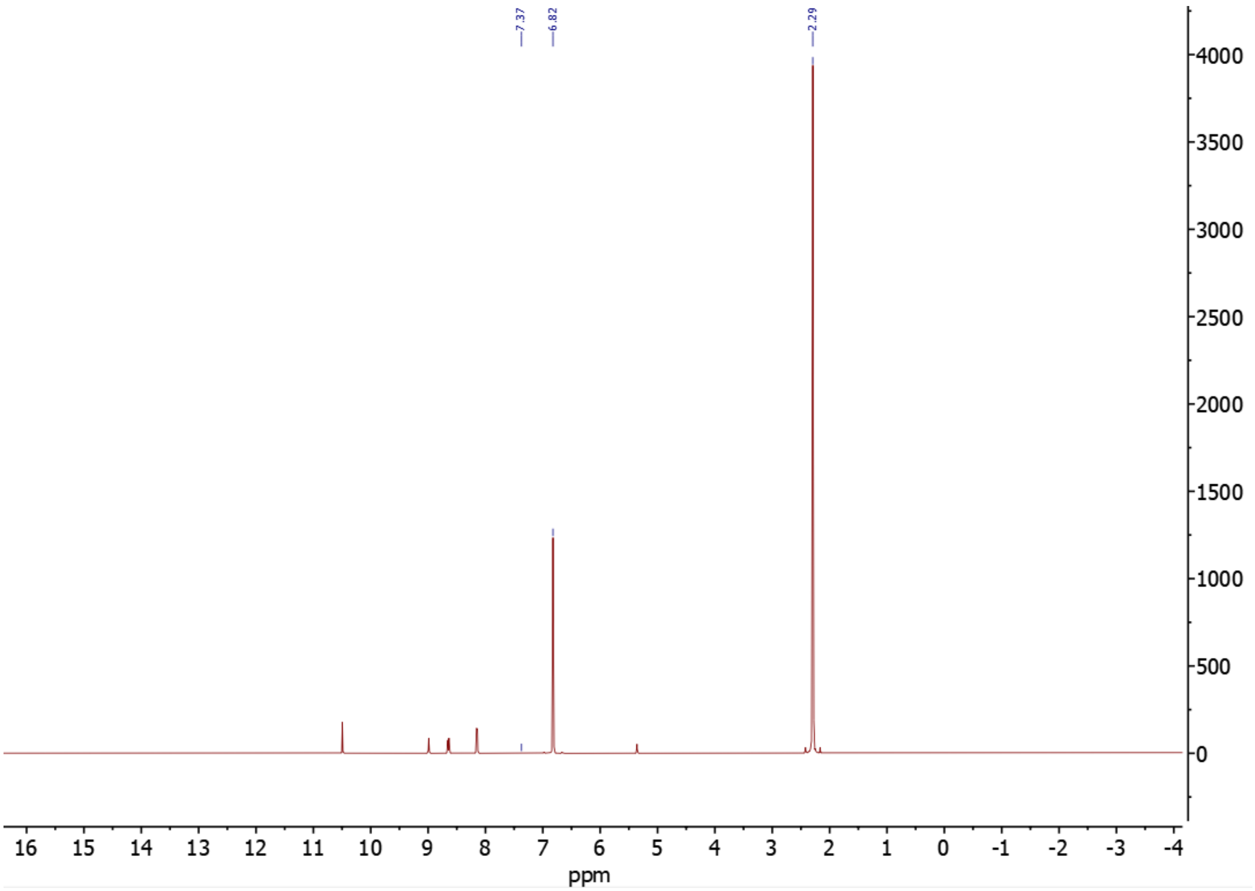

Figure S91. ^{1}H spectrum from Table S2 entry 1 after 1419 seconds of irradiation.

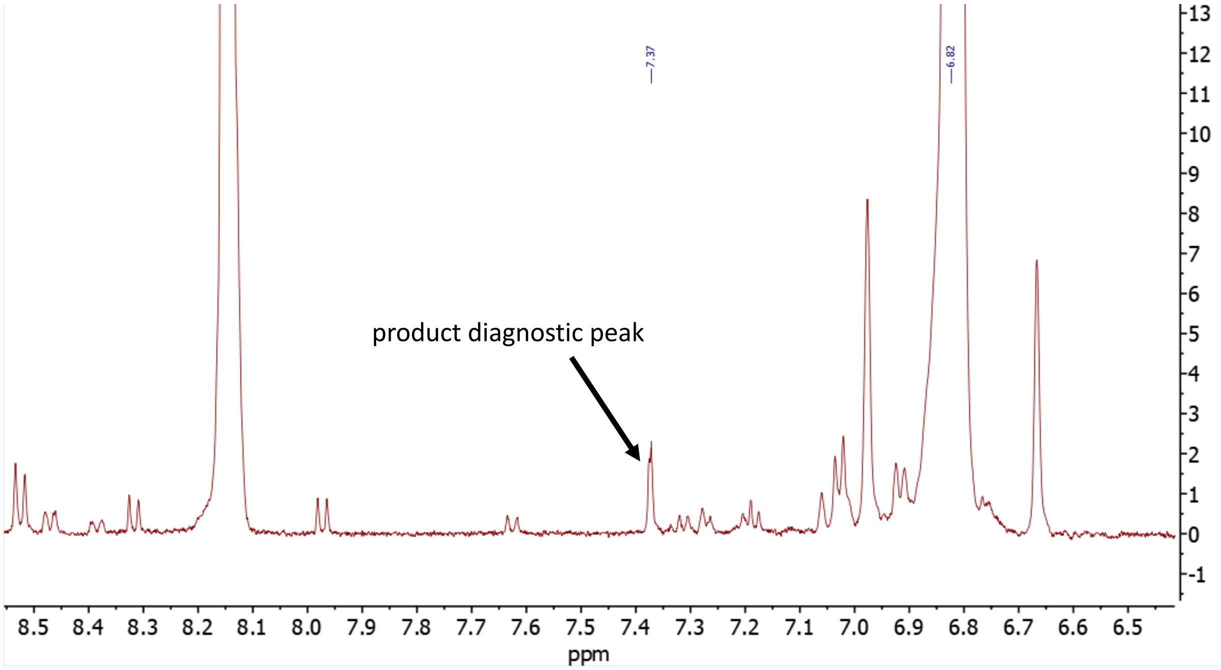

Figure S92. Detail of ^{1}H spectrum from Table S2 entry 1 after 1419 seconds of irradiation showing product peak.

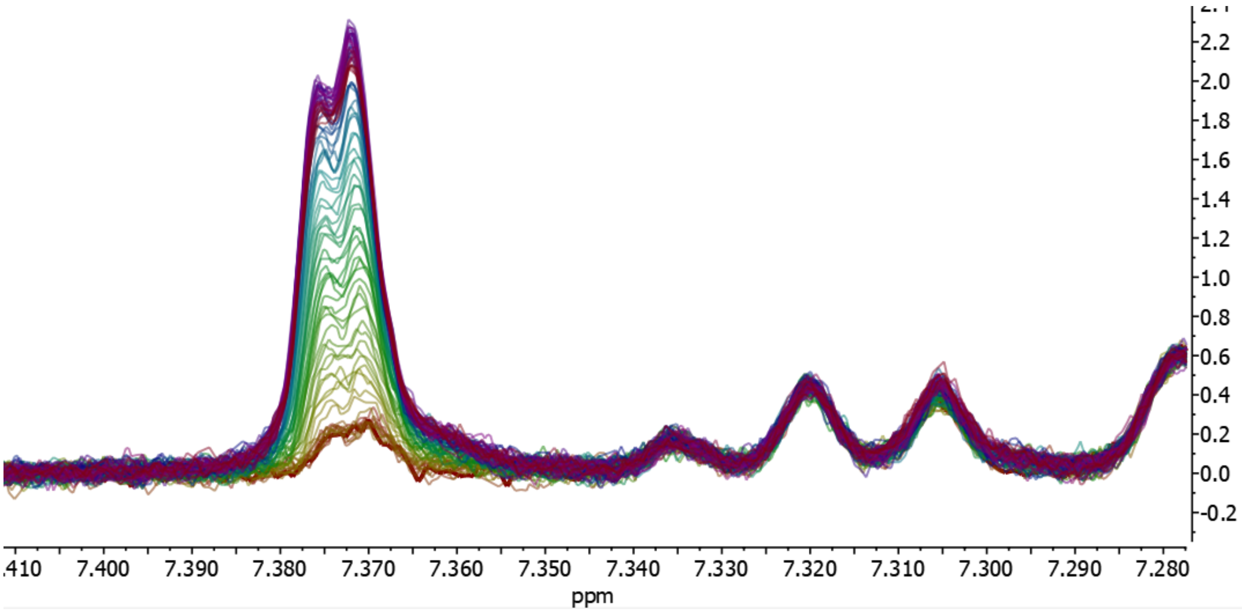

Figure S93. Stacked series of ^{1}H NMR spectra showing evolution of product over time from Table S2 entry 1.

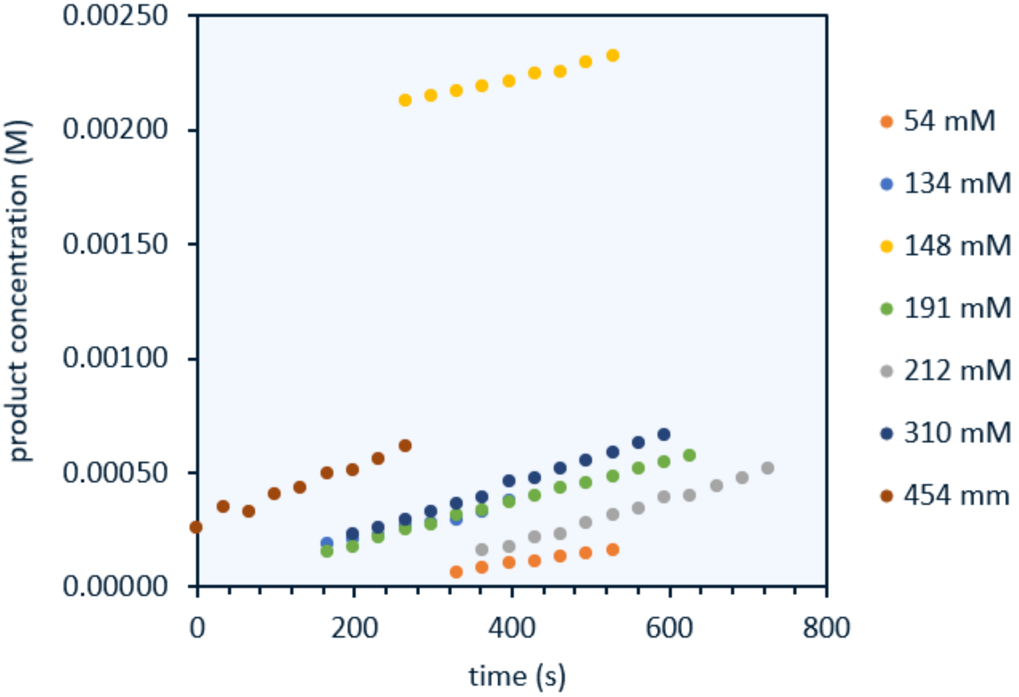

Figure S94. Overlaid plots of initial rates/linear regions of reactions of 2,4-DNBA with 440 nm light from Table S2. Legend indicates initial 2,4-DNBA concentration as determined by ^{1}H NMR.

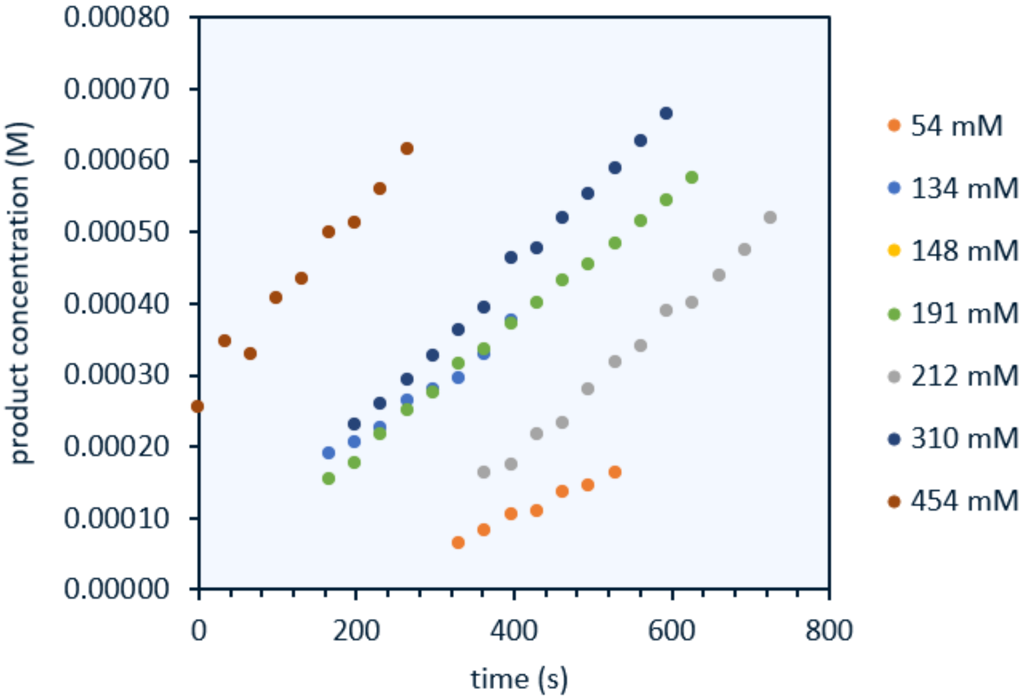

Figure S95. Overlaid plots of initial rates/linear regions of reactions of 2,4-DNBA with 440 nm light from Table S2, detail. Legend indicates initial 2,4-DNBA concentration as determined by ^{1}H NMR.

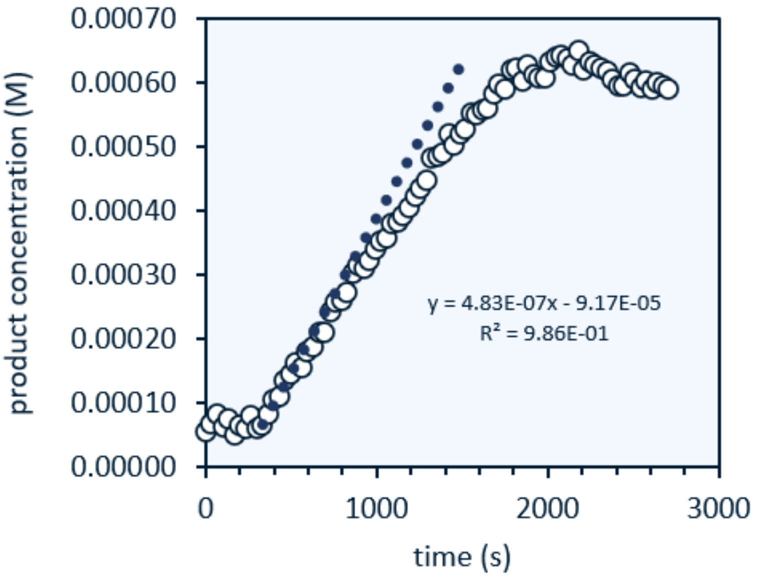

Figure S96. Concentration of product over time from reaction of 2,4-DNBA with 440 nm light at $[2,4\text{-DNBA}]_0$ = 54 mM from Table S2 entry 1: initial rates of formation. A series of dark spectra were initially recorded, then the light was turned on and the linear region of

product formation was measured. Note the drop in product formation due to precipitation of the product.

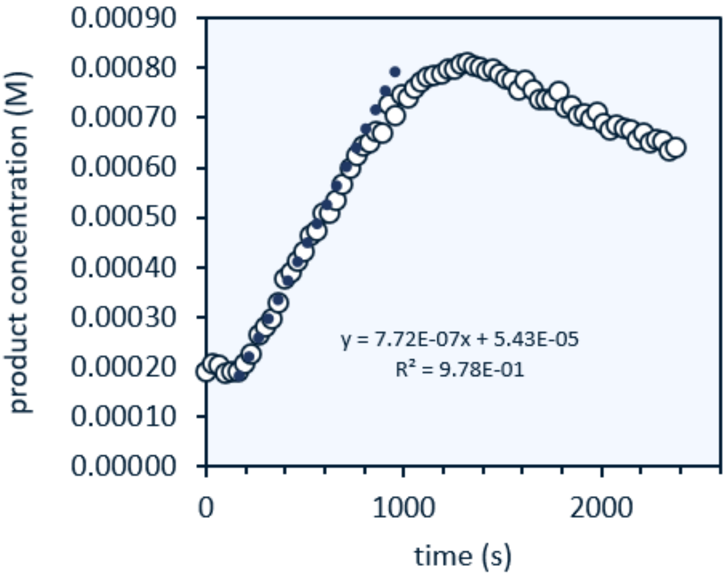

Figure S97. Concentration of product over time from reaction of 2,4-DNBA with 440 nm light at $[2,4\text{-DNBA}]_0$ = 134 mM from Table S2 entry 2: initial rates of formation. A series of dark spectra were initially recorded, then the light was turned on and the linear region of product formation was measured. Note the drop in product formation due to precipitation of the product.

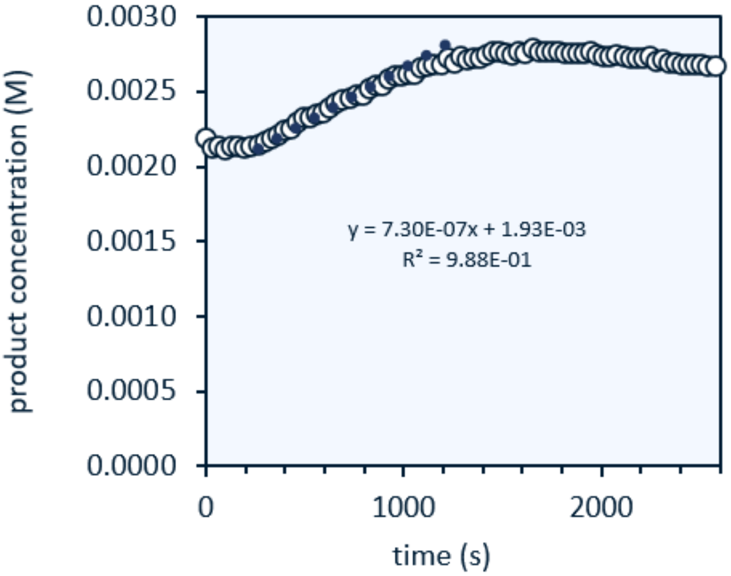

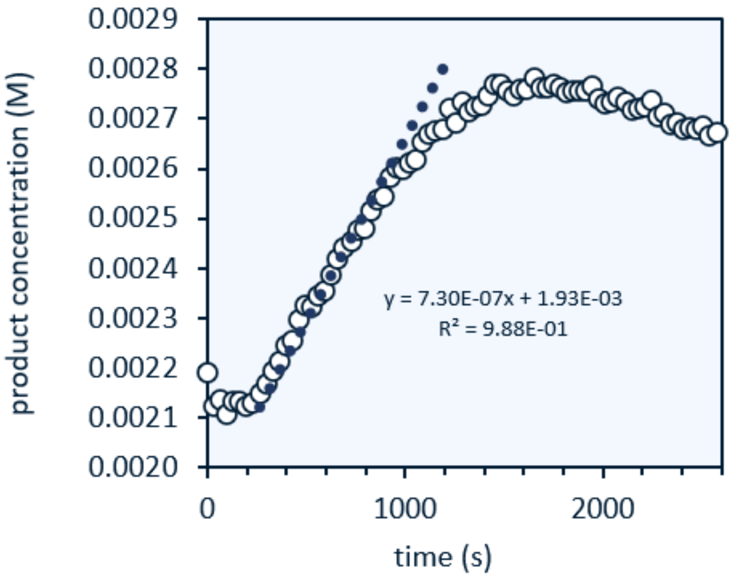

Figure S98. Concentration of product over time from reaction of 2,4-DNBA with 440 nm light at $[2,4\text{-DNBA}]_0$ = 148 mM from Table S2 entry 3: initial rates of formation. A series of dark spectra were initially recorded, then the light was turned on and the linear region of product formation was measured. Note the drop in product formation due to precipitation of the product. Above: full view. Below: zoomed-in view.

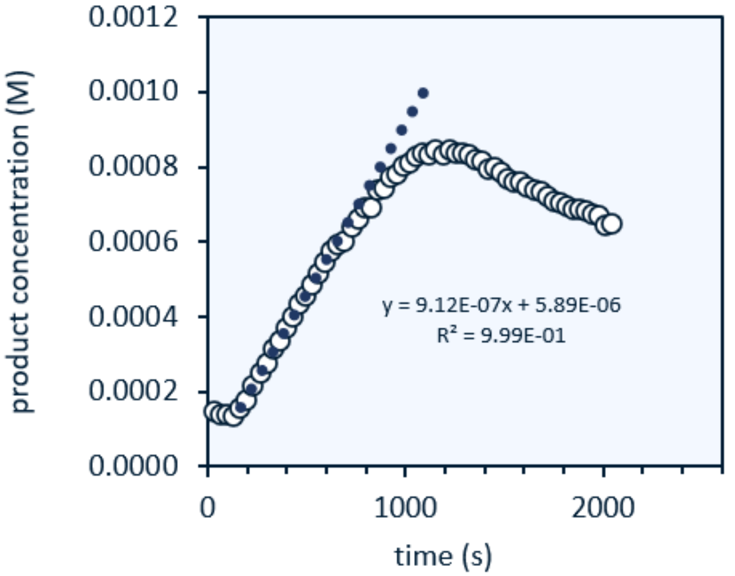

Figure S99. Concentration of product over time from reaction of 2,4-DNBA with 440 nm light at $[2,4\text{-DNBA}]_0$ = 190 mM from Table S2 entry 4: initial rates of formation. A series of dark spectra were initially recorded, then the light was turned on and the linear region of

product formation was measured. Note the drop in product formation due to precipitation of the product.

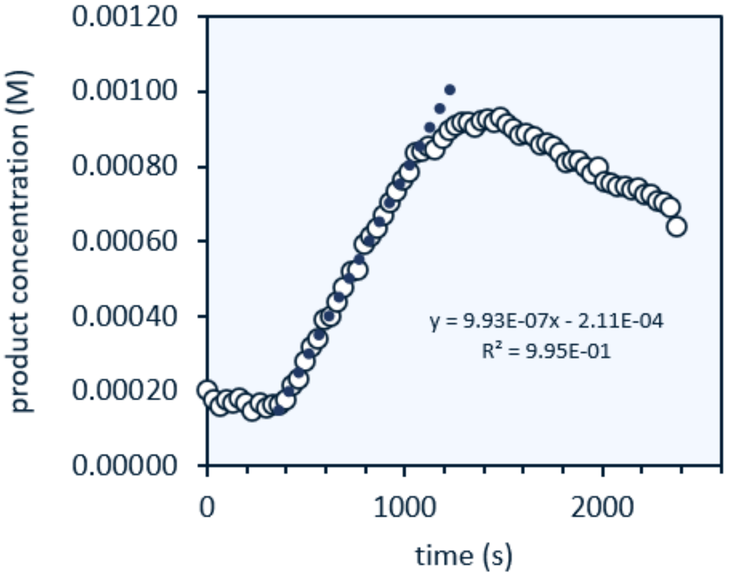

Figure S100. Concentration of product over time from reaction of 2,4-DNBA with 440 nm light at [2,4-DNBA]$_0$ = 212 mM from Table S2 entry 5: initial rates of formation. A series of dark spectra were initially recorded, then the light was turned on and the linear region of product formation was measured. Note the drop in product formation due to precipitation of the product.

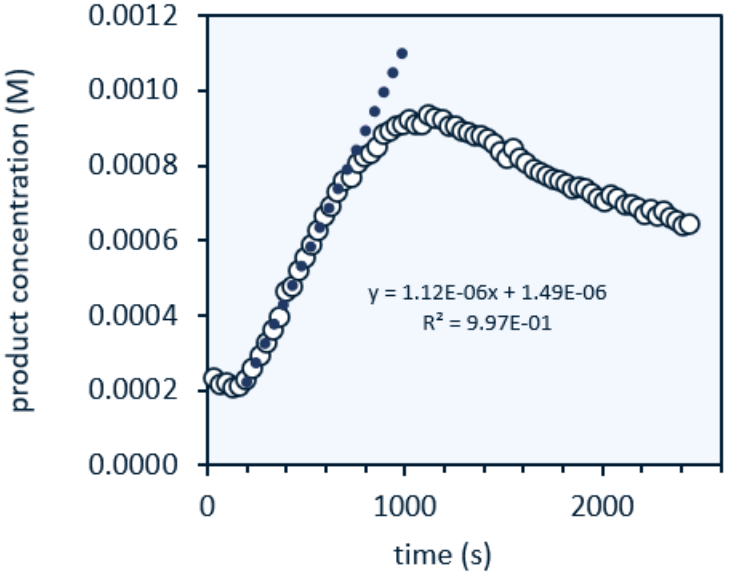

Figure S101. Concentration of product over time from reaction of 2,4-DNBA with 440 nm light at $[2,4\text{-DNBA}]_0$ = 310 mM from Table S2 entry 6: initial rates of formation. A series of dark spectra were initially recorded, then the light was turned on and the linear region of product formation was measured. Note the drop in product formation due to precipitation of the product.

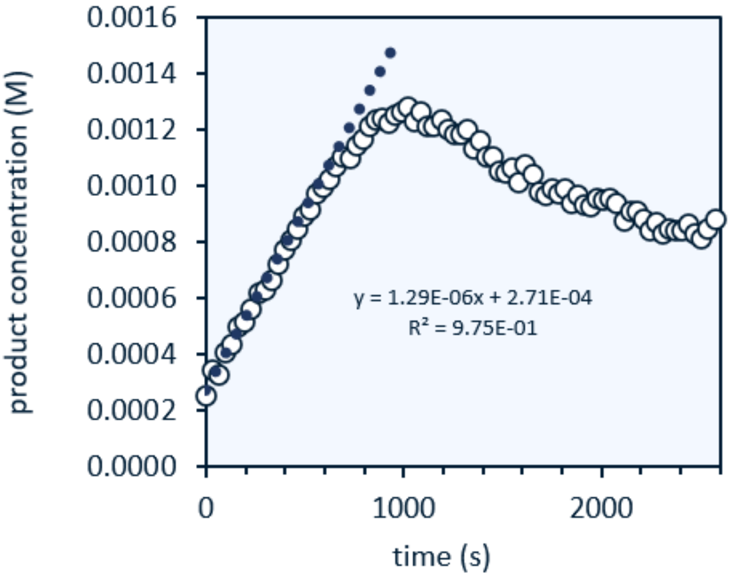

Figure S102. Concentration of product over time from reaction of 2,4-DNBA with 440 nm light at $[2,4\text{-DNBA}]_0$ = 454 mM from Table S2 entry 7: initial rates of formation. Note the drop in product formation due to precipitation of the product.

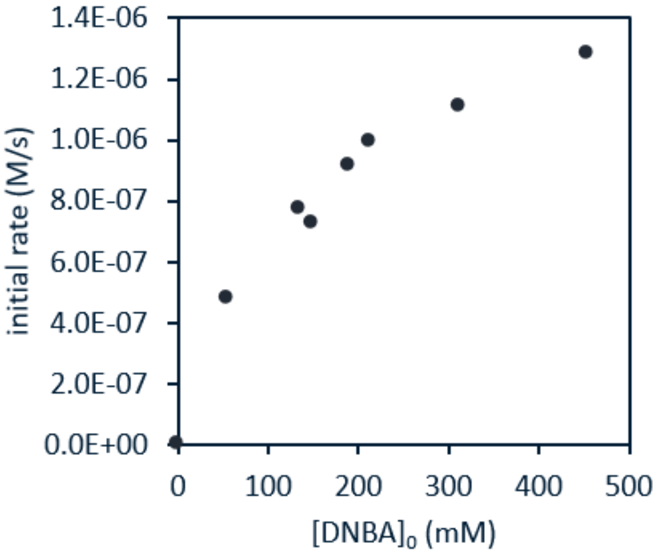

Figure S103. Plots of the initial rates of reaction of 2,4-DNBA at different concentrations.

Calculations to determine the intensity of the light source:

The following equation was used to determine the intensity of the light source, given that Φ = 0.077, $\varepsilon = 23.4$ M^{-1}cm^{-1}, and $b = 0.11$ cm, values taken from Ji and co-workers.

$$-\frac{d[Act]}{dt} = I_0\Phi\left(1 - 10^{-\varepsilon b[Act]}\right) \quad (1)$$

Gnuplot software was used to fit this equation (**Figure S104**). From this, it was determined that $I_0 = 1.8*10^{-5}$ einstein L^{-1}s^{-1}.

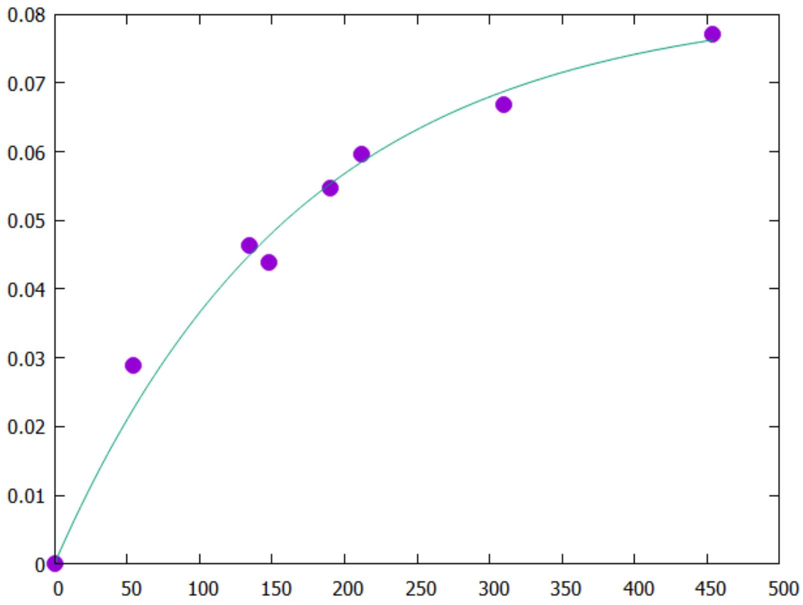

Figure S104. Calibration curve (rate (mM/min) vs. concentration (mM)) for the fiber optic light source using data from Table S2, fit using gnuplot.

With the intensity of the light source in hand, quantum yields (Φ) for the reactions of interest were determined as follows. In order to ensure that the observed rate corresponded to the photon-limited regime, the reaction was performed at two different initial concentrations, the second reaction twice was concentrated as the first. If the same initial rate was measured, we considered the reaction to be in the zero order, photon limited regime. Thus, the observed rate corresponded to the zeroth order kinetic constant k_0. Then, the following equation was applied, from which Φ was determined:

$$\Phi = \frac{k_0}{I_0} \quad (2)$$

Using this technique, quantum yields were determined for the following reactions.

Quantum yield of Pd-C homolytic cleavage of 1a in the presence of excess TEMPO

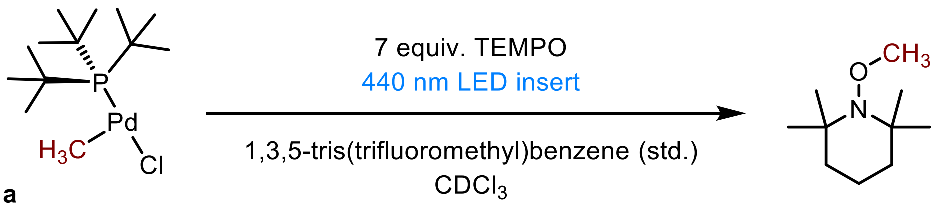

A solution of **1a** at the desired concentration containing 7 equivalents of TEMPO and 10 µL 1,3,5-tris(trifluoromethyl)benzene (std.) in 490 µL $CDCl_3$ was prepared in a nitrogen filled glovebox. This solution was transferred to an NMR tube, a coaxial insert was inserted,[11] and the tube was sealed first with electrical tape and then parafilm. The tube was exported from the glovebox, the fiber optic cable was inserted, and the sample was placed in the spectrometer. An initial dark ^{1}H spectrum was recorded, then ^{1}H NMR spectra began to be continuously recorded. After several dark spectra were recorded, the light source was turned on (set to 1.0 of a maximum of 10) and spectra were continuously recorded for the duration of the experiment by the steady state technique. The initial rate of TEMPO-Me formation was observed at two different initial concentrations of **1a**, one about twice as concentrated as the other. Essentially the same initial rate was observed for both reactions, and so it was used to determine the zeroth order kinetic constant k_0 (see Figure S110 to S114). Applying (2), using the value of I_0 determined from the calibration using 2,4-DNBA, then allowed Φ to be determined.

Table S3. Initial rates of TEMPO-Me formation at different initial concentrations of **1a**, several runs.

Entry	$[1a]_0$, mM	rate, M/s	Φ
1	120	$1.2*10^{-5}$	0.65
2	60	$9.3*10^{-6}$	0.52
3	60	$1.3*10^{-5}$	0.73

4	60	$1.4*10^{-5}$	0.78
5	60	$1.0*10^{-5}$	0.56

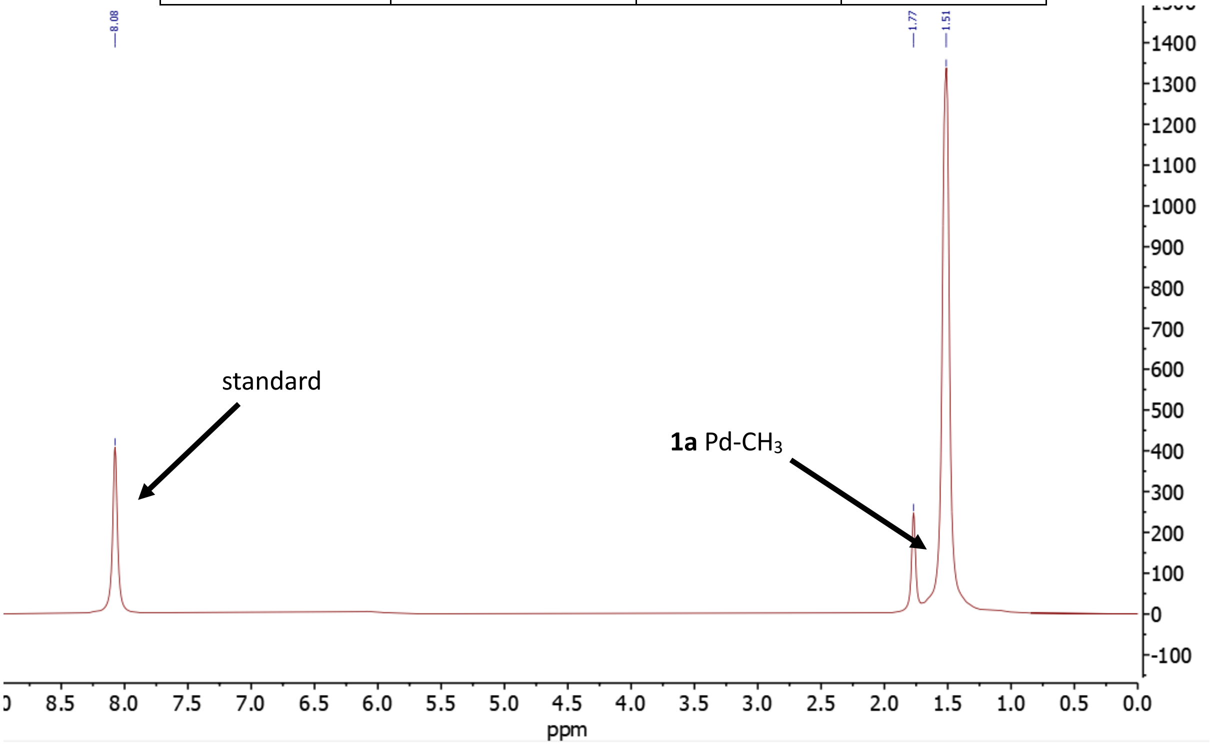

Figure S105. Initial ^{1}H spectrum of representative reaction from Table S3 (entry 2).

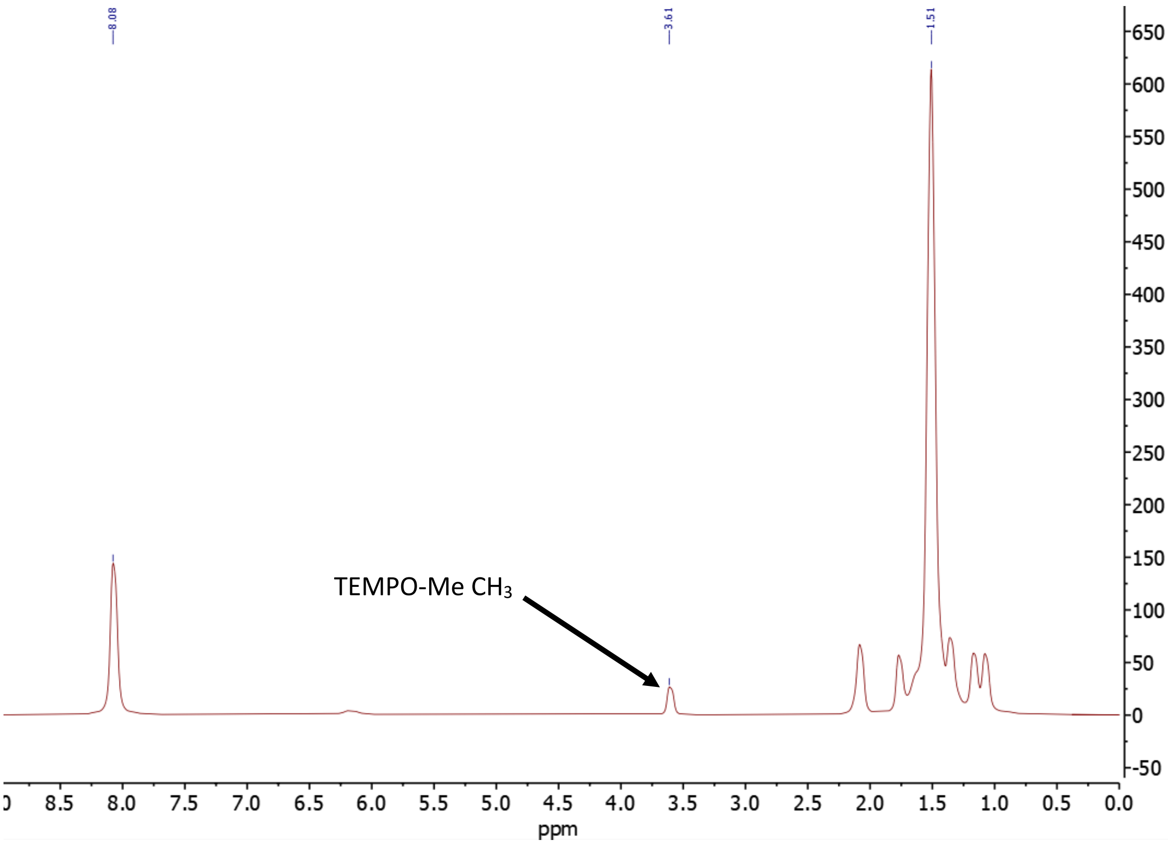

Figure S106. ^{1}H spectrum of representative reaction from Table S3 (entry 2) after 3795 seconds of irradiation showing product peak.

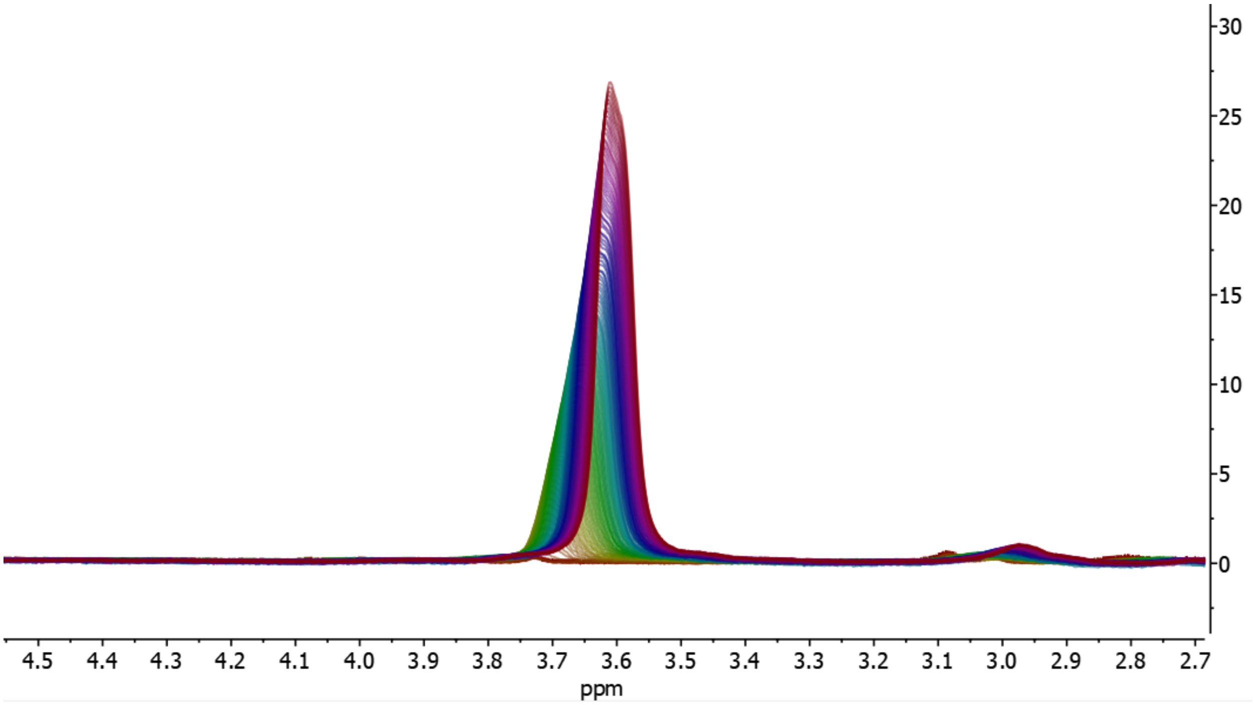

Figure S107. Overlay of ^{1}H spectra from Table S3 entry 2 showing evolution of product peak over time.

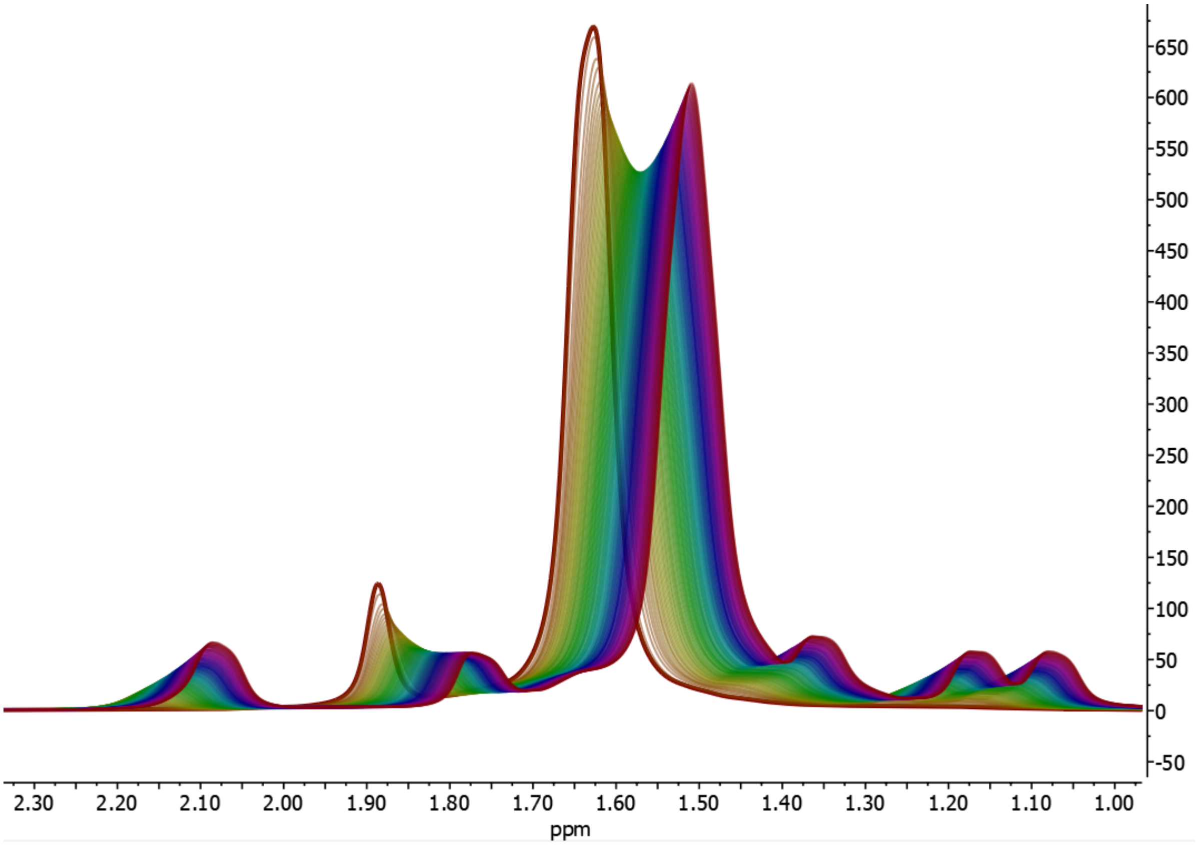

Figure S108. Overlay of ^{1}H spectra from Table S3 entry 2 showing decrease of starting material peaks over time.

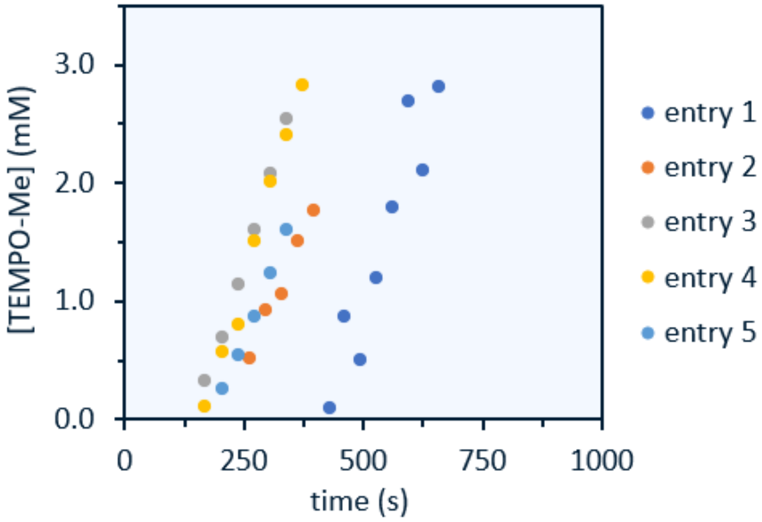

Figure S109. Overlaid plots of initial rates/linear regions of reactions from Table S3.

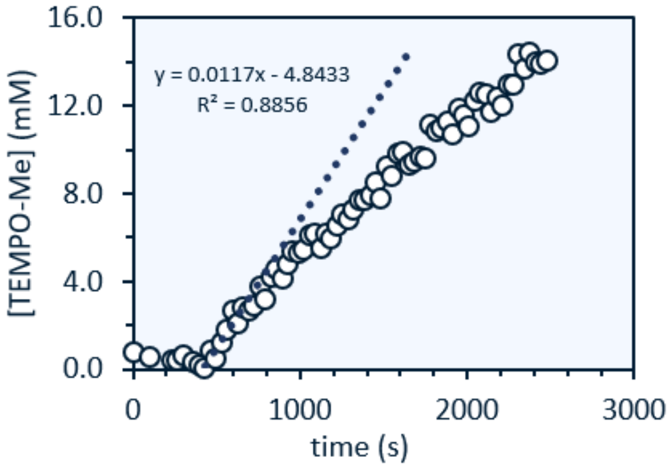

Figure S110. Initial rates of TEMPO-Me formation for irradiation of **1a** in the presence of excess TEMPO. In this case $[\mathbf{1a}]_0$ = 120 mM, see Table S3 entry 1. A series of dark spectra were initially recorded, where the concentration of TEMPO-Me does not change. The initial rate was determined from the first few data points recorded after irradiation began.

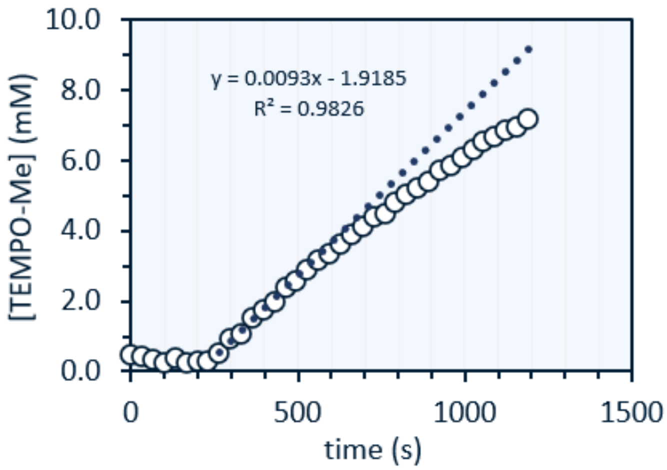

Figure S111. Initial rates of TEMPO-Me formation for irradiation of **1a** in the presence of excess TEMPO. In this case $[\mathbf{1a}]_0$ = 60 mM, see Table S3 entry 2. A series of dark spectra were initially recorded, where the concentration of TEMPO-Me does not change. The initial rate was determined from the first few data points recorded after irradiation began.

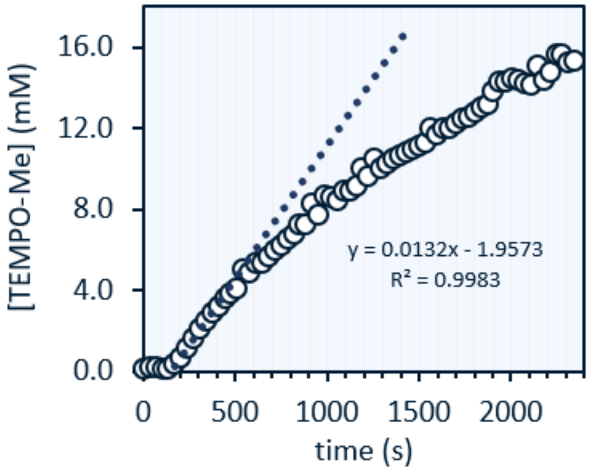

Figure S112. Initial rates of TEMPO-Me formation for irradiation of **1a** in the presence of excess TEMPO. In this case $[\mathbf{1a}]_0$ = 60 mM, see Table S3 entry 3. A series of dark spectra were initially recorded, where the concentration of TEMPO-Me does not change. The initial rate was determined from the first few data points recorded after irradiation began.

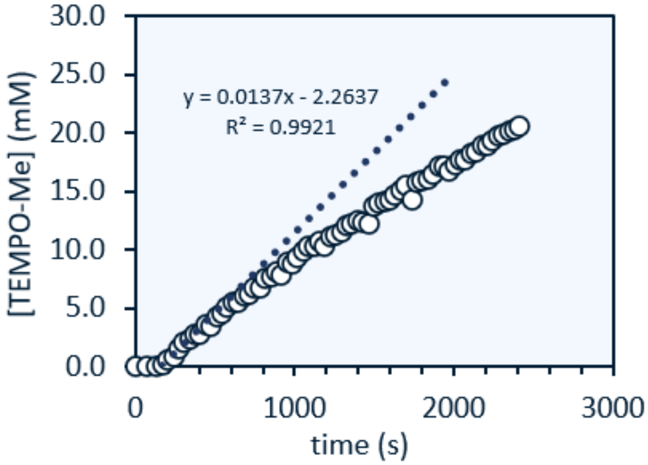

Figure S113. Initial rates of TEMPO-Me formation for irradiation of **1a** in the presence of excess TEMPO. In this case $[\mathbf{1a}]_0$ = 60 mM, see Table S3 entry 4. A series of dark spectra were initially recorded, where the concentration of TEMPO-Me does not change. The initial rate was determined from the first few data points recorded after irradiation began.

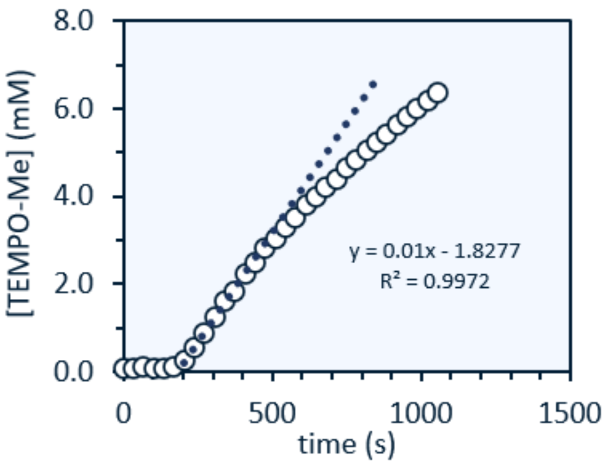

Figure S114. Initial rates of TEMPO-Me formation for irradiation of **1a** in the presence of excess TEMPO. In this case [**1a**]$_0$ = 60 mM, see Table S3 entry 5. A series of dark spectra were initially recorded, where the concentration of TEMPO-Me does not change. The initial rate was determined from the first few data points recorded after irradiation began.

Here, k_0 was determined by the average of several runs at different concentrations. The data are shown above. Averaging the initial rates of these reactions gives $k_0 = 1.2*10^{-5}$ M/s with a standard deviation of $0.2*10^{-5}$ M/s. Given $I_0 = 1.8*10^{-5}$ einstein $L^{-1}s^{-1}$, this gives an average value of $\Phi = 0.67$ with a standard deviation of 0.09. The linear region of the reaction profile appeared to be brief despite the overall conversion being low. In order to get an accurate initial rate, only the linear region for product formation that takes place immediately after irradiation begins was considered.

Quantum yield of Pd-C homolytic cleavage of 1a in the presence of excess TEMPO in solvents other than $CDCl_3$

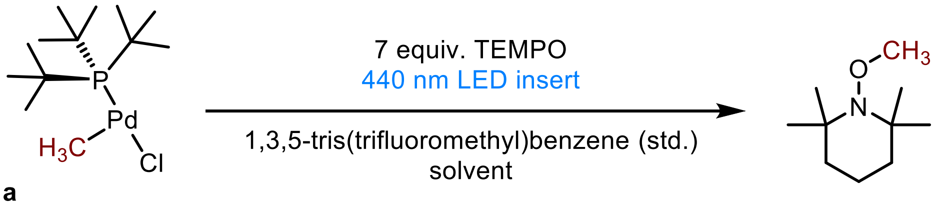

1a

A 120 mM solution of **1a** containing 7 equivalents of TEMPO and 10 μL 1,3,5-tris(trifluoromethyl)benzene (std.) in 490 μL of the desired solvent was prepared in a nitrogen filled glovebox. This solution was transferred to an NMR tube, a coaxial insert was inserted,[11] and the tube was sealed first with electrical tape and then parafilm. The tube was exported from the glovebox, the fiber optic cable was inserted, and the sample was placed in the spectrometer. An initial dark ^{1}H spectrum was recorded, then ^{1}H NMR spectra began to be continuously recorded. After several dark spectra were recorded, the light source was turned on (set to 1.0 of a maximum of 10) and spectra were continuously recorded for the duration of the experiment by the steady state technique. Based on prior experiments, it was assumed that the reactions were in the photon-limited regime. Applying (2), using the value of I_0 determined from the calibration using 2,4-DNBA, then allowed Φ to be determined.

Table S4. Initial rates of TEMPO-Me formation in different solvents.

Entry	$[1a]_0$, mM	Solvent	rate, M/s	Φ
1	120	C_6D_6	$1.2*10^{-6}$	0.67
2	120	MeCN-d_3	$1.4*10^{-6}$	0.75

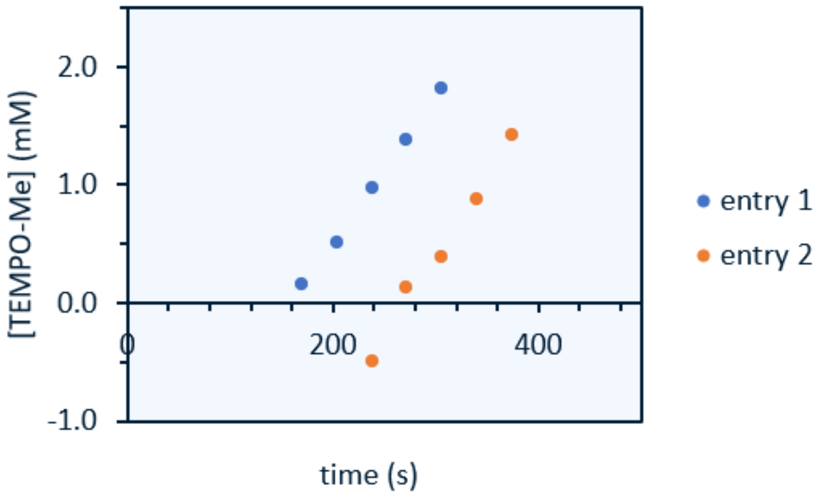

Figure S115. Overlaid plots of initial rates/linear regions of reactions from Table S4.

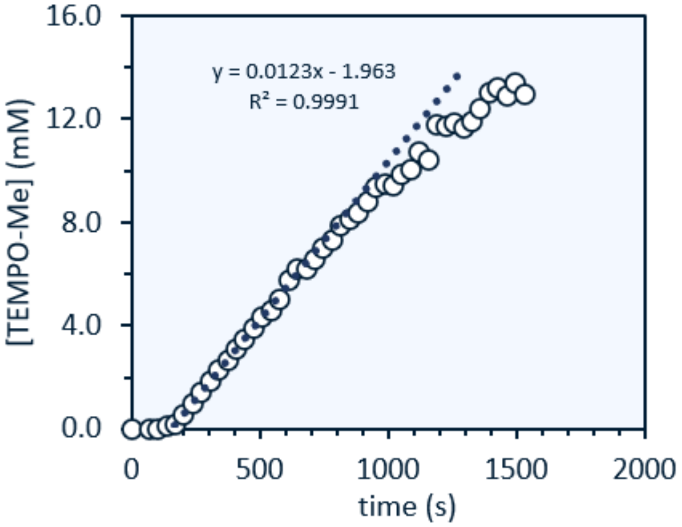

Figure S116. Initial rates of TEMPO-Me formation for irradiation of **1a** in the presence of excess TEMPO in C_6D_6. In this case $[\mathbf{1a}]_0$ = 120 mM, see Table S4 entry 1. A series of dark spectra were initially recorded, where the concentration of TEMPO-Me does not change. The initial rate was determined from the first few data points recorded after irradiation began.

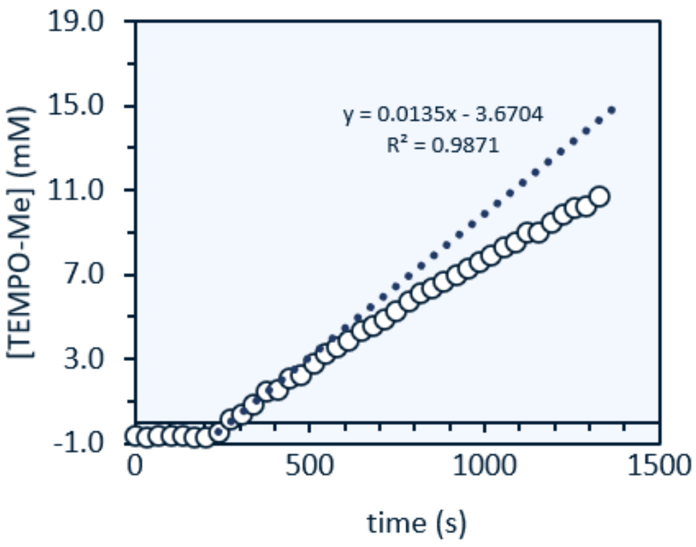

Figure S117. Initial rates of TEMPO-Me formation for irradiation of **1a** in the presence of excess TEMPO in MeCN-d_3. In this case [**1a**]$_0$ = 120 mM, see Table S4 entry 2. A series of dark spectra were initially recorded, where the concentration of TEMPO-Me does not change. The initial rate was determined from the first few data points recorded after irradiation began.

Quantum yield of Pd-C homolytic cleavage of 1a in the absence of TEMPO

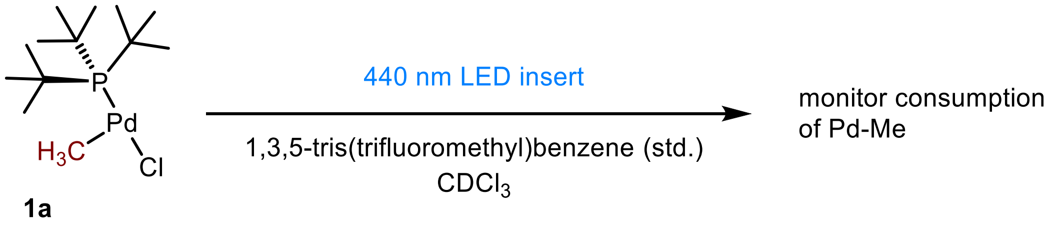

A solution of **1a** in $CDCl_3$ was prepared in a nitrogen filled glovebox at the desired concentration, along with 10 μL 1,3,5-tris(trifluoromethyl)benzene (std.). This solution was transferred to an NMR tube, a coaxial insert was inserted,[11] and the tube was sealed first with electrical tape and then parafilm. The tube was exported from the glovebox, the fiber optic cable was inserted, and the sample was placed in the spectrometer. An initial dark ^{1}H spectrum was recorded, then ^{1}H NMR spectra began to be continuously recorded. After several dark spectra were recorded, the light source was turned on (set to 1.0 of a maximum of 10) and spectra were continuously recorded for the duration of the experiment by the steady state technique. The initial rate of decay of Pd-Me was observed at two different initial concentrations of **1a**, one about twice as concentrated as the other. Essentially the same initial

rate was observed for both reactions, and so it was used to determine the zeroth order kinetic constant k_0. Applying (2), using the value of I_0 determined from the calibration using 2,4-DNBA, then allowed Φ to be determined.

Table S5. Initial rates of Pd-Me consumption at different initial concentrations of **1a**.

Entry	$[1a]_0$, mM	rate, M/s	Φ*
1	140	$1.26*10^{-5}$	0.79
2	80	$1.11*10^{-5}$	0.69

*Due to a change in the intensity of the light source over time, a different DNBA actinometric calibration had to be used for this experiments, *vide infra*.

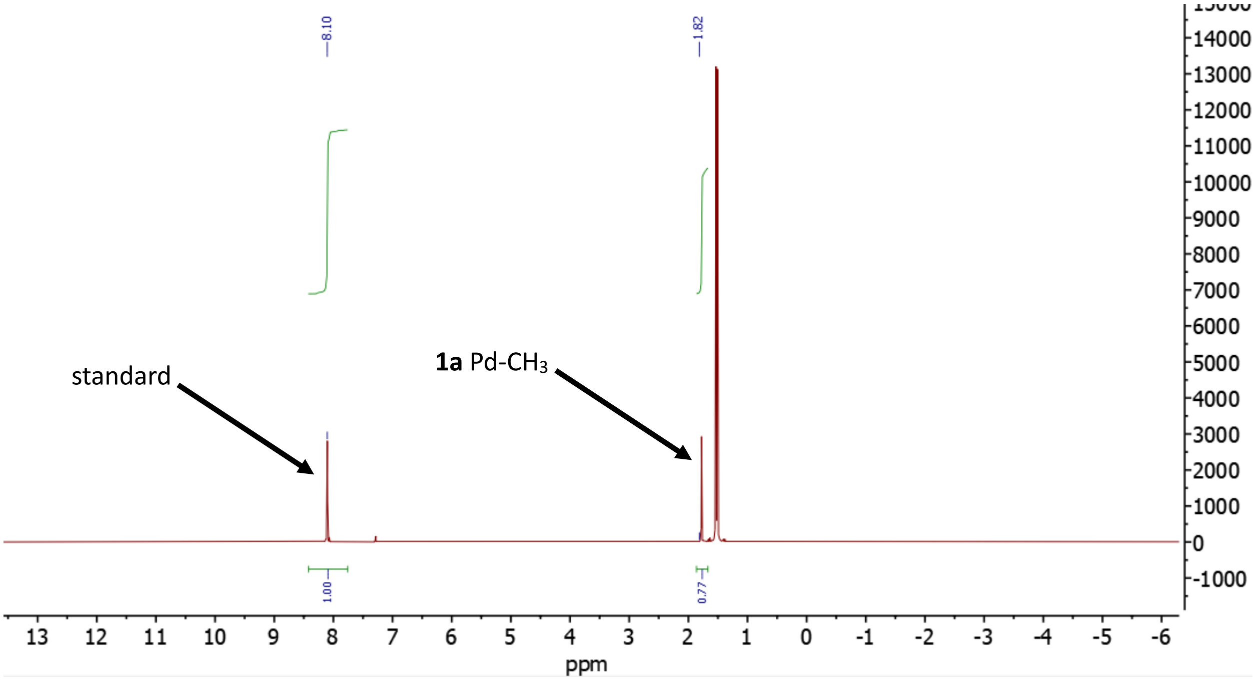

Figure S118. Initial ^{1}H spectrum from the reaction depicted in Figure S122.

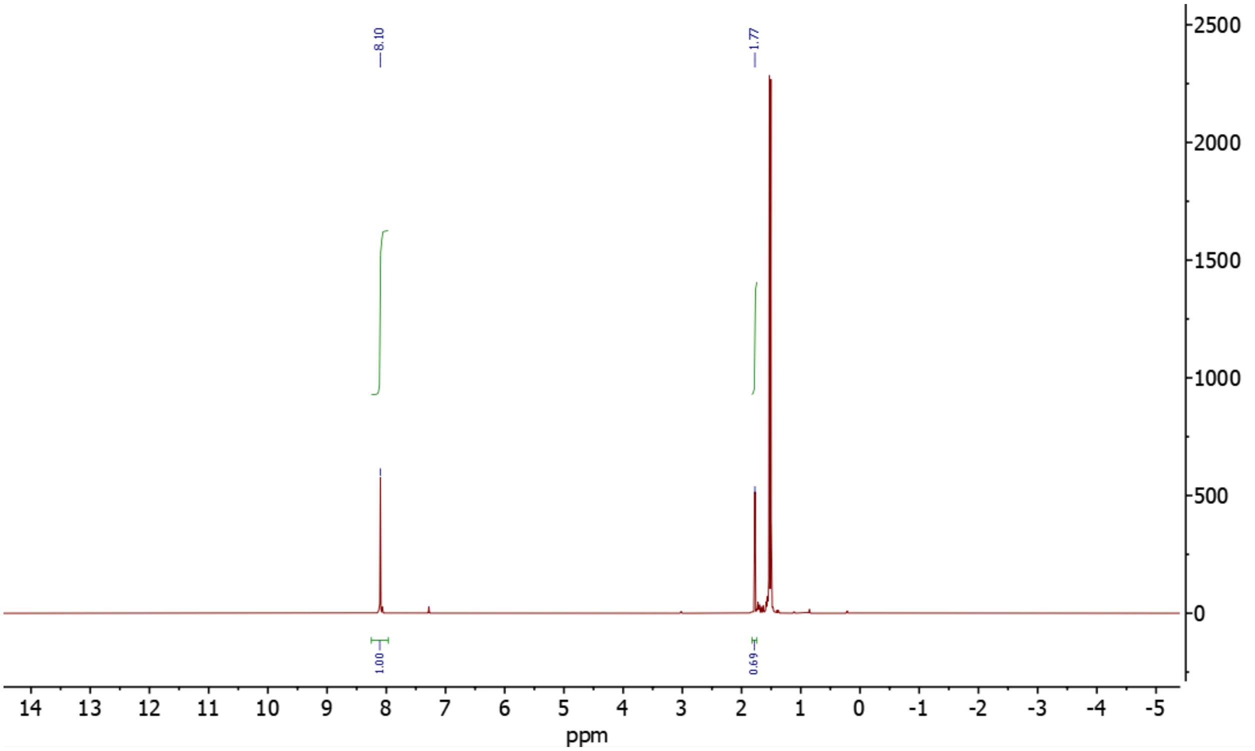

Figure S119. ^{1}H spectrum from the reaction depicted in Figure S122 after 1887 seconds of irradiation

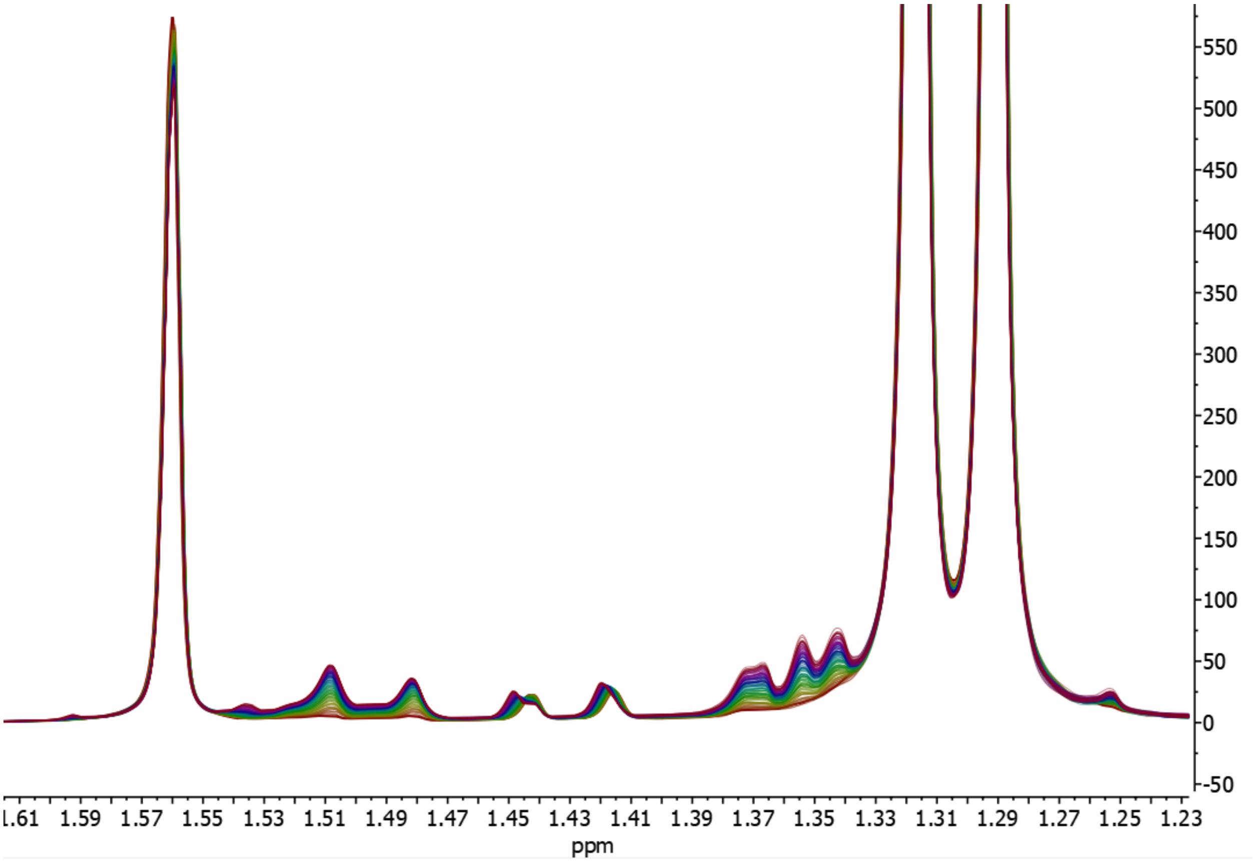

Figure S120. Detail of overlay of ^{1}H spectra from the reaction depicted in Figure S123 showing decrease of starting material peaks and formation of product peaks.

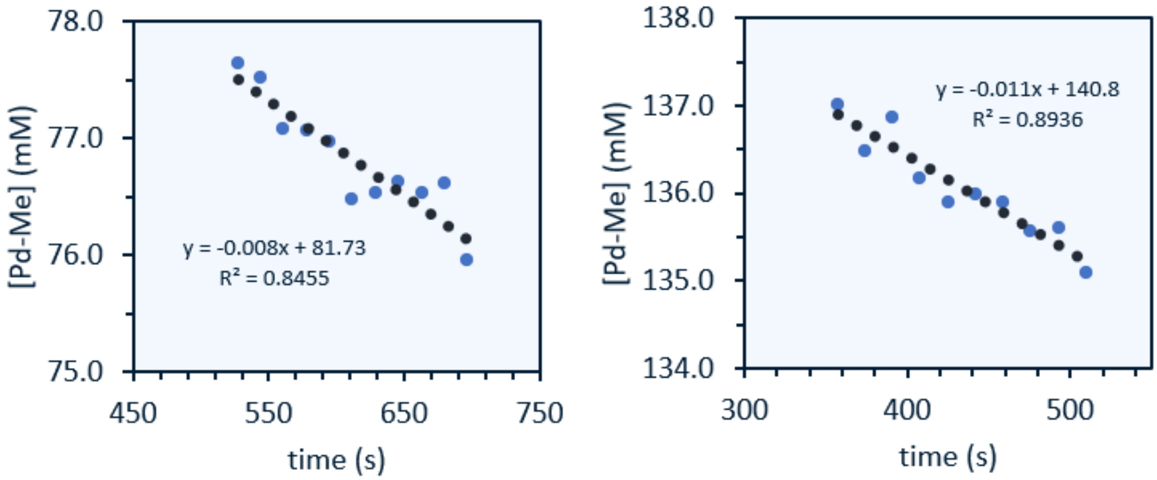

Figure S121. Linear regions used to calculate initial rates for reactions from Table S5: entry 1 where [**1a**]$_0$ = 80 mM (left) and entry 2 where [**1a**]$_0$ = 140 mM (right).

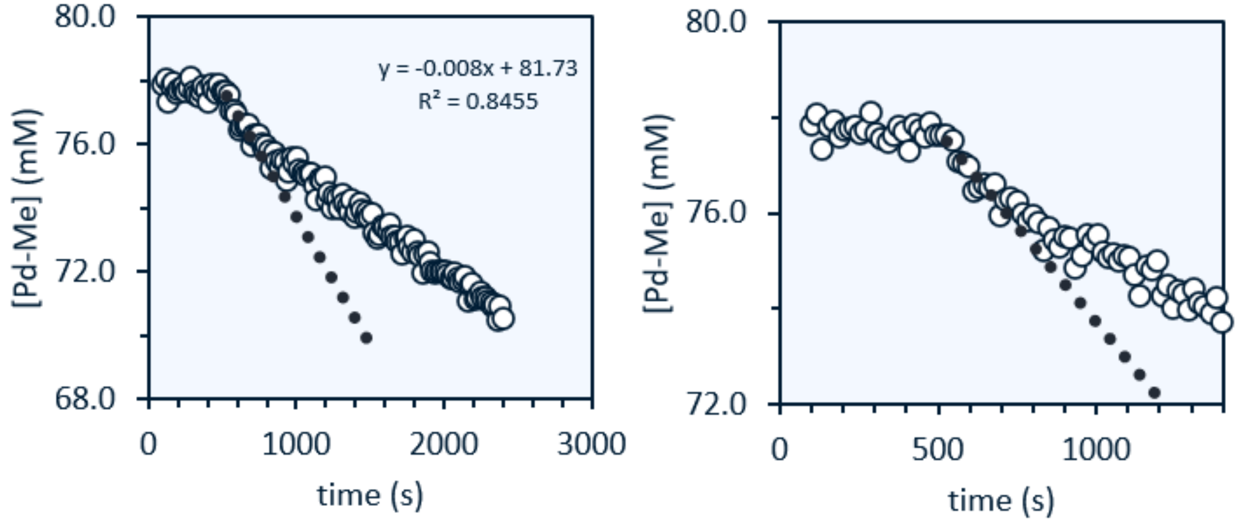

Figure S122. Initial rates of Pd-Me decay for irradiation of **1a**. In this case [**1a**]$_0$ = 80 mM. Left: full reaction course. Right: zoomed-in detail showing region used to determine initial rate.

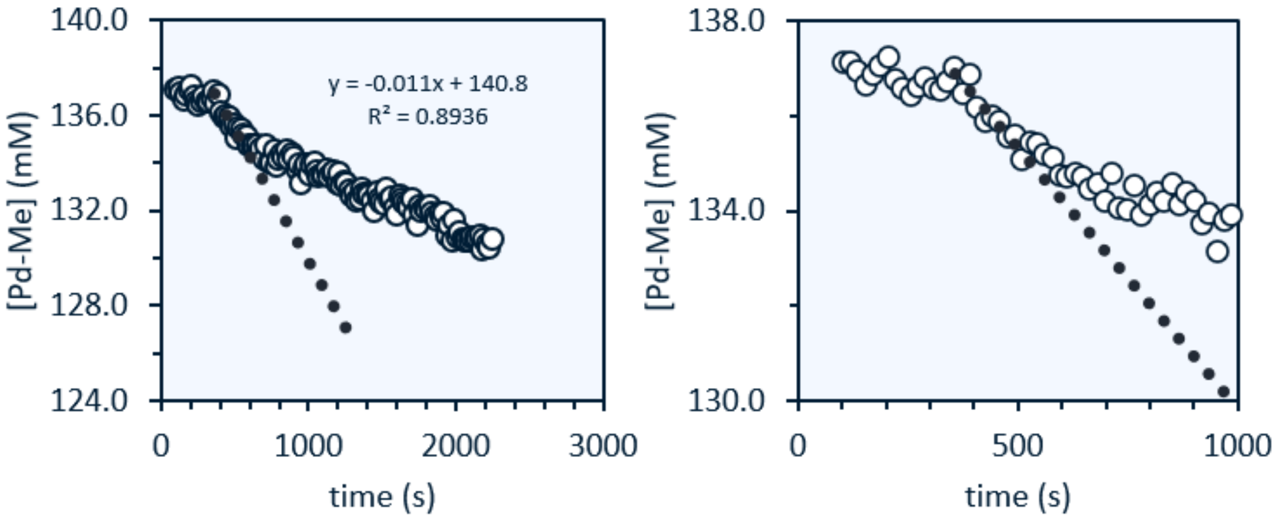

Figure S123. Initial rates of Pd-Me decay for irradiation of **1a**. In this case [**1a**]$_0$ = 140 mM. Left: full reaction course. Right: zoomed-in detail showing region used to determine initial rate.

For these data, it was determined that the intensity of the light source had changed compared to prior reactions. As such, a new calibration curve was prepared in the same way as before (see Table S2, Figures S96-S103, etc.)

Table S6. Initial rates of product formation at different initial concentrations of 2,4-DNBA.

[2,4-DNBA]$_0$, mM	rate, mM/min
49	0.0081
79	0.0117
122	0.0329
158	0.0301
233	0.0411
326	0.0588
320	0.0616

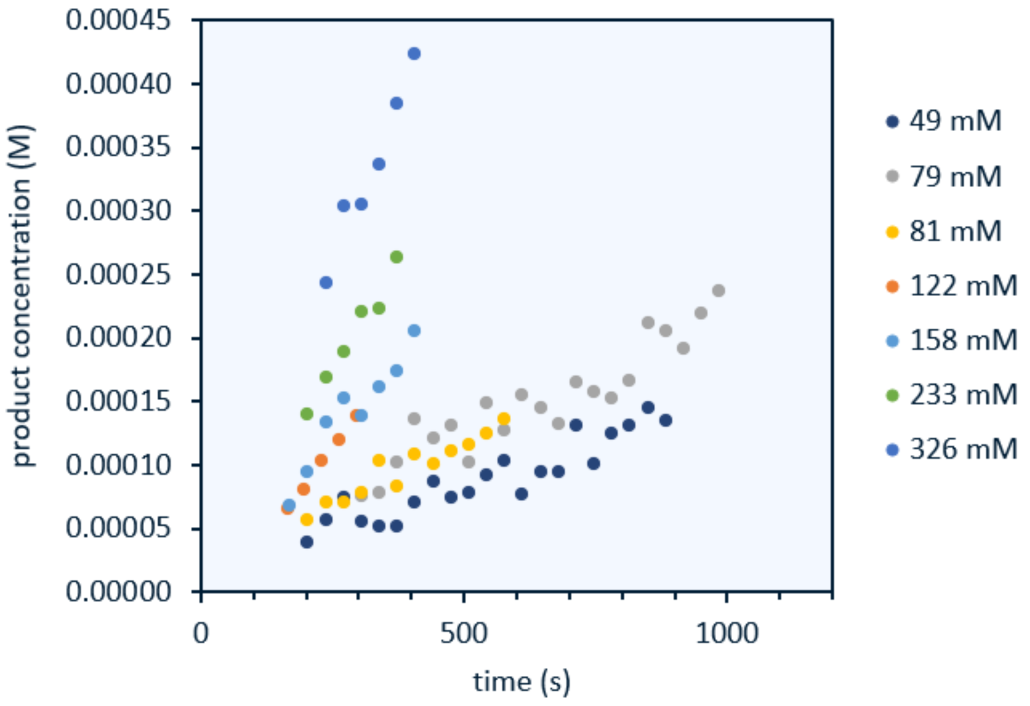

Figure S124. Overlaid plots of initial rates/linear regions of reactions of 2,4-DNBA with 440 nm light from Table S6. Legend indicates initial 2,4-DNBA concentration as determined by ^{1}H NMR.

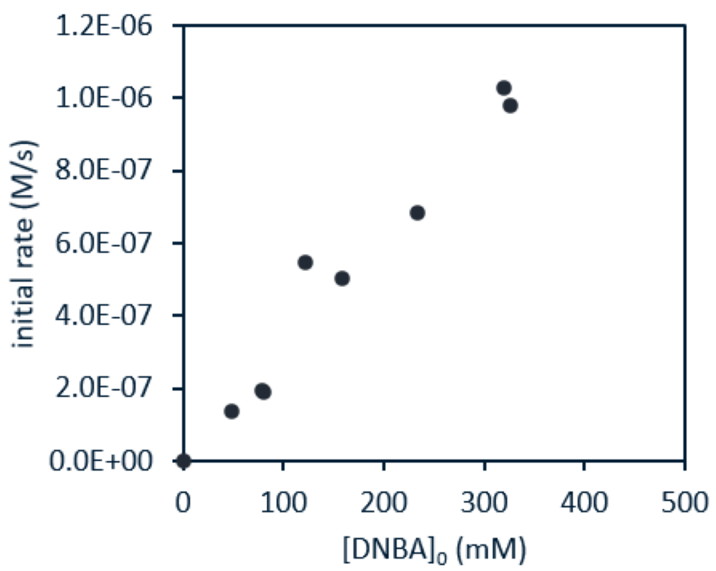

Figure S125. Plots of the initial rates of reaction of 2,4-DNBA at different concentrations for the second calibration curve using data from Table S6.

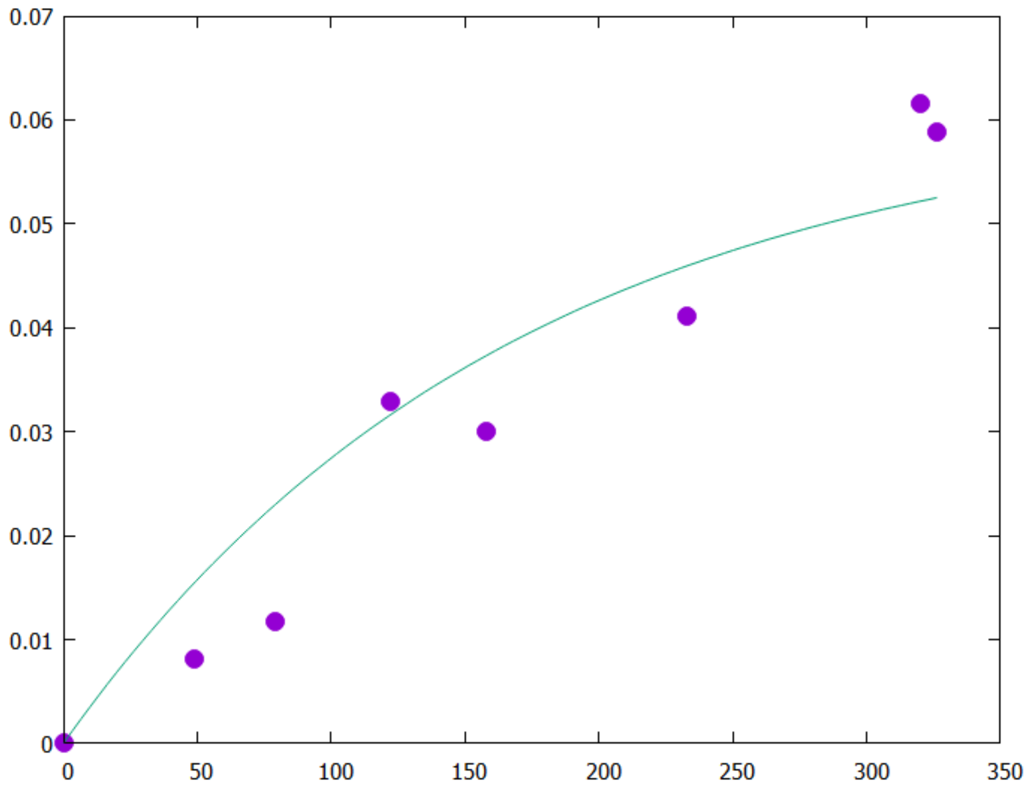

Figure S126. Second calibration curve (rate (mM/min) vs. concentration (mM)) for the fiber optic light source using data from Table S6, fit using gnuplot.

Analogously to the previous calibration curve, it was found that. $I_0 = 1.3*10^{-5}$ einstein $L^{-1}s^{-1}$ using (1). Averaging the initial rates of these reactions gives $k_0 = 9.5*10^{-6}$ M/s; this gives an average value of $\Phi = 0.73$ using (2).

Quantum yield of Pd-C homolytic cleavage of 2 in the presence of excess TEMPO

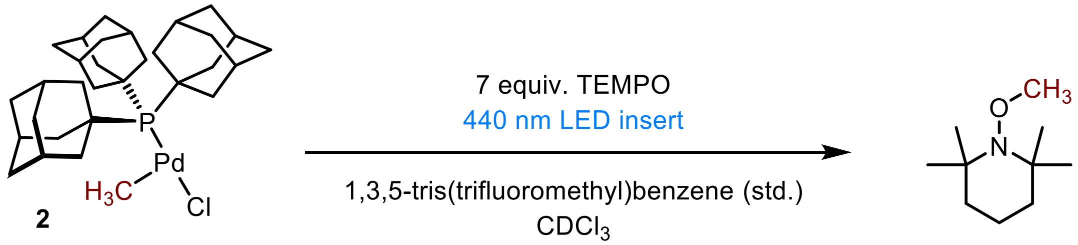

A solution of **2** at the desired concentration containing 7 equivalents of TEMPO and 10 µL 1,3,5-tris(trifluoromethyl)benzene (std.) in 490 µL $CDCl_3$ was prepared in a nitrogen filled glovebox. This solution was transferred to an NMR tube, a coaxial insert was inserted,[11] and the tube was sealed first with electrical tape and then parafilm. The tube was exported from the glovebox, the fiber optic cable was inserted, and the sample was placed in the spectrometer. An initial dark ^{1}H spectrum was recorded, then ^{1}H NMR spectra began to be continuously recorded. After several dark spectra were recorded, the light source was turned on (set to 1.0 of a maximum of 10) and spectra were continuously recorded for the duration of the experiment by the steady state technique. The initial rate of TEMPO-Me formation was observed at two different initial concentrations of **2**, one about twice as concentrated as the other. Essentially the same initial rate was observed for both reactions, and so it was used to determine the zeroth order kinetic constant k_0. Applying (2), using the value of I_0 determined from the calibration using 2,4-DNBA, then allowed Φ to be determined.

Table S7. Initial rates of TEMPO-Me formation at different initial concentrations of **2**.

Entry	$[2]_0$, mM	rate, M/s	Φ
1	60	$6.7*10^{-6}$	0.37
2	30	$6.4*10^{-6}$	0.36

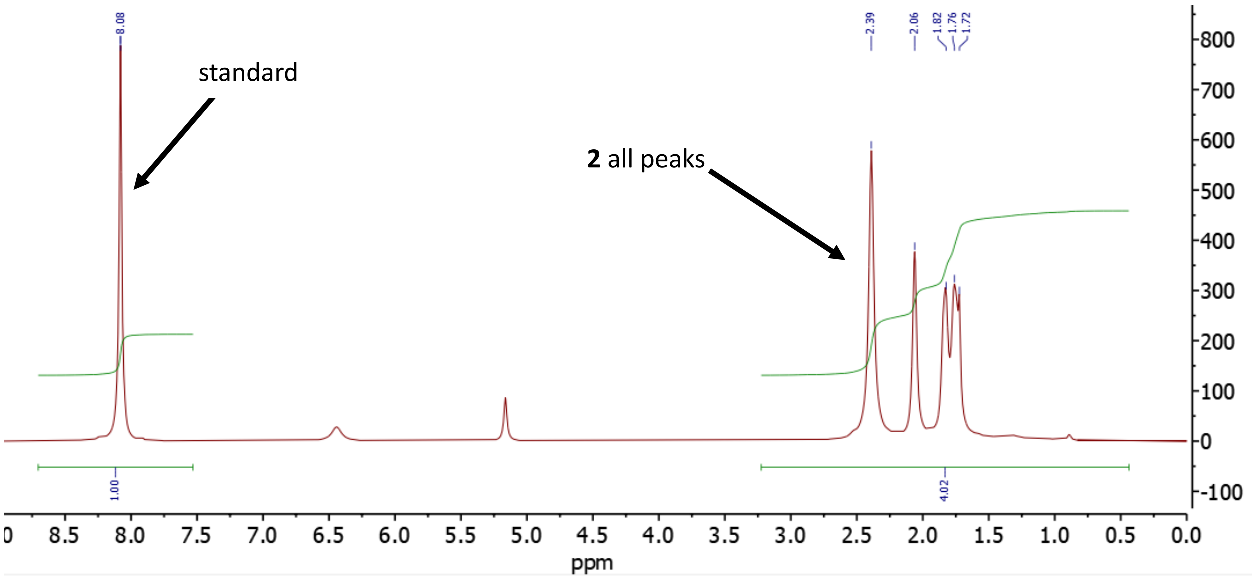

Figure S127. Initial ^{1}H spectrum from the reaction depicted in Figure S132.

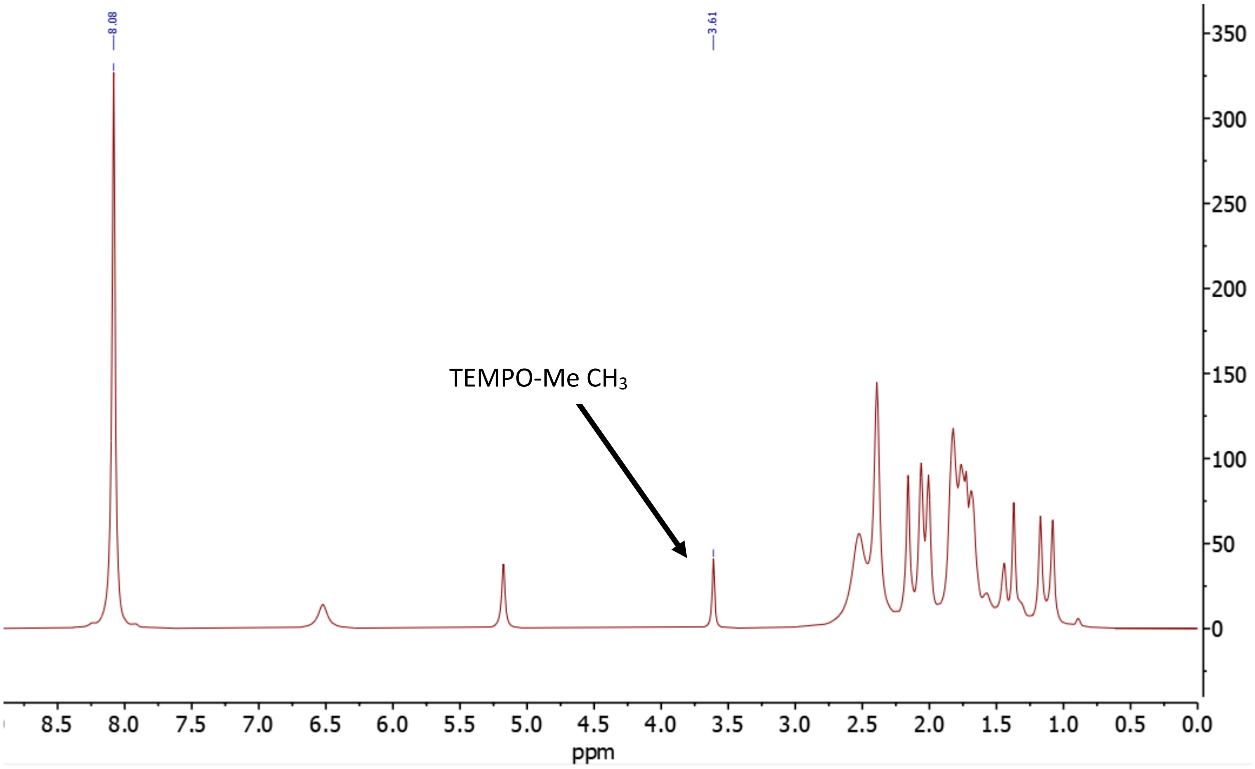

Figure S128. ^{1}H spectrum from the reaction depicted in Figure S132 after 3828 seconds of irradiation showing product peak.

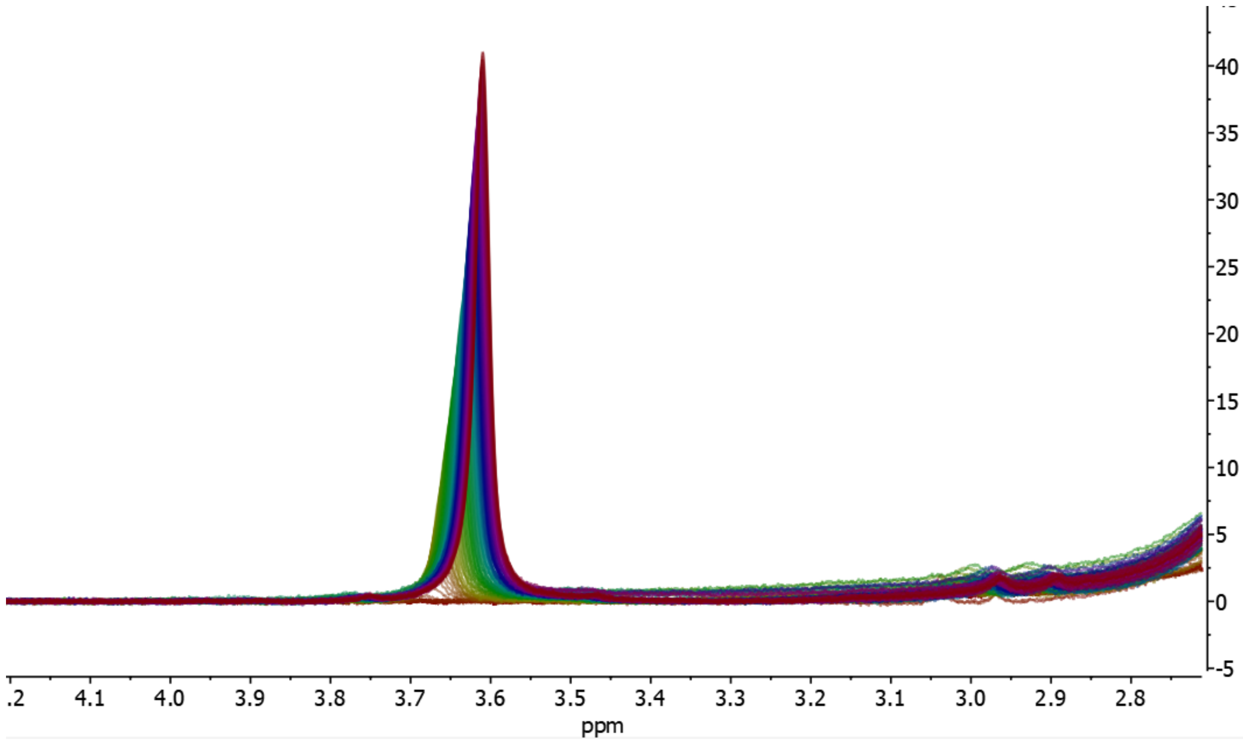

Figure S129. Overlay of ^{1}H spectra from the reaction depicted in Figure S132 showing evolution of product peak over time.

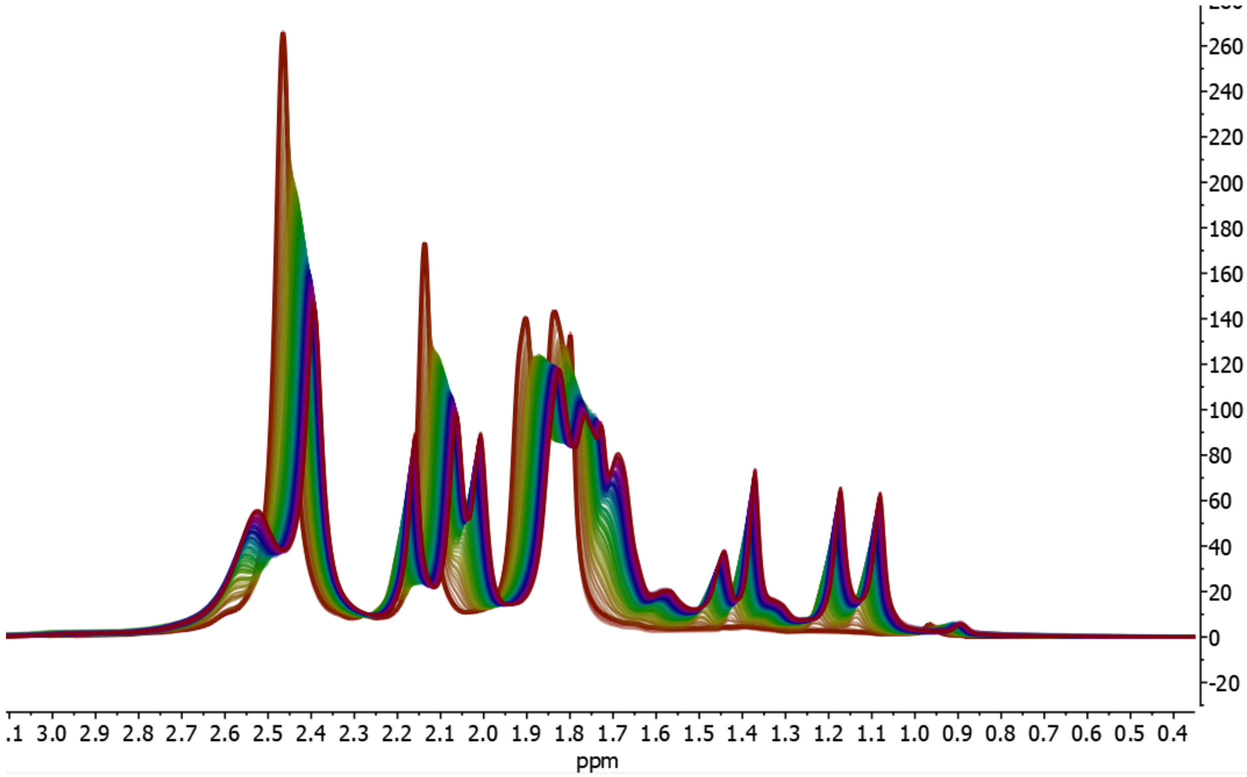

Figure S130. Overlay of ^{1}H spectra from the reaction depicted in Figure S132 showing

decrease of starting material peaks over time.

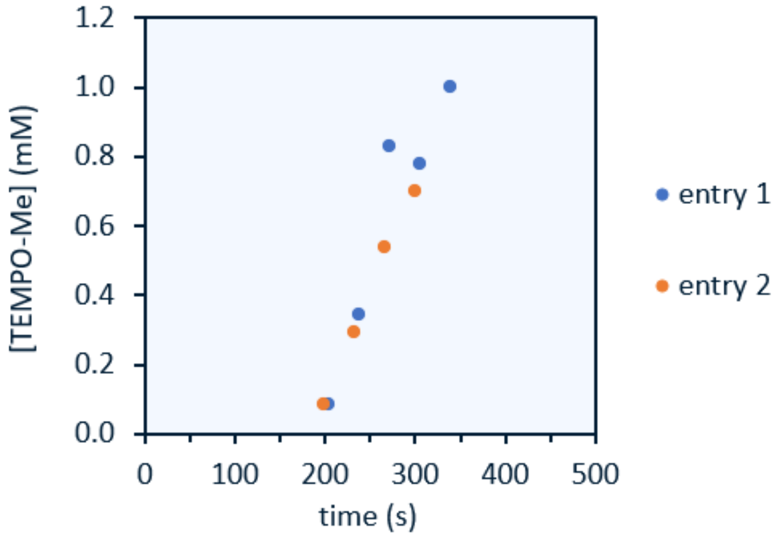

Figure S131. Overlaid plots of initial rates/linear regions of reactions from Table S7.

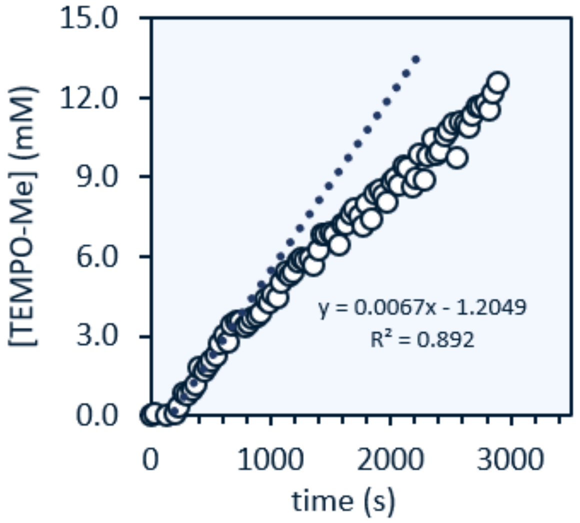

Figure S132. Initial rates of TEMPO-Me formation for irradiation of **2** in the presence of excess TEMPO. In this case [**2**]$_0$ = 50 mM. A series of dark spectra were initially recorded, where the concentration of TEMPO-Me does not change. The initial rate was determined from the first few data points recorded after irradiation began.

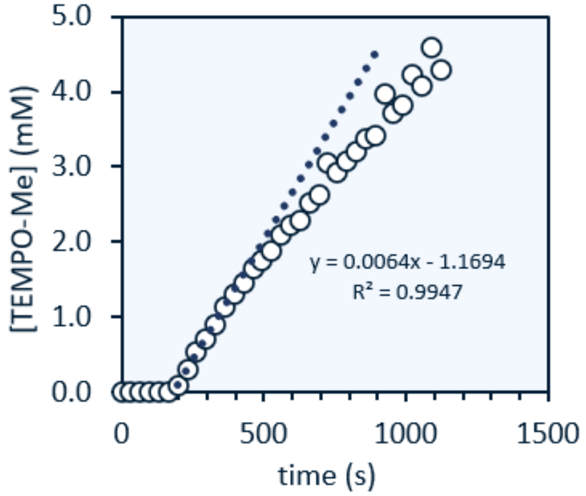

Figure S133. Initial rates of TEMPO-Me formation for irradiation of **2** in the presence of excess TEMPO. In this case $[\mathbf{2}]_0$ = 30 mM. A series of dark spectra were initially recorded, where the concentration of TEMPO-Me does not change. The initial rate was determined from the first few data points recorded after irradiation began.

The data are shown above Averaging the initial rates of these reactions gives $k_0 = 6.6*10^{-6}$ M/s. Given $I_0 = 1.8*10^{-5}$ einstein $L^{-1}s^{-1}$, this gives an average value of $\Phi = 0.36$.

Quantum yield of Pd-C homolytic cleavage of 3 in the presence of excess TEMPO

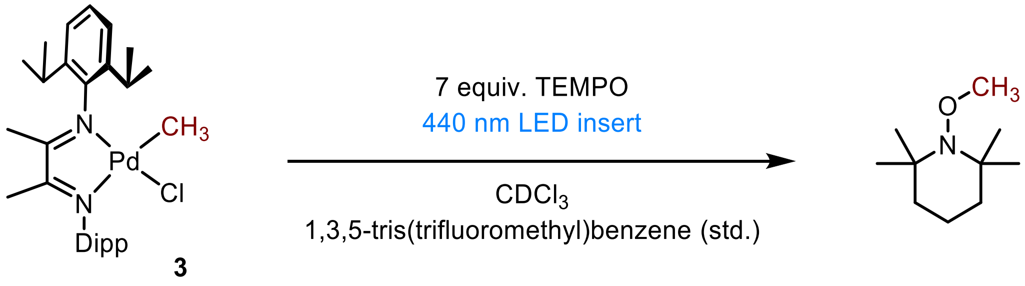

A solution of **3** at the desired concentration containing 7 equivalents of TEMPO and 10 μL 1,3,5-tris(trifluoromethyl)benzene (std.) in 490 μL $CDCl_3$ was prepared in a nitrogen filled glovebox. This solution was transferred to an NMR tube, a coaxial insert was inserted,[11] and the tube was sealed first with electrical tape and then parafilm. The tube was exported from the glovebox, the fiber optic cable was inserted, and the sample was placed in the spectrometer. An initial dark ^1H spectrum was recorded, then ^1H NMR spectra began to be continuously

recorded. After several dark spectra were recorded, the light source was turned on (set to 1.0 of a maximum of 10) and spectra were continuously recorded for the duration of the experiment by the steady state technique. The initial rate of TEMPO-Me formation was observed at two different initial concentrations of **3**, one about twice as concentrated as the other. Essentially the same initial rate was observed for both reactions, and so it was used to determine the zeroth order kinetic constant k_0 (see Figure S139 to S142). Applying (2), using the value of I_0 determined from the calibration using 2,4-DNBA, then allowed Φ to be determined.

Table S8. Initial rates of TEMPO-Me formation at different initial concentrations of **3**, several runs

Entry	$[3]_0$, mM	rate, M/s	Φ
1	60	$2.1*10^{-6}$	0.12
2	30	$1.8*10^{-6}$	0.10
3	30	$1.6*10^{-6}$	0.08
4	30	$1.9*10^{-6}$	0.11

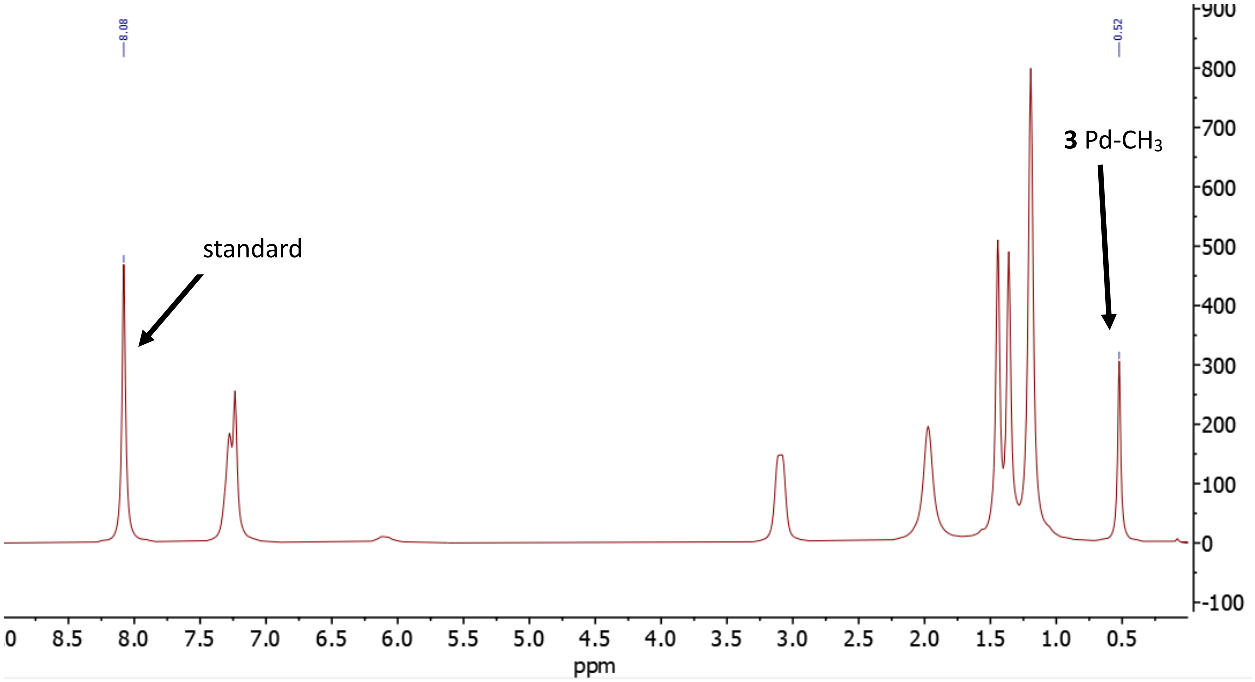

Figure S134. Initial ^{1}H spectrum of representative reaction from Table S8 (entry 3).

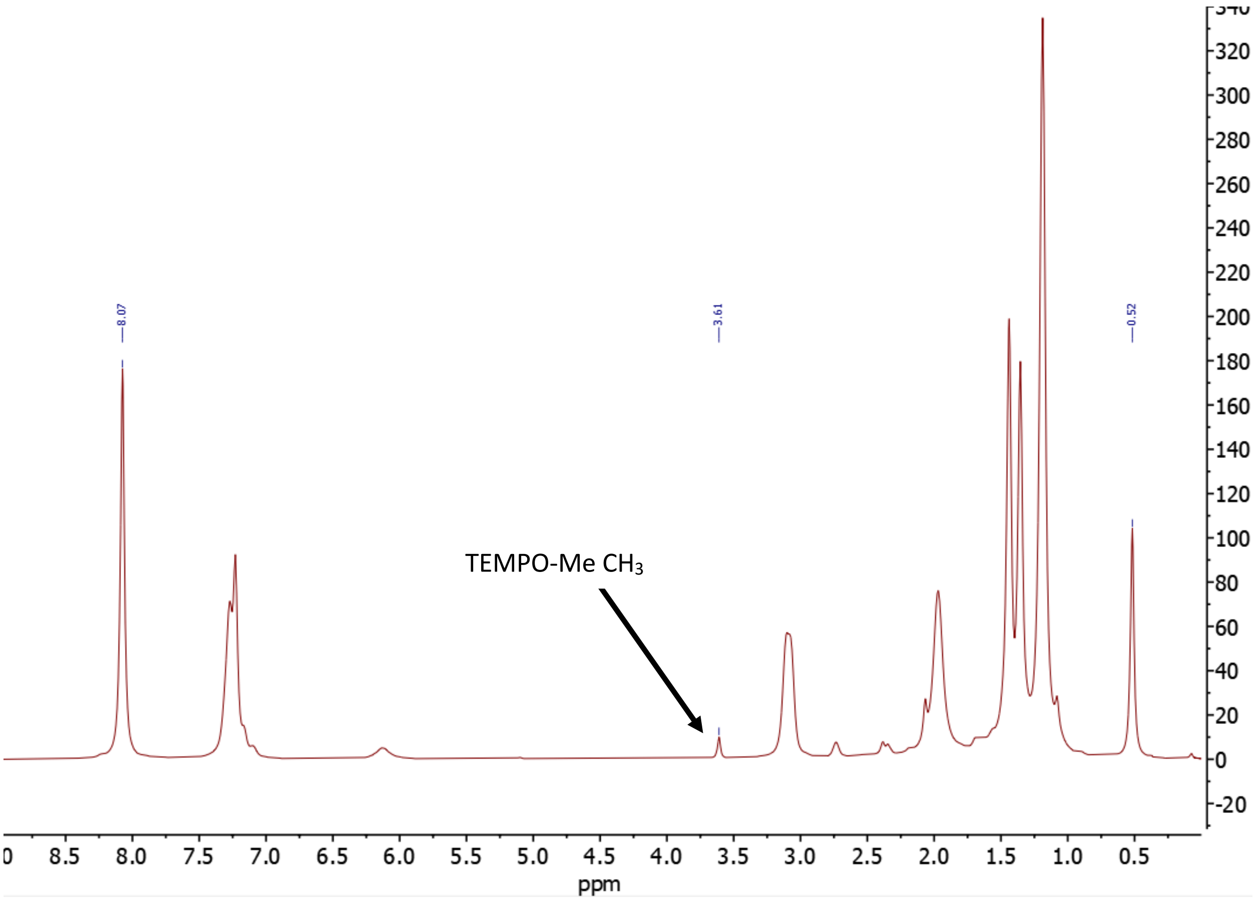

Figure S135. ^{1}H spectrum of representative reaction from Table S8 (entry 3) after 2380 seconds of irradiation showing product peak.

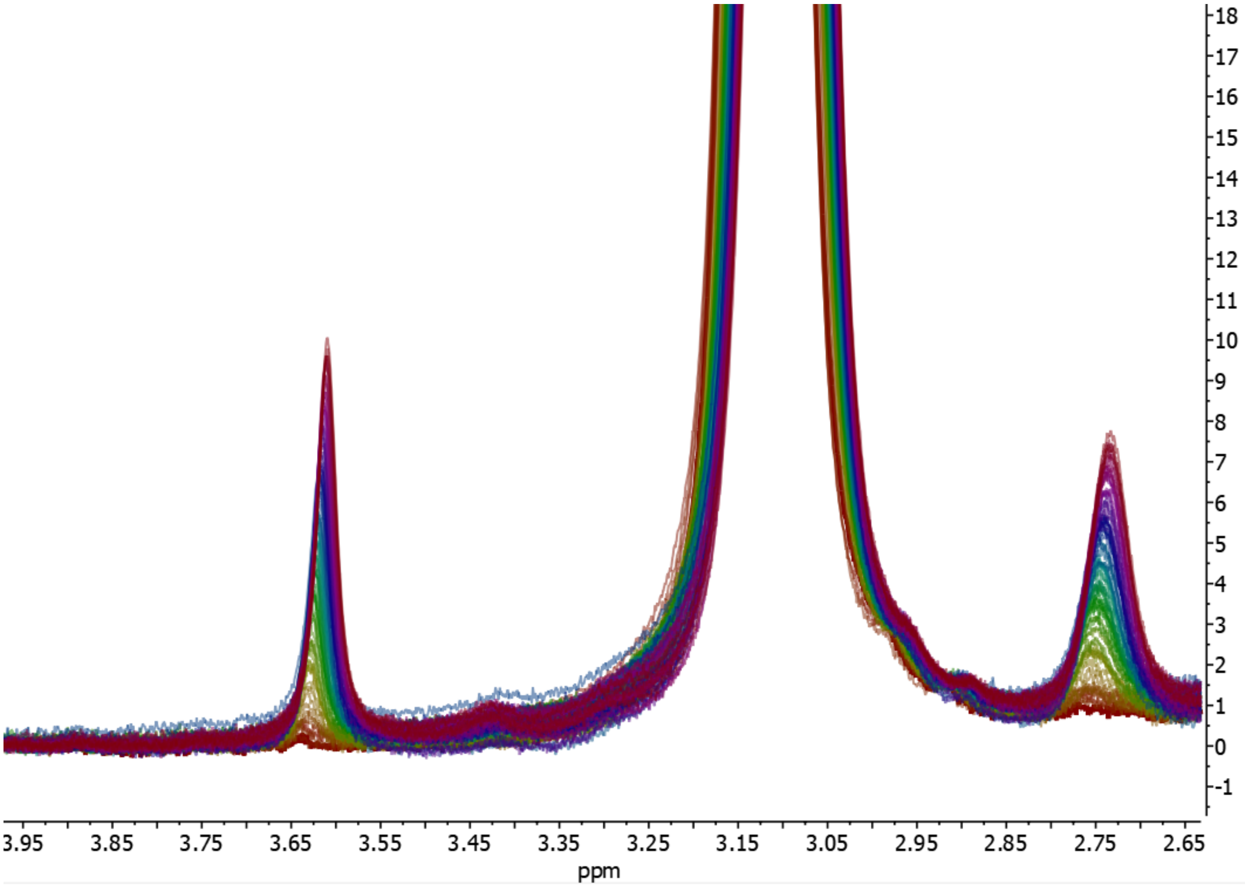

Figure S136. Overlay of ^{1}H spectra from Table S8 entry 3 showing evolution of product peak over time.

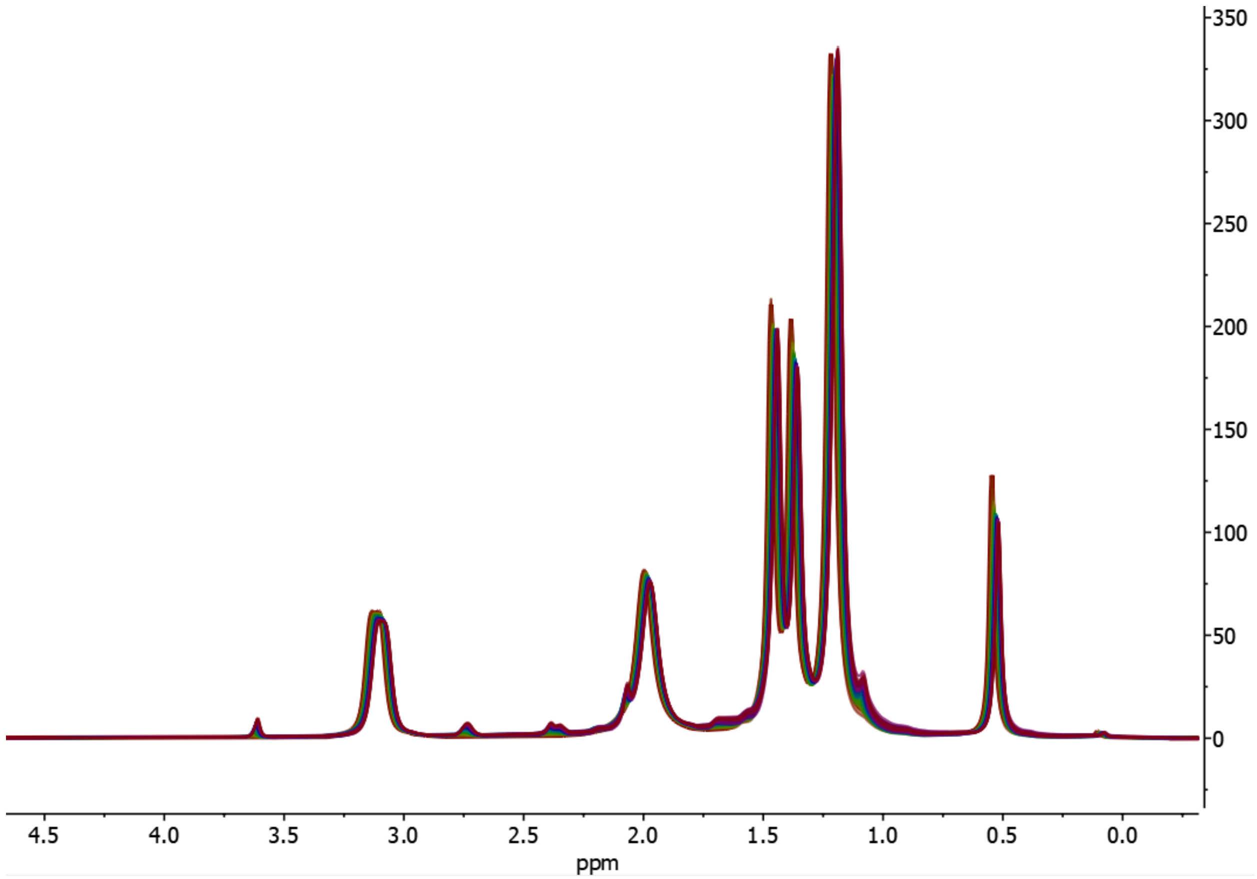

Figure S137. Overlay of ^{1}H spectra from Table S8 entry 3 showing decrease of starting material peaks over time.

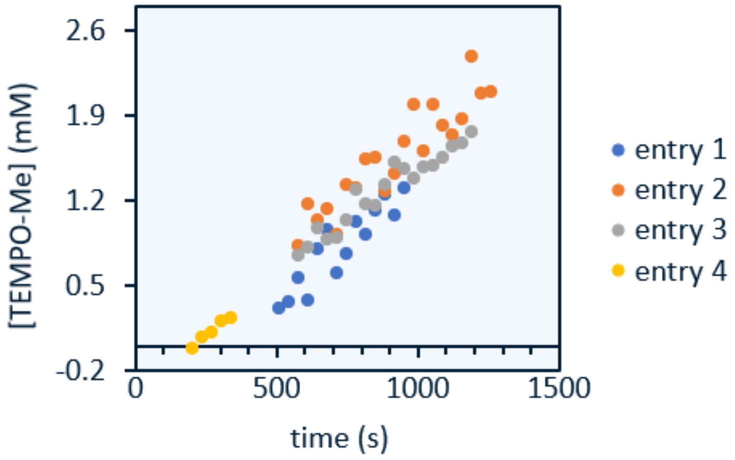

Figure S138. Overlaid plots of initial rates/linear regions of reactions from Table S8.

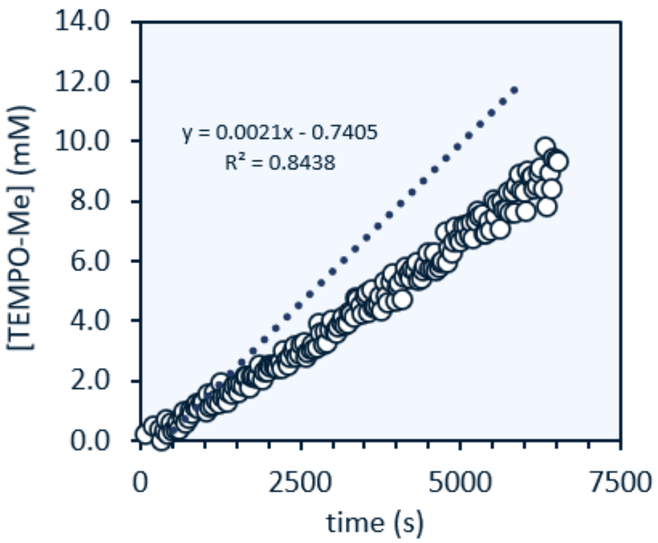

Figure S139. Initial rates of TEMPO-Me formation for irradiation of **3** in the presence of excess TEMPO. In this case $[\mathbf{3}]_0 = 60$ mM, see Table S8 entry 1. A series of dark spectra were initially recorded, where the concentration of TEMPO-Me does not change. The initial rate was determined from the first few data points recorded after irradiation began.

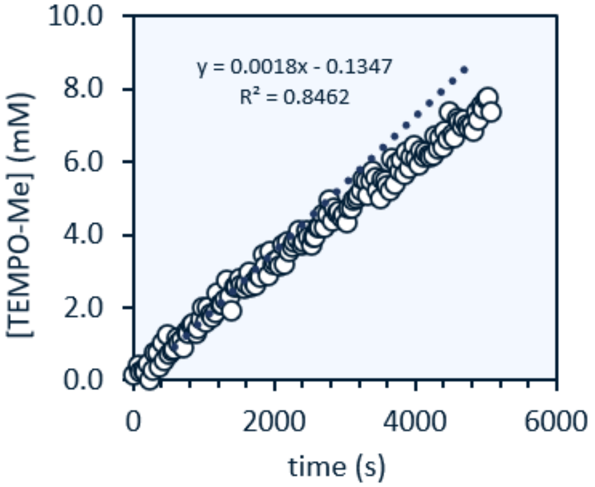

Figure S140. Initial rates of TEMPO-Me formation for irradiation of **3** in the presence of excess TEMPO. In this case $[\mathbf{3}]_0 = 30$ mM, see Table S8 entry 2. A series of dark spectra were initially recorded, where the concentration of TEMPO-Me does not change. The initial rate was determined from the first few data points recorded after irradiation began.

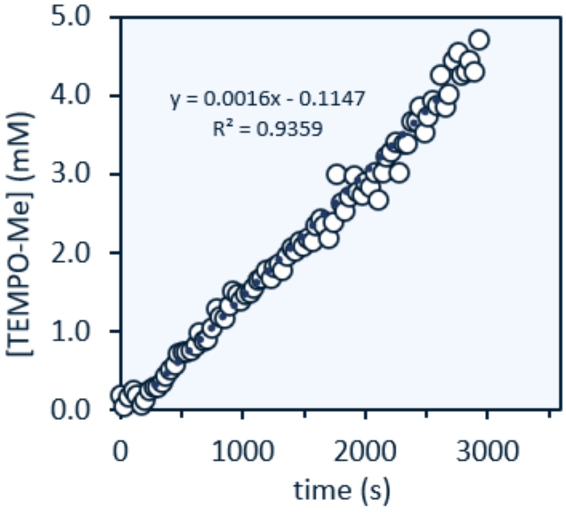

Figure S141. Initial rates of TEMPO-Me formation for irradiation of **3** in the presence of excess TEMPO. In this case $[\mathbf{3}]_0$ = 30 mM, see Table S8 entry 3. A series of dark spectra were initially recorded, where the concentration of TEMPO-Me does not change. The initial rate was determined from the first few data points recorded after irradiation began.

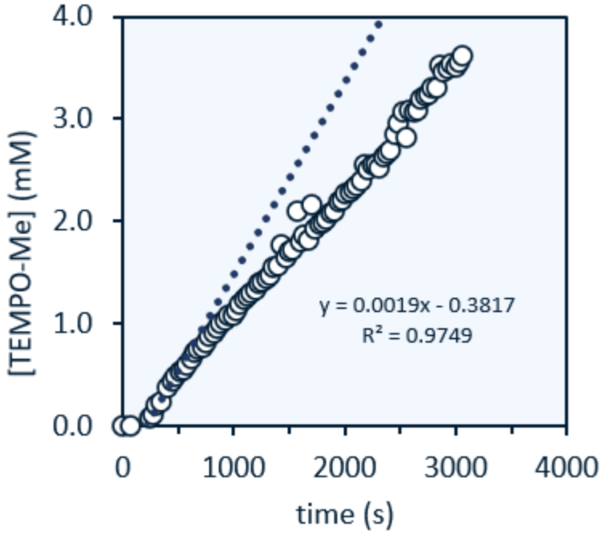

Figure S142. Initial rates of TEMPO-Me formation for irradiation of **3** in the presence of excess TEMPO. In this case $[\mathbf{3}]_0$ = 30 mM, see Table S8 entry 4. A series of dark spectra were initially recorded, where the concentration of TEMPO-Me does not change. The initial rate was determined from the first few data points recorded after irradiation began.

Here, k_0 was determined by the average of several runs at different concentrations. The data are shown above. Averaging the initial rates of these reactions gives $k_0 = 1.9*10^{-6}$ M/s with a standard deviation of $2*10^{-7}$ M/s. Given $I_0 = 1.8*10^{-5}$ einstein $L^{-1}s^{-1}$, this gives an average value of $\Phi = 0.11$ with a standard deviation of 0.01.

Quantum yield of Pd-C homolytic cleavage of 1a in the presence of excess TEMPO in solvents other than $CDCl_3$

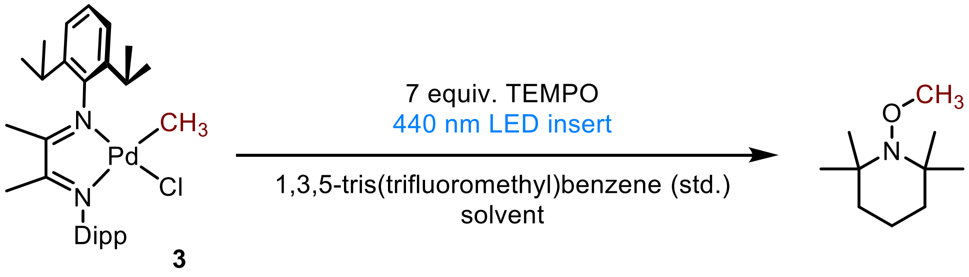

A solution of **3** of the desired concentration containing 7 equivalents of TEMPO and 10 µL 1,3,5-tris(trifluoromethyl)benzene (std.) in 490 µL of the desired solvent was prepared in a nitrogen filled glovebox. This solution was transferred to an NMR tube, a coaxial insert was inserted,[11] and the tube was sealed first with electrical tape and then parafilm. The tube was exported from the glovebox, the fiber optic cable was inserted, and the sample was placed in the spectrometer. An initial dark ^{1}H spectrum was recorded, then ^{1}H NMR spectra began to be continuously recorded. After several dark spectra were recorded, the light source was turned on (set to 1.0 of a maximum of 10) and spectra were continuously recorded for the duration of the experiment by the steady state technique. Essentially the same initial rate was observed for both reactions, and so it was used to determine the zeroth order kinetic constant k_0. Applying (2), using the value of I_0 determined from the calibration using 2,4-DNBA, then allowed Φ to be determined.

Table S9. Initial rates of TEMPO-Me formation at different initial concentrations of **1a**, two runs.

Entry	$[3]_0$, mM	Solvent	rate, M/s	Φ
1	30	MeCN-d_3	$2.5*10^{-6}$	0.14
2	60	MeCN-d_3	$3.0*10^{-6}$	0.17

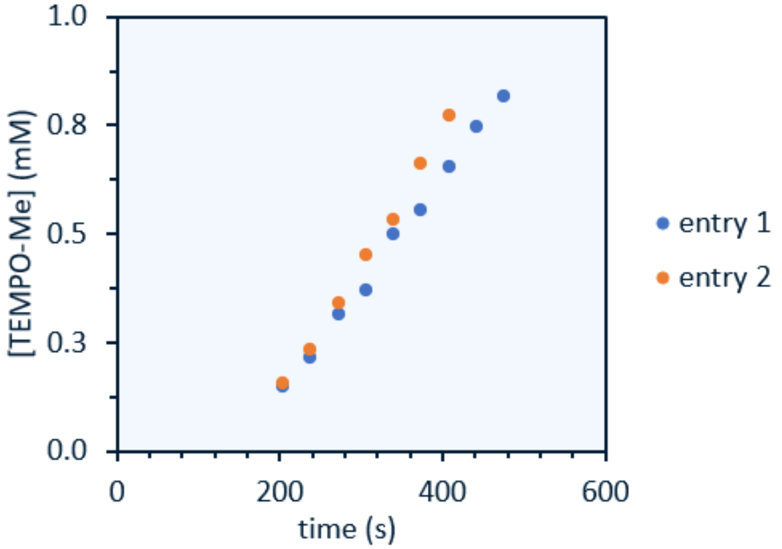

Figure S143. Overlaid plots of initial rates/linear regions of reactions from Table S9.

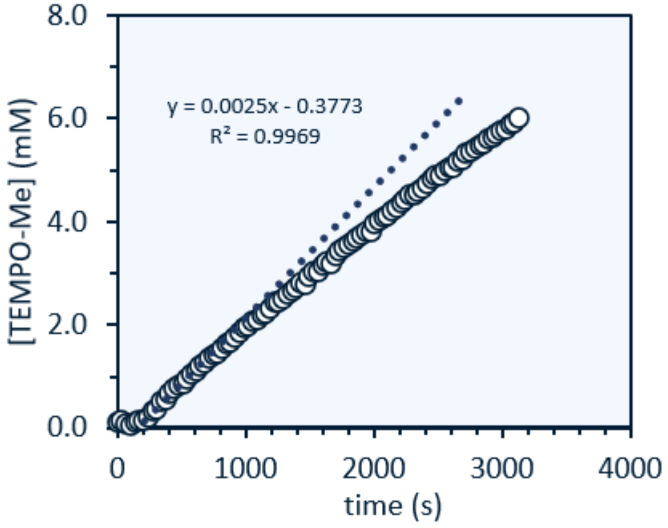

Figure S144. Initial rates of TEMPO-Me formation for irradiation of **3** in the presence of excess TEMPO in MeCN-d_3. In this case [**3**]$_0$ = 30 mM, see Table S9 entry 1. A series of dark spectra were initially recorded, where the concentration of TEMPO-Me does not change. The initial rate was determined from the first few data points recorded after irradiation began.

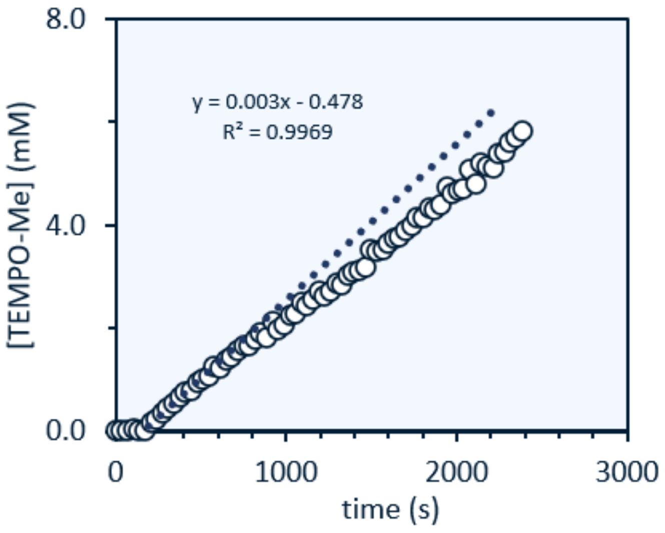

Figure S145. Initial rates of TEMPO-Me formation for irradiation of **3** in the presence of excess TEMPO in MeCN-d_3. In this case $[\mathbf{3}]_0$ = 60 mM, see Table S9 entry 2. A series of dark spectra were initially recorded, where the concentration of TEMPO-Me does not change. The initial rate was determined from the first few data points recorded after irradiation began.

Quantum yield of Pd-C homolytic cleavage of 3 in the absence of TEMPO

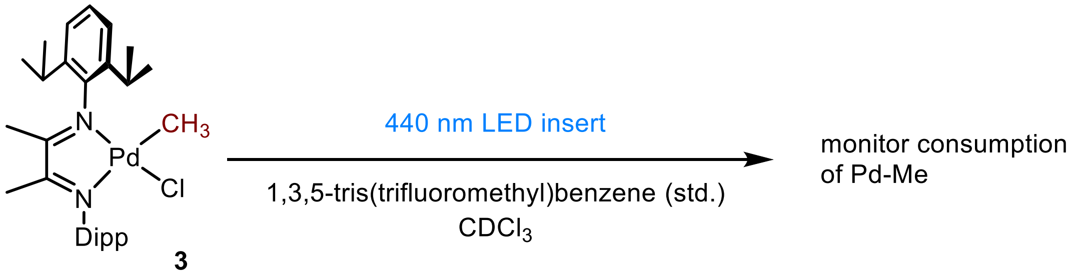

A solution of **3** at the desired concentration and 10 µL 1,3,5-tris(trifluoromethyl)benzene (std.) in 490 µL $CDCl_3$was prepared in a nitrogen filled glovebox. This solution was transferred to an NMR tube, a coaxial insert was inserted,[11] and the tube was sealed first with electrical tape and then parafilm. The tube was exported from the glovebox, the fiber optic cable was inserted, and the sample was placed in the spectrometer. An initial dark ^{1}H spectrum was recorded, then ^{1}H NMR spectra began to be continuously recorded. After several dark spectra were recorded, the light source was turned on (set to 1.0 of a maximum of 10) and spectra were continuously recorded for the duration of the experiment by the steady state technique. The initial rate of decay of Pd-Me was observed at two different initial concentrations of **3**, one about twice as

concentrated as the other. Essentially the same initial rate was observed for both reactions, and so it was used to determine the zeroth order kinetic constant k_0 (see Figure S149 and S150). Applying (2), using the value of I_0 determined from the calibration using 2,4-DNBA, then allowed Φ to be determined.

Table S10. Initial rates of Pd-Me consumption at different initial concentrations of **3**.

Entry	$[3]_0$, mM	rate, M/s	Φ
1	60	$3.0*10^{-6}$	0.17
2	30	$2.7*10^{-5}$	0.15

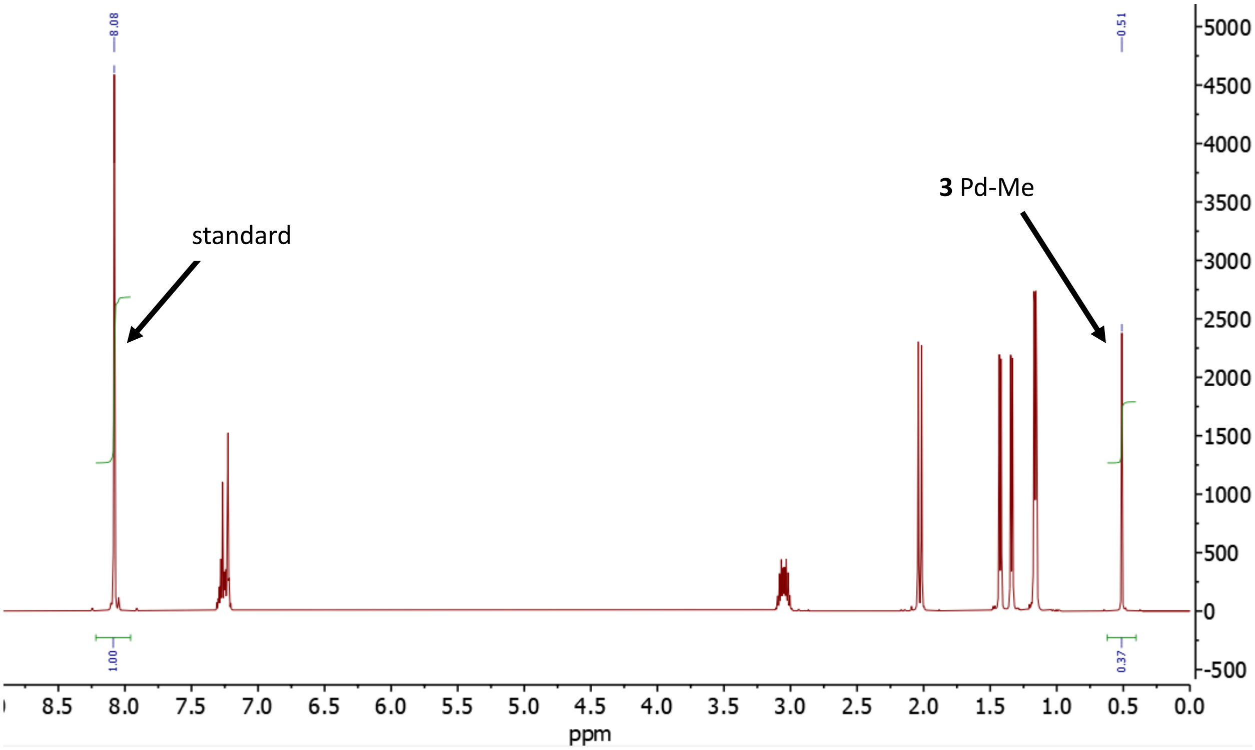

Figure S146. Initial ^{1}H spectrum from the reaction depicted in Figure S149.

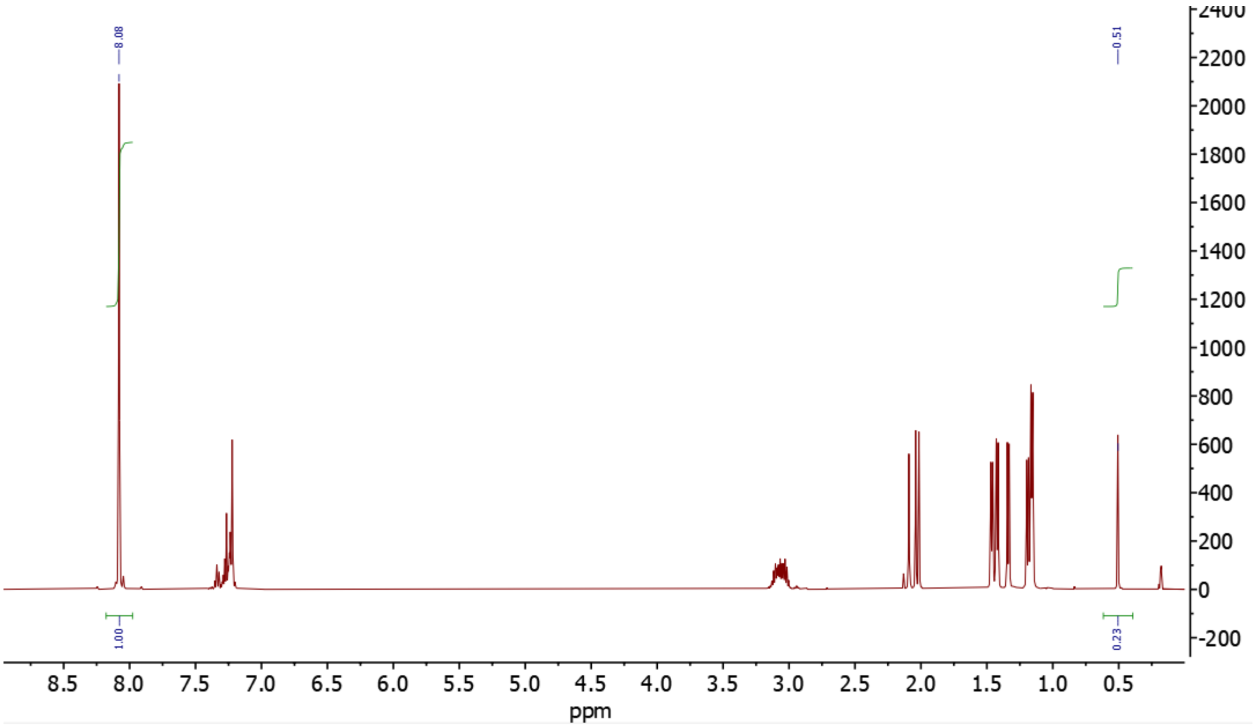

Figure S147. ¹H spectrum from the reaction depicted in Figure S149 after 4998 seconds of irradiation showing product peaks.

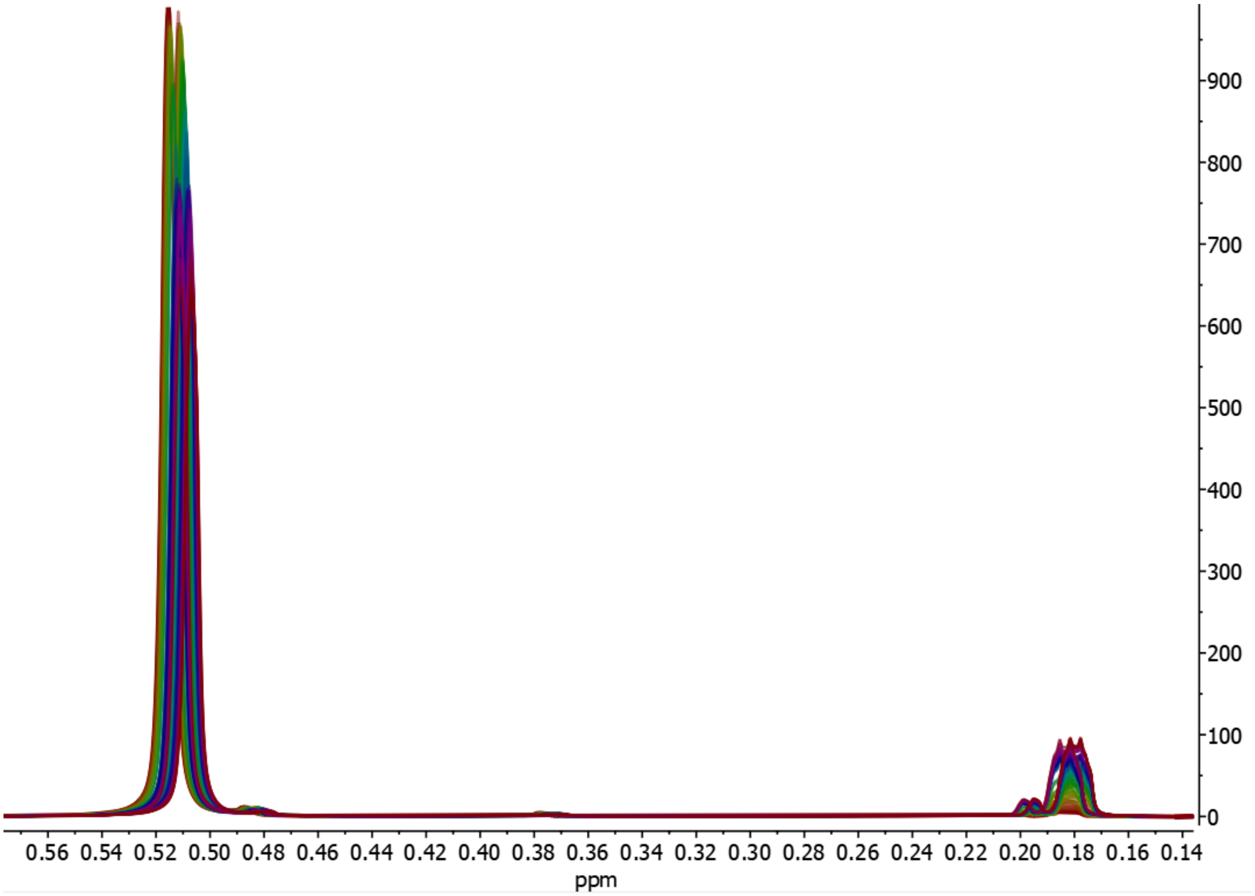

Figure S148. Overlay of ^{1}H spectra from the reaction depicted in Figure S149 showing decrease of starting material peaks and growth of the product peaks over time.

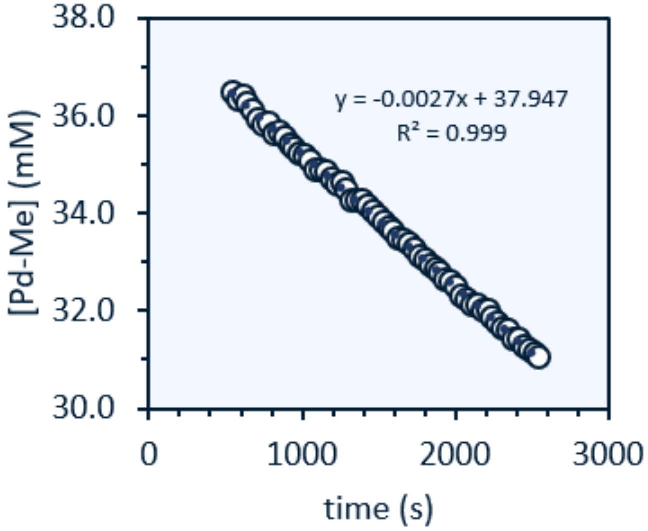

Figure S149. Initial rates of Pd-Me decay for irradiation of **3**. In this case $[\mathbf{3}]_0$ = 40 mM. Since

exceptionally linear behavior was observed, all the visible data points in the plot were used to fit the initial rate line.

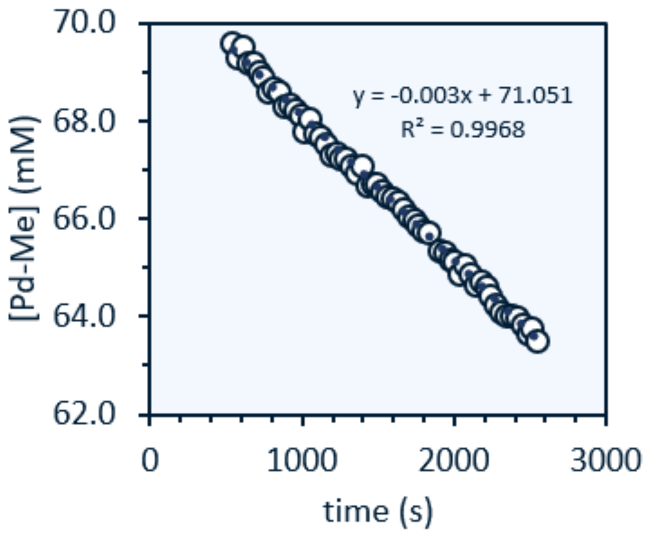

Figure S150. Initial rates of Pd-Me decay for irradiation of **3**. In this case $[\mathbf{3}]_0$ = 70 mM. Since exceptionally linear behavior was observed, all the visible data points in the plot were used to fit the initial rate line.

Averaging the initial rates of these reactions gives $k_0 = 2.9*10^{-6}$ M/s. Given $I_0 = 1.8*10^{-5}$ einstein $L^{-1}s^{-1}$, this gives an average value of $\Phi = 0.16$.

Quantum yield of Pd-C homolytic cleavage of 1a in the presence of excess TEMPO at different temperatures: Arrhenius-style plot

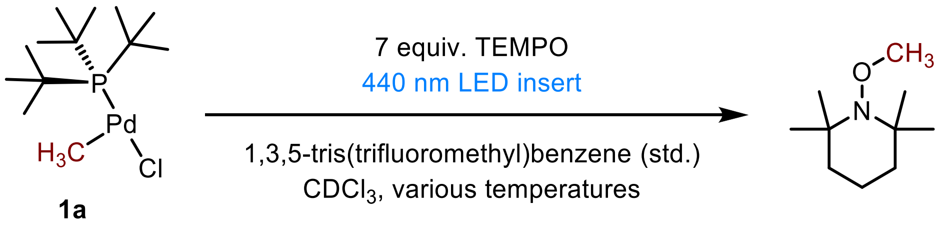

A 60 mM stock solution of **1a** containing 7 equivalents of TEMPO and 1.7 equivalents of 1,3,5-tris(trifluoromethyl)benzene (std.) in $CDCl_3$ was prepared in a nitrogen filled glovebox. 500 μL of this stock solution was transferred to an NMR tube, a coaxial insert was inserted,[11] and

the tube was sealed first with electrical tape and then parafilm. The tube was exported from the glovebox, the fiber optic cable was inserted, and the sample was placed in the spectrometer. The temperature in the probe was then adjusted to the desired temperature. An initial dark ^{1}H spectrum was recorded, then ^{1}H NMR spectra began to be continuously recorded. After several dark spectra were recorded, the light source was turned on (set to 1.0 of a maximum of 10) and spectra were continuously recorded for the duration of the experiment by the steady state technique. The initial rate of TEMPO-Me formation was observed and taken to be k_0 (see Figure S154 to S159). Applying (2), using the value of I_0 determined from the calibration using 2,4-DNBA, then allowed Φ to be determined at each temperature.

Table S11. Initial rates of TEMPO-Me formation from irradiation of **1a** at different temperatures.

entry	temperature (°C)	observed rate, M/s	Φ
1	-15	$5.2*10^{-6}$	0.29
2	-5	$6.4*10^{-6}$	0.35
3	5	$8.0*10^{-6}$	0.44
4	15	$1.0*10^{-5}$	0.56
5	26.9	$1.3*10^{-5}$	0.73
6	35	$1.5*10^{-5}$	0.84

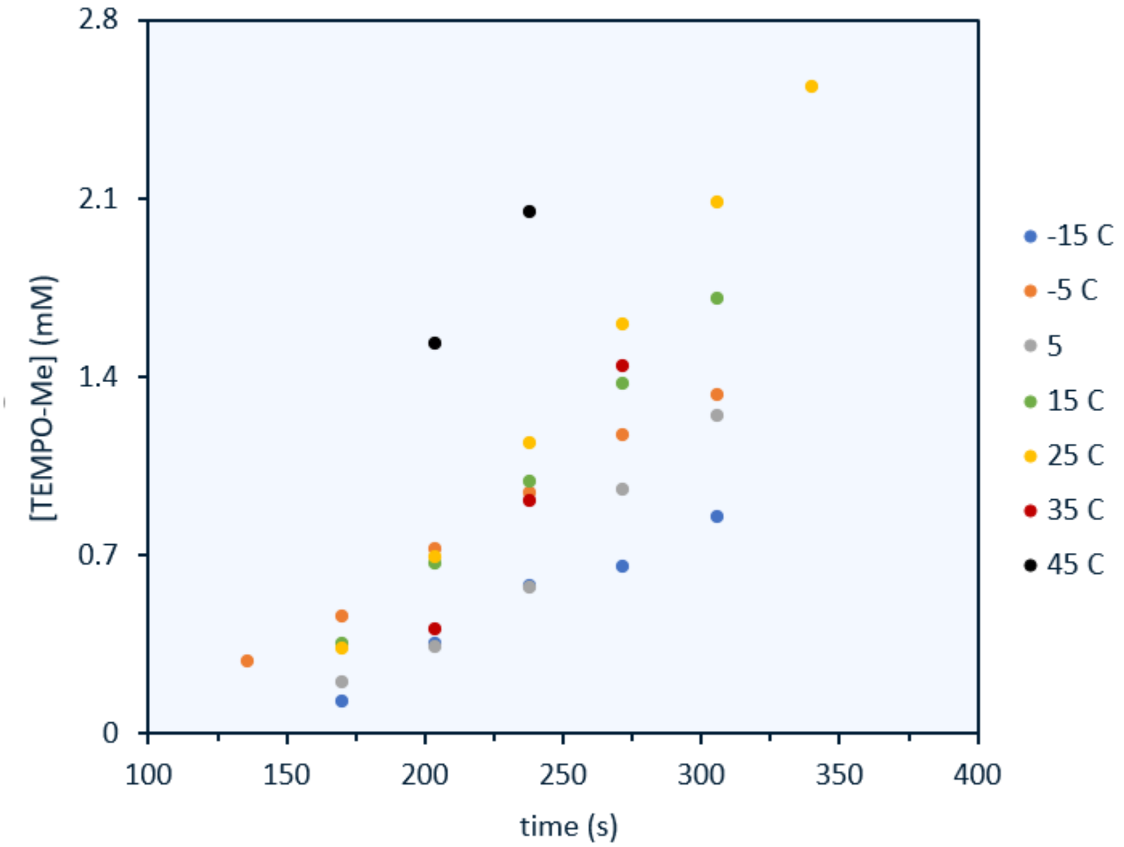

Figure S151. Overlay of initial rates of reactions from Table S11. At higher temperatures, the linear initial rate regime became shorter, so fewer data points were used.

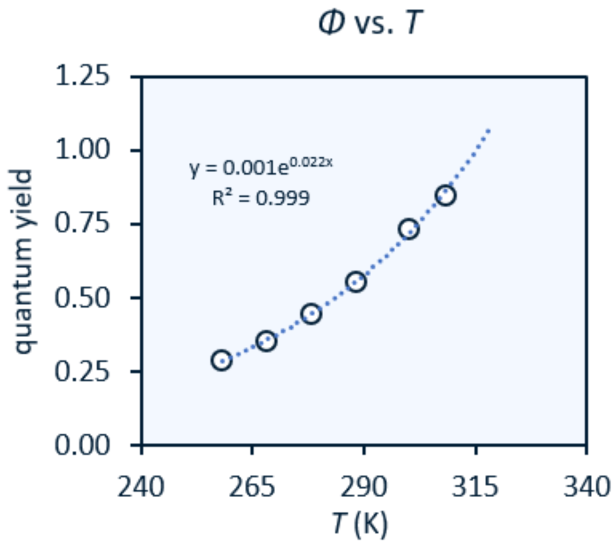

Figure S152. Quantum yield (Φ) vs. temperature for reaction of **1a** in the presence of excess TEMPO.

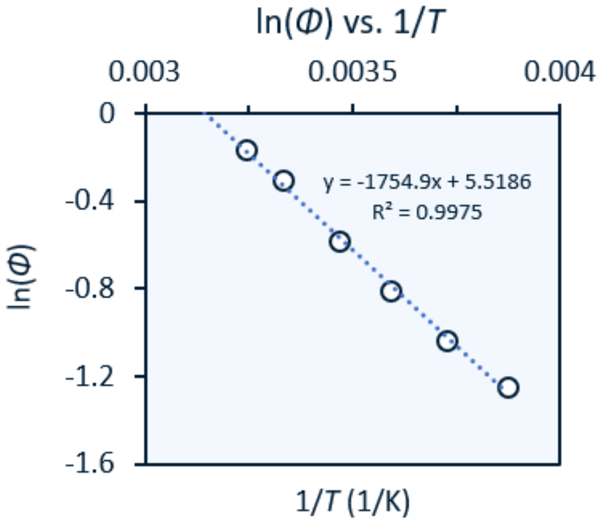

Figure S153. ln(Φ) vs. 1/T for reaction of **1a** in the presence of excess TEMPO.

We apply the equation $\Phi = \Phi_0 e^{-\frac{E_a}{RT}}$ which can be rearranged to $\ln(\Phi) = \ln(\Phi_0) - \frac{E_a}{R}\left(\frac{1}{T}\right)$. From this we set the slope of the line in Figure S153 equal to -E_a/R, obtaining $-1754.9\,K = -\frac{E_a}{R}$. Rearranging we obtain $E_a = R * 1754.9\,K = 1.99 * 10^{-3}\frac{kcal}{K*mol} * 1754.9\,K$ which results in $E_a = {\sim}4\frac{kcal}{mol}$.

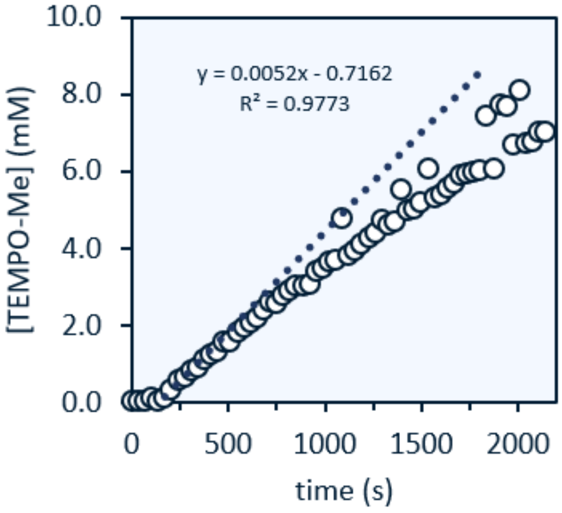

Figure S154. Initial rates of TEMPO-Me formation for irradiation of **1a** in the presence of excess TEMPO. In this case T = -15 °C, see Table S11 entry 1. A series of dark spectra were initially recorded, where the concentration of TEMPO-Me does not change. The initial rate was determined from the first few data points recorded after irradiation began.

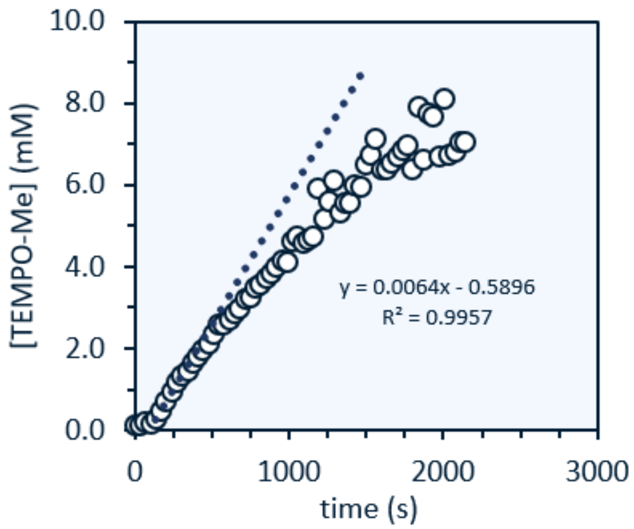

Figure S155. Initial rates of TEMPO-Me formation for irradiation of **1a** in the presence of excess TEMPO. In this case T = -5 °C, see Table S11 entry 2. A series of dark spectra were initially recorded, where the concentration of TEMPO-Me does not change. The initial rate was determined from the first few data points recorded after irradiation began.

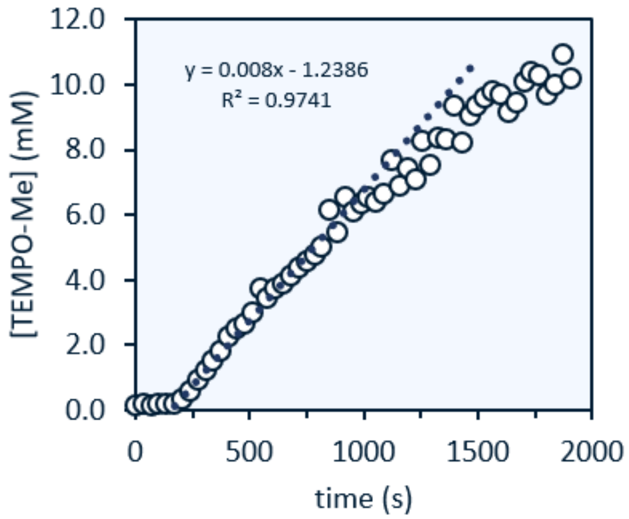

Figure S156. Initial rates of TEMPO-Me formation for irradiation of **1a** in the presence of excess TEMPO. In this case T = 5 °C, see Table S11 entry 3. A series of dark spectra were initially recorded, where the concentration of TEMPO-Me does not change. The initial rate was determined from the first few data points recorded after irradiation began.

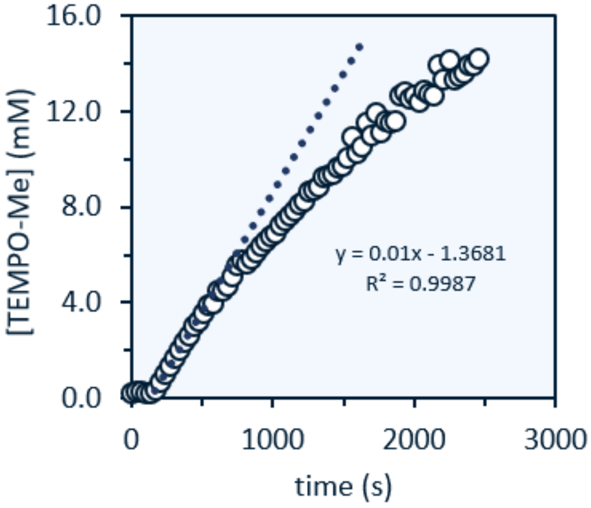

Figure S157. Initial rates of TEMPO-Me formation for irradiation of **1a** in the presence of excess TEMPO. In this case $T = 15$ °C, see Table S11 entry 4. A series of dark spectra were initially recorded, where the concentration of TEMPO-Me does not change. The initial rate was determined from the first few data points recorded after irradiation began.

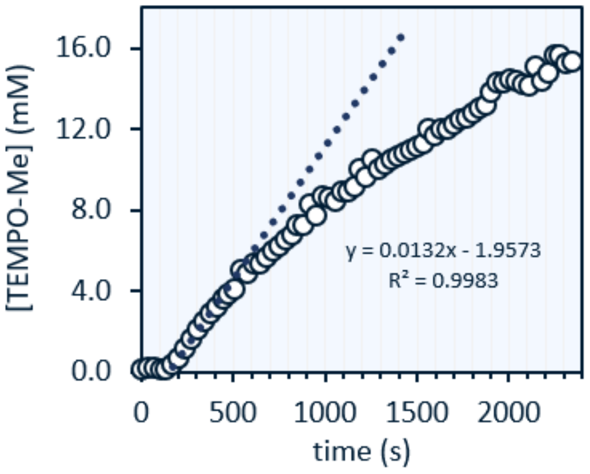

Figure S158. Initial rates of TEMPO-Me formation for irradiation of **1a** in the presence of excess TEMPO. In this case $T = 26.9$ °C, see Table S11 entry 5. A series of dark spectra were initially recorded, where the concentration of TEMPO-Me does not change. The initial rate was determined from the first few data points recorded after irradiation began. These data are the same as the data that appear in Table S3 entry 3, see also Figure S112.

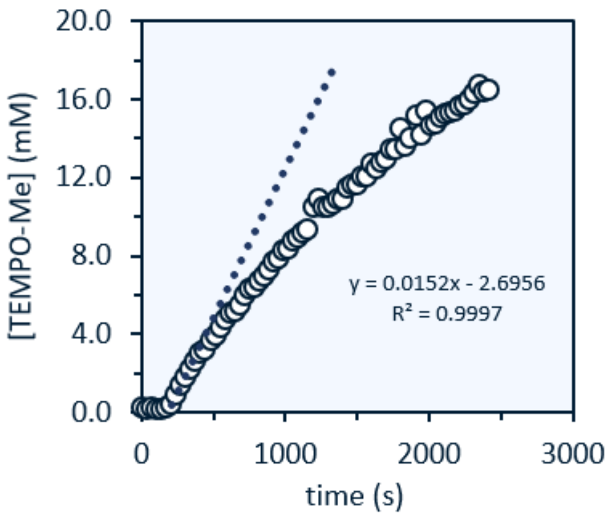

Figure S159. Initial rates of TEMPO-Me formation for irradiation of **1a** in the presence of excess TEMPO. In this case T = 35 °C, see Table S11 entry 6. A series of dark spectra were initially recorded, where the concentration of TEMPO-Me does not change. The initial rate was determined from the first few data points recorded after irradiation began.

Quantum yield of Pd-C homolytic cleavage of 1a in the presence of excess TEMPO at different temperatures: Arrhenius-style plot

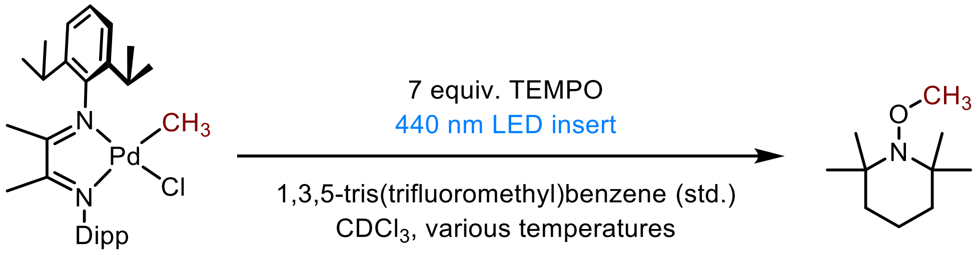

A 30 mM stock solution of **3** containing 7 equivalents of TEMPO and 1.7 equivalents of 1,3,5-tris(trifluoromethyl)benzene (std.) in $CDCl_3$ was prepared in a nitrogen filled glovebox. 500 µL of this stock solution was transferred to an NMR tube, a coaxial insert was inserted,[11] and the tube was sealed first with electrical tape and then parafilm. The tube was exported from the glovebox, the fiber optic cable was inserted, and the sample was placed in the spectrometer. The temperature in the probe was then adjusted to the desired temperature. An initial dark ^1H spectrum was recorded, then ^1H NMR spectra began to be continuously recorded. After several dark spectra were recorded, the light source was turned on (set to 1.0 of a maximum of 10) and

spectra were continuously recorded for the duration of the experiment by the steady state technique. The initial rate of TEMPO-Me formation was observed and taken to be k_0 (see Figure S163 to S168). Applying (2), using the value of I_0 determined from the calibration using 2,4-DNBA, then allowed Φ to be determined at each temperature.

Table S12. Initial rates of TEMPO-Me formation from irradiation of **1a** at different temperatures.

**entry**	**temperature (°C)**	**observed rate, M/s**	Φ
1	-15	$8.0*10^{-7}$	0.04
2	-5	$1.0*10^{-6}$	0.06
3	5	$1.2*10^{-6}$	0.06
4	15	$1.3*10^{-6}$	0.07
5	26.9	$1.8*10^{-6}$	0.10
6	35	$2.0*10^{-6}$	0.11

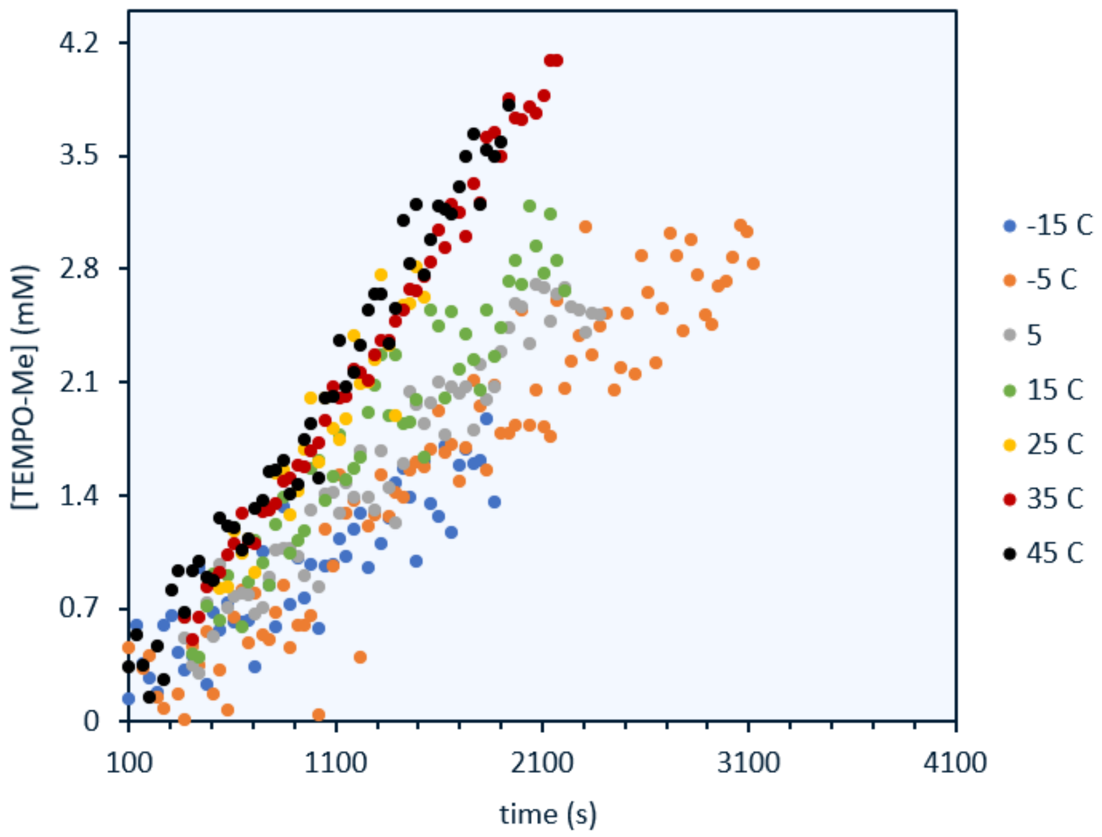

Figure S160. Overlay of initial rates of reactions from Table S12. Due to the high overall linearity of these reactions, along with the relatively high variance in measured product concentration at low temperatures, more data points were used for these reactions in order to determine the initial rates.

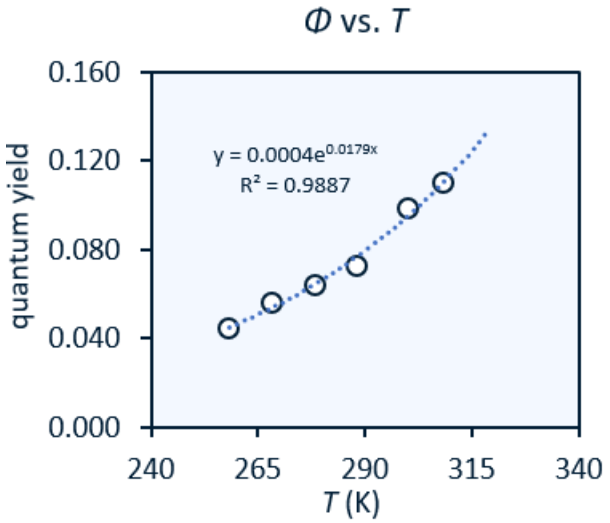

Figure S161. Quantum yield (*Φ*) vs. temperature for reaction of **3** in the presence of excess TEMPO.

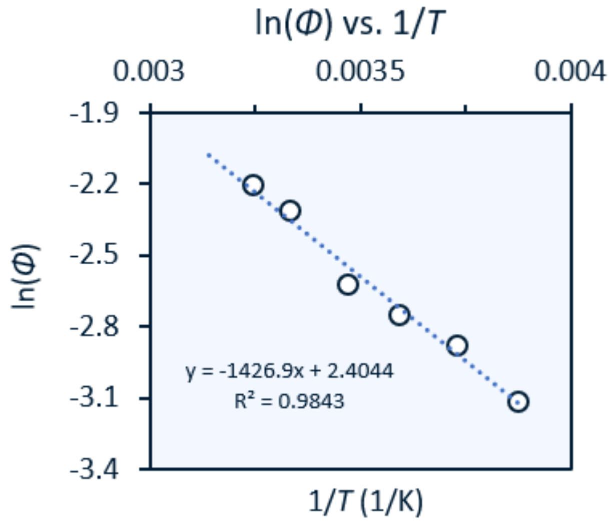

Figure S162. ln(*Φ*) vs. 1/T for reaction of **3** in the presence of excess TEMPO.

We apply the equation $\Phi = \Phi_0 e^{-\frac{E_a}{RT}}$ which can be rearranged to $\ln(\Phi) = \ln(\Phi_0) - \frac{E_a}{R}\left(\frac{1}{T}\right)$. From this we set the slope of the line in Figure S162 equal to -E_a/*R*, obtaining $-1426.9\ K = -\frac{E_a}{R}$. Rearranging we obtain $E_a = R * 1426.9\ K = 1.99 * 10^{-3}\frac{kcal}{K*mol} * 1426.9\ K$ which results in $E_a = {\sim}3\frac{kcal}{mol}$.

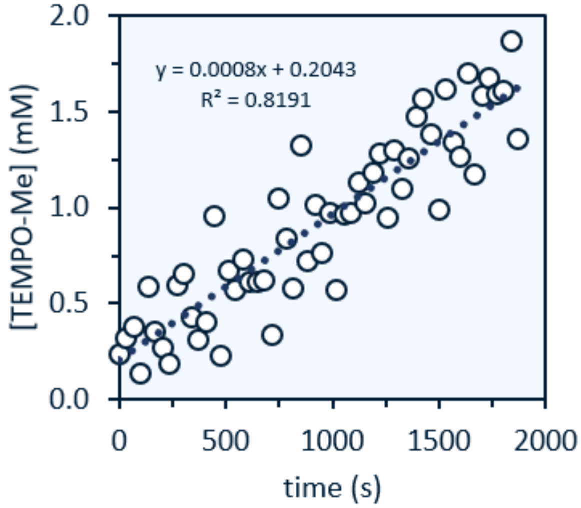

Figure S163. Initial rates of TEMPO-Me formation for irradiation of **3** in the presence of excess TEMPO. In this case *T* = -15 °C, see Table S12 entry 1. The initial rate was determined

from the first few data points recorded after irradiation began.

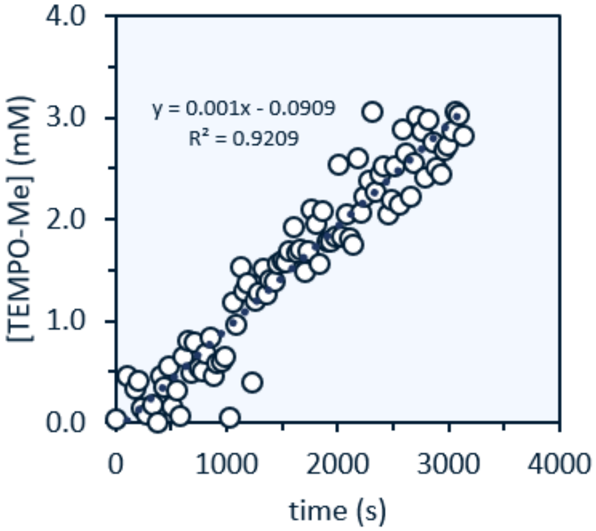

Figure S164. Initial rates of TEMPO-Me formation for irradiation of **3** in the presence of excess TEMPO. In this case T = -5 °C, see Table S12 entry 2. The initial rate was determined from the first few data points recorded after irradiation began.

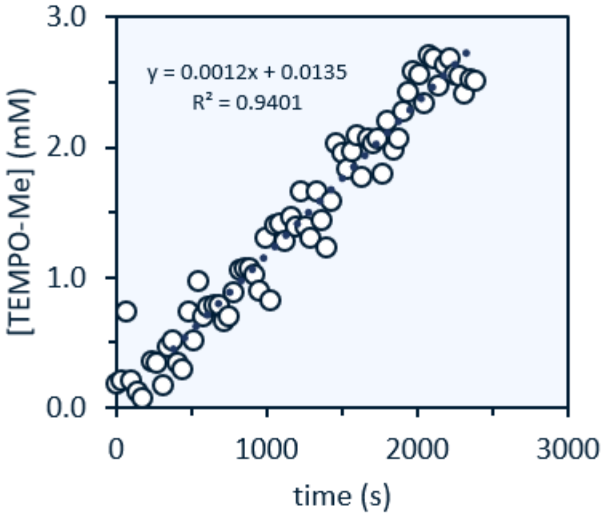

Figure S165. Initial rates of TEMPO-Me formation for irradiation of **3** in the presence of excess TEMPO. In this case T = 5 °C, see Table S12 entry 3. The initial rate was determined from the first few data points recorded after irradiation began.

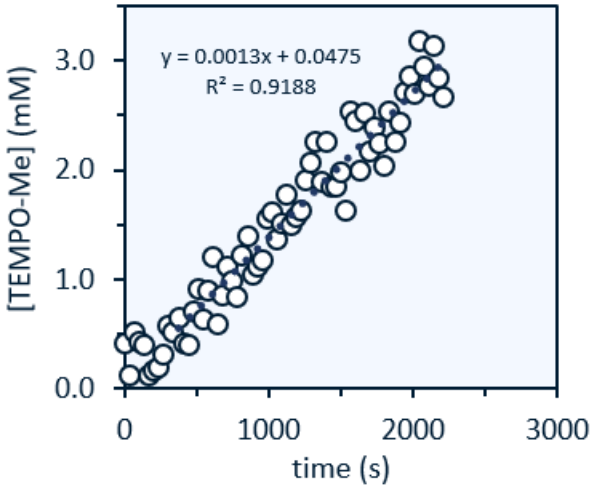

Figure S166. Initial rates of TEMPO-Me formation for irradiation of **3** in the presence of excess TEMPO. In this case $T = 15$ °C, see Table S12 entry 4. The initial rate was determined from the first few data points recorded after irradiation began.

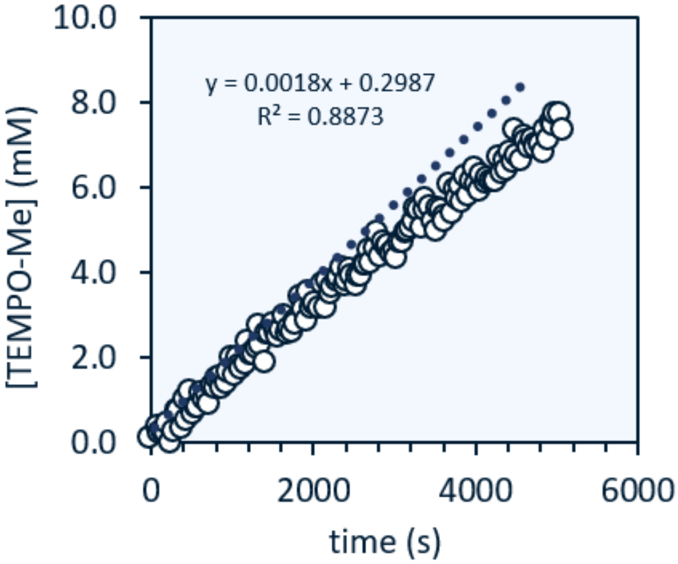

Figure S167. Initial rates of TEMPO-Me formation for irradiation of **3** in the presence of excess TEMPO. In this case $T = 26.9$ °C, see Table S12 entry 5. A series of dark spectra were initially recorded, where the concentration of TEMPO-Me does not change. The initial rate was determined from the first few data points recorded after irradiation began. These data are the same as the data that appear in Table S8 entry 2, see also Figure S140.

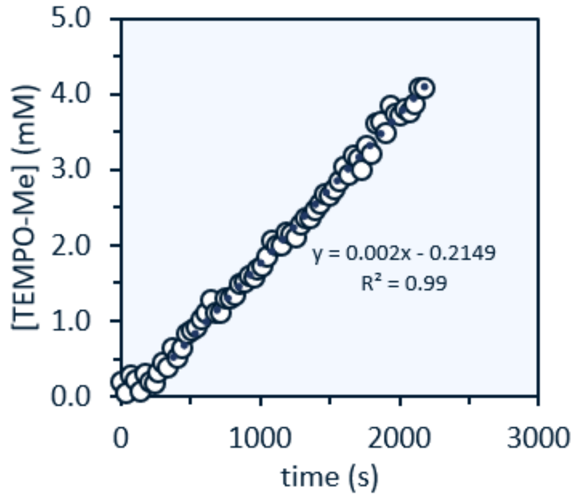

Figure S168. Initial rates of TEMPO-Me formation for irradiation of **3** in the presence of excess TEMPO. In this case T = 35 °C, see Table S12 entry 6. A series of dark spectra were initially recorded, where the concentration of TEMPO-Me does not change. The initial rate was determined from the first few data points recorded after irradiation began.

DFT calculations

Density functional theory (DFT)-based calculations were performed on Gaussian 16 A.03 software. unity.[13] For all calculations, Los Alamos ECP plus double-zeta basis set LANL2DZ[14] for Pd and triple-zeta basis set 6-311++G(d,p)[15] for all other elements were used. Grimme's D3 empirical dispersion correction[16] were employed. Solvation effects were accounted for using Truhlar's SMD variant[17] of the polarizable continuum model (PCM) with solvent-specific parameters for $CHCl_3$. Ground state geometry optimizations were carried out using Becke's three-parameter hybrid exchange-correlation functional B3LYP[18] in conjunction with frequency calculation to confirm that the structure was minimal on the potential energy surface. Linear response time-dependent DFT (TD-DFT) calculations were carried out on the optimized ground state geometry using Minnesota 06 functional[19] for **S3** and range-separated CAM-B3LYP functional for **1a**. Geometry optimizations on the lowest triplet excited state were carried out using Minnesota 06 functional for **S3** and range-separated CAM-B3LYP[20] functional for **1a** in conjunction with frequency calculation to confirm that the structure was minimal on the potential energy surface. The functional choice in TD-DFT calculations was justified by benchmarking studies via comparisons between experimental and calculated

absorption spectra. Population analysis such as Mayer bond order analysis and atomic contributions was performed using Multiwfn software.[21]

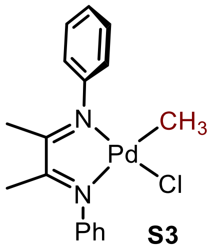

S3

To ease the computations, the structure of **3** was simplified to **S3** by removing the isopropyl groups from the aryl rings on the diimine ligand.

Ground state optimizations

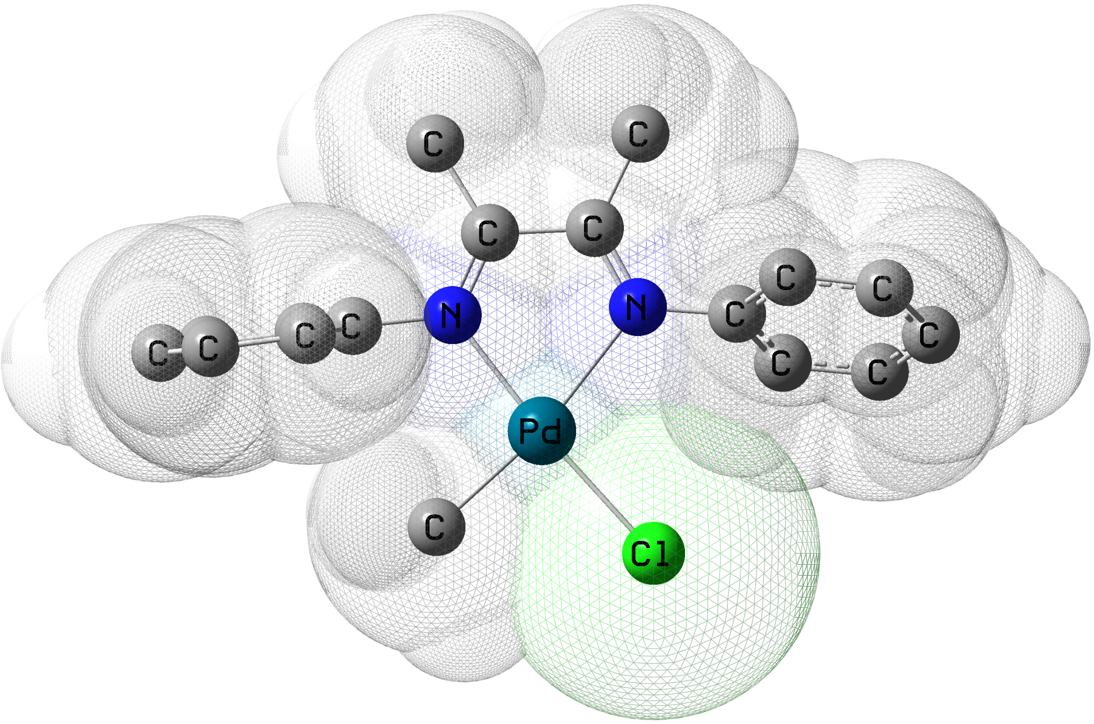

Figure S169. S_0 optimized geometry of **S3**. Surrounding meshes represent the solvation shell that is used in the calculation to account for solvent effects in SMD model.

Table S13. Cartesian coordinates for **S3** S_0 optimized geometry.

Tag	Symbol	X	Y	Z
1	C	-3.43746	0.31969	1.221123
2	C	-2.78261	0.451562	-0.00256
3	C	-3.48928	0.369962	-1.20154
4	C	-4.86981	0.181395	-1.17101
5	C	-5.53798	0.065961	0.04812
6	C	-4.81847	0.134506	1.241476

7	H	-2.86497	0.358849	2.140392
8	H	-2.95722	0.448655	-2.14248
9	H	-5.42115	0.119423	-2.10256
10	H	-6.61118	-0.08565	0.06801
11	H	-5.32997	0.036048	2.192393
12	C	-0.82621	1.781384	-0.09014
13	C	0.672493	1.850738	-0.12569
14	C	-1.60744	3.059318	-0.12932
15	H	-2.66668	2.894168	0.050449
16	H	-1.22446	3.761169	0.615372
17	H	-1.49107	3.533884	-1.10918
18	C	1.323854	3.198834	-0.22903
19	H	0.836036	3.796779	-1.00195
20	H	1.225395	3.745472	0.714201
21	H	2.382041	3.112833	-0.46598
22	N	-1.35911	0.605064	-0.02859
23	N	1.289073	0.721129	-0.07987
24	C	2.704668	0.625634	-0.03849
25	C	3.429961	1.149429	1.036391
26	C	3.360243	-0.07523	-1.05395
27	C	4.81333	0.991437	1.078146
28	H	2.90732	1.6593	1.837384
29	C	4.745354	-0.20529	-1.01685
30	H	2.779859	-0.51341	-1.85569
31	C	5.476096	0.32385	0.048105
32	H	5.371826	1.38992	1.917865
33	H	5.252242	-0.73966	-1.81246
34	H	6.553061	0.203967	0.081802
35	Pd	-0.08287	-1.04914	0.025195
36	C	-1.58854	-2.42392	0.12273

37	H	-2.25648	-2.23138	-0.72146
38	H	-1.19775	-3.43994	0.082113
39	H	-2.11464	-2.25211	1.066324
40	Cl	1.513007	-2.80098	0.095334

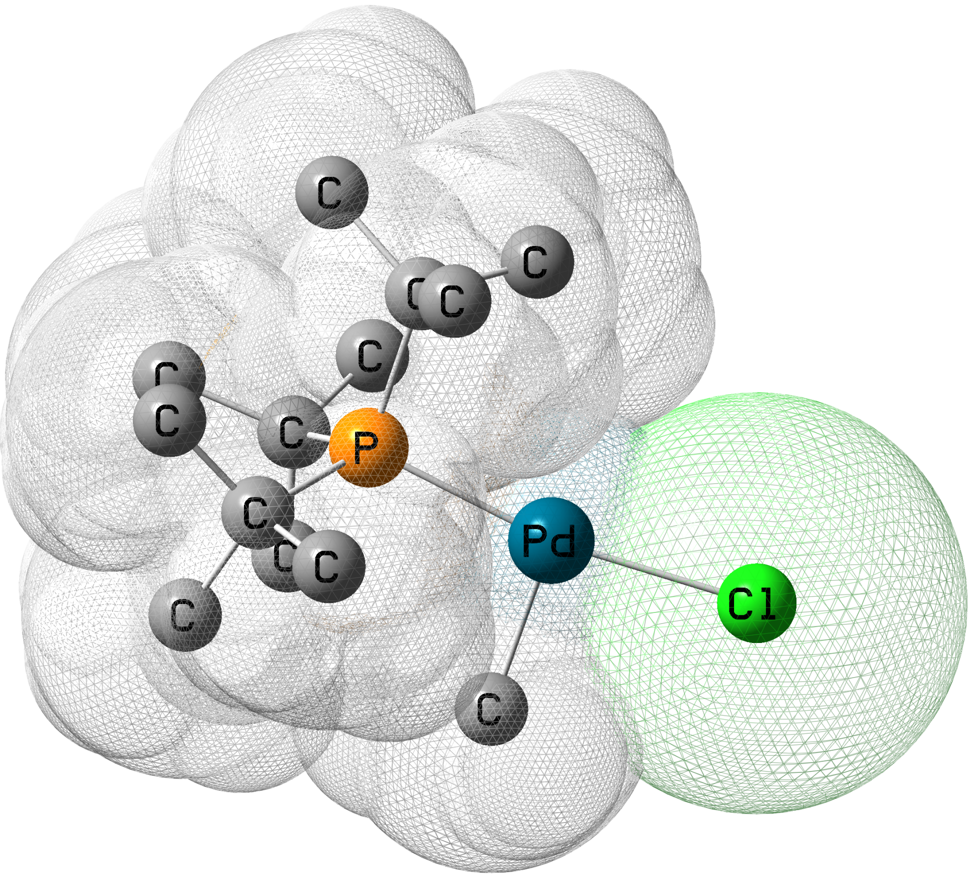

Figure S170. S_0 optimized geometry of **1a**. Surrounding meshes represent the solvation shell that is used in the calculation to account for solvent effects in SMD model.

Table S14. Cartesian coordinates for **1a** S_0 optimized geometry.

**Tag**	**Symbol**	**X**	**Y**	**Z**

1	C	-1.78897	-2.08918	-0.07775
2	H	-0.85881	-2.62988	0.05315
3	H	-2.49021	-2.31218	0.727399
4	H	-2.2379	-2.28995	-1.0519
5	C	1.659559	-1.02713	-1.34218
6	C	1.868502	-2.48856	-0.89167
7	H	0.935423	-2.99094	-0.64084
8	H	2.310672	-3.03355	-1.73256
9	H	2.555665	-2.58428	-0.05309
10	C	0.772497	-1.08008	-2.60557
11	H	0.599736	-0.1055	-3.05579
12	H	1.277582	-1.70077	-3.3539
13	H	-0.19786	-1.5324	-2.39474
14	C	3.043267	-0.45202	-1.70499
15	H	3.710147	-0.39434	-0.84436
16	H	3.510422	-1.11957	-2.43748
17	H	2.986248	0.534121	-2.16192
18	C	0.978538	1.898523	-0.36768
19	C	0.721773	2.16931	-1.86478
20	H	-0.24229	1.773242	-2.19212
21	H	0.69712	3.25431	-2.01199
22	H	1.501256	1.775034	-2.51389
23	C	2.356595	2.46163	0.019384
24	H	3.178908	1.947559	-0.47655
25	H	2.39619	3.51413	-0.28412
26	H	2.529663	2.430606	1.095091
27	C	-0.11558	2.686381	0.39304
28	H	-0.07643	2.572309	1.472182
29	H	0.011329	3.751189	0.168559
30	H	-1.12645	2.425187	0.055169

31	C	1.436984	-0.41109	1.751243
32	C	0.858863	-1.77171	2.199856
33	H	1.241498	-1.98861	3.203192
34	H	-0.23023	-1.73988	2.259005
35	H	1.145029	-2.60211	1.558977
36	C	0.924323	0.625912	2.771073
37	H	1.37357	1.609636	2.641146
38	H	-0.16353	0.723742	2.73985
39	H	1.193809	0.277518	3.773778
40	C	2.974728	-0.45869	1.821795
41	H	3.440383	0.47669	1.513251
42	H	3.267253	-0.64327	2.861636
43	H	3.397389	-1.26455	1.22256
44	P	0.734596	0.008788	0.005357
45	Cl	-3.99261	0.270644	-0.02227
46	Pd	-1.58536	-0.07056	-0.00983

TD-DFT and UV-vis spectrum simulation

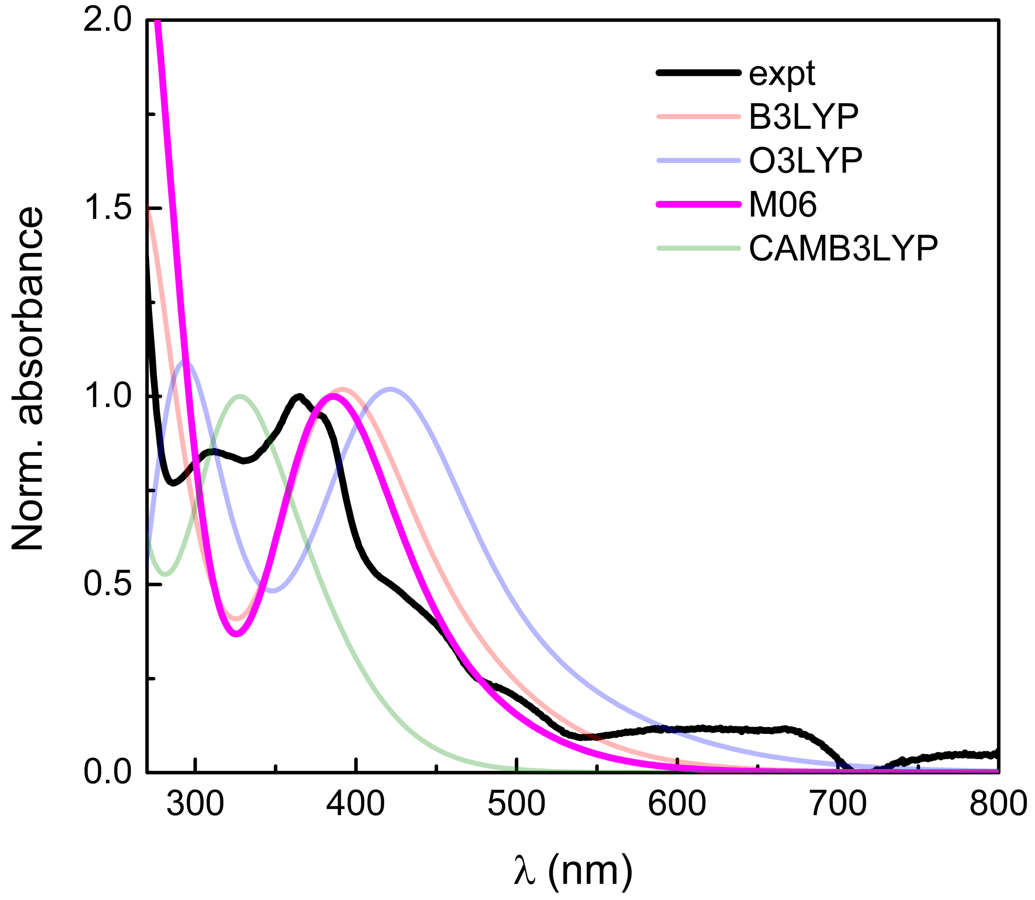

Figure S171. Comparisons between calculated absorption spectra and experimental spectrum of **S3** in $CHCl_3$. Functionals are varied based on different degrees of Hartree-Fock exchange component: M06 (27% HF exchange) > B3LYP (20% HF exchange) > O3LYP[22] (12% HF exchange), HF component in CAM-B3LYP is variable, being 19% in the short-range limit and 65% in the long range limit.

Table S15. TD-DFT calculated Franck-Condon transitions 1-40 for **S3**.

Excited state #	spin state	energy	wavelength	oscillation strength
1	triplet	2.3441 eV	528.93 nm	0

2	triplet	2.4497 eV	506.11 nm	0
3	triplet	2.5383 eV	488.45 nm	0
4	singlet	2.5457 eV	487.03 nm	0.0021
5	triplet	2.5842 eV	479.78 nm	0
6	singlet	2.6983 eV	459.49 nm	0.0132
7	singlet	2.7076 eV	457.90 nm	0.0246
8	triplet	2.7460 eV	451.51 nm	0
9	triplet	2.7532 eV	450.33 nm	0
10	triplet	2.8969 eV	427.99 nm	0
11	triplet	2.9790 eV	416.20 nm	0
12	triplet	3.0175 eV	410.88 nm	0
13	singlet	3.1339 eV	395.62 nm	0.0073
14	singlet	3.1565 eV	392.79 nm	0.0107
15	singlet	3.1775 eV	390.19 nm	0.0726
16	singlet	3.1832 eV	389.49 nm	0.0016
17	singlet	3.2435 eV	382.25 nm	0.1185
18	triplet	3.4466 eV	359.73 nm	0
19	singlet	3.4469 eV	359.70 nm	0.0017
20	triplet	3.5208 eV	352.15 nm	0
21	singlet	3.6136 eV	343.11 nm	0.0251
22	triplet	3.6213 eV	342.37 nm	0
23	triplet	3.7146 eV	333.78 nm	0
24	triplet	3.7374 eV	331.74 nm	0
25	triplet	3.7767 eV	328.28 nm	0
26	singlet	3.8504 eV	322.01 nm	0.0066
27	triplet	4.0401 eV	306.88 nm	0
28	triplet	4.0740 eV	304.33 nm	0
29	singlet	4.0889 eV	303.22 nm	0.0051
30	singlet	4.1061 eV	301.95 nm	0.0115
31	triplet	4.2886 eV	289.10 nm	0

32	triplet	4.3132 eV	287.45 nm	0
33	singlet	4.3439 eV	285.42 nm	0.0494
34	triplet	4.3604 eV	284.34 nm	0
35	singlet	4.4259 eV	280.13 nm	0.108
36	singlet	4.4678 eV	277.51 nm	0.0584
37	singlet	4.6100 eV	268.95 nm	0.0631
38	singlet	4.6411 eV	267.14 nm	0.0368
39	singlet	4.6765 eV	265.12 nm	0.0026
40	singlet	4.7559 eV	260.69 nm	0.009

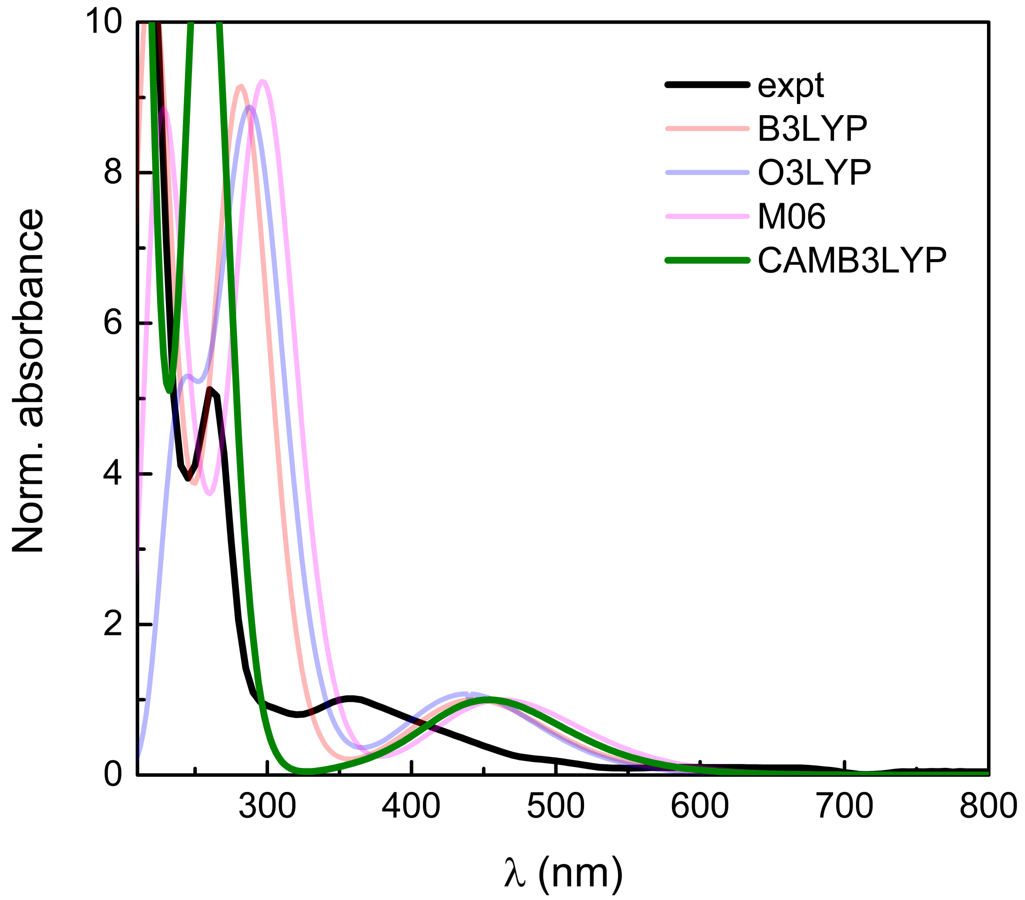

Figure S172. Comparisons between calculated absorption spectra and experimental spectrum of **1a** in $CHCl_3$. Functionals are varied based on different degrees of Hartree-Fock exchange component: M06 (27% HF exchange) > B3LYP (20% HF exchange) > O3LYP (12% HF exchange), HF component in CAM-B3LYP is variable, being 19% in the short-range limit and 65% in the long-range limit.

Table S16. TD-DFT calculated Franck-Condon transitions 1-40 for **3**.

excited state #	spin state	energy	wavelength	oscillation strength
1	triplet	2.0382 eV	608.30 nm	0
2	triplet	2.5536 eV	485.54 nm	0
3	triplet	2.6776 eV	463.04 nm	0
4	singlet	2.7213 eV	455.61 nm	0.0327
5	triplet	2.9032 eV	427.06 nm	0
6	singlet	3.1245 eV	396.81 nm	0.0003
7	singlet	3.2807 eV	377.92 nm	0.0033
8	singlet	3.3156 eV	373.94 nm	0.0013
9	triplet	3.7771 eV	328.25 nm	0
10	triplet	4.2892 eV	289.06 nm	0
11	singlet	4.8220 eV	257.12 nm	0.3997
12	triplet	4.9811 eV	248.91 eV	0
13	triplet	5.0503 eV	245.50 nm	0
14	singlet	5.2035 eV	238.27 nm	0.0018
15	singlet	5.2236 eV	237.35 nm	0.0195
16	triplet	5.2970 eV	234.06 nm	0
17	triplet	5.3975 eV	229.70 nm	0
18	singlet	5.6998 eV	217.52 nm	0.0435
19	triplet	5.7469 eV	215.74 nm	0
20	singlet	5.8274 eV	212.76 nm	0.2102
21	singlet	5.8900 eV	210.50 nm	0.0024
22	triplet	5.9665 eV	207.80 nm	0

23	triplet	6.0018 eV	206.58 nm	0
24	triplet	6.0941 eV	203.45 nm	0
25	singlet	6.1176 eV	202.67 nm	0.2553
26	singlet	6.1729 eV	200.85 nm	0.2424
27	triplet	6.2249 eV	199.18 nm	0
28	triplet	6.2613 eV	198.02 nm	0
29	triplet	6.2910 eV	197.08 nm	0
30	singlet	6.3121 eV	196.42 nm	0.0883
31	triplet	6.3293 eV	195.89 nm	0
32	triplet	6.3835 eV	194.23 nm	0
33	triplet	6.3981 eV	193.78 nm	0
34	singlet	6.4268 eV	192.92 nm	0.0329
35	singlet	6.4413 eV	192.48 nm	0.025
36	singlet	6.4700 eV	191.63 nm	0.0026
37	singlet	6.4943 eV	190.91 nm	0.0079
38	singlet	6.5213 eV	190.12 nm	0.0091
39	singlet	6.5593 eV	189.02 nm	0.289
40	singlet	6.6893 eV	185.35 nm	0.4093

Triplet/singlet excited state optimizations

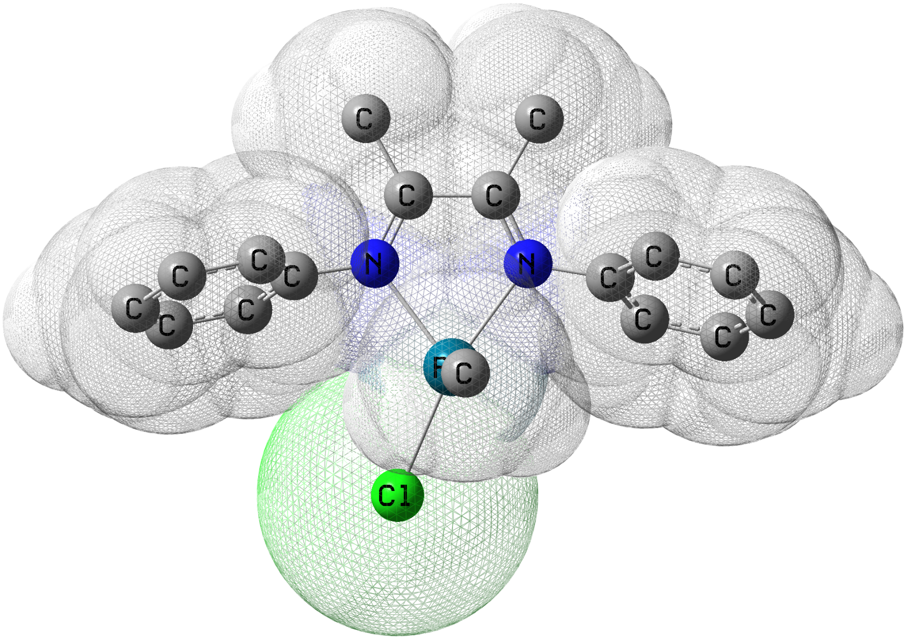

Figure S173. T_1 optimized geometry of **S3**. Surrounding meshes represent the solvation shell that is used in the calculation to account for solvent effects in SMD model.

Table S17. Cartesian coordinates for **3** T_1 optimized geometry.

Tag	Symbol	X	Y	Z
1	C	3.482787	0.931852	0.898997
2	C	2.770963	0.397659	-0.17384
3	C	3.389731	-0.49434	-1.04781
4	C	4.728087	-0.81024	-0.8758

5	C	5.444668	-0.26784	0.184494
6	C	4.816589	0.594108	1.074662
7	H	2.981108	1.590618	1.602735
8	H	2.808305	-0.92841	-1.85683
9	H	5.210653	-1.49502	-1.56641
10	H	6.489322	-0.52786	0.324763
11	H	5.366751	1.00419	1.916171
12	C	0.875693	1.827107	-0.43449
13	C	-0.60577	1.875973	-0.44694
14	C	1.64546	3.100315	-0.5034
15	H	2.704961	2.920505	-0.68838
16	H	1.255002	3.739129	-1.30064
17	H	1.551382	3.665815	0.431805
18	C	-1.29051	3.191987	-0.57906
19	H	-1.06608	3.838063	0.277763
20	H	-0.9338	3.712934	-1.47382
21	H	-2.37196	3.078391	-0.65331
22	N	1.393555	0.648392	-0.34306
23	N	-1.20828	0.743106	-0.32077
24	C	-2.60131	0.609406	-0.14291
25	C	-3.33768	-0.1133	-1.07688
26	C	-3.21629	1.112708	1.001337
27	C	-4.69762	-0.29695	-0.88197
28	H	-2.83153	-0.53012	-1.94304
29	C	-4.57463	0.909973	1.194981
30	H	-2.61887	1.64268	1.739185
31	C	-5.31902	0.212356	0.252107
32	H	-5.27301	-0.85497	-1.61424
33	H	-5.0522	1.294821	2.091044
34	H	-6.38166	0.053517	0.407674

35	Pd	0.044613	-1.02294	-0.00056
36	C	0.144704	-0.74266	2.158063
37	H	0.215814	0.32841	2.369023
38	H	-0.77212	-1.18161	2.557679
39	H	1.036946	-1.28516	2.482015
40	Cl	-0.91784	-3.23412	-0.23765

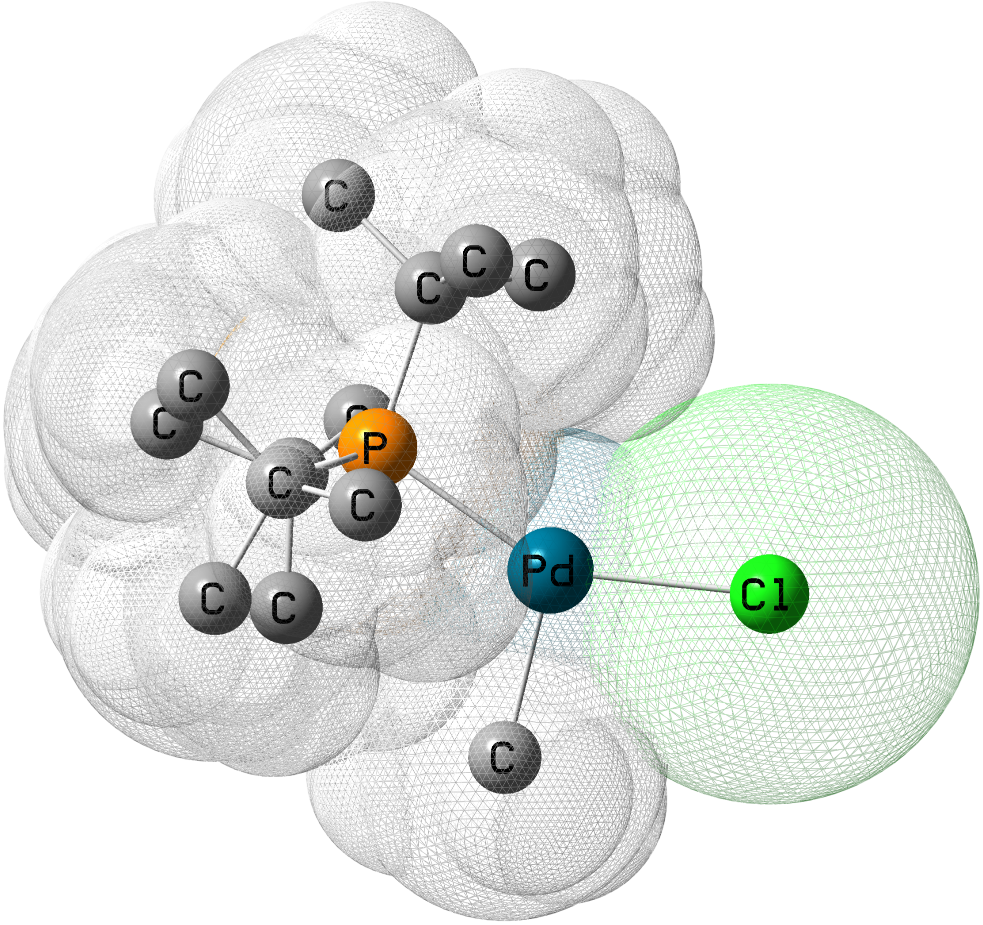

Figure S174. T_1 optimized geometry of **1a**. Surrounding meshes represent the solvation shell that is used in the calculation to account for solvent effects in SMD model.

Table S18. Cartesian coordinates for **3** T_1 optimized geometry.

Tag	Symbol	X	Y	Z
1	C	2.215189	-2.26963	-0.07399
2	H	1.367997	-2.93862	-0.21059
3	H	2.934305	-2.37142	-0.888
4	H	2.705733	-2.43786	0.886329
5	C	-1.39549	-0.80178	1.588959
6	C	-1.41768	-2.32549	1.400111
7	H	-0.45745	-2.70876	1.055357
8	H	-1.62137	-2.78033	2.373701
9	H	-2.1991	-2.65925	0.720528
10	C	-0.35685	-0.53021	2.693448
11	H	-0.2757	0.516091	2.972536
12	H	-0.6529	-1.0855	3.588348
13	H	0.636034	-0.89001	2.406461
14	C	-2.78226	-0.35352	2.063769
15	H	-3.55359	-0.5271	1.313878
16	H	-3.04885	-0.93614	2.95112
17	H	-2.81096	0.696821	2.348199
18	C	-1.16396	1.91912	0.063827
19	C	-0.75037	2.468769	1.437482
20	H	0.286225	2.225512	1.680139
21	H	-0.82597	3.559005	1.401433
22	H	-1.39215	2.128599	2.24699
23	C	-2.62623	2.294278	-0.20009
24	H	-3.31507	1.815797	0.494742
25	H	-2.73416	3.37658	-0.07732
26	H	-2.94063	2.053537	-1.21449
27	C	-0.2621	2.639497	-0.95599
28	H	-0.48661	2.397073	-1.99024

29	H	-0.40579	3.71739	-0.83662
30	H	0.795472	2.430262	-0.77482
31	C	-1.61829	-0.72627	-1.5358
32	C	-0.94674	-2.07568	-1.84522
33	H	-1.41135	-2.48544	-2.74698
34	H	0.119026	-1.95542	-2.04586
35	H	-1.06033	-2.8133	-1.05657
36	C	-1.35662	0.157856	-2.76413
37	H	-1.91119	1.093521	-2.74286
38	H	-0.29471	0.378299	-2.89275
39	H	-1.6843	-0.39199	-3.65088
40	C	-3.13061	-0.93263	-1.38893
41	H	-3.66073	-0.00881	-1.16204
42	H	-3.5217	-1.31205	-2.33827
43	H	-3.37746	-1.66584	-0.62269
44	P	-0.78829	0.046043	-0.00579
45	Cl	3.902538	0.83573	-0.0322
46	Pd	1.60501	-0.17619	-0.07511

Table S19. Mayer bond order analysis of ground state and triplet excited state geometries for **S3** and **1a**.

**Complex**	**Pd-C bond order in GS**	**Pd-C bond order in T1**
**S3**	0.946	0.833
**1a**	1.139	0.648

Table S20. Population analysis and Mayer bond order for linear TD-DFT calculation of **S3**.

**Excited state #**	**Molecular moiety**	**Δpopulation[3]**	**Pd-C bond order (vs. 0.946, GS bond order)**
4 (487.03 nm)	[1]Diimine ligand	0.5275	0.927

	Pd	-0.21187	
	[2]CH_3	-0.07262	
	Cl	-0.24237	
6 (459.49 nm)	Diimine ligand	0.51747	0.965
	Pd	-0.34573	
	CH_3	-0.04528	
	Cl	-0.12643	
7 (457.90 nm)	Diimine ligand	0.48562	0.991
	Pd	-0.23503	
	CH_3	-0.02389	
	Cl	-0.22643	
13 (395.62 nm)	Diimine ligand	0.07406	0.833
	Pd	-0.15377	
	CH_3	0.24445	
	Cl	-0.16474	
14 (392.79 nm)	Diimine ligand	0.06625	0.856
	Pd	-0.14163	
	CH_3	0.23677	
	Cl	-0.16115	
15 (390.19 nm)	Diimine ligand	0.11047	0.925
	Pd	-0.01384	
	CH_3	-0.04459	
	Cl	-0.05202	
16 (389.49 nm)	Diimine ligand	0.04203	0.802
	Pd	-0.34379	
	CH_3	0.23632	
	Cl	0.06549	
17 (382.25 nm)	Diimine ligand	0.41326	0.999
	Pd	-0.26274	
	CH_3	-0.03061	
	Cl	-0.11988	

[1] Diimine ligand moiety includes atoms #1-34.
[2] CH_3 moiety includes atoms #36-39.
[3] Negative number suggests electron loss, while positive number suggests electron gain.

From the table we can assign excited state #4, 6, 7, and 17 to dominantly metal-to-ligand (diimine) charge transfer (MLCT) transitions. Excited state #13, 14, and 16 have some metal-to-ligand (CH_3) charge transfer characters. Excited state #15 is a local excited (LE) state, mostly likely to be d-d transitions centered on the Pd. These characters can also be visualized in the figure below (**Figure S175**).

Table S21. Atomic contributions to HOMO and LUMO for selected Franck-Condon transitions for **S3**.

**Excited state #**	**MO**	**Atomic contributions**
6 (459.49 nm)	HOMO	Pd (d, 52.31%), Cl (p, 23.86%)
	LUMO	C12 (p, 21.07%), N23 (p, 20.38%), N22 (p, 20.17%), C13 (p, 19.83%)
7 (457.90 nm)	HOMO	Pd (d, 68.86%), Cl (p, 22.67%)
	LUMO	C12 (p, 20.91%), N23 (p, 20.24%), N22 (p, 20.01%), C13 (p, 19.69%)

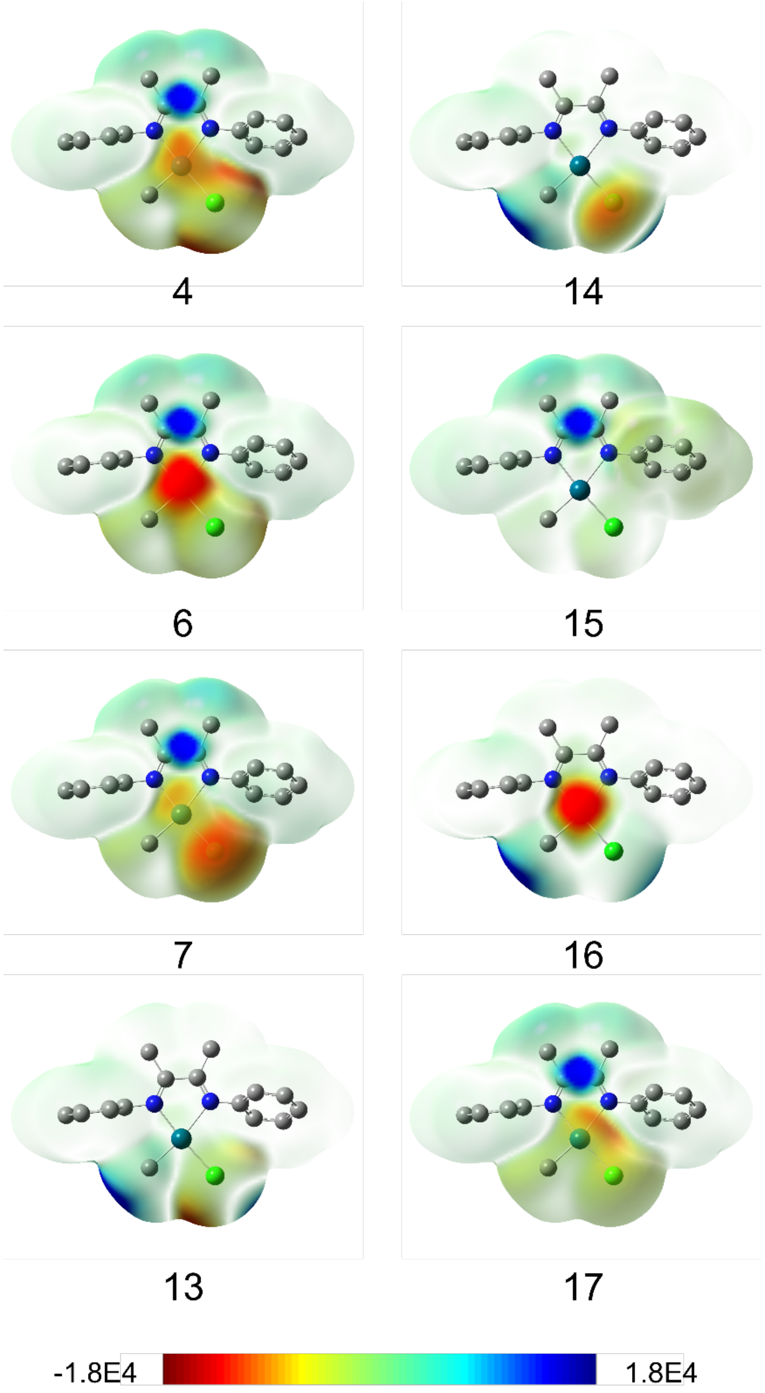

Figure S175. Difference density isosurfaces for selected calculated transitions for **S3**. Blue represents electron gain and red represents electron loss.

Table S22. Population analysis for linear TD-DFT calculation of **1a**.

Excited state #	Molecular moiety	Δcharges[3]	Pd-C bond order (vs. 1.139, GS bond order)
4 (455.61 nm)	[1]Phosphine ligand	0.07013	0.709
	Pd	-0.31646	
	[2]CH_3	0.25178	
	Cl	-0.00547	
6 (396.81 nm)	Phosphine ligand	0.09117	0.769
	Pd	-0.17933	
	CH_3	0.27433	
	Cl	-0.18585	
7 (377.92 nm)	Phosphine ligand	0.08797	0.791
	Pd	-0.3308	
	CH_3	0.2265	
	Cl	0.01635	
8 (373.94 nm)	Phosphine ligand	0.0753	0.782
	Pd	-0.1658	
	CH_3	0.25937	
	Cl	-0.1688	

[1] Phosphine ligand moiety includes atoms #3, 7-45.
[2] CH_3 moiety includes atoms #2, 4-6.
[3] Negative number suggests electron loss, while positive number suggests electron gain.

From the table we can assign all the visible transitions to metal-to-ligand (CH_3) charge transfer (MLCT) state. These characters can also be visualized in the figure below (**Figure S176**).

Table S23. Atomic contributions to HOMO and LUMO for selected Franck-Condon transitions for **1a**.

Excited state #	MO	Atomic contributions
4 (455.61 nm)	HOMO	Pd (d, 78.35%), Pd (s, 13.12%), P (s, 14.20%)
	LUMO	Pd (d, 34.01%), C2 (p, 89.71%), Pd (p, 14.38%)

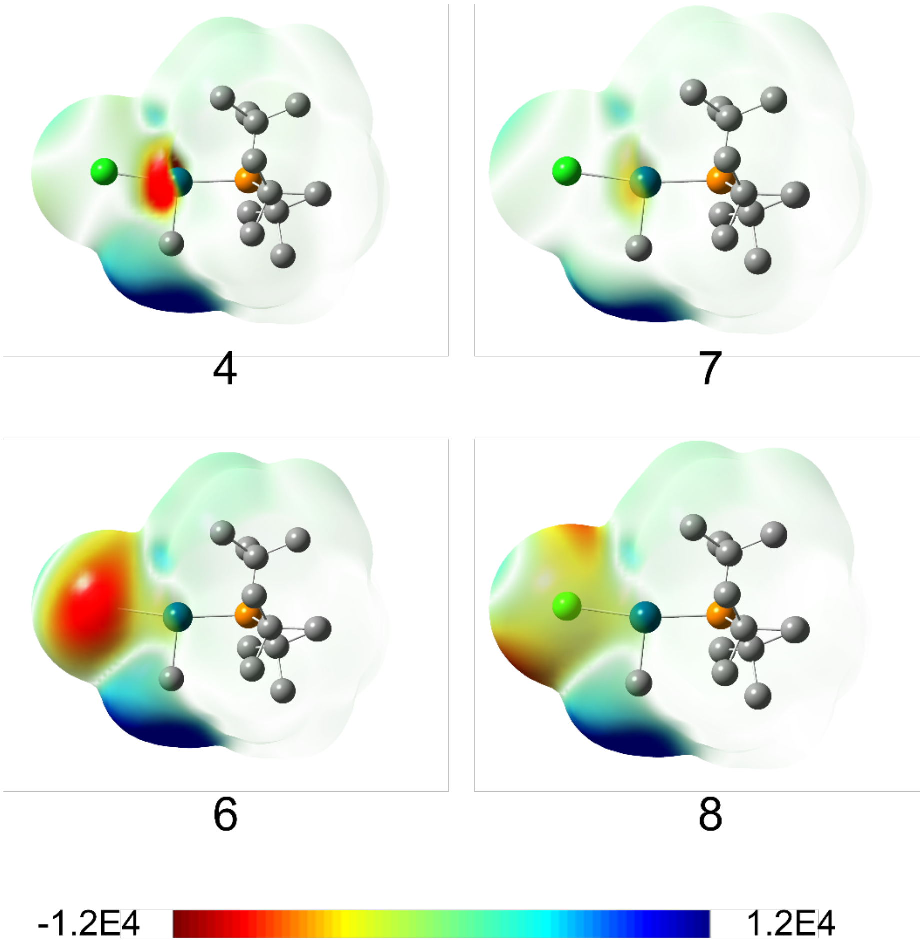

Figure S176. Difference density isosurfaces for selected calculated transitions for **1a**. Blue represents electron gain and red represents electron loss.

Calculation of Pd-C BDFE values

A model isodesmic exchange reaction was chosen to calculate the BDFEs,

diimine-Pd^{II}-CH_3 or tBu_3P-Pd^{II}-CH_3 + $[Pd^{I}]^{+}$ → diimine-Pd^{I} or tBu_3P-Pd^{I} radical + $[Pd^{II}\text{-}CH_3]^{+}$, dG

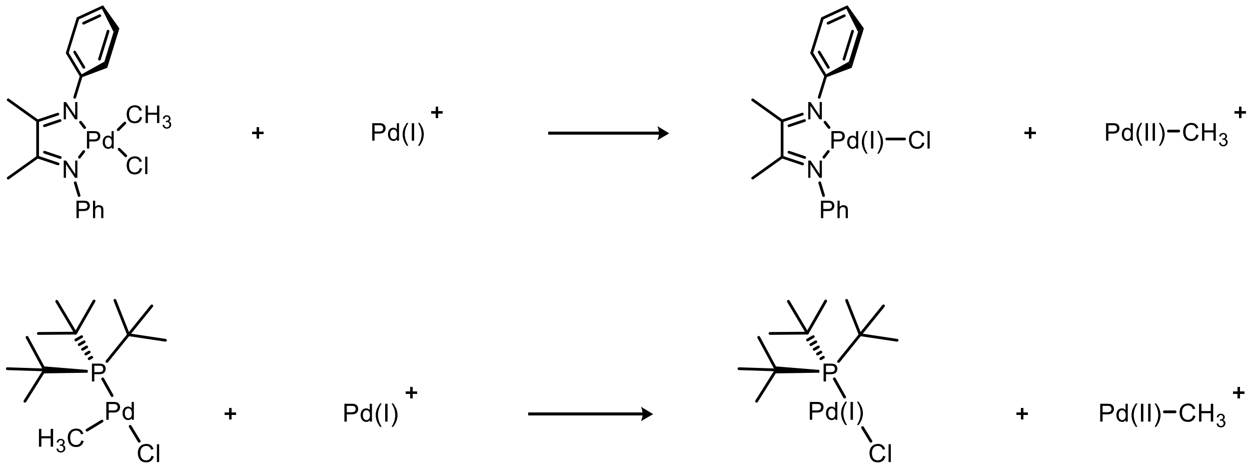

Table S24. Calculation of BDFE of **S3** in S_0 state

G (**S3**(S_0), B3LYP), hartree	G(Pd atom, B3LYP), hartree	G (**S3** radical, B3LYP), hartree	G (Pd-Me, B3LYP), hartree	ΔG S_0, hartree	ΔG S_0, kcal mol^{-1}
-1355.785479	-126.546651	-1315.893961	-166.437837	0.000332	0.21

Table S25. Calculation **S3** triplet energy

G (**S3**(T_1), M06), hartree	G (**S3**(S_0), M06), hartree	triplet energy, hartree	triplet energy, kcal mol^{-1}
-1355.062379	-1355.107308	0.044929	28.3

Therefore, the result of the isodesmic reaction from Table S24 is more exoergic by 28.3 kcal/mol, resulting in ΔG **S3**(T_1) of -28.1 kcal/mol.

Table S26. Calculation of BDFE of **1a** in S_0 state

G (**1a**(S_0), B3LYP), hartree	G (Pd atom, B3LYP), hartree	G (**1a** radical, B3LYP), hartree	G (Pd-Me, B3LYP), hartree	ΔG S_0, hartree	ΔG S_0, kcal mol^{-1}
-1441.7379	-126.546651	-1401.851815	-166.437837	-0.005101	-3.2

Table S27. Calculation of **1a** triplet energy

G (**1a**(T_1), CAM-B3LYP), hartree	G (**1a**(S_0), CAM-B3LYP), hartree	triplet energy, hartree	triplet energy, kcal mol^{-1}
-1441.22943	-1441.286264	0.056834	35.8

Therefore, the result of the isodesmic reaction from Table S24 is more exoergic by 35.8 kcal/mol, resulting in ΔG for **1a**(T_1) of -28.1 kcal/mol.

Table S28. BDFE values for **1a** and **S3** in S_0 and T_1 states, determined relative to Pd(II)-Me^+ [1]

Pd-Me BDFE of **S3**(S_0) (kcal/mol)	Pd-Me BDFE of **S3**(T_1) (kcal/mol)	Pd-Me BDFE of **1a**(S_0) (kcal/mol)	Pd-Me BDFE of **1a**(T_1) (kcal/mol)
59	31	56	20

[1]Relative BDFE values calculated with respect to the model Pd(II)-Me^+ species, which has a reported BDFE of 59 kcal/mol in the gas phase (see Beauchamp et al. *J. Am. Chem. Soc.* **1984,** *106* (16), 4403.)

Coupled Cluster Calculations

Geometry optimizations were performed using DFT on Gaussian 16 A.03 software. unity.[23] For all calculations, Los Alamos ECP plus double-zeta basis set LANL2DZ[24] for Pd and triple-zeta basis set 6-311++G(d,p)[15] for all other elements were used. Grimme's D3 empirical dispersion correction[16] were employed. Because the coupled cluster calculations were performed in the gas phase, no solvent correction was performed, and the structure of **1a** was reoptimized. Geometry optimizations were carried out using Becke's three-parameter hybrid exchange-correlation functional B3LYP[18] in conjunction with frequency calculation to confirm that the structure was minimal on the potential energy surface.

Single point calculations were performed with Coupled Cluster with Singles, Doubles and perturbative Triples (CCSD(T)) on the ORCA quantum chemistry package version 4.2.1. All such calculations were performed with Domain-based Local Pair Natural Orbitals (DLPNO), the TightPNO setting, the TightSCF convergence criteria, and the Ahlrich's type triple-zeta basis set def2-TZVPP. The RIJCOSX approximation was implemented using the def2-TZVPP/C and def2/J auxiliary basis sets. All DLPNO-CCSD(T) calculations were performed in the gas phase. For consistency between open and closed shell calculations, the setting

UseFullLMP2Guess was set to False. The T1 diagnostic for each structure is recorded, which never exceeded 0.015, indicating good single-reference behavior for all structures.

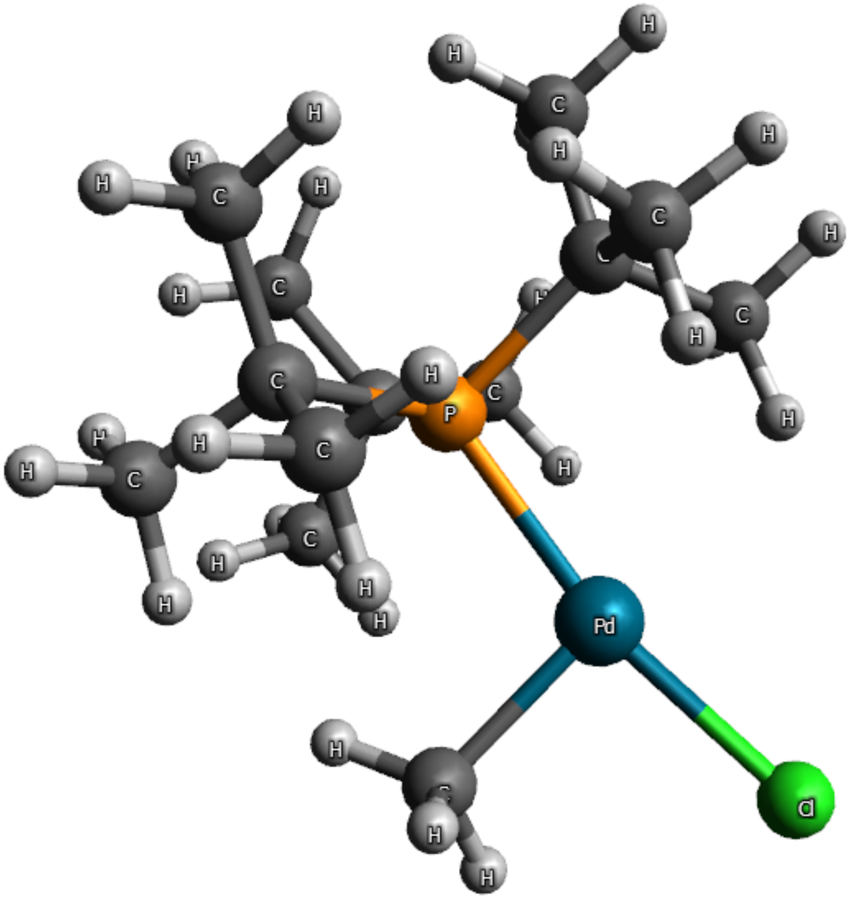

Figure 177. Optimized geometry of **1a** in the gas phase.

Table 29. Cartesian coordinates for **1a** S_0 optimized geometry.

**Tag**	**Symbol**	**X**	**Y**	**Z**
1	C	2.97798	-0.35956	1.84418
2	C	1.44059	-0.31132	1.76701
3	C	0.85547	-1.63955	2.30111
4	H	3.39683	-1.2023	1.29499
5	H	3.44703	0.55315	1.47773
6	H	-0.23398	-1.60155	2.33926
7	H	1.14918	-2.5118	1.72245

8	C	0.92558	0.78401	2.72444
9	H	-0.16289	0.86886	2.69101
10	H	1.36453	1.76165	2.53154
11	C	0.95952	1.87376	-0.45937
12	C	2.31931	2.48302	-0.07719
13	C	0.73186	2.06795	-1.9739
14	H	3.15715	1.96206	-0.53991
15	H	2.48104	2.49994	1.00004
16	H	1.53403	1.65532	-2.58305
17	H	-0.21735	1.63843	-2.30088
18	C	-0.17293	2.67014	0.23772
19	H	-1.17102	2.33999	-0.0808
20	H	-0.13606	2.63451	1.32219
21	C	3.07724	-0.54612	-1.64274
22	C	1.6782	-1.08293	-1.28063
23	C	0.82111	-1.18674	-2.563
24	H	3.73168	-0.46012	-0.7757
25	H	3.03961	0.42228	-2.1391
26	H	0.70661	-0.23986	-3.08359
27	H	-0.17463	-1.5746	-2.34835
28	C	1.84143	-2.52377	-0.75075
29	H	0.88566	-2.98015	-0.49522
30	H	2.50521	-2.58938	0.10951
31	H	3.2729	-0.48148	2.89215
32	H	1.22251	-1.7898	3.32197
33	H	1.20139	0.50334	3.746
34	H	2.28637	-3.12901	-1.5472
35	H	3.54908	-1.24592	-2.34111
36	H	1.31428	-1.88026	-3.25255
37	H	-0.0934	3.72112	-0.06047

38	H	0.69281	3.14282	-2.17808
39	H	2.34806	3.52237	-0.42296
40	P	0.72565	0.00393	0.00141
41	Pd	-1.59428	-0.08846	-0.0068
42	Cl	-3.9164	0.29606	-0.01195
43	C	-1.84309	-2.11105	-0.05724
44	H	-2.72678	-2.28545	0.5524
45	H	-2.03796	-2.34826	-1.10305
46	H	-0.9744	-2.63555	0.3276

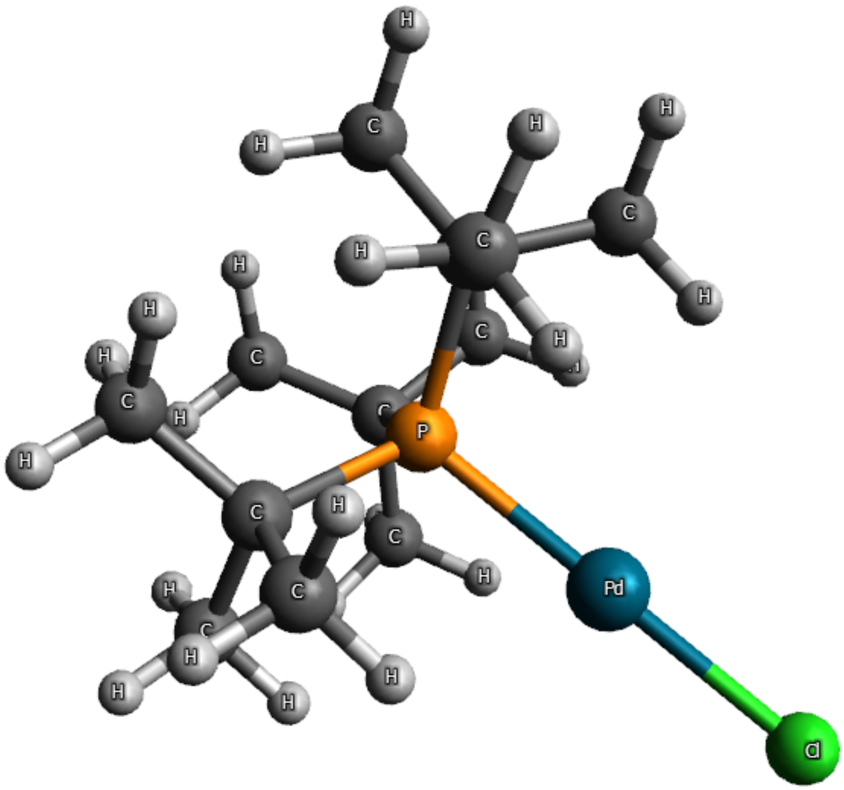

Figure 178. Optimized geometry of (tBu$_3$P)Pd(Cl) in the gas phase.

Table 30. Cartesian coordinates for (tBu$_3$P)Pd(Cl) D$_1$ optimized geometry.

**Tag**	**Symbol**	**X**	**Y**	**Z**

1	C	-2.77074	0.16783	-2.00703
2	C	-1.29438	0.51446	-1.74259
3	C	-0.40584	-0.16112	-2.81449
4	H	-2.94801	-0.90703	-2.02641
5	H	-3.44395	0.61123	-1.27336
6	H	0.64665	0.10789	-2.69037
7	H	-0.47797	-1.24505	-2.82308
8	C	-1.09783	2.03322	-1.93037
9	H	-0.06974	2.33854	-1.72309
10	H	-1.77359	2.62799	-1.31779
11	C	-1.28102	1.24937	1.32535
12	C	-2.77021	1.61942	1.20319
13	C	-1.02448	0.66263	2.72935
14	H	-3.42523	0.75057	1.26354
15	H	-2.9922	2.14955	0.2774
16	H	-1.67198	-0.18135	2.96206
17	H	0.01646	0.3565	2.85746
18	C	-0.42411	2.5357	1.24171
19	H	0.64278	2.3171	1.33893
20	H	-0.57167	3.09897	0.32461
21	C	-2.74051	-1.86337	0.8436
22	C	-1.2616	-1.77625	0.42597
23	C	-0.37489	-2.34704	1.55898
24	H	-3.41631	-1.47525	0.0818
25	H	-2.93911	-1.3361	1.77646
26	H	-0.48253	-1.82313	2.50449
27	H	0.68361	-2.33924	1.28478
28	C	-1.02809	-2.69507	-0.79165
29	H	0.00635	-2.65055	-1.14026
30	H	-1.69152	-2.47416	-1.62619

31	H	-3.05412	0.55809	-2.99068
32	H	-0.72283	0.19477	-3.8006
33	H	-1.30838	2.28061	-2.97568
34	H	-1.22611	-3.72754	-0.48669
35	H	-2.99979	-2.91511	1.00737
36	H	-0.66286	-3.38983	1.72974
37	H	-0.70298	3.19069	2.07384
38	H	-1.23035	1.44093	3.47098
39	H	-3.03616	2.28875	2.02877
40	P	-0.63779	0.00131	-0.00035
41	Pd	1.70216	0.00707	-0.00503
42	Cl	4.05654	0.00991	-0.00727

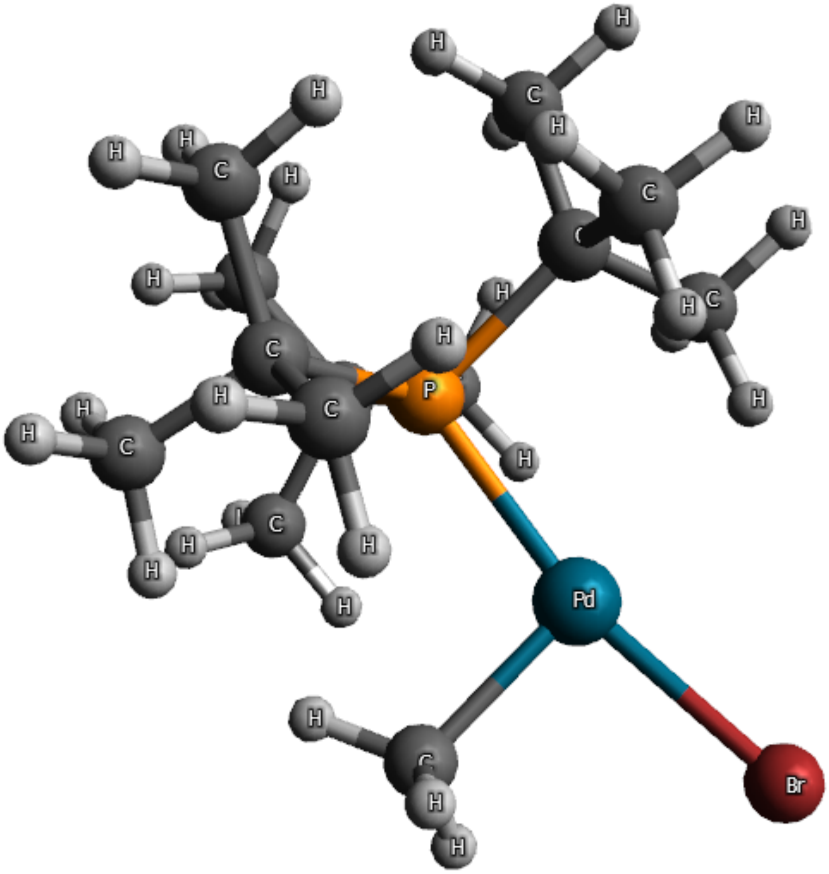

Figure 179. Optimized geometry of **1b** in the gas phase.

Table 31. Cartesian coordinates for **1b** S_0 optimized geometry.

**Tag**	**Symbol**	**X**	**Y**	**Z**
1	C	3.36673	-0.26221	1.83583
2	C	1.82854	-0.28362	1.76254
3	C	1.30471	-1.64205	2.28415
4	H	3.82256	-1.07957	1.27792
5	H	3.79178	0.6744	1.4762
6	H	0.21446	-1.65604	2.31788
7	H	1.64129	-2.4948	1.69979
8	C	1.26696	0.77904	2.73046
9	H	0.17571	0.81551	2.69985
10	H	1.66132	1.77697	2.5449
11	C	1.24444	1.89487	-0.44738
12	C	2.57804	2.56068	-0.06709
13	C	0.99947	2.08928	-1.95929
14	H	3.43562	2.08123	-0.53833
15	H	2.74429	2.5755	1.00953
16	H	1.81324	1.7139	-2.57701
17	H	0.06611	1.62394	-2.28293
18	C	0.08201	2.63576	0.26108
19	H	-0.90244	2.26234	-0.05212
20	H	0.12789	2.59666	1.34509
21	C	3.45964	-0.41502	-1.66121
22	C	2.09188	-1.02226	-1.29103
23	C	1.22774	-1.16291	-2.56494
24	H	4.1151	-0.30065	-0.79806
25	H	3.37069	0.55239	-2.15246
26	H	1.05182	-0.21906	-3.07389
27	H	0.25856	-1.60962	-2.34199

28	C	2.33326	-2.45578	-0.7709
29	H	1.40492	-2.96345	-0.51126
30	H	3.00619	-2.49268	0.08392
31	H	3.66998	-0.37965	2.88195
32	H	1.67456	-1.78312	3.3053
33	H	1.55755	0.50233	3.749
34	H	2.80245	-3.03228	-1.57477
35	H	3.96048	-1.0876	-2.3661
36	H	1.75145	-1.82018	-3.26742
37	H	0.11198	3.69062	-0.03244
38	H	0.91513	3.163	-2.15571
39	H	2.55881	3.60317	-0.40393
40	P	1.09594	0.01316	0.00173
41	Pd	-1.22819	-0.17674	-0.00257
42	Br	-3.67972	0.16458	-0.00723
43	C	-1.38776	-2.20968	-0.06011
44	H	-2.20509	-2.44237	0.6189
45	H	-1.65847	-2.44234	-1.08981
46	H	-0.46417	-2.69321	0.24062

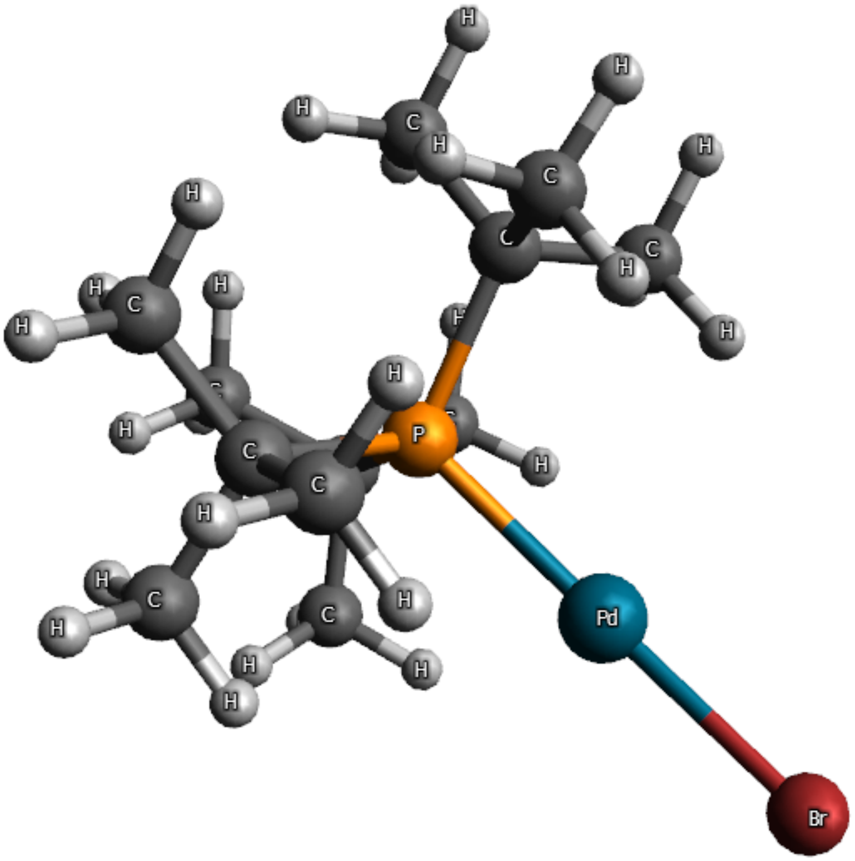

Figure 180. Optimized geometry of (tBu3P)Pd(Br) in the gas phase.

Table 32. Cartesian coordinates for (tBu3P)Pd(Br) D_1 optimized geometry.

Tag	Symbol	X	Y	Z
1	C	-3.14438	1.89384	0.76441
2	C	-1.6601	1.54516	0.97698
3	C	-0.78574	2.75989	0.58207
4	H	-3.35742	2.19565	-0.26091
5	H	-3.8105	1.07104	1.0232
6	H	0.27669	2.56416	0.75132
7	H	-0.91141	3.07159	-0.451
8	C	-1.4092	1.31955	2.48278
9	H	-0.37022	1.04852	2.6847

10	H	-2.0621	0.56279	2.91476
11	C	-1.67709	-1.60971	0.85403
12	C	-3.15246	-1.57214	1.29181
13	C	-1.46919	-2.80363	-0.10085
14	H	-3.83197	-1.37491	0.46273
15	H	-3.33656	-0.82963	2.06782
16	H	-2.13984	-2.785	-0.95842
17	H	-0.43855	-2.86301	-0.4583
18	C	-0.78365	-1.89423	2.08546
19	H	0.2717	-1.96779	1.80881
20	H	-0.87531	-1.15111	2.87259
21	C	-3.18308	-0.29947	-1.97788
22	C	-1.69869	0.0739	-1.81442
23	C	-0.83161	-0.86457	-2.68763
24	H	-3.84218	0.32941	-1.37975
25	H	-3.38059	-1.34012	-1.72216
26	H	-0.936	-1.91636	-2.4359
27	H	0.2287	-0.60398	-2.6271
28	C	-1.47563	1.49381	-2.37555
29	H	-0.43879	1.81766	-2.25939
30	H	-2.13097	2.23736	-1.92481
31	H	-3.40364	2.741	1.40898
32	H	-1.06888	3.60815	1.21439
33	H	-1.61067	2.25792	3.00925
34	H	-1.69536	1.47792	-3.4478
35	H	-3.46683	-0.16734	-3.02782
36	H	-1.14052	-0.7472	-3.73203
37	H	-1.08008	-2.85878	2.51119
38	H	-1.67762	-3.72574	0.45103
39	H	-3.42072	-2.54822	1.71099

40	P	-1.03759	0.0004	-0.00132
41	Pd	1.31129	-0.00806	-0.0112
42	Br	3.78627	0.00019	-0.00454

Table 33. Cartesian coordinates for (tBu_3P)Pd(Br) D_1 optimized geometry.

Tag	Symbol	X	Y	Z
1	C	0	0	0
2	H	0.2413	-1.05356	0
3	H	-0.12065	0.52677	0.93604
4	H	-0.12065	0.52677	-0.93604

Table 34. Results of B3LYP-D3 geometry optimization and DLPNO-CCSD(T) single point calculations.

Structure	B3LYP-D3 Enthalpy Correction	B3LYP-D3 Free Energy Correction	CCSD(T) Electronic Energy	CCSD(T) Enthalpy	CCSD(T) Free Energy	CCSD(T) T1 Diagnostic
Me	0.033603	0.009831	-39.76178589	-39.728183	-39.7519549	0.0146402
**1a**	0.432874	0.357818	-1440.904487	-1440.4716	-1440.54667	0.0130179
(tBu_3P) Pd(Cl)	0.393786	0.322223	-1401.062324	-1400.6685	-1400.7401	0.0135047
**1b**	0.432844	0.356596	-3553.890554	-3553.4577	-3553.53396	0.0122411
(tBu_3P) Pd(Br)	0.393649	0.320498	-3514.049068	-3513.6554	-3513.72857	0.0129747

Table 35. BDE and BDFE of **1a** and **1b**.

Structure	BDE	BDFE
**1a**	47.0	34.3
**1b**	46.5	33.5

UV-vis spectroscopy

Absorption spectra were recorded on an Agilent Cary 60 spectrophotometer. Samples for measurement were made up inside a N_2-filled glovebox and sealed in a 2 mm quartz cuvette capped with a J-Young valve. Samples for low temperature measurements were prepared inside a N_2-filled glove box and sealed in a J-Young capped thick-wall quartz NMR tube. The NMR tube was then submerged into liquid N_2 held in a quartz flask and equilibrated for 15 minutes. Formation of glass state was eye-checked as a good glass state should preserve perfect transparency. The whole setup including NMR tube and quartz liquid N_2 flask was then transferred to sampling chamber for absorption measurement.

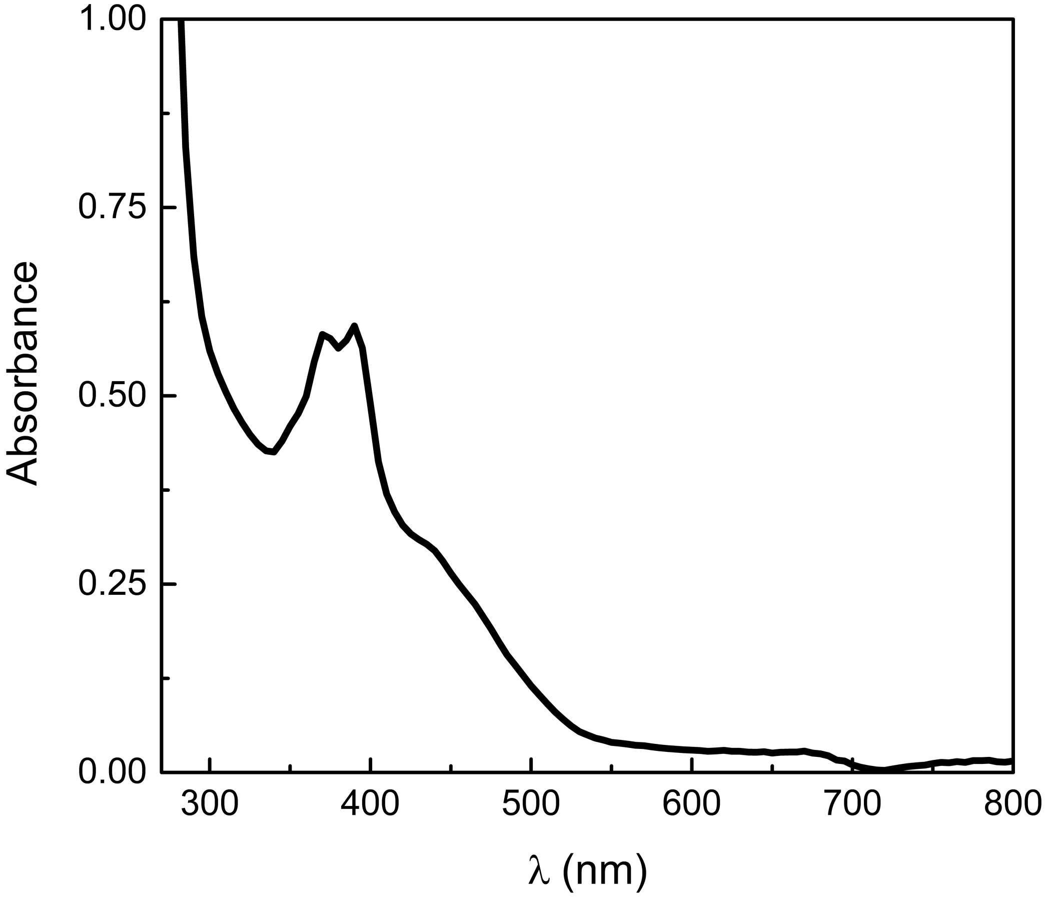

Figure S181. Absorption spectrum of **3** in benzene at room temperature.

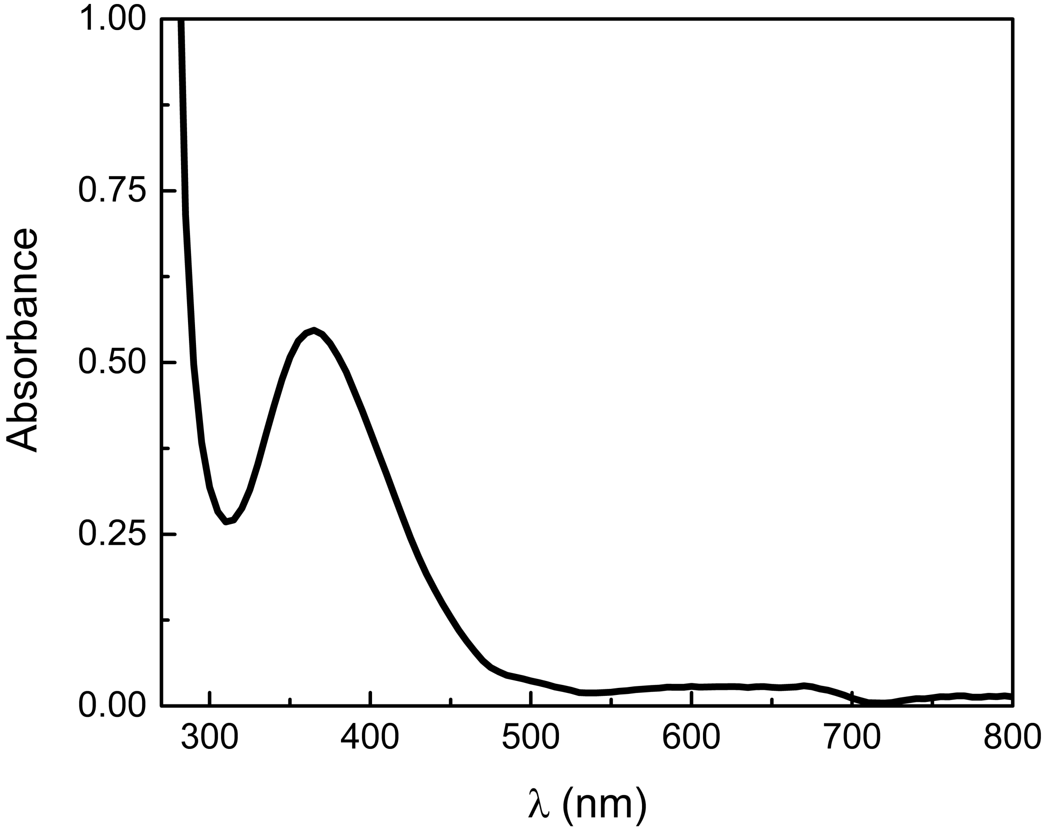

Figure S182. Absorption spectrum of **1a** in benzene at room temperature.

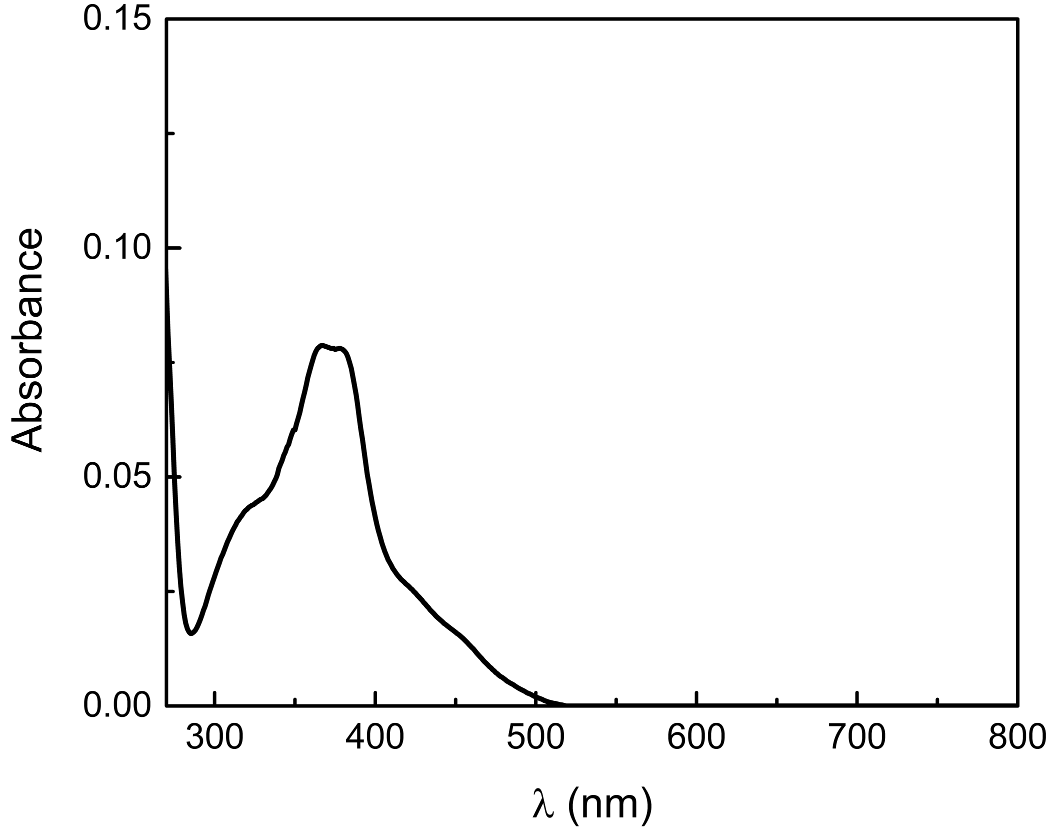

Figure S183. Absorption spectrum of **3** in $CHCl_3$ at room temperature.

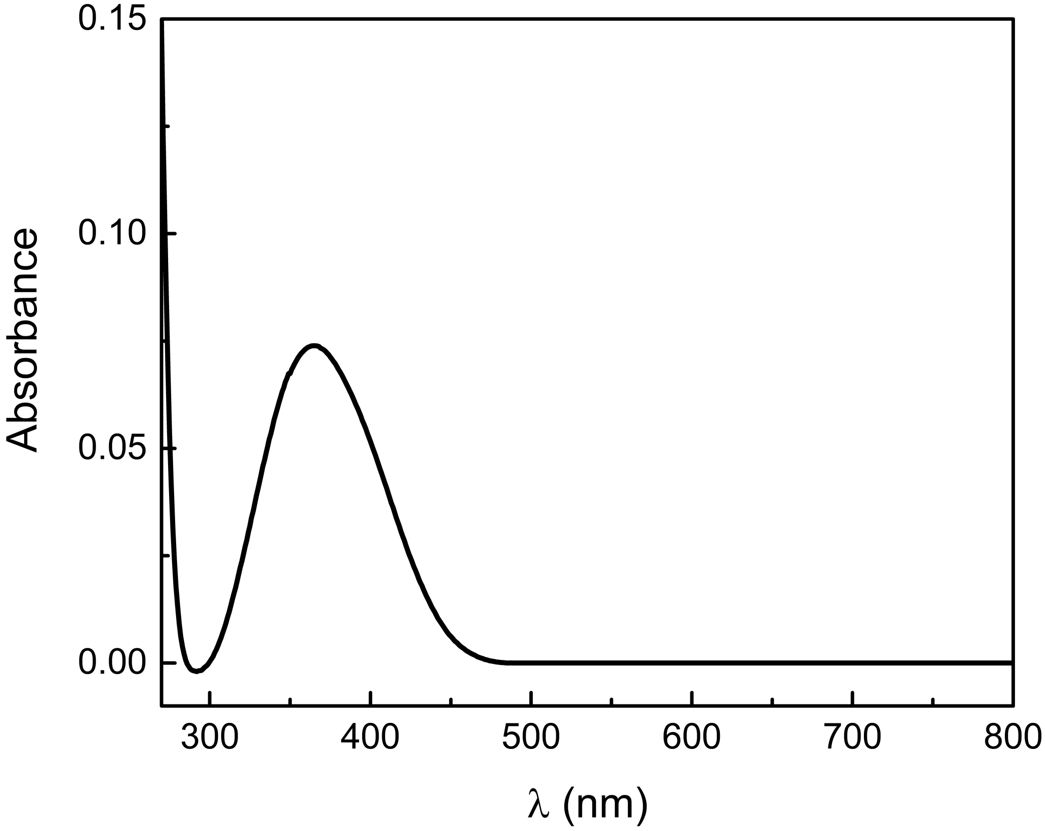

Figure S184. Absorption spectrum of **1a** in $CHCl_3$ at room temperature.

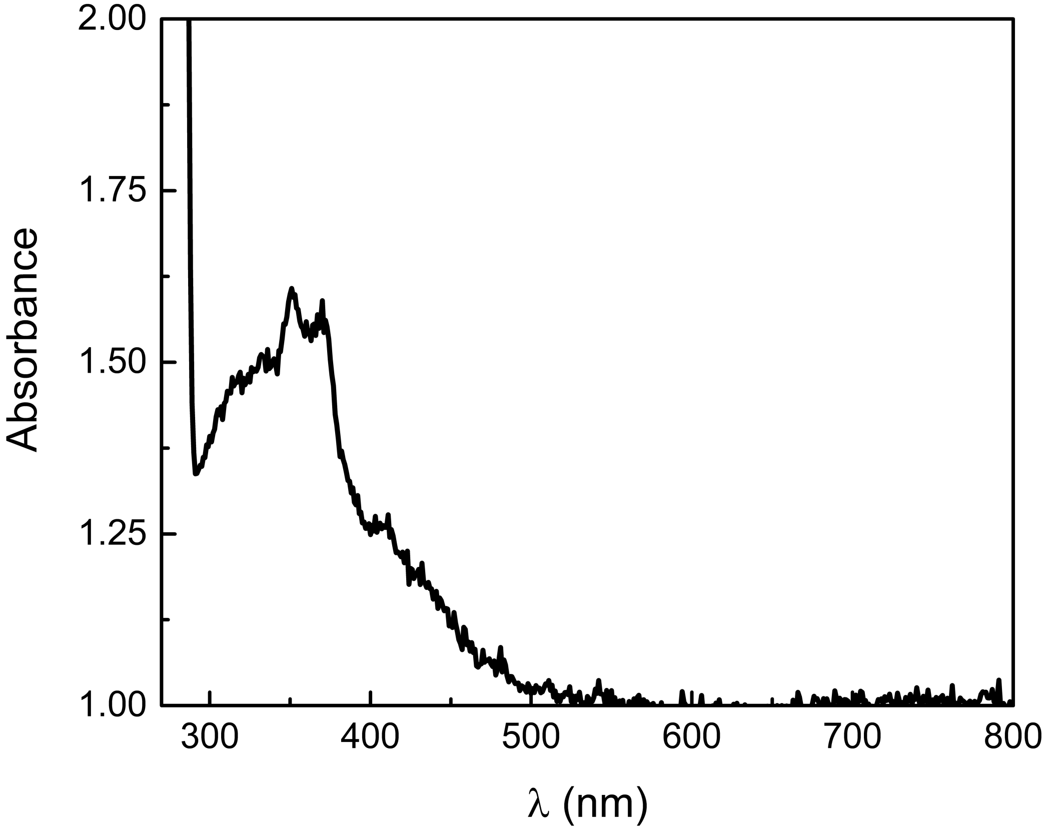

Figure S185. Absorption spectrum of **3** in 2-methyl-THF glass state at 77 K. High background due to heavy scattering caused by the use of NMR tube with a curved surface instead of a proper absorption cuvette.

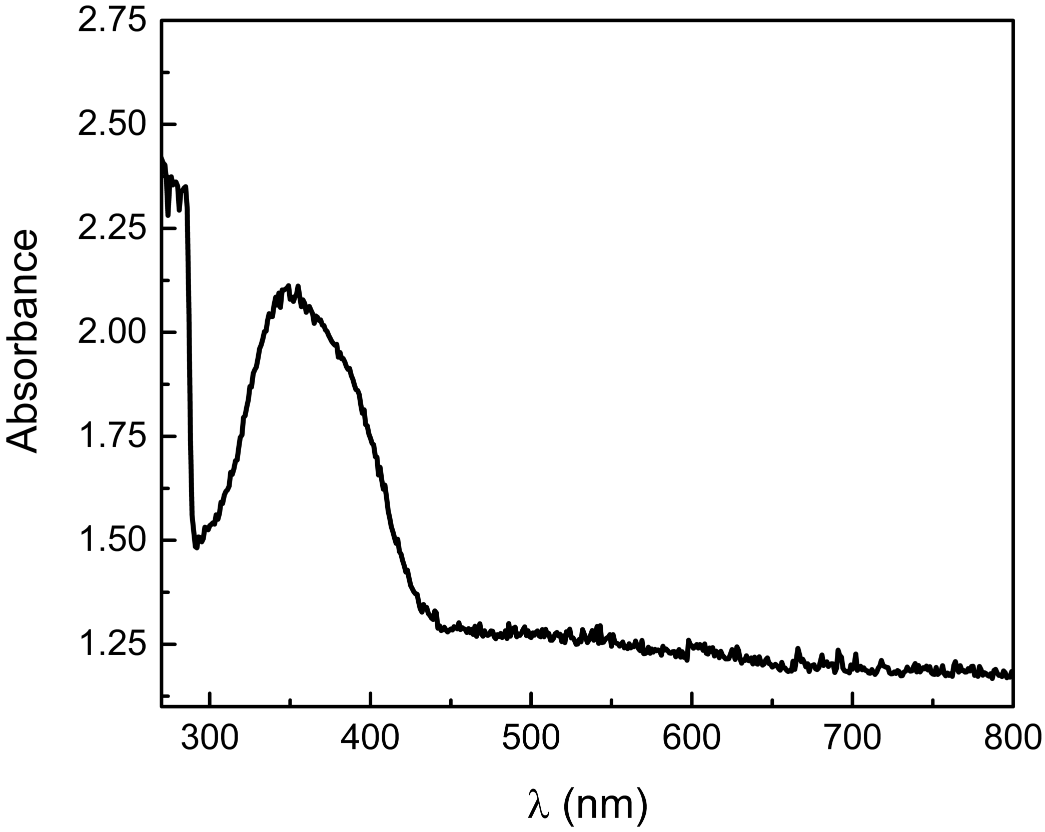

Figure S186. Absorption spectrum of **1a** in 2-methyl-THF glass state at 77 K. High background due to heavy scattering caused by the use of NMR tube with a curved surface instead of a proper absorption cuvette.

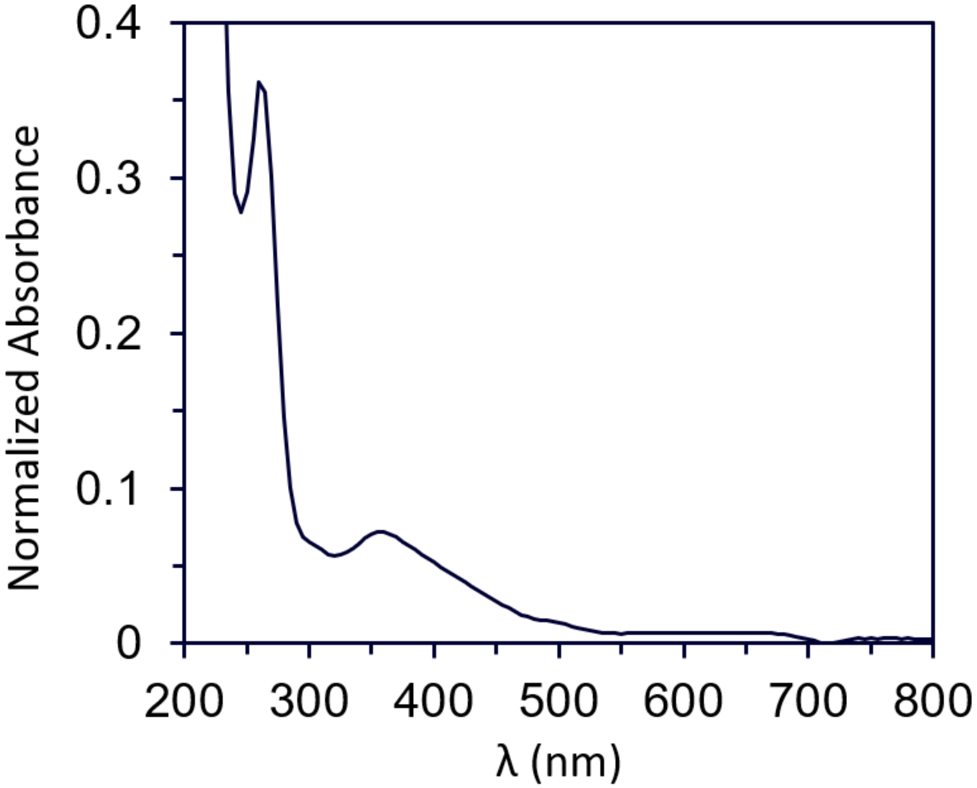

Figure S187. Absorption spectrum of **2** in THF at room temperature.

Fluorescence spectroscopy

Emission spectra were recorded on an Agilent Cary Eclipse fluorescence spectrophotometer. Samples for measurement were made up inside a N_2-filled glovebox and sealed in a 1 cm quartz cuvette capped with a septum cap. Sample absorbance at the wavelength of photoexcitation was never above 0.1 to avoid any inner filter effects.[25, 26] Samples for low temperature measurements were prepared inside a N_2-filled glove box and sealed in a J-Young capped thick-wall quartz NMR tube. The NMR tube was then submerged into liquid N_2 held in a quartz flask and equilibrated for 15 minutes. Formation of glass state was eye-checked as a good glass state should preserve perfect transparency. The whole setup including NMR tube and quartz liquid N_2 flask was then transferred to sampling chamber for emission measurement.

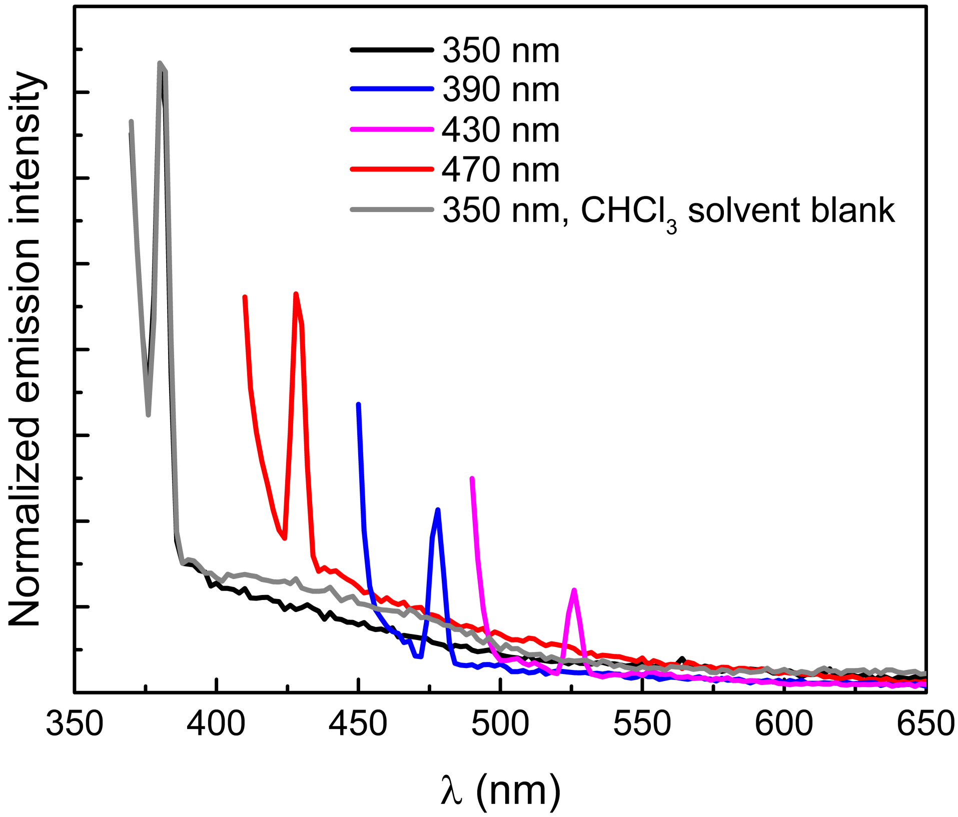

Figure S188. Room temperature emission measurements of **3** in $CHCl_3$ at different excitation wavelengths versus solvent blank. No obvious sign of luminescence was observed.

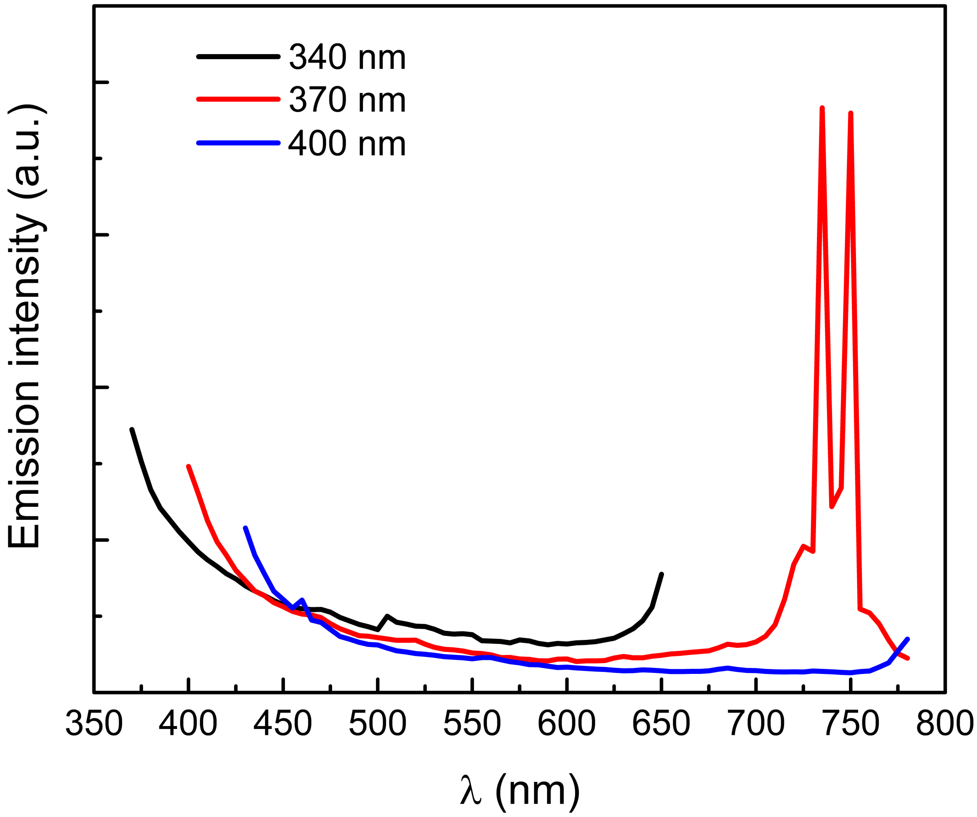

Figure S189. 77 K emission measurements of **3** in 2-methyl-THF glass state at different excitation wavelengths. The huge peak at 740 nm is the second order of 370 nm excitation scattering. No obvious sign of luminescence was observed.

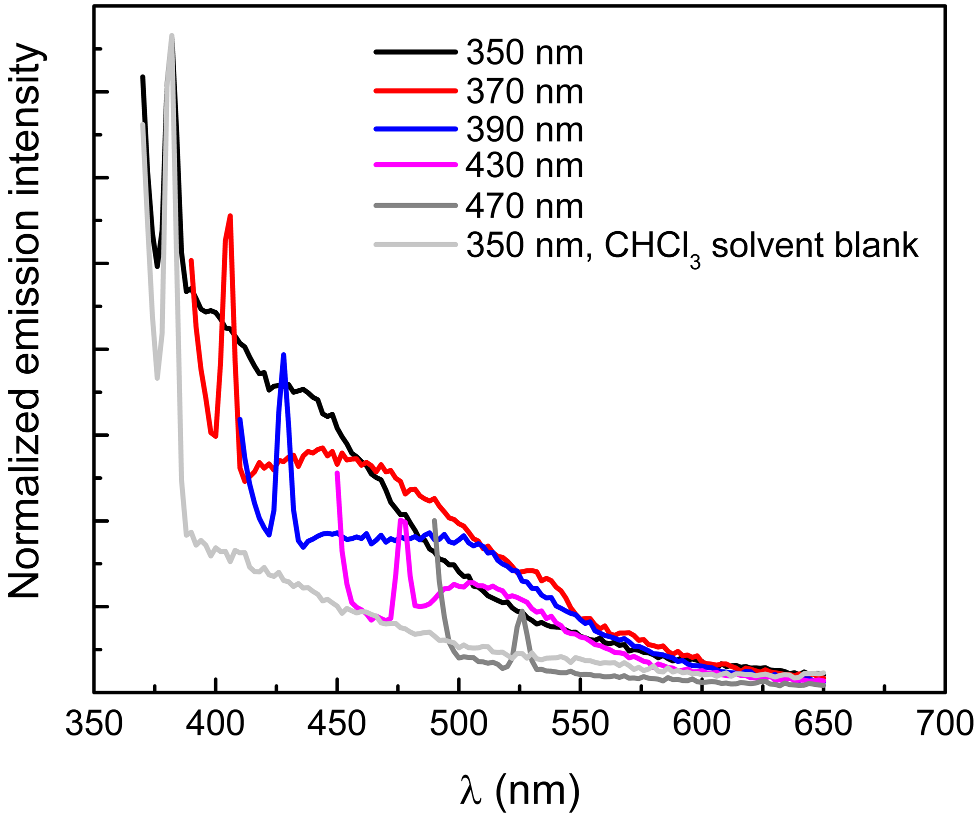

Figure S190. Room temperature emission measurements of **1a** in $CHCl_3$ at different excitation wavelengths versus solvent blank. Narrow and broad peaks all shift while excitation wavelengths are varied, suggesting no sigh of luminescence.

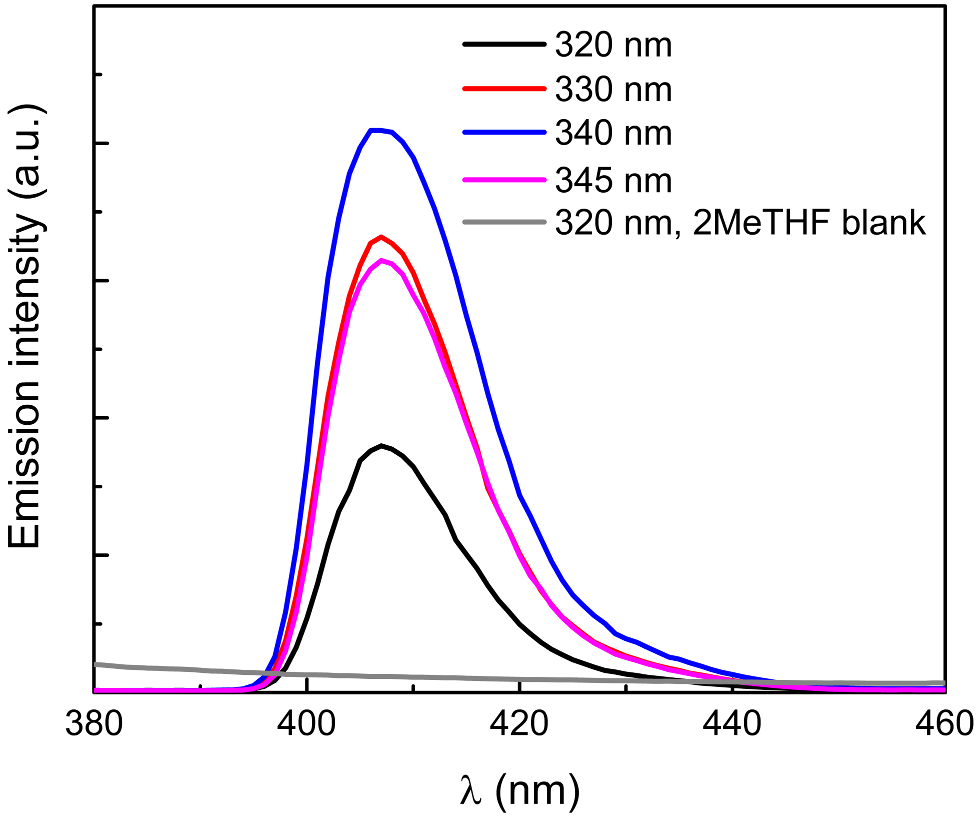

Figure S191. 77 K emission measurements of **1a** in 2-methyl-THF glass state at different excitation wavelengths versus solvent blank. An emission feature peaked around 408 nm was observed.

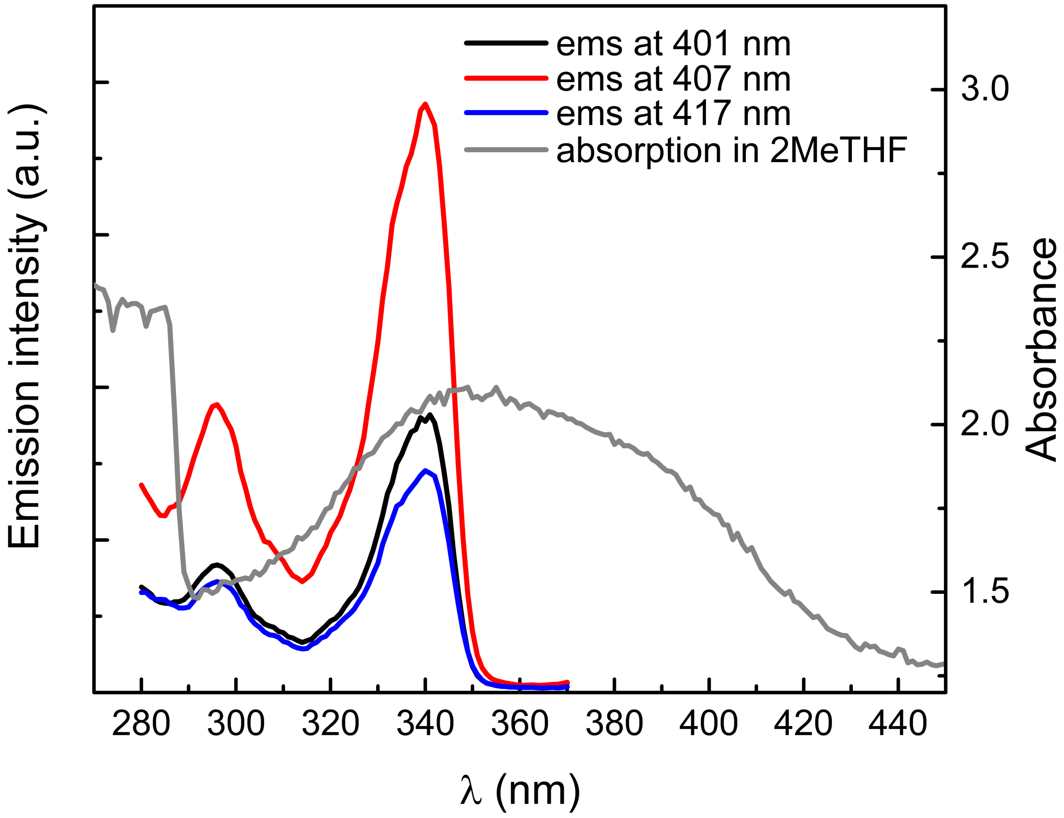

Figure S192. 77 K excitation scan of **1a** in 2-methyl-THF glass state monitored at different emission wavelengths versus absorption spectrum. Excitation scan does not match the absorption spectrum, suggesting that the emissive species is unlikely to be **1a**.

Transient absorption spectroscopy

For transient absorption measurements, a 1 kHz regeneratively amplified Ti:Sapphire laser (Coherent Libra, Santa Clara, California) with a commercial optical parametric amplifier (OPerA Solo, Vilnius, Lithuania) and commercial transient absorption spectrometer (Ultrafast Systems Helios, Sarasota, Florida) were used with a detailed setup description reported previously.[27] In brief, the output pulse of the Ti:Sapphire laser Coherent Libra, centered at 800 nm with a duration of ca. 45 fs and pulse power of 4 W, is split with a 90-10 (r-t) beamsplitter to generate the pump and probe. The reflected portion of the output 800 nm is directed into the commercial optical parametric amplifier OperA Solo to generate light at 370 and 450 nm. Both the ouput of the OperA Solo and transmitted portion of the output 800 nm are directed into a commercial transient absorption spectrometer Helios. The pump pulse is chopped at 500 Hz with its polarization controlled by a broadband λ/2 waveplate and then focused into the sample position. The remaining 800 nm is mechanically delayed and then focused into a 2 mm thick translating CaF_2 crystal to generate a white light continuum from 325 nm to 800 nm with its intensity and polarization controlled by a combination of λ/2 waveplate and polarizer. After filtering the remaining fundamental light, the continuum is tuned to overlap with pump pulse and focused into the sample position and eventually enters a CCD camera for detection. All experiments on this setup were done at magic angle to avoid reorientation effects of the samples on nanosecond timescale. Samples for measurement were made up inside a N2-filled glovebox and sealed in a 2 mm quartz cuvette capped with a J-Young valve. Sample absorbance at the wavelength of photoexcitation was never above 0.5 to avoid non-linear effects. Pre and post scan absorption measurements were performed to ensure the photodegradation was minimal during the measurements (absorbance change less than 10%). Since the photolysis of both Pd complexes are quite severe, samples were refreshed every 15-20 minutes and monitored with absorption spectrometer to minimize the photodegradation.

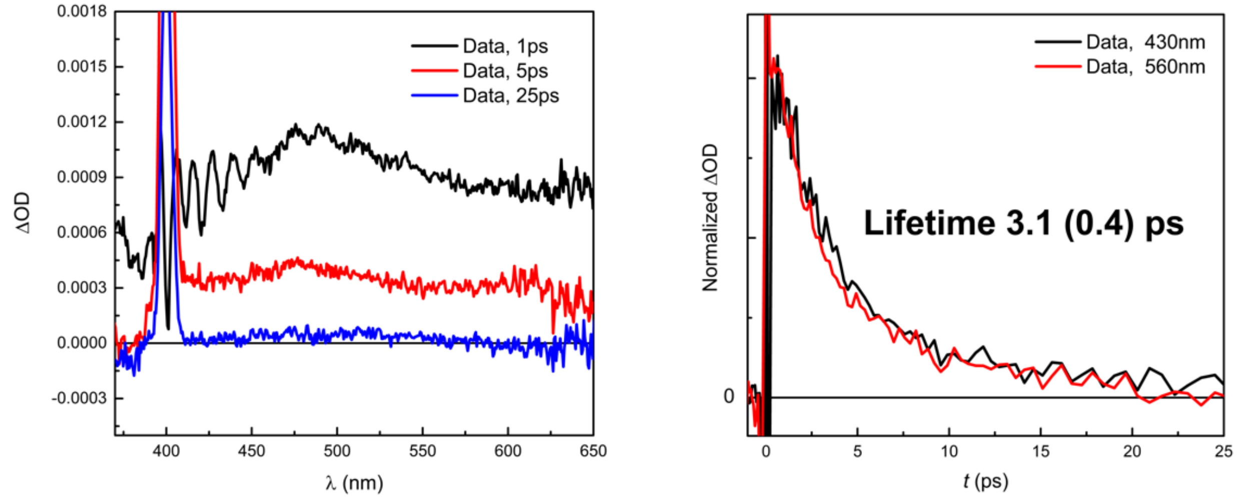

Figure S193. Transient absorption spectra (left) and single wavelength traces (right) of **3** in $CDCl_3$ after photoexcitation at 400 nm, 100 μW.

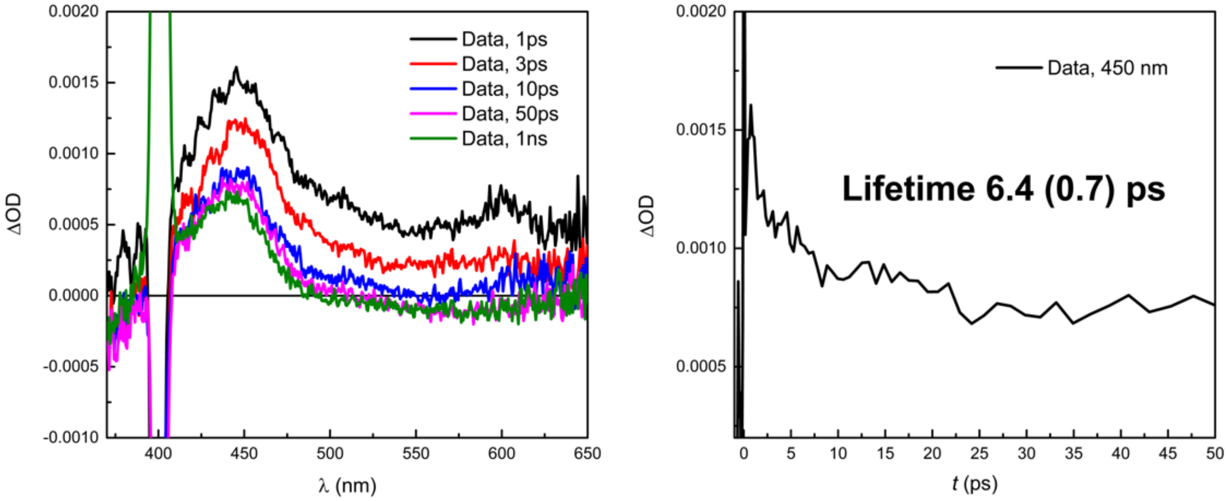

Figure S194. Transient absorption spectra (left) and single wavelength traces (right) of **1a** in $CDCl_3$ after photoexcitation at 400 nm, 100 μW.

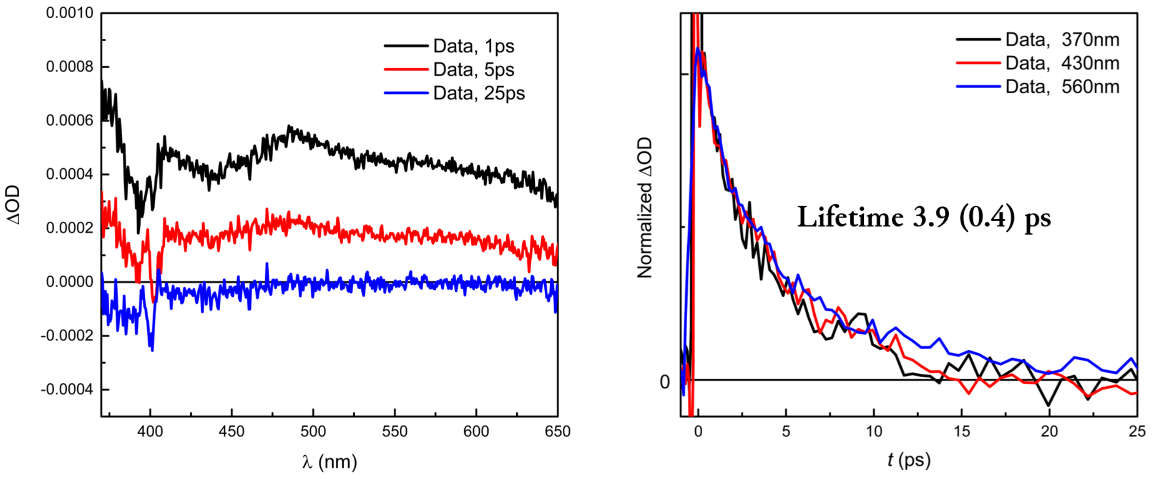

Figure S195. Transient absorption spectra (left) and single wavelength traces (right) of **3** in benzene after photoexcitation at 400 nm, 150 μW.

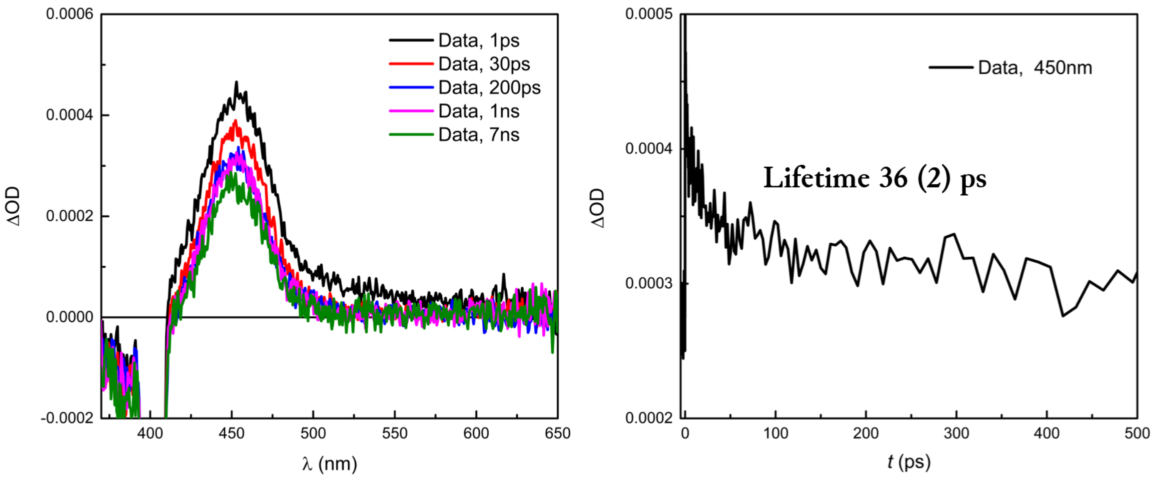

Figure S196. Transient absorption spectra (left) and single wavelength traces (right) of **1a** in benzene after photoexcitation at 400 nm, 40 μW. The residual signal peaked at 450 nm lives up to microsecond timescale.

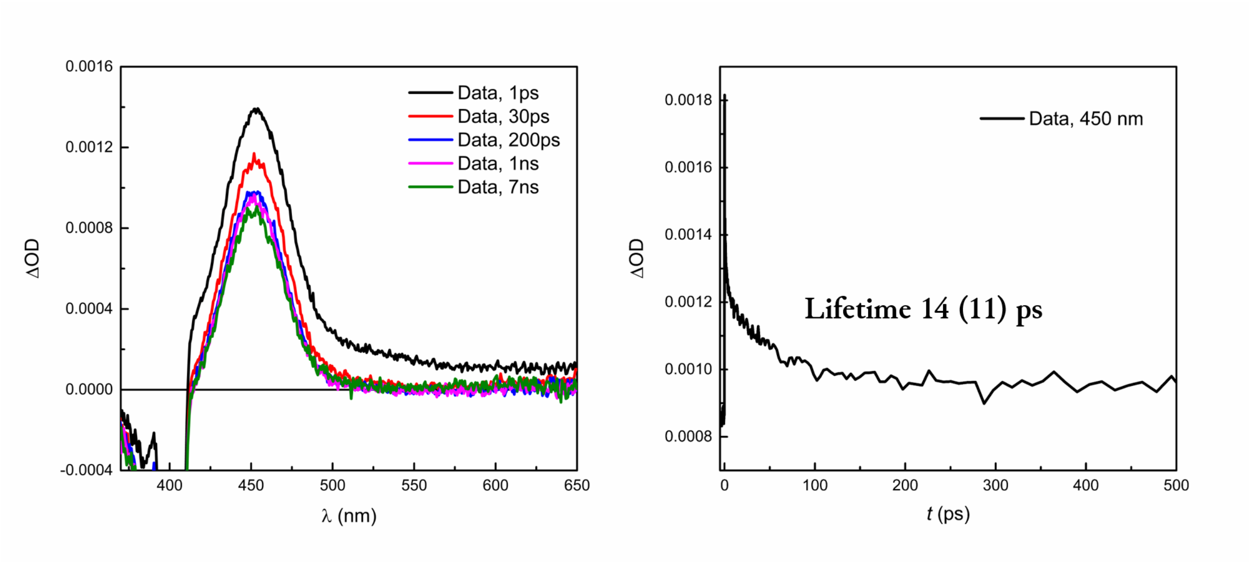

Figure S197. Transient absorption spectra (left) and single wavelength traces (right) of **1a** in benzene after photoexcitation at 400 nm, 100 μW. The residual signal peaked at 450 nm lives up to microsecond timescale.

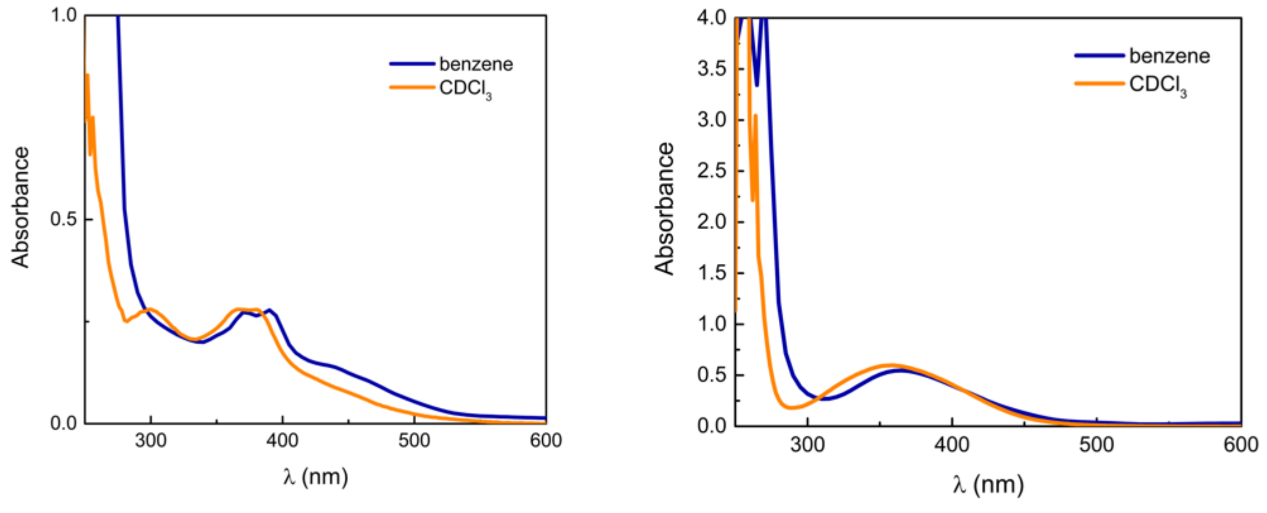

Figure S198. Comparison of the absorption spectra between benzene (blue curves) and $CDCl_3$ (orange curves) for **3** (left) and **1a** (right).

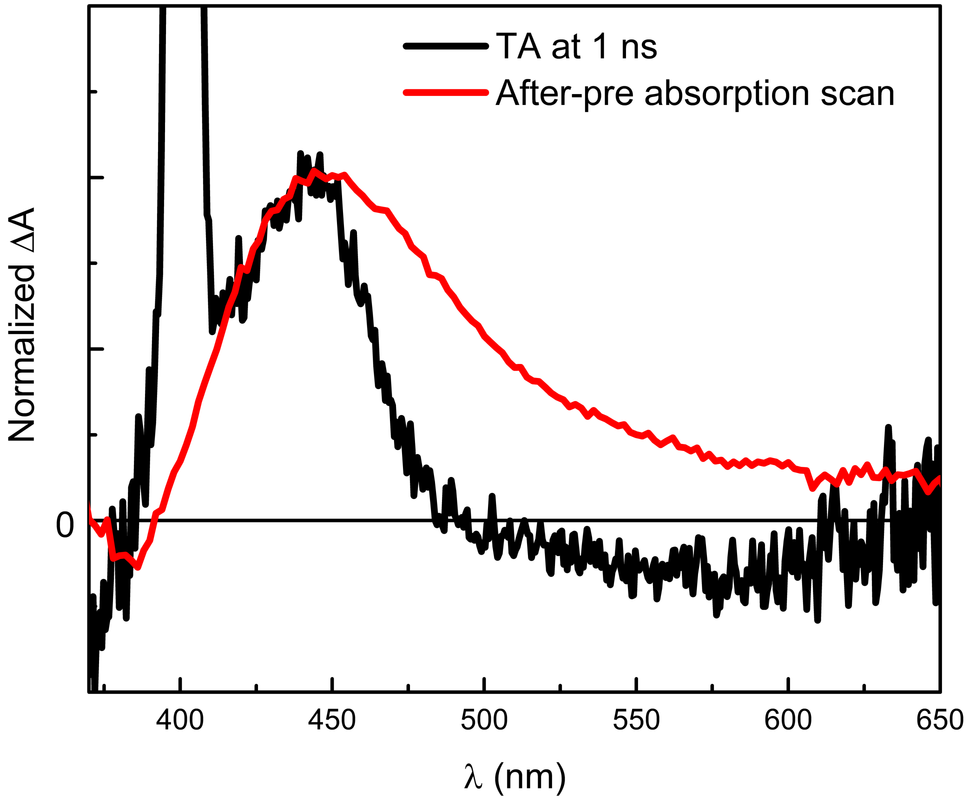

Figure S199. Comparison between transient absorption and steady-state difference absorption spectra of **1a** in $CDCl_3$ before and after photoexcitation at 400 nm, 100 μW.

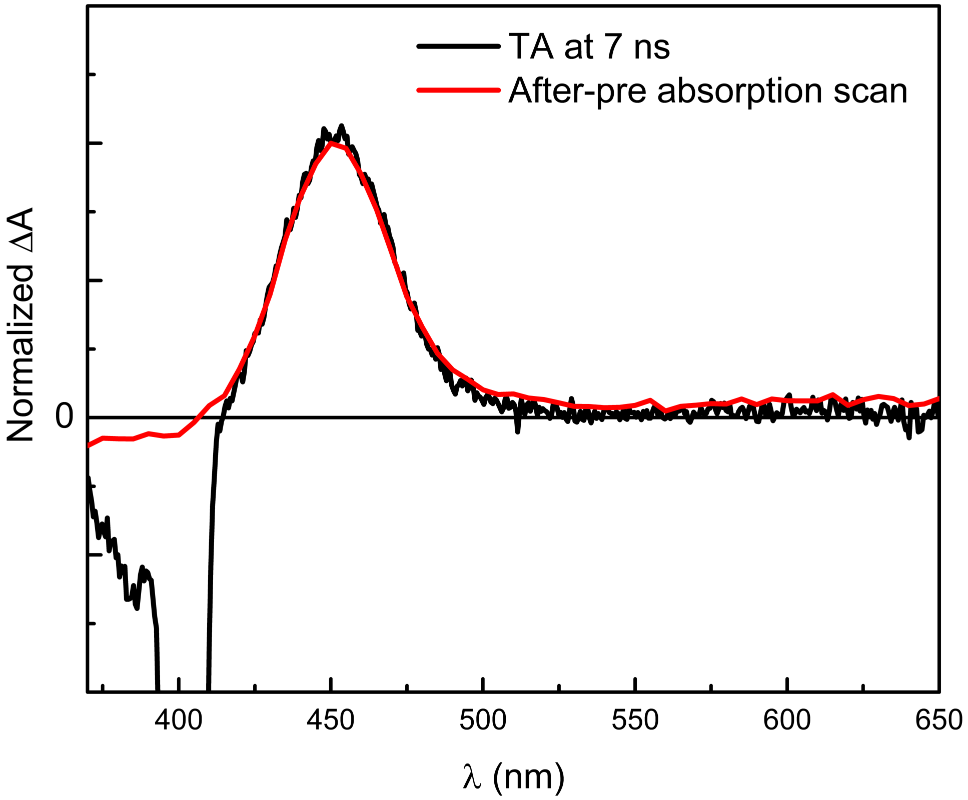

Figure S200. Comparison between transient absorption and steady-state difference absorption spectra of **1a** in benzene before and after photoexcitation at 400 nm, 100 μW.

X-ray crystallographic data

Crystals were mounted on MiTeGen loops under oil at 100 K. Disorder in structures **5b** and **S2** was modelled by the "SQUEEZE" method in Platon.

Table S36. Instrumental and computational data for **1b**, **2**, **5a**, and **5b**.

**Structure**	**1b**	**2**	**5a**	**5b**
Radiation type/source	Mo Kα fine focus tube	Mo Kα fine focus tube	Mo Kα fine focus tube	Mo Kα IμS micro-focus source
Diffractometer measurement device type	Bruker Apex II CCD	Bruker Photon 100 CMOS	Bruker Apex II CCD	Bruker Photon 100 CMOS
Data collection	Bruker Instrument Service v2014.11.0.1	Bruker Instrument Service vV6.2.6	Bruker Instrument Service v2014.11.0.1	Bruker Instrument Service vV6.2.6
Cell refinement	APEX2 v2014.11-0 (Bruker AXS)	SAINT V8.38A (Bruker AXS Inc., 2017)	SAINT V8.34A (Bruker AXS Inc., 2013)	SAINT V8.38A (Bruker AXS Inc., 2017)
Data reduction	SAINT V8.34A (Bruker AXS Inc., 2013)	SAINT V8.38A (Bruker AXS Inc., 2017)	SAINT V8.34A (Bruker AXS Inc., 2013)	SAINT V8.38A (Bruker AXS Inc., 2017)
Structure solution	SHELXL-2014 (Sheldrick, 2014)	SHELXT (Sheldrick, 2016)	SHELXL-2014 (Sheldrick, 2014)	SHELXT (Sheldrick, 2016)
Structure refinement	SHELXL-2014 (Sheldrick, 2014)	SHELXL-2016/6 (Sheldrick, 2016)	SHELXL-2014/7 (Sheldrick, 2014)	SHELXL-2014/7 (Sheldrick, 2014)

Table S37. Instrumental and computational data for **4** and **S2**.

**Structure**	**4**	**S2**
Radiation type/source	Mo Kα fine focus tube	Cu Kα IμS micro-focus source
Diffractometer measurement device type	Bruker Apex II CCD	Bruker Apex II CCD
Data collection	Bruker Instrument Service v2014.11.0.1	Bruker Instrument Service v2014.11.0.1
Cell refinement	SAINT V8.34A (Bruker AXS Inc., 2013)	SAINT V8.34A (Bruker AXS Inc., 2013)
Data reduction	SAINT V8.34A (Bruker AXS Inc., 2013)	SAINT V8.34A (Bruker AXS Inc., 2013)
Structure solution	SHELXL-2014 (Sheldrick, 2014)	SHELXL-2014 (Sheldrick, 2014)
Structure refinement	SHELXL-2014/7 (Sheldrick, 2014)	SHELXL-2014/7 (Sheldrick, 2014)

Table S38. Crystal and refinement data for **1b**, **2**, **5a**, and **5b**.

Structure	1b	2	5a	5b
Empirical formula	$C_{13}H_{30}BrPPd$	$C_{31}H_{48}ClPPd$	$C_{21}H_{45}ClNOPPd$	$C_{21}H_{45}BrNOPPd$
Formula weight (g/mol)	403.65	593.51	500.40	544.86
T (K)	100(2)	100(2)	100(2)	100(2)
a (Å)	8.5537(2)	12.1765(11)	14.7317(3)	31.4611(9)
b (Å)	9.5525(2)	14.8521(14)	12.1009(3)	8.1772(2)
c (Å)	10.1463(2)	16.3152(15)	14.8868(3)	23.0772(6)
α (°)	91.5542(9)	86.928(3)	90	90
β (°)	91.4639(9)	74.898(3)	115.6637(10)	124.5770(9)
γ (°)	94.1010(9)	77.450(3)	90	90
Volume ($Å^3$)	826.31(3)	2780.6(4)	2392.03(9)	4888.3(2)
Z	2	4	4	8
Crystal system	triclinic	triclinic	monoclinic	monoclinic
Space group	P -1	P -1	P 1 21/c 1	C 1 2/c 1
d_{calc} (g/cm^3)	1.622	1.418	1.389	1.481
θ range (°)	2.01 to 40.25°	2.14 to 29.57°	2.27 to 43.11°	1.57 to 41.15°
μ (mm^{-1})	3.616	0.839	0.965	2.470
Abs. correction	Multi-scan	Multi-scan	Multi-scan	Multi-scan
GOF	1.057	1.099	1.008	1.011
R_1[a], wR_2[b] [I>2 σ(I)]	0.0155, 0.0371	0.0569, 0.1174	0.0186, 0.0423	0.0198, 0.0422
Radiation type	Mo Kα	Mo Kα	Mo Kα	Mo Kα

[a]$R_1 = \Sigma||F_o| - |F_c||/\Sigma|F_o|$. [b] $wR_2 = [\Sigma[w(F_o^2 - F_c^2)^2]/\Sigma[w(F_o^2)^2]]^{1/2}$; $w = 1/[\sigma^2(F_o^2) + (0.0175P)^2 + 0.2298P]$ where $P = (F_o^2 + 2F_c^2)/3$

Table S39. Crystal and refinement data for **1b**, **2**, **5a**, and **5b**.

Structure	4	S2
Empirical formula	$C_{16}H_{38}ClPPdSi$	$C_{39}H_{63}ClNOPPd$
Formula weight (g/mol)	431.37	734.72
T (K)	100(2)	100(2)
a (Å)	10.3300(4)	12.5212(4)
b (Å)	15.1751(6)	17.2596(6)
c (Å)	14.0651(6)	18.8102(7)
α (°)	90	90
β (°)	110.1259(18)	98.389(2)
γ (°)	90	90
Volume (Å^3)	2070.20(15)	4021.6(2)
Z	4	4
Crystal system	monoclinic	monoclinic
Space group	P 1 21/c 1	P 1 21/n 1
d_{calc} (g/cm^3)	1.384	1.213
θ range (°)	2.04 to 45.29°	3.49 to 66.60°
μ (mm^{-1})	1.153	4.994
Abs. correction	Multi-scan	Multi-scan
GOF	1.000	1.074
R_1^a, wR_2^b [I>2 σ(I)]	0.0233, 0.0497	0.0422, 0.1132
Radiation type	Mo Kα	Cu Kα

$^a R_1 = \Sigma||F_o| - |F_c||/\Sigma|F_o|$. ^{b} $wR_2 = [\Sigma[w(F_o^2 - F_c^2)^2]/\Sigma[w(F_o^2)^2]]^{1/2}$; $w = 1/[\sigma^2(F_o^2) + (0.0175P)^2 + 0.2298P]$ where $P = (F_o^2 + 2F_c^2)/3$

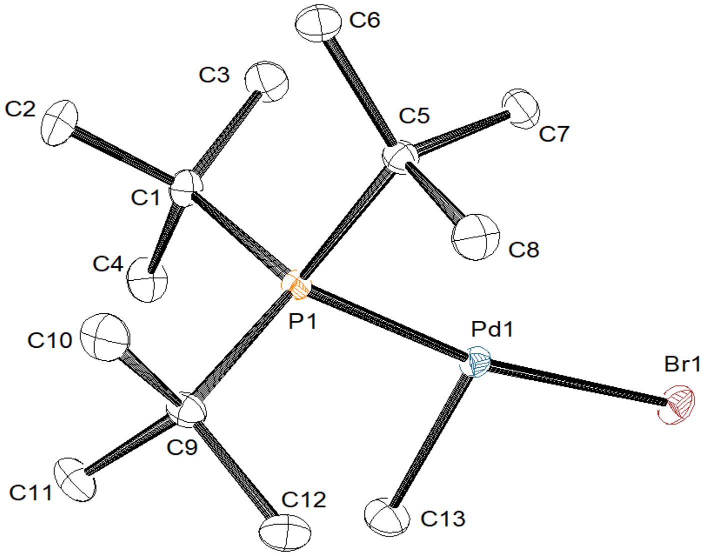

Figure S201. Structural drawing of **1b** with 50% probability anisotropic displacement ellipsoids.

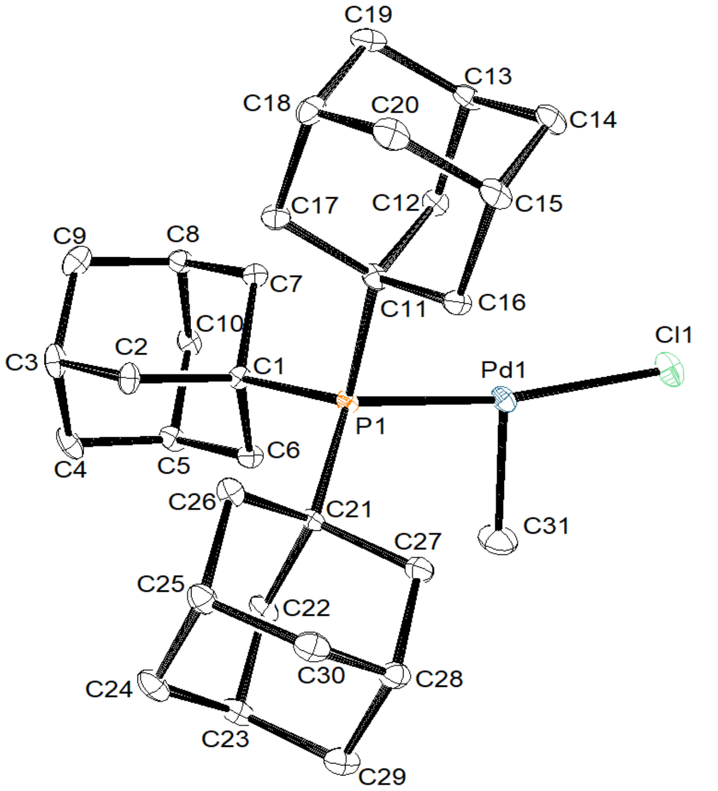

Figure S202. Structural drawing of **2** with 50% probability anisotropic displacement ellipsoids.

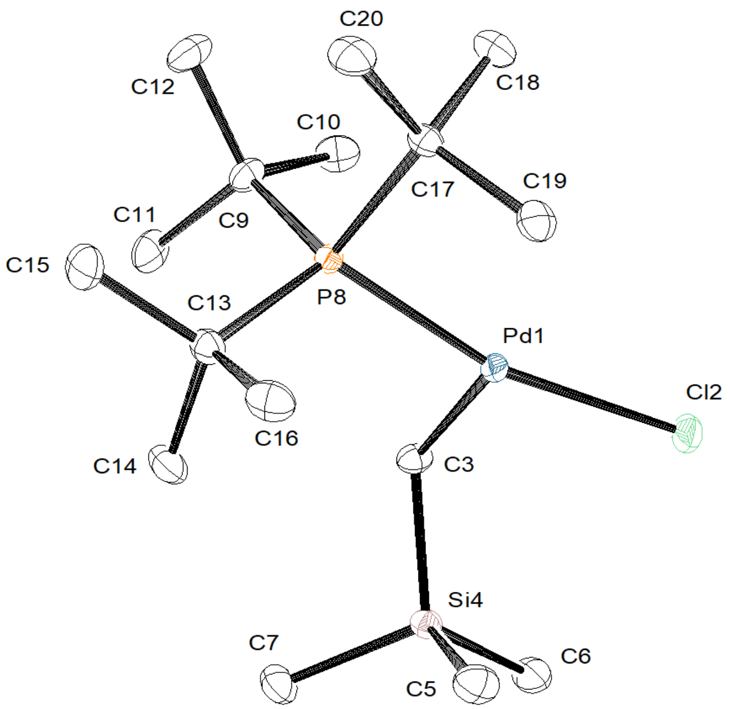

Figure S203. Structural drawing of **4** with 50% probability anisotropic displacement ellipsoids.

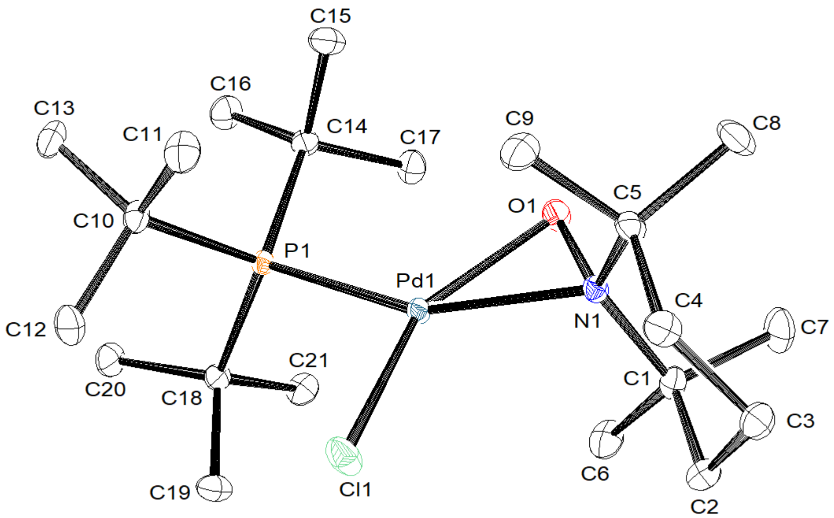

Figure S204. Structural drawing of **5a** with 50% probability anisotropic displacement ellipsoids.

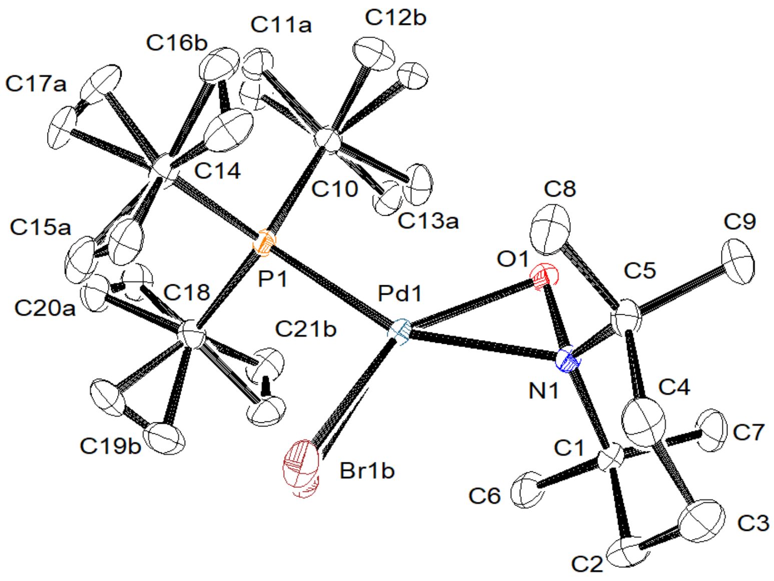

Figure S205. Structural drawing of **5b** with 50% probability anisotropic displacement ellipsoids.

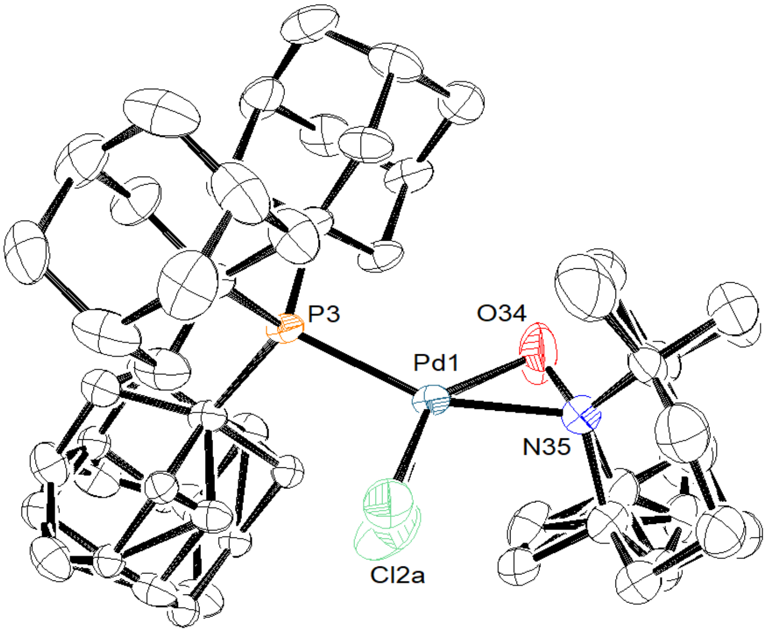

Figure S206. Structural drawing of **S2** with 50% probability anisotropic displacement ellipsoids.

Extinction coefficient data

Measurements were performed on an Agilent Cary 8454 Spectrophotometer. Samples were prepared by successive addition of aliquots of a stock solution of either complex to a cuvette containing 3 mL of $CDCl_3$ under air.

Table S40. Absorbance vs. concentration data for 1a

concentration (M)	absorbance (370 nm)
$1.9*10^{-6}$	0.0002
$6.5*10^{-6}$	0.0090
$1.1*10^{-5}$	0.0186
$1.6*10^{-5}$	0.0287
$2.0*10^{-5}$	0.0390
$2.5*10^{-5}$	0.0502
$4.4*10^{-5}$	0.0906
$6.2*10^{-5}$	0.1297

Figure S207. Plot of absorbance vs. concentration for 1a, for calculating extinction coefficient

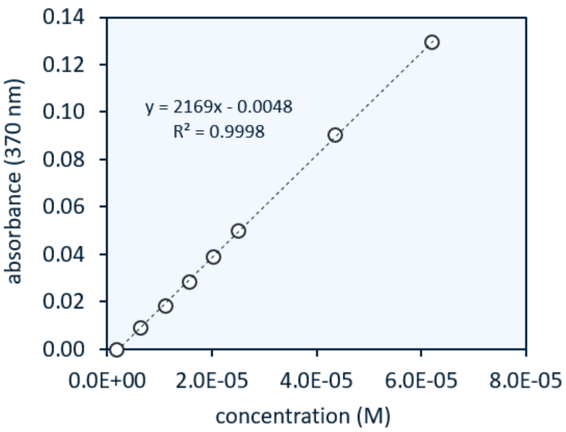

Figure S208. Overlay of absorbance traces of 1a at different conc.s, units of traces in M

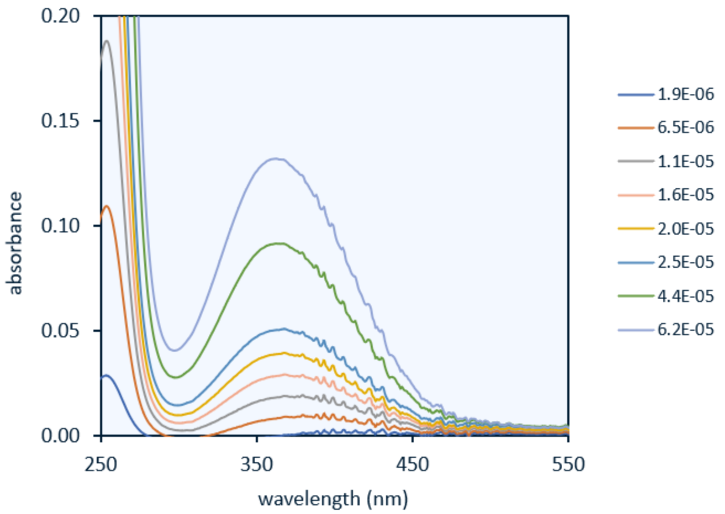

Table S41. Absorbance vs. concentration data for 3

concentration (M)	absorbance (370 nm)
$1.2*10^{-6}$	0.010
$4.2*10^{-6}$	0.019
$7.1*10^{-6}$	0.032
$1.0*10^{-5}$	0.044
$1.3*10^{-5}$	0.058
$1.6*10^{-5}$	0.072
$1.9*10^{-5}$	0.083
$3.1*10^{-5}$	0.134
$4.3*10^{-5}$	0.186

Figure S209. Plot of absorbance vs. concentration for 3, for calculating extinction coefficient, units of traces in M

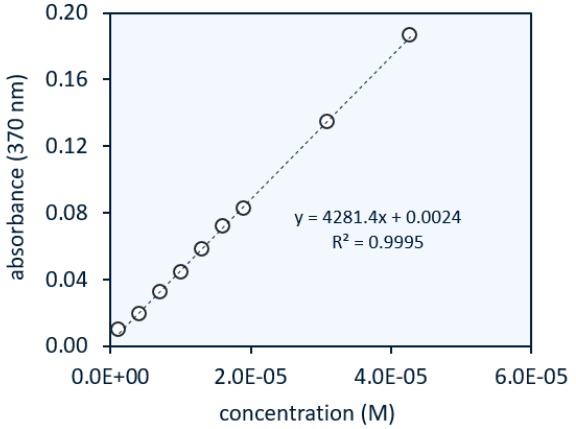

Figure S210. Overlay of absorbance traces of 3 at different conc.s, units of traces in M

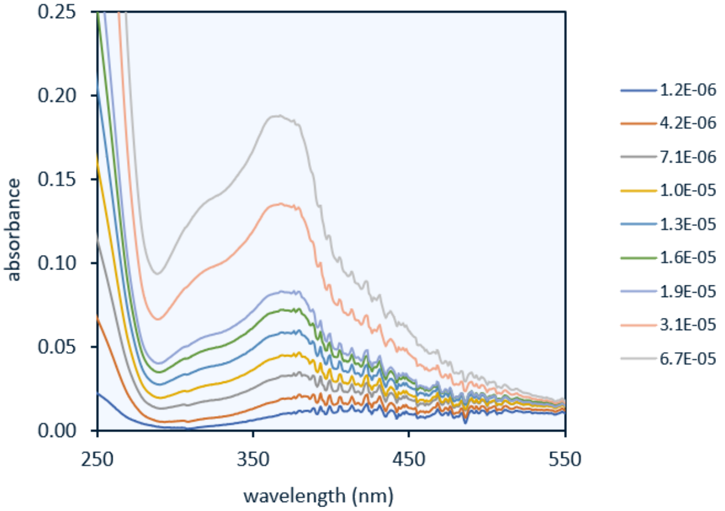

References

1. J. Wiedermann, K. Mereiter and K. Kirchner, Palladium imine and amine complexes derived from 2-thiophenecarboxaldehyde as catalysts for the Suzuki cross-coupling of aryl bromides, *J. Mol. Catal. A: Chem.*, 2006, **257**, 67.
2. D. Evrard, D. Lucas, Y. Mugnier, P. Meunier and J.-C. Hierso, On the Mechanistic Behavior of Highly Efficient Palladium−Tetraphosphine Catalytic Systems for Cross-Coupling Reactions: First Spectroscopic and Electrochemical Studies of Oxidative Addition on Pd(0)/Multidentate Ferrocenylpolyphosphine Complexes, *Organometallics*, 2008, **27**, 2643.
3. E. V. Salo and Z. B. Guan, Late-transition-metal complexes with bisazaferrocene ligands for ethylene oligomerization, *Organometallics*, 2003, **22**, 5033.
4. E. V. Salo and Z. Guan, Late-Transition-Metal Complexes with Bisazaferrocene Ligands for Ethylene Oligomerization, *Organometallics*, 2003, **22**, 5033.
5. L. Y. Chen, P. Ren and B. P. Carrow, Tri(1-adamantyl)phosphine: Expanding the Boundary of Electron-Releasing Character Available to Organophosphorus Compounds, *J. Am. Chem. Soc.*, 2016, **138**, 6392.
6. M. Yamashita, I. Takamiya, K. Jin and K. Nozaki, Syntheses and structures of bulky monophosphine-ligated methylpalladium complexes: Application to homo- and copolymerization of norbornene and/or methoxycarbonylnorbornene, *Organometallics*, 2006, **25**, 4588.
7. C. C. C. J. Seechurn, T. Sperger, T. G. Scrase, F. Schoenebeck and T. J. Colacot, Understanding the Unusual Reduction Mechanism of Pd(II) to Pd(I): Uncovering Hidden Species and Implications in Catalytic Cross-Coupling Reactions, *J. Am. Chem. Soc.*, 2017, **139**, 5194.
8. L. Y. Chen, H. Francis and B. P. Carrow, An "On-Cycle" Precatalyst Enables Room-Temperature Polyfluoroarylation Using Sensitive Boronic Acids, *ACS Catal.*, 2018, **8**, 2989.
9. H. G. Lee, P. J. Milner and S. L. Buchwald, An Improved Catalyst System for the Pd-Catalyzed Fluorination of (Hetero)Aryl Triflates, *Org. Lett.*, 2013, **15**, 5602.
10. W. E. Piers, A. J. V. Marwitz and L. G. Mercier, Mechanistic Aspects of Bond Activation with Perfluoroarylboranes, *Inorg. Chem.*, 2011, **50**, 12252.
11. Y. N. Ji, D. A. DiRocco, C. M. Hong, M. K. Wismer and M. Reibarkh, Facile Quantum Yield Determination via NMR Actinometry, *Org. Lett.*, 2018, **20**, 2156.
12. D. Kurandina, M. Parasram and V. Gevorgyan, Visible Light-Induced Room-Temperature Heck Reaction of Functionalized Alkyl Halides with Vinyl Arenes/Heteroarenes, *Angew. Chem. Int. Ed.*, 2017, **56**, 14212.
13. Frisch, M. J.; Trucks, G. W.; Schlegel, H. B.; Scuseria, G. E.; Robb, M. A.; Cheeseman, J. R.; Scalmani, G.; Barone, V.; Petersson, G. A.; Nakatsuji, H.; Li, X.; Caricato, M.; Marenich, A. V.; Bloino, J.; Janesko, B. G.; Gomperts, R.; Mennucci, B.; Hratchian, H. P.; Ortiz, J. V.; Izmaylov, A. F.; Sonnenberg, J. L.; Williams; Ding, F.; Lipparini, F.; Egidi, F.; Goings, J.; Peng, B.; Petrone, A.; Henderson, T.; Ranasinghe, D.; Zakrzewski, V. G.; Gao, J.; Rega, N.; Zheng, G.; Liang, W.; Hada, M.; Ehara, M.; Toyota, K.; Fukuda, R.; Hasegawa, J.;

Ishida, M.; Nakajima, T.; Honda, Y.; Kitao, O.; Nakai, H.; Vreven, T.; Throssell, K.; Montgomery Jr., J. A.; Peralta, J. E.; Ogliaro, F.; Bearpark, M. J.; Heyd, J. J.; Brothers, E. N.; Kudin, K. N.; Staroverov, V. N.; Keith, T. A.; Kobayashi, R.; Normand, J.; Raghavachari, K.; Rendell, A. P.; Burant, J. C.; Iyengar, S. S.; Tomasi, J.; Cossi, M.; Millam, J. M.; Klene, M.; Adamo, C.; Cammi, R.; Ochterski, J. W.; Martin, R. L.; Morokuma, K.; Farkas, O.; Foresman, J. B.; Fox, D. J. Gaussian 16 Rev. B.01, Wallingford, CT, 2016.
14. T. H. Dunning Jr. and P. J. Hay, in Modern Theoretical Chemistry, Ed. H. F. Schaefer III, Vol. 3 (Plenum, New York, 1977) 1-28.
15. R. Krishnan, J. S. Binkley, R. Seeger and J. A. Pople, Self-Consistent Molecular-Orbital Methods .20. Basis Set for Correlated Wave-Functions, *J. Chem. Phys.*, 1980, **72**, 650.
16. S. Grimme, J. Antony, S. Ehrlich and H. Krieg, A consistent and accurate ab initio parametrization of density functional dispersion correction (DFT-D) for the 94 elements H-Pu, *J. Chem. Phys.*, 2010, **132**, 154104.
17. A. V. Marenich, C. J. Cramer and D. G. Truhlar, Universal Solvation Model Based on Solute Electron Density and on a Continuum Model of the Solvent Defined by the Bulk Dielectric Constant and Atomic Surface Tensions, *J. Phys. Chem. B*, 2009, **113**, 6378.
18. P. J. Stephens, F. J. Devlin, C. F. Chabalowski and M. J. Frisch, Ab-Initio Calculation of Vibrational Absorption and Circular-Dichroism Spectra Using Density-Functional Force-Fields, *J. Phys. Chem.*, 1994, **98**, 11623.
19. Y. Zhao and D. G. Truhlar, The M06 suite of density functionals for main group thermochemistry, thermochemical kinetics, noncovalent interactions, excited states, and transition elements: two new functionals and systematic testing of four M06-class functionals and 12 other functionals, *Theor. Chem. Acc.*, 2008, **120**, 215.
20. T. Yanai, D. P. Tew and N. C. Handy, A new hybrid exchange-correlation functional using the Coulomb-attenuating method (CAM-B3LYP), *Chem. Phys. Lett.*, 2004, **393**, 51.
21. T. Lu and F. W. Chen, Multiwfn: A multifunctional wavefunction analyzer, J Comput Chem, 2012, 33, 580.
22. A. J. Cohen and N. C. Handy, Dynamic correlation, Mol Phys, 2001, 99, 607.
23. Frisch, M. J.; Trucks, G. W.; Schlegel, H. B.; Scuseria, G. E.; Robb, M. A.; Cheeseman, J. R.; Scalmani, G.; Barone, V.; Petersson, G. A.; Nakatsuji, H.; Li, X.; Caricato, M.; Marenich, A. V.; Bloino, J.; Janesko, B. G.; Gomperts, R.; Mennucci, B.; Hratchian, H. P.; Ortiz, J. V.; Izmaylov, A. F.; Sonnenberg, J. L.; Williams; Ding, F.; Lipparini, F.; Egidi, F.; Goings, J.; Peng, B.; Petrone, A.; Henderson, T.; Ranasinghe, D.; Zakrzewski, V. G.; Gao, J.; Rega, N.; Zheng, G.; Liang, W.; Hada, M.; Ehara, M.; Toyota, K.; Fukuda, R.; Hasegawa, J.; Ishida, M.; Nakajima, T.; Honda, Y.; Kitao, O.; Nakai, H.; Vreven, T.; Throssell, K.; Montgomery Jr., J. A.; Peralta, J. E.; Ogliaro, F.; Bearpark, M. J.; Heyd, J. J.; Brothers, E. N.; Kudin, K. N.; Staroverov, V. N.; Keith, T. A.; Kobayashi, R.; Normand, J.; Raghavachari, K.; Rendell, A. P.; Burant, J. C.; Iyengar, S. S.; Tomasi, J.; Cossi, M.; Millam, J. M.; Klene, M.; Adamo, C.; Cammi, R.; Ochterski, J. W.; Martin, R. L.; Morokuma, K.; Farkas, O.; Foresman, J. B.; Fox, D. J. Gaussian 16 Rev. B.01, Wallingford, CT, 2016.
24. T. H. Dunning Jr. and P. J. Hay, in Modern Theoretical Chemistry, Ed. H. F. Schaefer III, Vol. 3 (Plenum, New York, 1977) 1-28.
25. M. Kubista, R. Sjoback, S. Eriksson and B. Albinsson, Experimental Correction for the Inner-Filter Effect in Fluorescence-Spectra, *Analyst*, 1994, **119**, 417.
26. A. V. Fonin, A. I. Sulatskaya, I. M. Kuznetsova and K. K. Turoverov, Fluorescence of Dyes in Solutions with High Absorbance. Inner Filter Effect Correction, *Plos One*, 2014, **9**, e103878.
27. B. J. Shields, B. Kudisch, G. D. Scholes and A. G. Doyle, Long-Lived Charge-Transfer States of Nickel(II) Aryl Halide Complexes Facilitate Bimolecular Photoinduced Electron Transfer, *J. Am. Chem. Soc.*, 2018, **140**, 3035.
